# Domain-Based Identification and Analysis of Glutamate Receptor Ion Channels and Their Relatives in Prokaryotes

**DOI:** 10.1371/journal.pone.0012827

**Published:** 2010-10-06

**Authors:** Mao-Feng Ger, Gloria Rendon, Jeffrey L. Tilson, Eric Jakobsson

**Affiliations:** 1 Center for Biophysics and Computational Biology, University of Illinois at Urbana-Champaign, Urbana, Illinois, United States of America; 2 National Center for Supercomputing Applications, Urbana, Illinois, United States of America; 3 Renaissance Computing Institute, Raleigh, North Carolina, United States of America; University of Oxford, United Kingdom

## Abstract

Voltage-gated and ligand-gated ion channels are used in eukaryotic organisms for the purpose of electrochemical signaling. There are prokaryotic homologues to major eukaryotic channels of these sorts, including voltage-gated sodium, potassium, and calcium channels, Ach-receptor and glutamate-receptor channels. The prokaryotic homologues have been less well characterized functionally than their eukaryotic counterparts. In this study we identify likely prokaryotic functional counterparts of eukaryotic glutamate receptor channels by comprehensive analysis of the prokaryotic sequences in the context of known functional domains present in the eukaryotic members of this family. In particular, we searched the nonredundant protein database for all proteins containing the following motif: the two sections of the extracellular glutamate binding domain flanking two transmembrane helices. We discovered 100 prokaryotic sequences containing this motif, with a wide variety of functional annotations. Two groups within this family have the same topology as eukaryotic glutamate receptor channels. Group 1 has a potassium-like selectivity filter. Group 2 is most closely related to eukaryotic glutamate receptor channels. We present analysis of the functional domain architecture for the group of 100, a putative phylogenetic tree, comparison of the protein phylogeny with the corresponding species phylogeny, consideration of the distribution of these proteins among classes of prokaryotes, and orthologous relationships between prokaryotic and human glutamate receptor channels. We introduce a construct called the Evolutionary Domain Network, which represents a putative pathway of domain rearrangements underlying the domain composition of present channels. We believe that scientists interested in ion channels in general, and ligand-gated ion channels in particular, will be interested in this work. The work should also be of interest to bioinformatics researchers who are interested in the use of functional domain-based analysis in evolutionary and functional discovery.

## Introduction

**Figure 1:**
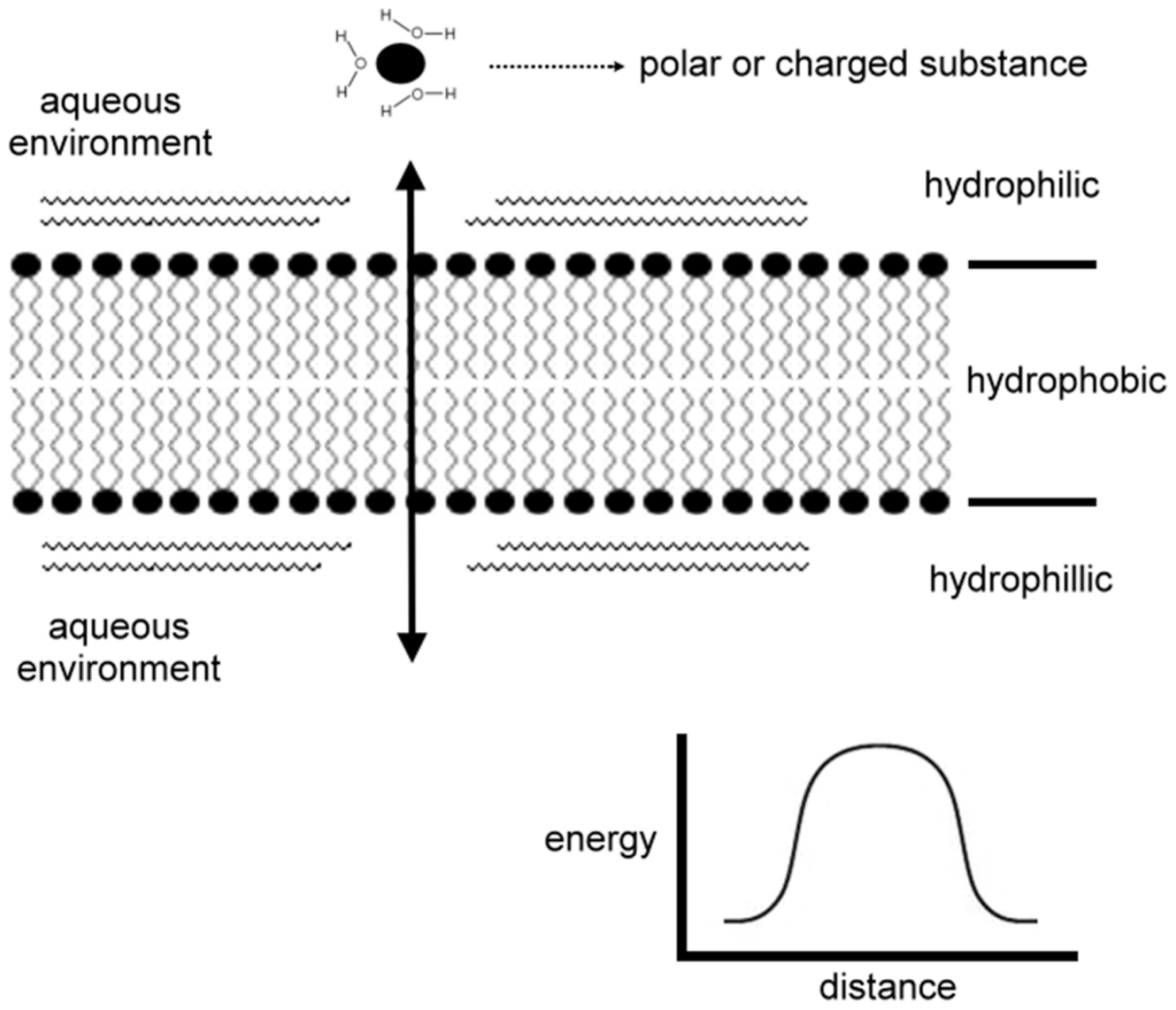
Energy barrier for an ion to move across the membrane.

It is estimated that 20%–40% of genes code for integral membrane proteins in archaea, bacteria, and eukaryote [1]. Because of the enormous energy barrier associated with moving ions across lipid bilayers [2] (Figure 1), proteins are essential for the transmembrane movement of polar and charged substances. Specific transmembrane proteins, like ion channels, transporters and pumps, appear to have arisen in very early forms of cellular life [3].

**Figure 2:**
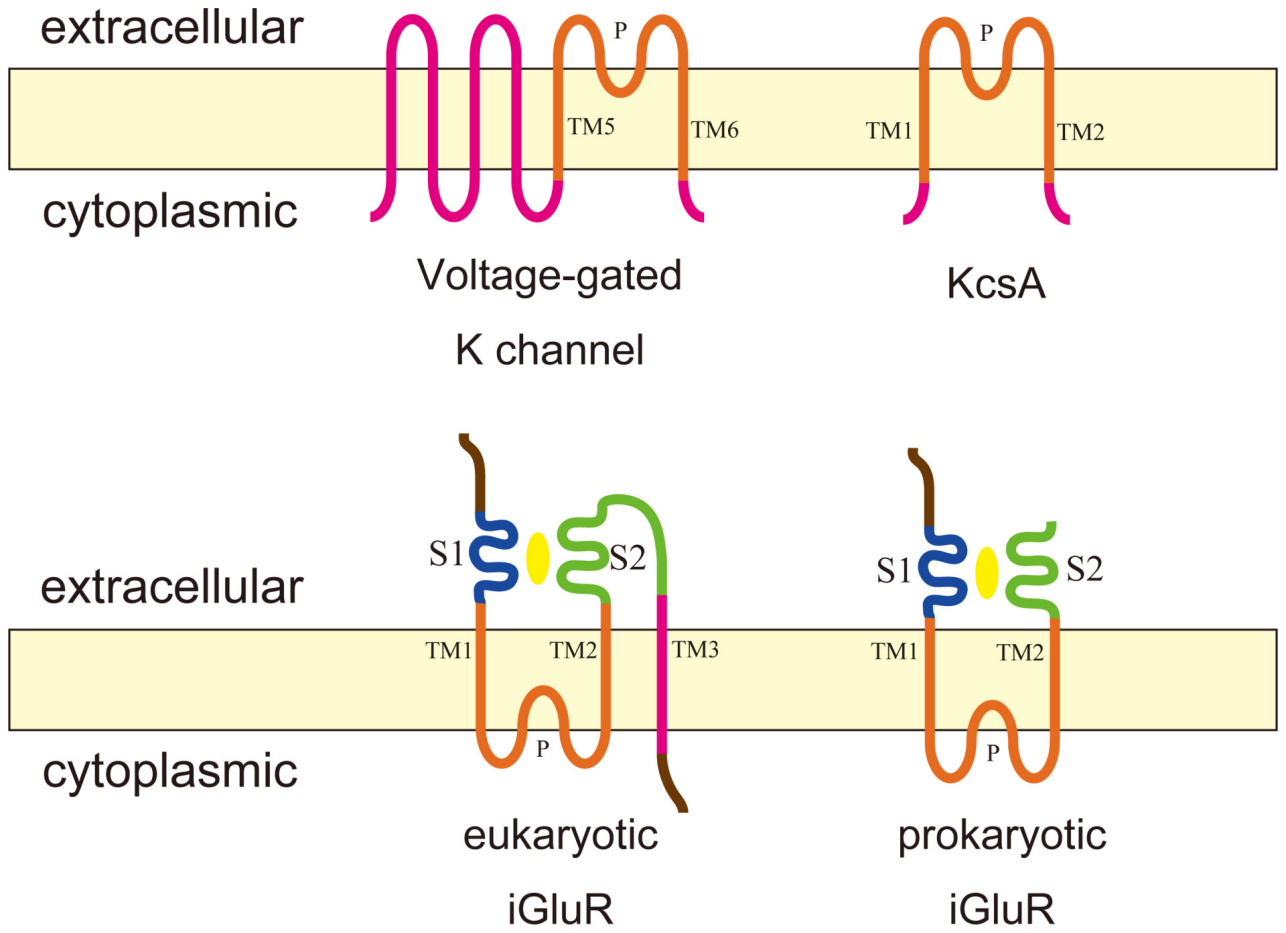
Topology diagrams of pore-loop ion channels.

Ion channels are specialized transmembrane proteins through which cations or anions move passively down the electrochemical gradients that are created by ion pumps. Ion channels differ greatly in their structural and functional properties and are classified by their selectivity (Na^+^, K^+^, Ca^2+^ and Cl^−^) and activation mechanism (voltage-gated or ligand-gated). The largest subfamily of ion channels is comprised of the pore-loop channels, all of which carry a basic structural unit – a re-entrant pore-loop flanked by two transmembrane helices (TM's). (Figure 2) The ion selectivity is conferred by the pore-loop [4]. This common topology can be interpreted to suggest that the pore-loop channels have a common ancestor. This suggestion was born out by the discovery of a prokaryotic channel that contained the ligand-binding extracellular domain characteristic of glutamate receptor channels but a pore-loop characteristic of a potassium channel [5].

Glutamate, a major excitatory neurotransmitter, activates two receptor families: metabotropic glutamate receptor proteins (mGluR), which activate biochemical cascades, and ionotropic glutamate receptors, which form cation selective ion channels (iGluR) and are members of the pore-loop subfamily. Compared to the voltage-gated members of the pore-loop subfamily, iGluR's have opposite transmembrane orientation to the others (the pore-loop re-enters from the intracellular side). There are three major eukaryotic iGluR's subtypes, the AMPA, kainite and NMDA receptors, which form cation channels permeable to Na^+^, K^+^ and Ca^2+^. Because of the difficulty of purification and crystallization of integral membrane proteins, we only have the high resolution structure for the extracellular ligand-binding domain of iGluR [6]. Some critical amino acids are identified in ligand-binding sequence.

In addition to the above-mentioned glutamate-receptor channel homologue, many other homologues to mammalian ion channels have been found in sequenced prokaryotic genomes, such as K^+^ channels, Na^+^ channels, and Cl^−^ channels [7]. In addition Kuner, et al [8] noted the existence of other prokaryotic sequences bearing a resemblance to eukaryotic glutamate receptor channels.

The relative simplicity of prokaryotic ion channels makes them excellent objects for biophysical research [9]. A particularly notable example is the use of a prokaryotic potassium channel to make the first high resolution structure determination of voltage gated channels [10]. In many ways studying prokaryotic homologues can shed significant light on eukaryotic channels, as well the prokaryotic channels being of interest in their own right. For these reasons, a few years ago our laboratory (in collaboration with the laboratory of I. Aravind at NIH) set out to find prokaryotic homologues to the Ach receptor channel family. A straightforward BLAST [11] search yielded no results. We therefore undertook a search based on finding sequences with conserved domains characteristic of Ach receptor channel proteins and with the appropriate topology. That approach yielded a number of predicted prokaryotic members of this channel family [12]. One of our predicted channes was cloned, expressed, and functionally characterized as a channel [13] and high resolution structures were determined [14]. We anticipate that comprehensive identification of members of this group will lead to further functional and structural characterization of this family of channels, as well as insights into evolutionary and comparative aspects of channel biology. In the present study we extend this approach to a systematic domain-based search to identify and characterize in the nonredundant protein database all the prokaryotic homologues of the glutamate receptor channel family; i.e., prokaryotic iGluR's.

## Materials and Methods

### Searching for Prokaryotic iGluR's

**Figure 3:**
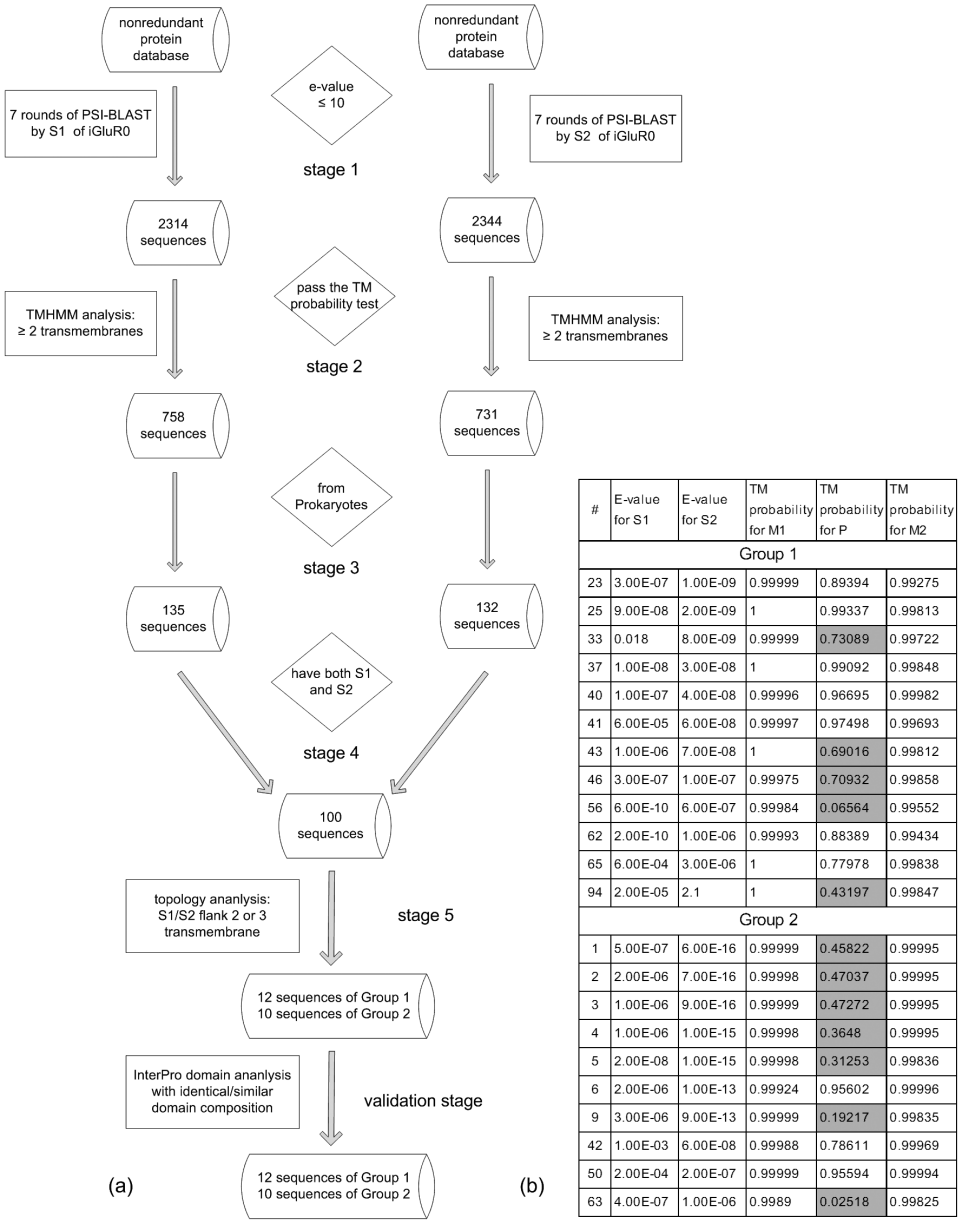
The searching strategy for finding prokaryotic iGluR's and the statistical proof. The strategy in (a) includes 5 stages and an additional validation stage. At each stage, we select protein sequences which are qualified for the requirements. In (b), the statistical e-values for S1 and S2 identification and TM probability scores by PSI-BLAST and TMHMM, respectively. The TM probability scores which do not pass the TM probability test are shaded in (b) and not counted as TM'S.

The overall strategy for discovery of the prokaryotic iGluR's is provided in the flow chart of the five stage screening process, plus a validation stage using the InterPro database, in Figure 3(a).

We begin the search with the sequence iGluR0 from Synechocystic PCC6803 [15] which has been well characterized both functionally [5] and structurally [16]. At stage 1 in Figure 3a, we used PSI-BLAST [11] to search the SDSC nonredundant protein database for the S1 binding region (NSEYVRQNSISAGITAVAEGELDILIGPISVTPERAAIEGITFTQPYFSSGIGLLIP, 57 aa long). This returned 2314 sequences with an E-value below 10. We applied the same method separately with the S2 segment of the binding region (EAVMFDRPALIYYTRQNPNLNLEVTEIRVSLEPYGFVLKENSPLQKTINVEMLNLLYSRVIAEFTERWL, 69 aa long) and returned 2344 sequences. At stage 2 in Figure 3a, we invoked TransMembrane Hidden Markov Model [TMHMM] [17] to predict the number of transmembrane (TM) helices in each sequence. We eliminated all sequences with fewer than 2 TM's, which is the minimal number for the iGluR structure. This left us with 758 sequences with S1 and at least 2 TM's and with 731 sequences with S2 and at least 2 TM's. At stage 3, we separated the prokaryotic sequences from the eukaryotes. We found 135 sequences with S1 and 2 TM's and 132 sequences with S2 and 2 TM's. At stage 4, out of the 135 and the 132 we keep only the sequences that have both S1 and S2, which total 100 (see Data S1 for detailed list). The annotations of the 100 sequences, clearly related to each other, are varied. In the definition line of the SDSC nonredundant protein database, 51 of them are annotated as ABC-type amino acid transporter or something similar, 13 of them are annotated as binding proteins, 14 of them are annotated as hypothetical proteins, 2 of them are annotated as K channels, plus some other scattered annotations (Table 1).

**Table 1:** Annotation of 100 bacterial sequences found to contain glutamate binding domains and two transmembrane domains.

gene annotation	protein No.	quantity
ABC transport system glutamine-binding protein	1,5,7,15,18,31,36,39,53,58,59,61	12
ABC-type amino acid transport/signal	2,4,6,8,10,12,13,17,24,26,27,28,29,35,38,45,47,48, 49,54,55,64,69,71,77, 78,81,82,83,85,88,90,91,95, 96,97,98, 99,52	39
transporter	19,21	2
binding protein	66,80	2
extracellular solute-binding protein	9,25,33,40,41,46,63,65,70,73,89	11
hypothetical protein	16,20,22,43,50,56,57,60,67,72,75,76, 79,100	14
iGluR	3,23,37,62	4
K channel	42,94	2
sensory transduction protein kinase	34	1
sensory box protein	87	1
IMP dehydrogenase/GMP reductase	84	1
Unknown function	30,32,44,51,68,74,86,92,93	9

**Figure 4:**
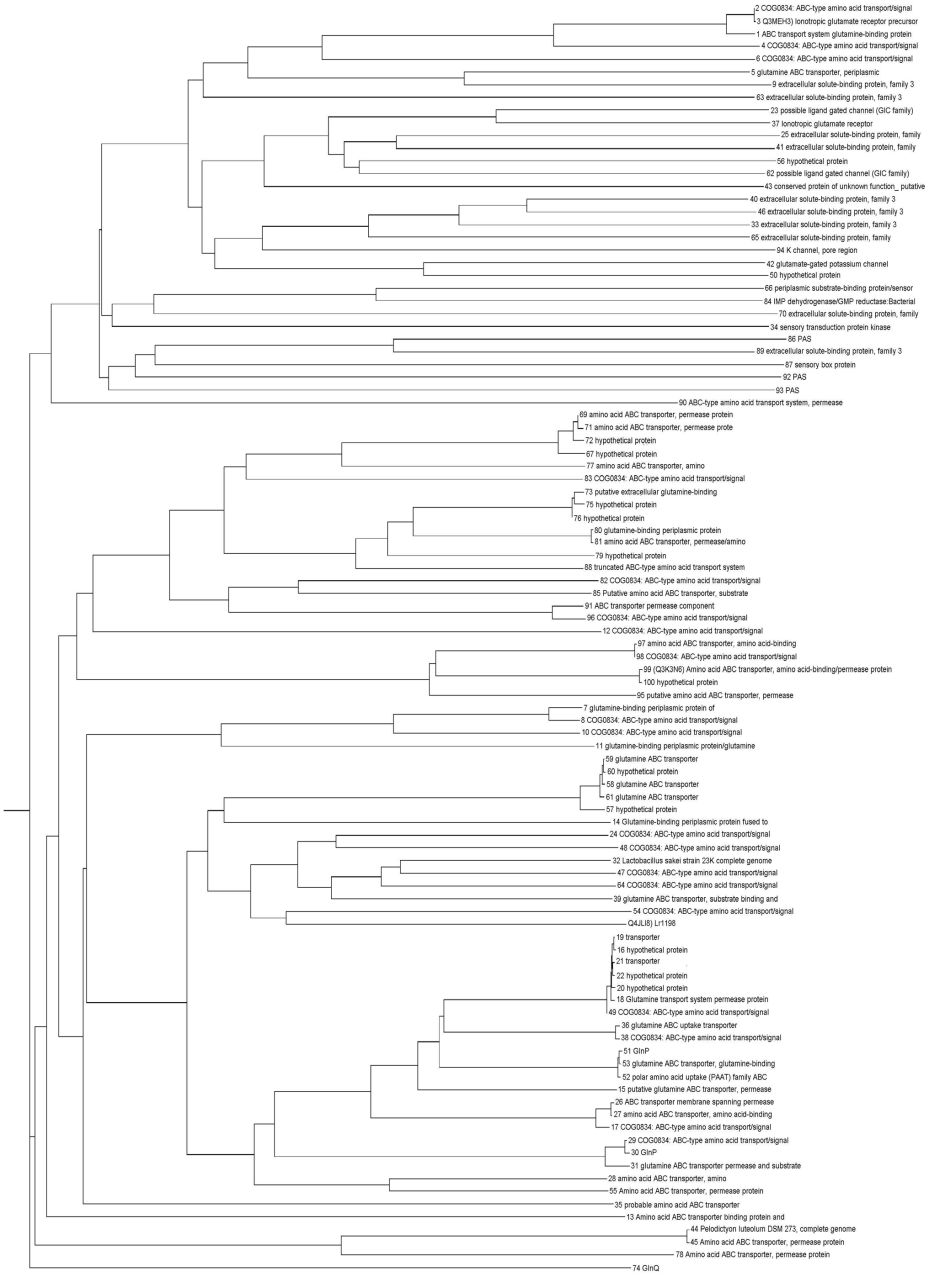
Phylogenetic tree for 100 sequences. Phylogenetic tree for 100 potential prokaryotic glutamate receptor channels as determined by presence of glutamate binding domain and transmembrane helices. (An electronic version of Figure 4 is included in supplementary materials to permit expansion for greater readability, Data S2.) The sequences are labeled with the definition line from the SDSC nonredundant protein database.

To explore the relationships among the 100 sequences, we aligned the sequences with ClustalW [18] and built a phylogenetic tree for them by DRAWGRAM [19]. The result is shown in Figure 4.

A notable feature of Figure 4 is that in many cases there is a disconnect between how close the sequences are on the tree and the similarity of the annotations. In some cases proteins that are quite similar are annotated differently, while sequences that seem quite far apart have the same annotation. A BLAST [11] of each of the 100 was done against the nonredundant database (data not shown) and confirmed that the sequence that gave the best hit was usually the one that was closest on the tree, and that the closest one on the tree was always one of the top few.

**Figure 5:**
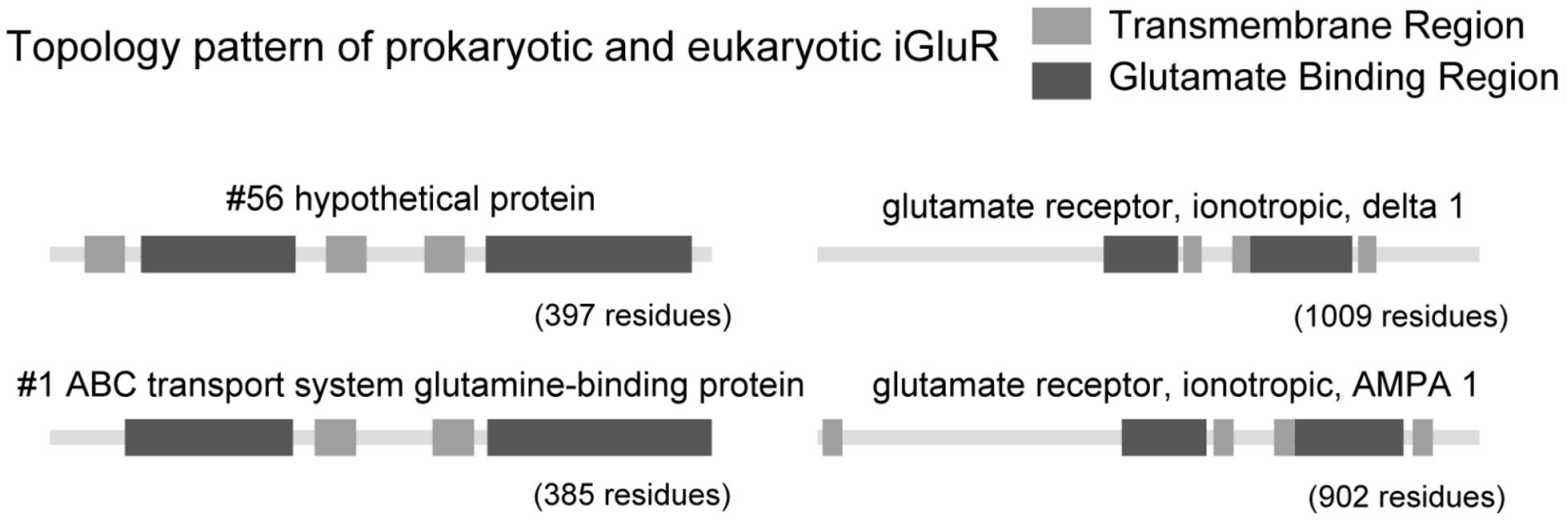
Topology pattern for Group 1 and Group 2. The eukaryotic counterpart of prokaryotic iGluR #56 is delta 1. The eukaryotic counterpart of prokaryotic iGluR #1 is AMPA 1. They are all with the structure of the S1 and S2 glutamate binding domains flanking two TM helices, in turn flanking a P-loop.

We then performed a topology analysis (stage 5 in Figure 3a) for the 100 sequences. The transmembrane regions are determined by TMHMM [17] and the glutamate binding regions are determined by sequence alignment. Through the visualization tool SeqVISTA [20], we can see the relative positions and lengths for TM's and glutamate binding regions in each protein. 22 of the 100 can be identified as having the characteristic topology of glutamate receptor channels; i.e., the S1 and S2 glutamate binding domains flanking two TM helices (M1 and M2 region), in turn flanking a pore-loop (a domain that looks like a partial TM helix, P region). (One of the 22 sequences is the authoritative sequence that we used as our initial probe [5].) Figure 3b shows the e-values and TM probability scores for the S1/S2 and TM regions of the 22 sequences. It is seen that the statistical evidence for the identification and the topology are very strong. Figure 5 shows the SeqVISTA pattern characteristic of these 22 sequences and, for comparison, the SeqVISTA pattern for the human glutamate receptor channel orthologous (by the standard of reciprocal best hits) to the particular prokaryotic sequence shown. There are some differences. The human proteins are much larger, having an extra TM near the C-terminus. But there is a major similarity, i.e., the glutamate binding domains flanking two TM domains and a pore-loop. The supplementary material (Data S3) includes the SeqVISTA diagrams for all 100 prokaryotic sequences in our search. Besides the 22 sequences, the other 78 prokaryotic sequences that have the glutamate binding domain and two or more TM helices have somewhat different topologies.

## Results

### Features and Evolution of the Prokaryotic Glutamate Receptor Channels

Of the 22 putative channels, 12 of them have a distinctive potassium channel selectivity filter. We designate these as our Group 1. The other 10 have P regions we do not recognize as distinctively similar to any channel with a known particular selectivity. Their annotations in the SDSC nonredundant protein database are shown in Table 2. Based on our analysis we would suggest that Group 1 be annotated as “putative glutamate-sensitive potassium channel” (except for #56, for which the word “putative” should be left off, since it has been functionally characterized as a glutamate-sensitive potassium channel [5].) We would suggest that Group 2 be annotated as “putative glutamate-sensitive ion channel”. Besides TM, we also used signalP [21] to test the existence of signal peptide. We found that two members of Group1 and two members of Group 2 lack the signal peptides which help the orientation of ion channel. The reasons for this may be the following: 1) They are pseudogenes; 2) they may have a different mechanism of inserting into membranes, or 3) they are oppositely oriented in the membrane than the other Group 1 and Group 2 channels. Motif searching has important significance in predicting the structures and functions of proteins. Therefore, we analyze the protein sequences by InterProScan [22] which is a web-based motif searching tool (http://www.ebi.ac.uk/interpro/) and federates 13 InterPro member databases into one resource. By searching the different protein signature databases, we can get a more comprehensive understanding of our target proteins. In order to efficiently utilize InterProScan, we developed a high throughput workflow around the InterProScan core program, that we call MotifNetwork [23].

**Table 2:** Gene list of Group 1 and Group 2.

	Group 1	Protein ID
23	Possible ligand gated channel (GIC family	NP_896860.1
25	extracellular solute-binding protein, family	ZP_00674117.1
33	extracellular solute-binding protein, family 3	YP_378562.1
37	Ionoropic glutamate receptor	YP_376778.1
40	extracellular solute-binding protein, family 3	ABB23418.1
41	extracellular solute-binding protein, family	ZP_00517290.1
43	conserved protein of unknown function_ putative	YP_339120.1
46	extracellular solute-binding protein, family 3	ZP_00660701.1
56	hypothetical protein	NP_441171.1
62	Possible ligand gated channel (GIC family)	NP_894348.1
65	extracellular solute-binding protein, family	ZP_00530895.1
94	K channel, pore region	ZP_00533070.1
	Group 2	
1	ABC transport system glutamine-binding protein	NP_486951.1
2	COG0834: ABC-type amino acid transport/signal	ZP_00157839.2
3	Q3MEH3) Ionotropic glutamate receptor precursor	ABA20613.1
4	COG0834: ABC-type amino acid transport/signal	ZP_00108493.1
5	glutamine ABC transporter, periplasmic	YP_168531.1
6	COG0834: ABC-type amino acid transport/signal	ZP_00053934.2
9	extracellular solute-binding protein, family 3	ZP_00622239.1
42	glutamate-gated potassium channel	YP_204476.1
50	hypothetical protein	YP_132561.1
63	extracellular solute-binding protein, family 3	ZP_00629025.1

Through MotifNetwork, we found that all 100 sequences have a glutamate binding motif, which was expected because we took glutamate binding region as our PSI-BLAST probe. We also found that none of the Group 1 or Group 2 members had a domain characteristic of ABC transporters, reinforcing our view, stated above, that such annotation for those particular sequences is in error.

**Figure 6:**
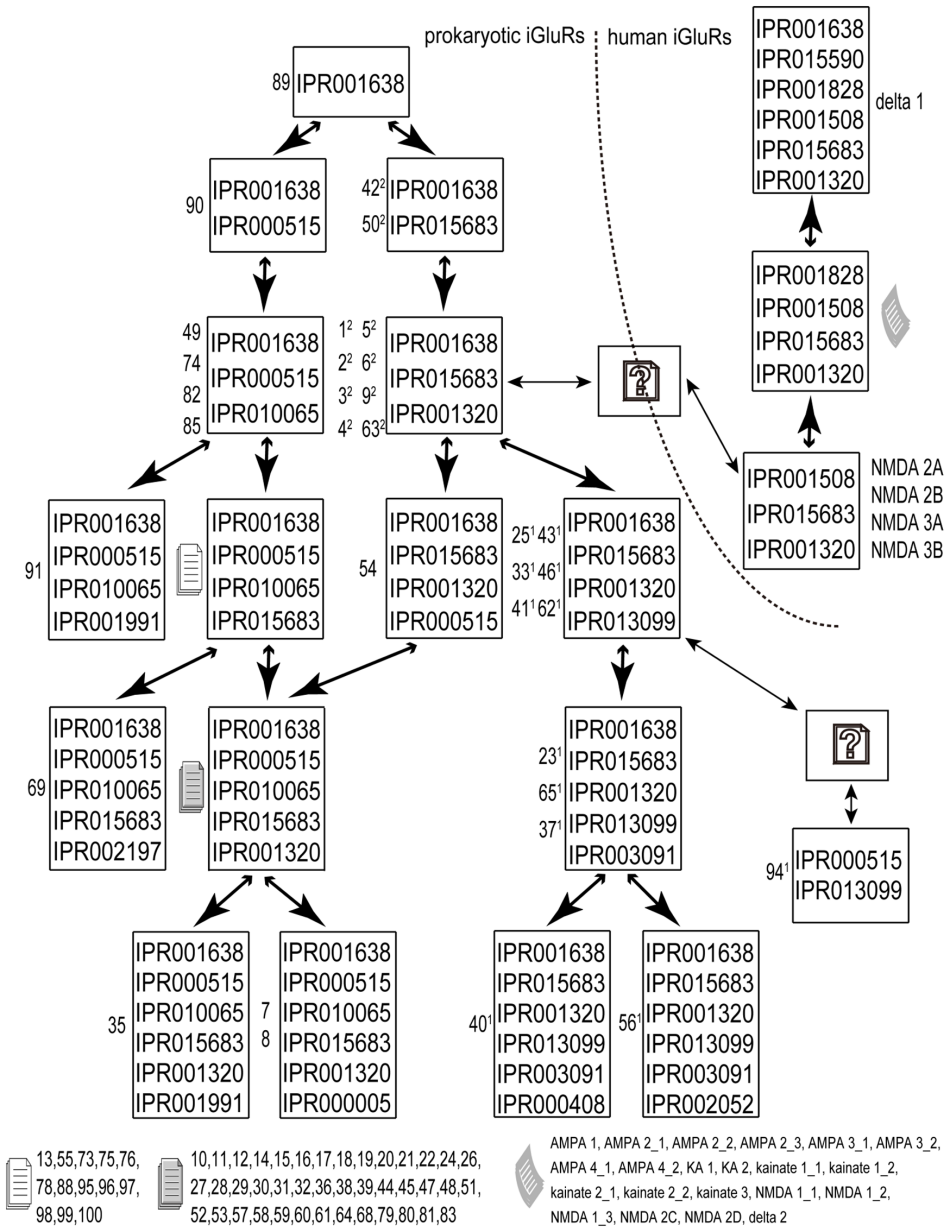
Evolutionary Domain Network of 100 sequences. IPR001638: Bacterial extracellular solute-binding protein, family 3. IPR015683: Glutamate receptor-related. IPR000515: Binding-protein-dependent transport systems inner membrane component. IPR010065: Amino acid ABC transporter, permease protein, 3-TM region, His/Glu/Gln/Arg/opine. IPR001320: Ionotropic glutamate receptor. IPR013099: Ion transport 2. IPR003091: Voltage-dependent potassium channel. IPR001991: Sodium:dicarboxylate symporter. IPR002197: Helix-turn-helix, Fis-type. IPR000005: Helix-turn-helix, AraC type. IPR000408: Regulator of chromosome condensation, RCC1. IPR002052: N-6 Adenine-specific DNA methylase. IPR001508: NMDA receptor. IPR001828: Extracellular ligand-binding receptor. IPR015590: Aldehyde dehydrogenase.

The results of the above are summarized in an Evolutionary Domain Network (EDN) (Figure 6). In the EDN representation, the proteins are grouped into domain sets according to the domain composition of each. (By “domain composistion” we mean the list of domains contained in the set.) The first row of the EDN contains all domain sets that consist of only a single domain. The second row contains those domain sets with two domains, the third row with three, etc. Tie lines are drawn between domain sets that can be derived from each other by the addition or subtraction of a single domain, representing roughly the evolutionary process of domain recombination. It should be noted that we have not screened out overlapping domains. Thus in some cases the same section of the protein sequence may be represented by two domain designations. We did attempt to screen overlaps, but any automated overlap screening resulted in loss of significant information, so we elected to report all MotifNetwork hits regardless of overlap.

By inspection of Figure 6, we see that all Group 1 sequences contain the IPR013099, whose short title is Ion transport 2. This domain represents a K^+^ channel selectivity filter. As far as we have been able to determine so far, the combination of glutamate channel binding site and potassium channel selectivity filter represented by Group 1 is only in bacteria. No members of Group 1 can be found in archaea, neither can Group 2.

All Group 2 sequences have two domains in common: IPR001638 (Bacterial extracellular binding protein) and IPR015638 (glutamate receptor related). These are overlapping regions. The selectivity filter and permeation pathway have not apparently been defined as a distinctive InterPro domain.

Just one domain set appears disconnected from the others, and is placed on the right hand side of Figure 6. This contains domains IPR000515 and IPR013099. Only one protein (#94) is contained in this domain set. The existence of the potassium channel selectivity filter, plus the orientation of the glutamate binding domains to the transmembrane domains, defines this as a Group 1 channel. However the domain IPR000515, with this one exception, is only associated with the other sequences that do not have the structure of the glutamate binding domains flanking two TM domains and a pore-loop. It thus appears that sequence 94, despite its outlier status in Figure 6, may be a part of a linkage between the channel proteins and the non-channel proteins in this study. The intermediate domain sets have either vanished or have not yet been sequenced.

Inspection of Figure 6 shows that Human iGluR's can be connected to the prokaryotic scheme by intermediate steps equivalent to the net exchange of IPR001508 with IPR0016308 between NMDA receptor channels and Group 2 prokaryotic channels. This implies that Group 2 proteins might share a closer relationship to eukaryotic iGluR's than other prokaryotic glutamate-binding proteins and NMDA's are closer to prokaryotic iGluR's than are other eukaryotic iGluR's. Delta 1 protein reacquired IPR001638 (otherwises only found in prokaryotes among the group we are studying) in its motif composition, which may result from a genetic recombination from outside (for example virus-mediated transfer from prokaryortes). It may be that some of the missing intermediates will appear in a more complete study of all the eukaryotic members of this family, which will be the subject of a future study.

### Sequence analysis of Group 1 and Group 2 sequences

In order to identify the possible functions of Group 1 and Group 2 prokaryotic genes, we first made a multiple sequence alignment. In order to optimize the alignment, we align the domains separately and then join the alignments. We used the domain definitions of Mayer et al. [16] for the S1, S2, and channel regions (M1, P and M2). The conservation comparison is listed as Table 3. We can see that Group 2 is more conserved in glutamate binding region than Group 1 but less conserved in channel region.

**Table 3:** Conservation comparison of Group 1 and Group 2.

		S1	S2	channel
Group 1	identical	10/97	1/132	17/115
Group 1	Strongly conserved	10/97	15/132	25/115
Group 1	Weakly conserved	9/97	12/132	10/115
Group 2	Identical	12/93	10/129	2/120
Group 2	Strongly conserved	16/93	17/129	15/120
Group 2	Weakly conserved	6/93	11/129	11/120

**Figure 7:**
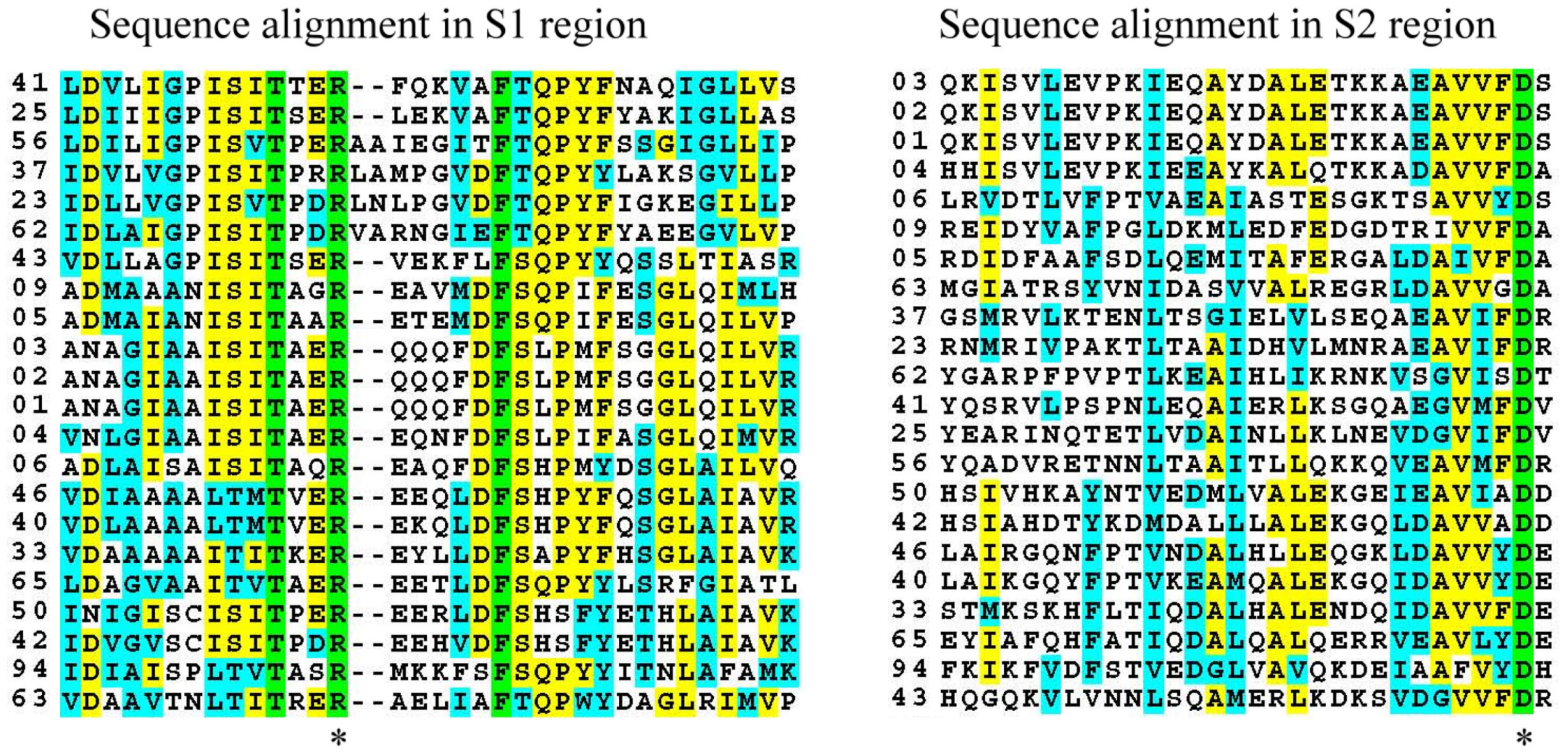
The sequence alignment in S1 and S2 region of Group1 and Group 2 proteins.

In previous research about prokaryotic iGluR, scientists have identified some amino acids which are important in glutamate binding [5], specifically an Arg in S1 which interacts with α–carboxy group of L-glutamate and an Asp in S2 which interacts with α–amino group of L-glutamate. These are totally conserved in the Group 1 and Group 2 alignments. This conservation is shown in Figure 7. (The full alignments are shown in Data S4).

### Phylogenetic analysis of Group 1 and Group 2 sequences

We made phylogenetic trees for the different regions (S1, S2, and P region) in Group 1 and Group 2 sequences. (Data S5) It is seen that the trees have essentially the same structure. We can conclude that the glutamate binding region and channel region have remained together for a long time in evolutionary history.

**Figure 8:**
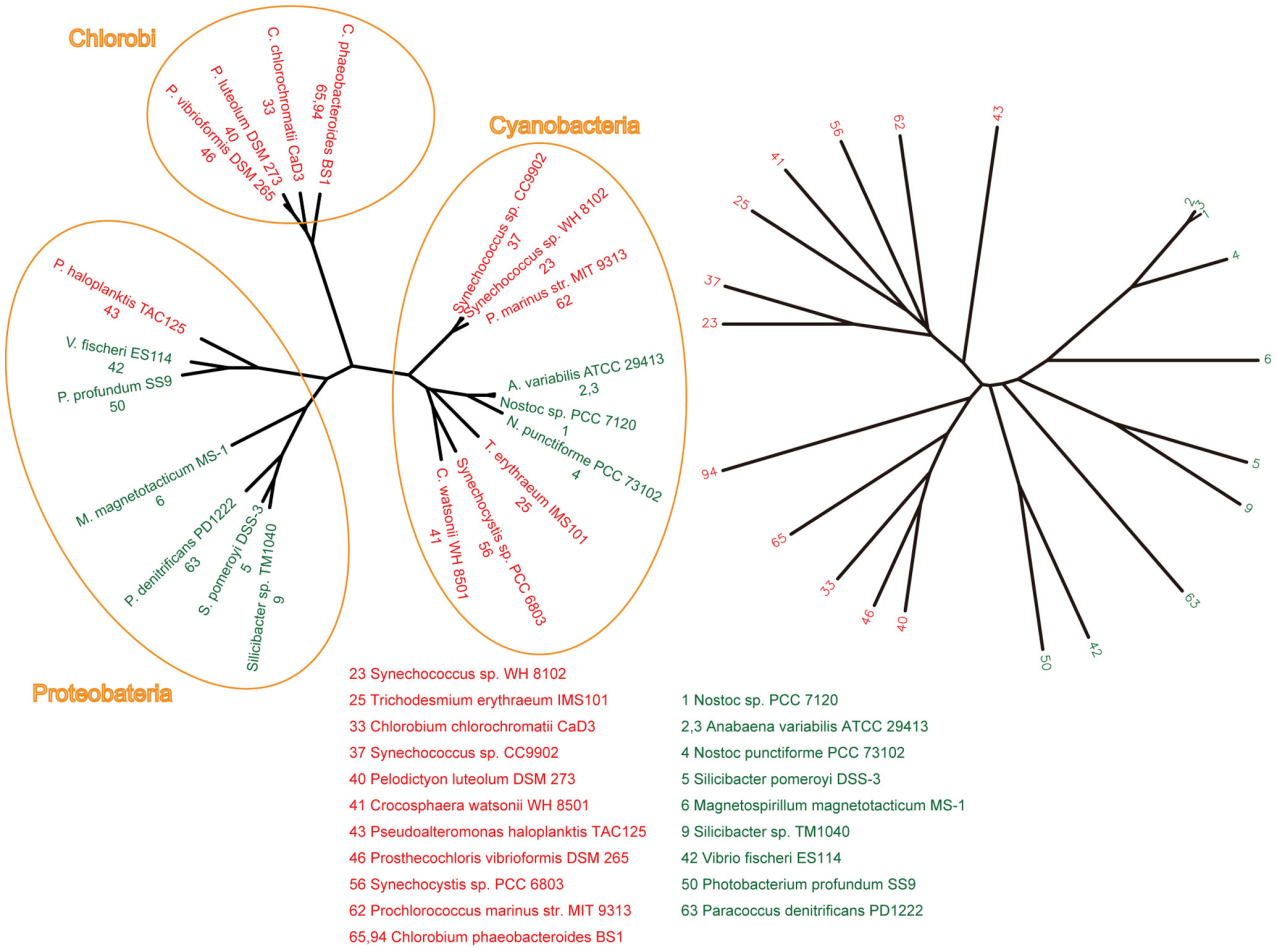
Phylogenetic trees of 16s rRNA genes and Group 1/Group 2 genes. Left hand side is the 16sRNA tree for the species that contain Group 1 and Group 2 prokaryotic glutamate receptor channels. Right hand side is the tree for the Group 1 and Group 2 proteins. The fact that the clustering patterns are different for the two trees indicates horizontal gene transfer of glutamate receptor channels among the bacteria. In particular, it seems there must have been a minimum of two transfers, one from cyanobacteria to proteobacteria, and one from proteobacteria to cyanobacteria.

We compared the phylogenetic tree of 16s rRNA genes with the phylogenetic tree of Group 1 and Group 2 genes in Figure 8. In this figure it is seen that in the tree of protein sequences (right hand tree) the Group 1 sequences (red) are clearly clustered together and separate from the Group 2 sequences (green). However in the 16s RNA sequences, the organisms containing Group 1 and Group 2 do not separate into distinct clusters from each other, indicating horizontal gene transfer (HGT) between the ancestors of some proteobacteria and some cyanobacteria.

### Comparison with eukaryotic glutamate receptor channels

Although iGluR research started with higher eukaryotic genomes, we still want to know if we can find all eukaryotic iGluR's by Group 1 and Group 2 sequences. First, we build a human iGluR list as a comparison by keyword search (Table 4).

**Table 4:** Human iGluR's.

AMPA	AMPA 1	NP_000818.1	906 aa
AMPA	AMPA 2 isoform 1	NP_000817.2	883 aa
AMPA	AMPA 2 isoform 2	NP_001077088.1	883 aa
AMPA	AMPA 2 isoform 3	NP_001077089.1	836 aa
AMPA	glutamate receptor 3 isoform flip	NP_015564.4	894 aa
AMPA	glutamate receptor 3 isoform flop	NP_000819.3	894 aa
AMPA	AMPA 4 isoform 1	NP_000820.3	902 aa
AMPA	AMPA 4 isoform 2	NP_001070711.2	884 aa
Kainate	kainate 1 isoform 1	NP_000821.1	918 aa
Kainate	kainate 1 isoform 2	NP_783300.1	905 aa
Kainate	kainate 2 isoform 1	NP_068775.1	908 aa
Kainate	kainate 2 isoform 2	NP_786944.1	869 aa
Kainate	kainite 3	NP_000822.2	919 aa
Kainate	glutamate receptor KA1	NP_055434.2	956 aa
Kainate	glutamate receptor KA2	NP_002079.3	980 aa
NMDA	NMDA receptor 1 isoform NR1-1	NP_000823.4	885 aa
NMDA	NMDA receptor 1 isoform NR1-2	NP_067544.1	901 aa
NMDA	NMDA receptor 1 isoform NR1-3	NP_015566.1	938 aa
NMDA	N-methyl-D-aspartate receptor subunit 2A	NP_000824.1	1464 aa
NMDA	N-methyl-D-aspartate receptor subunit 2D	NP_000825.2	1336 aa
NMDA	N-methyl-D-aspartate receptor subunit 2C	NP_000826.2	1233 aa
NMDA	N-methyl-D-aspartate receptor subunit 2B	NP_000827.2	1484 aa
NMDA	N-methyl-D-aspartate 3A	NP_597702.1	1115 aa
NMDA	N-methyl-D-aspartate 3B	NP_619635.1	1043 aa
Delta	delta 1	NP_060021.1	1009 aa
Delta	delta 2	NP_001501.2	1007 aa

Then, we used each of the Group1 and Group 2 as probes to blast human genome (BLASTP) [11], and accepted all hit with an e-value lower than 10. From the result (Table 5), we found that we can retrieve more human iGluR's using Group 2 as a probe. This implies that Group 2 sequences are closer to eukaryotic homologues than Group 1 sequences.

**Table 5:** Reverse BLAST result against human genome using Group 1 and Group2 as a probe.

Group 1		Group 2	
protein No.	ratio	protein No.	ratio
23	13/26	1	26/26
25	26/26	2	26/26
33	26/26	3	26/26
37	13/26	4	26/26
40	26/26	5	26/26
41	24/26	6	25/26
43	19/26	9	26/26
46	26/26	42	25/26
56	16/26	50	20/26
62	14/26	63	18/26
65	26/26		
94	26/26		

We also tested the orthologous relationship between eukaryotic iGluR prokaryotic iGluR by the “reciprocal-best-hits” criterion (data not shown). Both Group1 and Group2 members are orthologous to eukaryotic iGluR. This suggests two possible hypotheses. The first one is that Group 2 is the descendant of Group1 and eukaryotic iGluR is descendant of Group 2, because Group 2 is closer to eukaryotic iGluR in the phylogenetic map (data not shown). The other hypothesis is that eukaryotic iGluR is descendant of Group 2 and Group 1 is the combination of Group 2 and prokaryotic potassium channels.

## Discussion

Our results have implications for gene annotation, microbial communication and the evolution of cellular communication, and the origin and evolution of circadian rhythms.

### Gene Annotation

The gene products we identified as being homologous to ionotropic glutamate receptors are largely annotated otherwise. In this paper, we did individualized analysis to identify these gene products as likely ionotropic glutamate receptors. The key addition to the previous annotation comes from analysis by functional domains and by how those domains fit into the overall topology of the protein, especially where they are relative to the transmembrane helices. Our group has developed a high-throughput computational environment for such scanning (MotifNetwork) [23], based on the functional domain definitions in the InterPro database. MotifNetwork is being enhanced to consider topology as well, so we anticipate that the procedures described in this paper will ultimately be completely automated.

### Microbial Communication and the Evolution of Cellular Communication

In previous work our group used domain analysis to discover previously unknown prokaryotic members of the Ach Receptor Ion Channel family [12], a discovery which was later experimentally confirmed [13]. In this paper we extend the work to another major group of ligand-gated channels, the glutamate receptor channel family. These two discoveries together contribute to larger questions. What is the evolutionary origin of the electrochemical signaling mechanisms utilized in neuronal, neuromuscular, and neuroendocrine systems? To what extent do contemporary prokaryotes use these mechanisms to communicate? It should be noted that the patterns of occurrence of the two families of ligand-gated channels are very different. The prokaryotic Ach receptor channels are distributed across widely varying types of prokaryotes, both bacteria and archaea. By contrast, we found glutamate receptor channels only in bacteria, and clustered in particular bacterial subgroups. Because the sequence coverage of microbial genomes is still so sparse relative to the full range of microbial diversity, it is not possible to assess the full significance of this contrast. Based on our analysis of the existing data, it appears that horizontal transfer was the major mechanism for disseminating the prokaryotic members of the Ach receptor channel family. The members of the glutamate receptor channel family show evidence of at least two incidents of horizontal transfer (see Figure 8) but otherwise disseminate and variegate by descent. Based on the evolutionary domain network of the prokaryotic channels, it appears that domain reorganization was a significant factor in their evolution.

### Origin and Evolution of Circadian Rhythms

We note three facts:

1. Among all prokaryotes, cyanobacteria have been shown to exhibit circadian rhythms [24].
2. In this paper, we find that among prokaryotes, ionotropic glutamate receptor channels are disproportionately present in cyanobacteria.
3. In animal brain slice preparations, glutamate resets circadian rhythms in a manner similar to light [25].

From this combination of facts, we are moved to suggest that glutamate signaling may provide a link connecting the circadian regulation of animals and cyanobacteria. This suggestion needs to be tested by further work.

## Supporting Information

Data S1 Detailed information for 100 sequences included in this analysis.
(0.10 MB PDF)

Data S2 Phylogenetic tree for 100 sequences included in this analysis.
(0.78 MB PDF)

Data S3 Topology patterns for 100 sequences included in this analysis.
(9.04 MB PDF)

Data S4 Sequence alignments of S1, S2 and channel regions in Group 1 and Group 2.
(2.00 MB PDF)

Data S5 Phylogenetic trees of S1, S2 and channel regions in Group 1 and Group 2.
(0.10 MB PDF)
