# Supplementary material for: Domain-Based Identification and Analysis of Glutamate Receptor Ion Channels and Their Relatives in Prokaryotes
Source: PLoS One. 2010 Oct 6;5(10):e12827. doi: 10.1371/journal.pone.0012827 (PMC2950845; doi:10.1371/journal.pone.0012827)
Supplement: Data S1 — Detailed information for 100 sequences included in this analysis. (0.10 MB PDF) [file pone.0012827.s001.pdf]

#	NRDB #	gene annotation	species	protein acc #	nucleotide acc #	aa
1	1460795	ABC transport system glutamine-binding protein	Nostoc sp. PCC 7120	NP_486951.1	NC_003272	385
2	3455650	COG0834: ABC-type amino acid transport/signal	Anabaena variabilis ATCC 29413	ZP_00157839.2	NZ_AAEA01000050	356
3	4367368	Q3MEH3) Ionotropic glutamate receptor precursor	Anabaena variabilis ATCC 29413			385
4	2674627	COG0834: ABC-type amino acid transport/signal	Nostoc punctiforme PCC 73102	ZP_00108493.1	NZ_AAAY02000049	405
5	3574492	glutamine ABC transporter, periplasmic	Silicibacter pomeroyi DSS-3	YP_168531.1	NC_003911	361
6	2998022	COG0834: ABC-type amino acid transport/signal	Magnetospirillum magnetotacticum MS-1	ZP_00053934.2	NZ_AAAP01003795	355
7	1461065	glutamine-binding periplasmic protein of	Nostoc sp. PCC 7120	NP_487227.1	NC_003272	501
8	3466224	COG0834: ABC-type amino acid transport/signal	Anabaena variabilis ATCC 29413	ZP_00161034.2	NZ_AAEA01000006	501
9	4110730	extracellular solute-binding protein, family 3	Silicibacter sp. TM1040	ZP_00622239.1	NZ_AAFG02000007	354
10	2693307	COG0834: ABC-type amino acid transport/signal	Nostoc punctiforme PCC 73102	ZP_00107004.1	NZ_AAAY02000074	501
11	25947	glutamine-binding periplasmic protein/glutamine	Synechocystis sp. PCC 6803	NP_440904.1	NC_000911	530
12	3459274	COG0834: ABC-type amino acid transport/signal	Nostoc punctiforme PCC 73102	ZP_00109899.2	NZ_AAAY02000016	496
13	3657767	Amino acid ABC transporter binding protein and	Gluconobacter oxydans 621H	YP_191970.1	NC_006677	508
14	797334	Glutamine-binding periplasmic protein fused to	Clostridium acetobutylicum ATCC 824	NP_346756.1	NC_003030	477
15	1972140	putative glutamine ABC transporter, permease	Streptococcus mutans UA159	NP_721218.1	NC_004350	728
16	730124	hypothetical protein	Streptococcus pyogenes M1 GAS	NP_269437.1	NC_002737	724
17	4149401	COG0834: ABC-type amino acid transport/signal	Streptococcus pneumoniae TIGR4	ZP_00404160.1	NZ_AAGY01000014	539
18	3236238	Glutamine transport system permease protein	Streptococcus pyogenes MGAS10394	YP_060363.1	NC_006086	724
19	4239816	transporter	Streptococcus pyogenes MGAS5005	YP_282439.1	NC_007297	724
20	1889490	hypothetical protein	Streptococcus pyogenes SSI-1	NP_802123.1	NC_004606	724
21	4239581	transporter	Streptococcus pyogenes MGAS6180	YP_280525.1	NC_007296	724
22	1755498	hypothetical protein	Streptococcus pyogenes MGAS8232	NP_607432.1	NC_003485	724
23	2321967	possible ligand gated channel (GIC family)	Synechococcus sp. WH 8102	NP_896860.1	NC_005070	356
24	3459393	COG0834: ABC-type amino acid transport/signal	Leuconostoc mesenteroides subsp. mesentero	ZP_00063230.2	NZ_AABH02000012	463
25	2658706	extracellular solute-binding protein, family	Trichodesmium erythraeum IMS101	ZP_00674117.1	NZ_AABK04000007	360
26	1103184	ABC transporter membrane spanning permease	Streptococcus pneumoniae R6	NP_358713.1	NC_003098	721
27	794435	amino acid ABC transporter, amino acid-binding	Streptococcus pneumoniae TIGR4	NP_345706.1	NC_003028	721
28	2140426	amino acid ABC transporter, amino	Enterococcus faecalis V583	NP_814508.1	NC_004668	722
29	3860261	COG0834: ABC-type amino acid transport/signal	Lactococcus lactis subsp. cremoris SK11	ZP_00383312.1	NZ_AAGO01000019	704
30	3972343	GlnP	Lactococcus lactis subsp. cremoris	AAX82492.1	AY881998	714
31	656323	glutamine ABC transporter permease and substrate	Lactococcus lactis subsp. lactis Il1403	NP_267915.1	NC_002662	714

32	4521252	Lactobacillus sakei strain 23K complete genome	Lactobacillus sakei strain 23K	CAI55799.1	CR936503	478
33	4508428	extracellular solute-binding protein, family 3	Chlorobium chlorochromatii CaD3	YP_378562.1	NC_007514	352
34	2076917	sensory transduction protein kinase	Clostridium tetani E88	NP_782862.1	NC_004557	673
35	1518316	probable amino acid ABC transporter	Clostridium perfringens str. 13	NP_563009.1	NC_003366	502
36	3498920	glutamine ABC uptake transporter	Streptococcus thermophilus LMG 18311	YP_139919.1	NC_006448	736
37	4507879	Ionotropic glutamate receptor	Synechococcus sp. CC9902	YP_376778.1	NC_007513	357
38	3861175	COG0834: ABC-type amino acid transport/signal	Streptococcus thermophilus LMD-9	ZP_00388109.1	NZ_AAGS01000073	736
39	2080013	glutamine ABC transporter, substrate binding and	Lactobacillus plantarum WCFS1	NP_784545.1	NC_004567	478
40	4508147	extracellular solute-binding protein, family 3	Pelodictyon luteolum DSM 273	ABB23418.1	CP000096	378
41	2999572	extracellular solute-binding protein, family	Crocosphaera watsonii WH 8501	ZP_00517290.1	NZ_AADV02000060	368
42	3681965	glutamate-gated potassium channel	Vibrio fischeri ES114	YP_204476.1	NC_006840	366
43	4462221	conserved protein of unknown function_ putative	Pseudoalteromonas haloplanktis TAC125	YP_339120.1	NC_007481	349
44	4511146	Pelodictyon luteolum DSM 273, complete genome	Pelodictyon luteolum DSM 273	ABB23724.1	CP000096	990
45	4511158	Amino acid ABC transporter, permease protein	Pelodictyon luteolum DSM 273	YP_374941.1	NC_007512	990
46	4391792	extracellular solute-binding protein, family 3	Prosthecochloris vibrioformis DSM 265	ZP_00660701.1	NZ_AAJD01000001	383
47	3178539	COG0834: ABC-type amino acid transport/signal	Pediococcus pentosaceus ATCC 25745	ZP_00323477.1	NZ_AAEV01000004	483
48	3176719	COG0834: ABC-type amino acid transport/signal	Oenococcus oeni PSU-1	ZP_00320163.1	NZ_AABJ03000001	468
49	3644201	COG0834: ABC-type amino acid transport/signal	Streptococcus pyogenes M49 591	ZP_00365576.1	NZ_AAFV01000170	401
50	2959346	hypothetical protein	Photobacterium profundum SS9	YP_132561.1	NC_006371	372
51	760694	GlnP	Streptococcus agalactiae	AAK57382.1	AF372619	727
52	4422797	polar amino acid uptake (PAAT) family ABC	Streptococcus agalactiae A909	YP_330104.1	NC_007432	727
53	1919262	glutamine ABC transporter, glutamine-binding	Streptococcus agalactiae 2603V/R	NP_688460.1	NC_004116	727
54	3856627	COG0834: ABC-type amino acid transport/signal	Lactobacillus delbrueckii subsp. bulgaricus A	ZP_00386722.1	NZ_AAGQ01000090	334
55	4127605	Amino acid ABC transporter, permease protein	Enterococcus faecium DO	ZP_00604829.1	NZ_AAAK03000089	500
56	26211	hypothetical protein	Synechocystis sp. PCC 6803	NP_441171.1	NC_000911	397
57	1432651	hypothetical protein	Listeria innocua Clip11262	NP_470182.1	NC_003212	480
58	3176893	glutamine ABC transporter	Listeria monocytogenes str. 1/2a F6854	ZP_00232467.1	NZ_AADQ01000002	480
59	2959597	glutamine ABC transporter	Listeria monocytogenes str. 4b F2365	YP_013467.1	NC_002973	480
60	1429910	hypothetical protein	Listeria monocytogenes EGD-e	NP_464373.1	NC_003210	480
61	3177117	glutamine ABC transporter	Listeria monocytogenes str. 4b H7858	ZP_00229847.1	NZ_AADR01000005	480
62	2322249	possible ligand gated channel (GIC family)	Prochlorococcus marinus str. MIT 9313	NP_894348.1	NC_005071	359
63	4124856	extracellular solute-binding protein, family 3	Paracoccus denitrificans PD1222	ZP_00629025.1	NZ_AAIT01000002	360

64	3859018	COG0834: ABC-type amino acid transport/signal	Lactobacillus casei ATCC 334	ZP_00384469.1	NZ_AAGR01000100	469
65	4138225	extracellular solute-binding protein, family	Chlorobium phaeobacteroides BS1	ZP_00530895.1	NZ_AAIC01000004	416
66	2508200	periplasmic substrate-binding protein/sensor	Geobacter sulfurreducens PCA	NP_953800.1	NC_002939	590
67	1434059	hypothetical protein	Listeria innocua Clip11262	NP_471683.1	NC_003212	486
68	3942849	Q4JLI8) Lr1198	Lactobacillus reuteri			487
69	3169121	amino acid ABC transporter, permease protein	Listeria monocytogenes str. 4b H7858	ZP_00232127.1	NZ_AADR01000117	395
70	4405283	extracellular solute-binding protein, family	Pelobacter propionicus DSM 2379	ZP_00676317.1	NZ_AAJH01000002	780
71	2959738	amino acid ABC transporter, permease protein	Listeria monocytogenes str. 4b F2365	YP_014872.1	NC_002973	486
72	1431258	hypothetical protein	Listeria monocytogenes EGD-e	NP_465774.1	NC_003210	486
73	3050602	putative extracellular glutamine-binding	Staphylococcus aureus subsp. aureus MRSA	YP_041324.1	NC_002952	485
74	2679109	GlnQ	Mycobacterium avium subsp. paratuberculos	NP_958986.1	NC_002944	579
75	1807804	hypothetical protein	Staphylococcus aureus subsp. aureus MW2	NP_646616.1	NC_003923	485
76	740171	hypothetical protein	Staphylococcus aureus subsp. aureus N315	NP_374965.1	NC_002745	441
77	2139439	amino acid ABC transporter, amino	Enterococcus faecalis V583	NP_814632.1	NC_004668	487
78	4510424	Amino acid ABC transporter, permease protein	Pelodictyon luteolum DSM 273	ABB23899.1	CP000096	508
79	3967309	hypothetical protein	Staphylococcus haemolyticus JCSC1435	YP_253019.1	NC_007168	484
80	2051188	glutamine-binding periplasmic protein	Staphylococcus epidermidis ATCC 12228	NP_765096.1	NC_004461	485
81	3625978	amino acid ABC transporter, permease/amino	Staphylococcus epidermidis RP62A	YP_188964.1	NC_002976	485
82	3178530	COG0834: ABC-type amino acid transport/signal	Pediococcus pentosaceus ATCC 25745	ZP_00323409.1	NZ_AAEV01000004	480
83	2675945	COG0834: ABC-type amino acid transport/signal	Leuconostoc mesenteroides subsp. mesentero	ZP_00064352.1	NZ_AABH02000075	485
84	4148754	IMP dehydrogenase/GMP reductase:Bacterial	Desulfuromonas acetoxidans DSM 684	ZP_00550581.1	NZ_AAEW01000009	616
85	4521215	Putative amino acid ABC transporter, substrate	Lactobacillus sakei subsp. sakei 23K	CAI55358.1	CR936503	487
86	4405380	PAS	Pelobacter propionicus DSM 2379	ZP_00678091.1	NZ_AAJH01000010	###
87	2028708	sensory box protein	Pseudomonas putida KT2440	NP_742553.1	NC_002947	###
88	4284112	truncated ABC-type amino acid transport system	Staphylococcus saprophyticus subsp. saprop	YP_301027.1	NC_007350	398
89	4397468	extracellular solute-binding protein, family 3	Syntrophobacter fumaroxidans MPOB	ZP_00665141.1	NZ_AAJF01000004	445
90	2495900	ABC-type amino acid transport system, permease	Onion yellows phytoplasma OY-M	NP_950330.1	NC_005303	601
91	2742825	ABC transporter permease component	Lactobacillus johnsonii NCC 533	NP_964641.1	NC_005362	497
92	4400497	PAS	Thiomicrospira denitrificans ATCC 33889	ZP_00650326.1	NZ_AAJA01000001	633
93	4162782	PAS	Magnetococcus sp. MC-1	ZP_00606263.1	NZ_AAAN03000005	###
94	4119172	K channel, pore region	Chlorobium phaeobacteroides BS1	ZP_00533070.1	NZ_AAIC01000173	358
95	1972106	putative amino acid ABC transporter, permease	Streptococcus mutans UA159	NP_720706.1	NC_004350	517

96	2688998	COG0834: ABC-type amino acid transport/signal	Lactobacillus gasseri	ZP_00045971.1	NZ_AAAO02000001	501
97	793755	amino acid ABC transporter, amino acid-binding	Streptococcus pneumoniae TIGR4	NP_344974.1	NC_003028	521
98	4149446	COG0834: ABC-type amino acid transport/signal	Streptococcus pneumoniae TIGR4	ZP_00402876.1	NZ_AAGY01000123	507
99	4422730	(Q3K3N6) Amino acid ABC transporter, amino acid-binding/permease protein, His/Glu/Gln/Arg/opine family	Streptococcus agalactiae A909			516
100	1919160	hypothetical protein	Streptococcus agalactiae NEM316	NP_734602.1	NC_004368	516
