# Supplementary figures and images for: Domain-Based Identification and Analysis of Glutamate Receptor Ion Channels and Their Relatives in Prokaryotes
Source: PLoS One. 2010 Oct 6;5(10):e12827. doi: 10.1371/journal.pone.0012827 (PMC2950845; doi:10.1371/journal.pone.0012827)

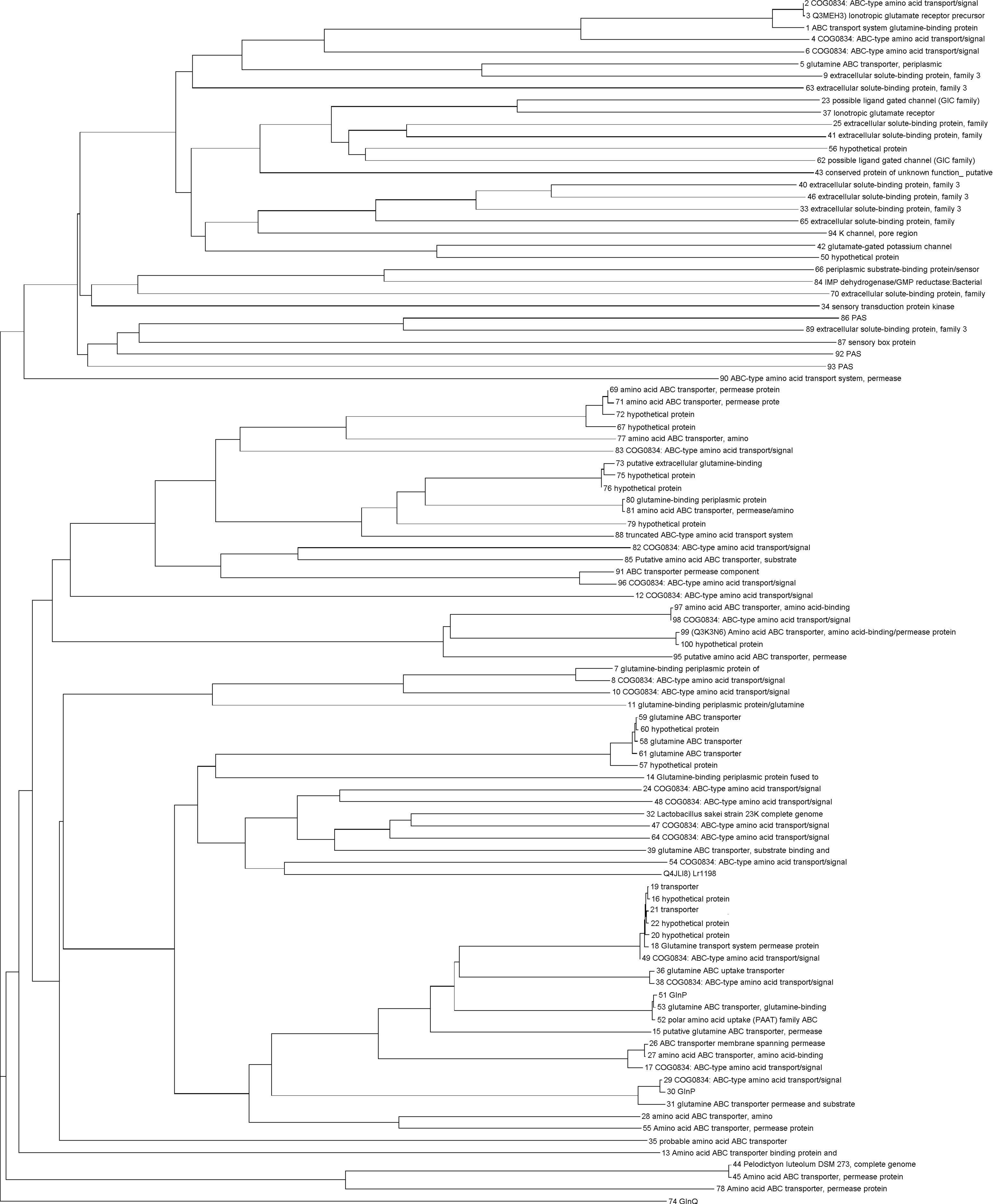

Supplement: Data S2 — Phylogenetic tree for 100 sequences included in this analysis. (0.78 MB PDF) [file pone.0012827.s002.pdf]

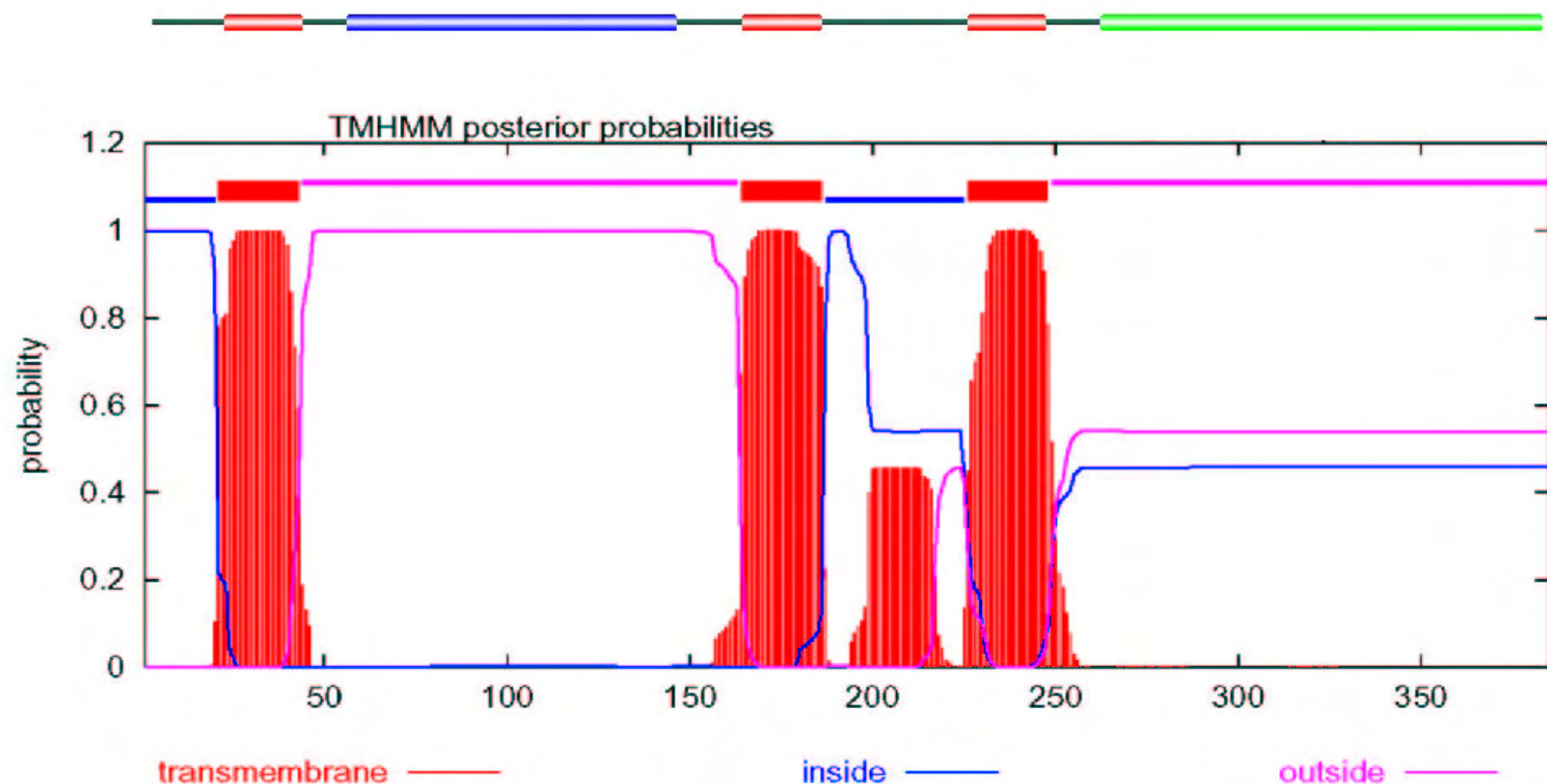

01

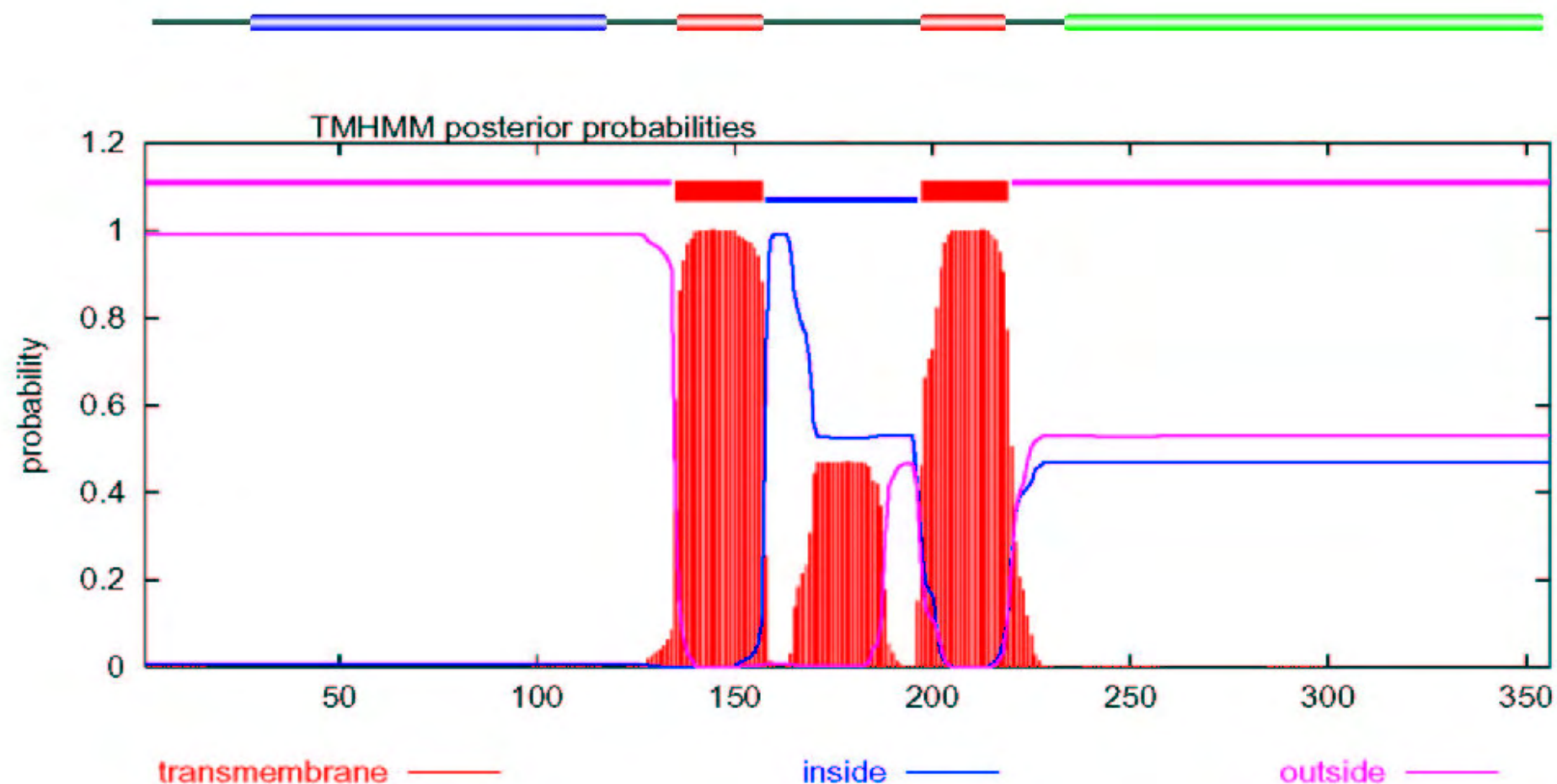

02

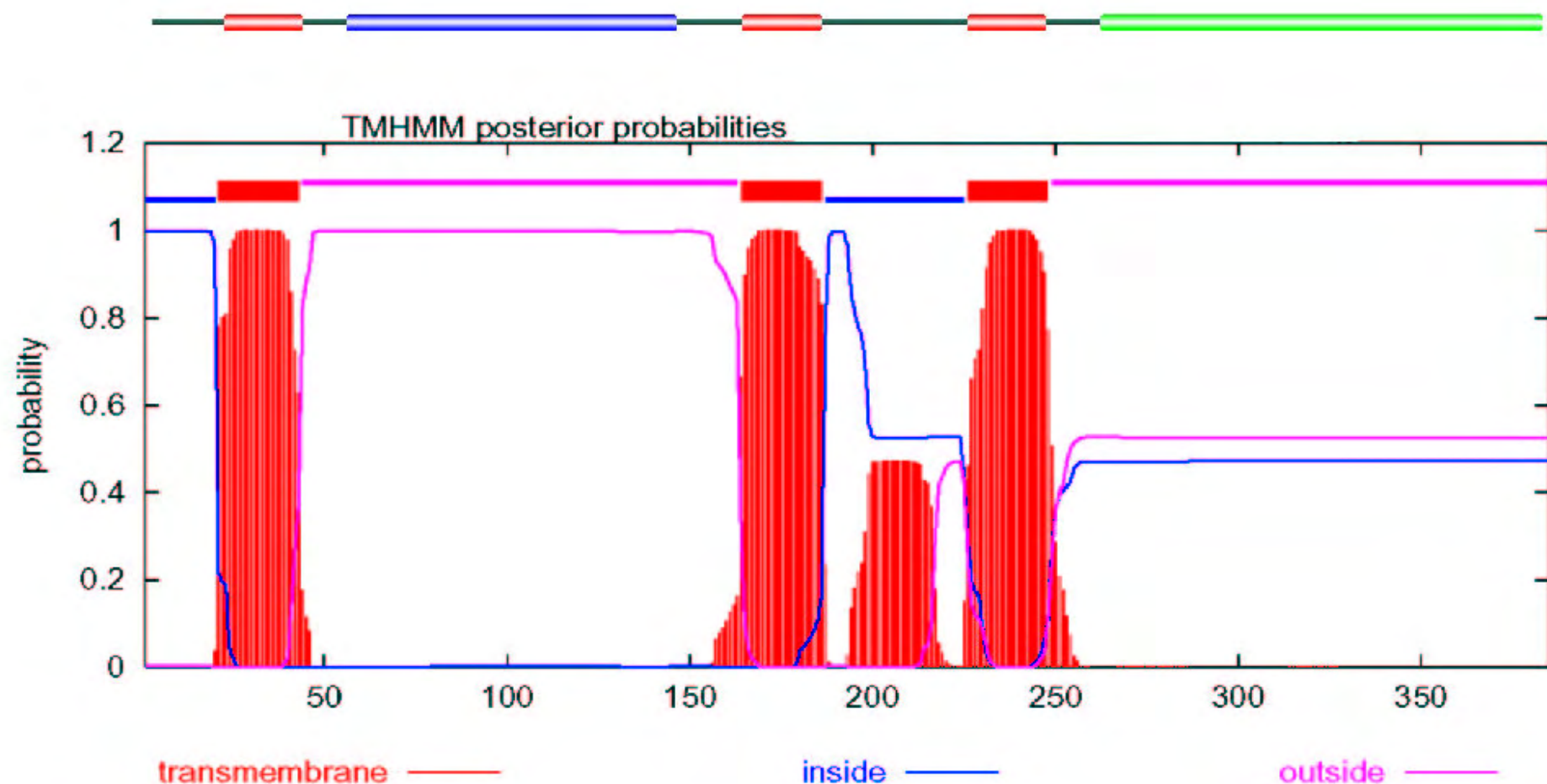

03

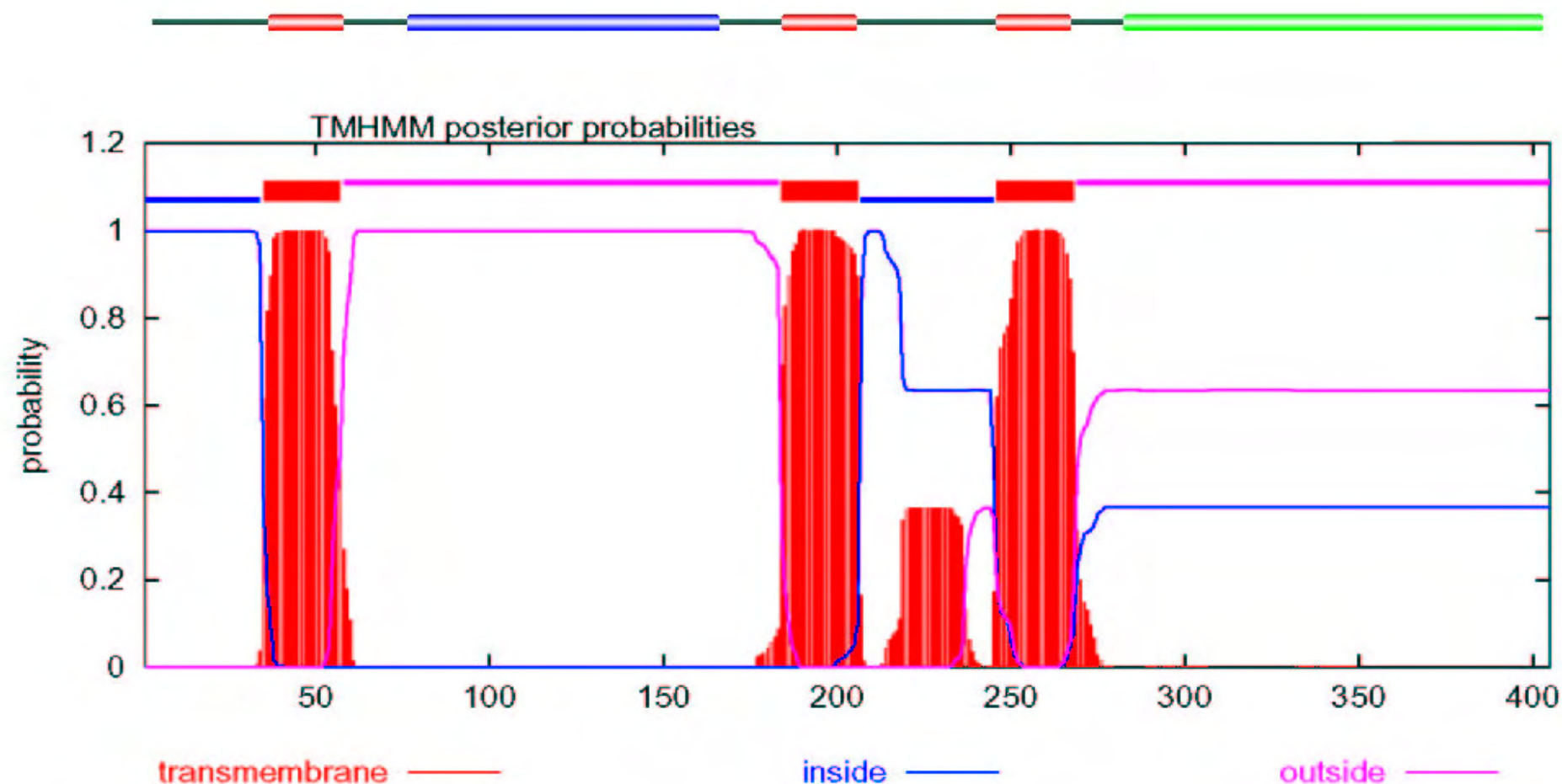

04

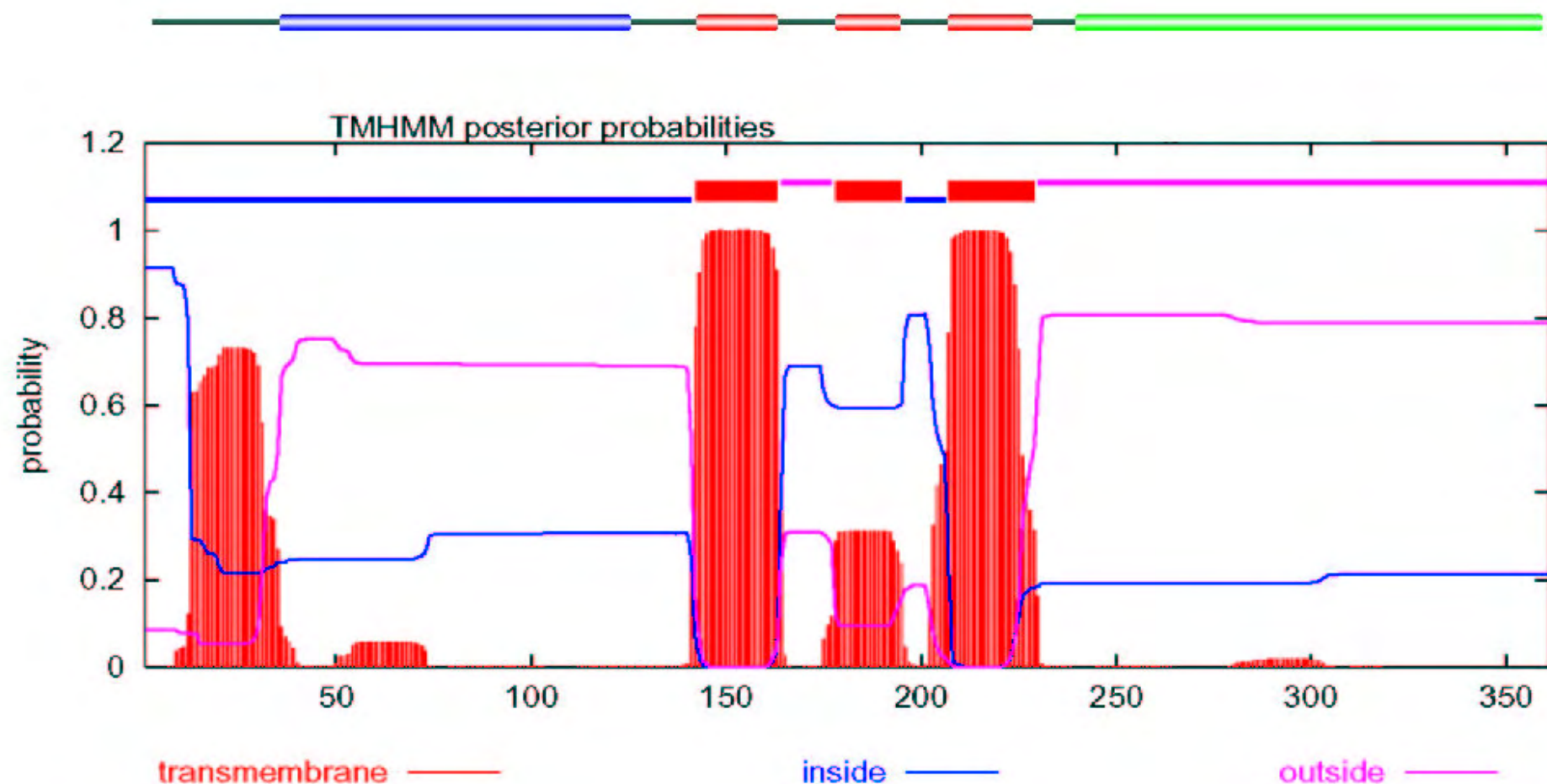

05

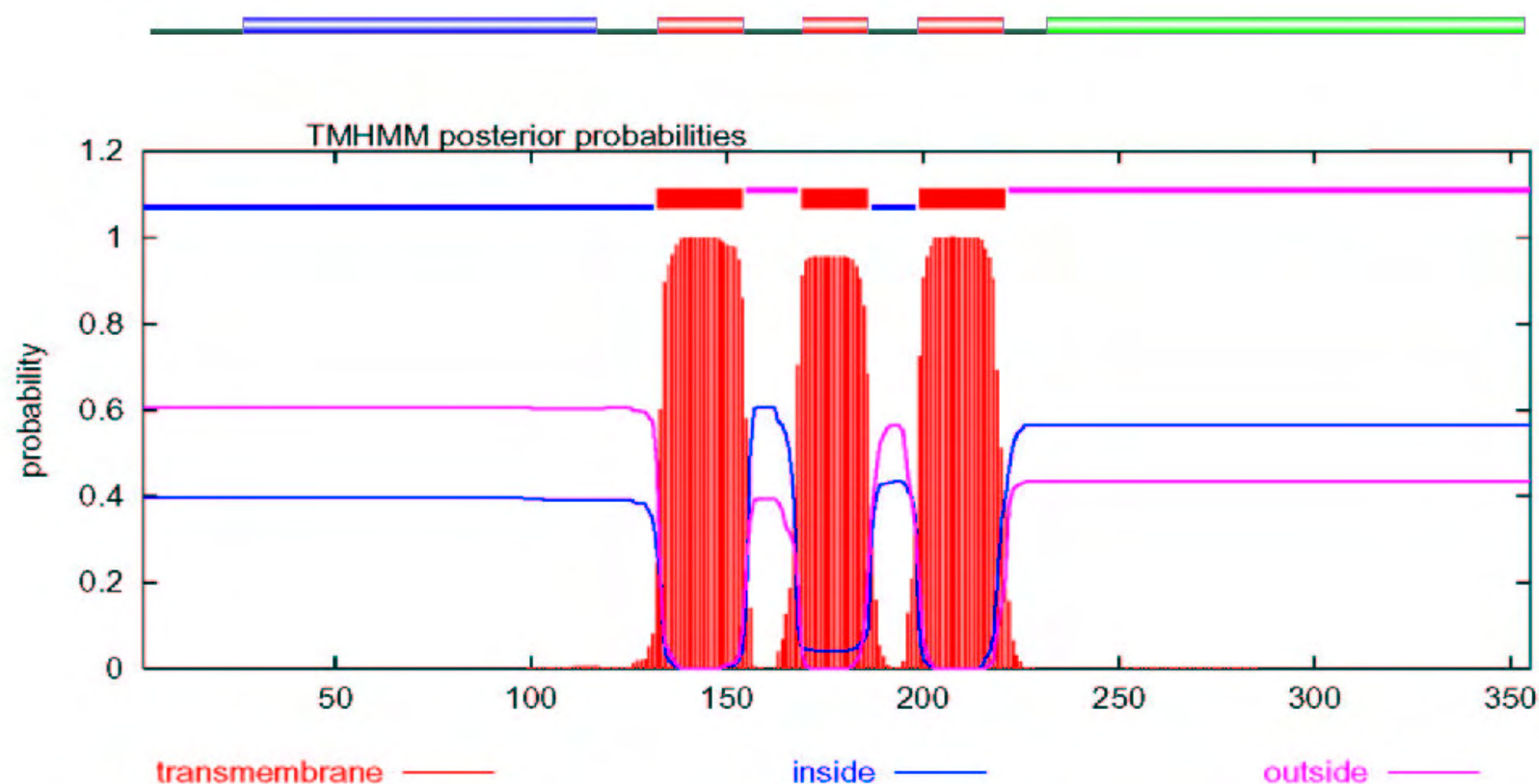

06

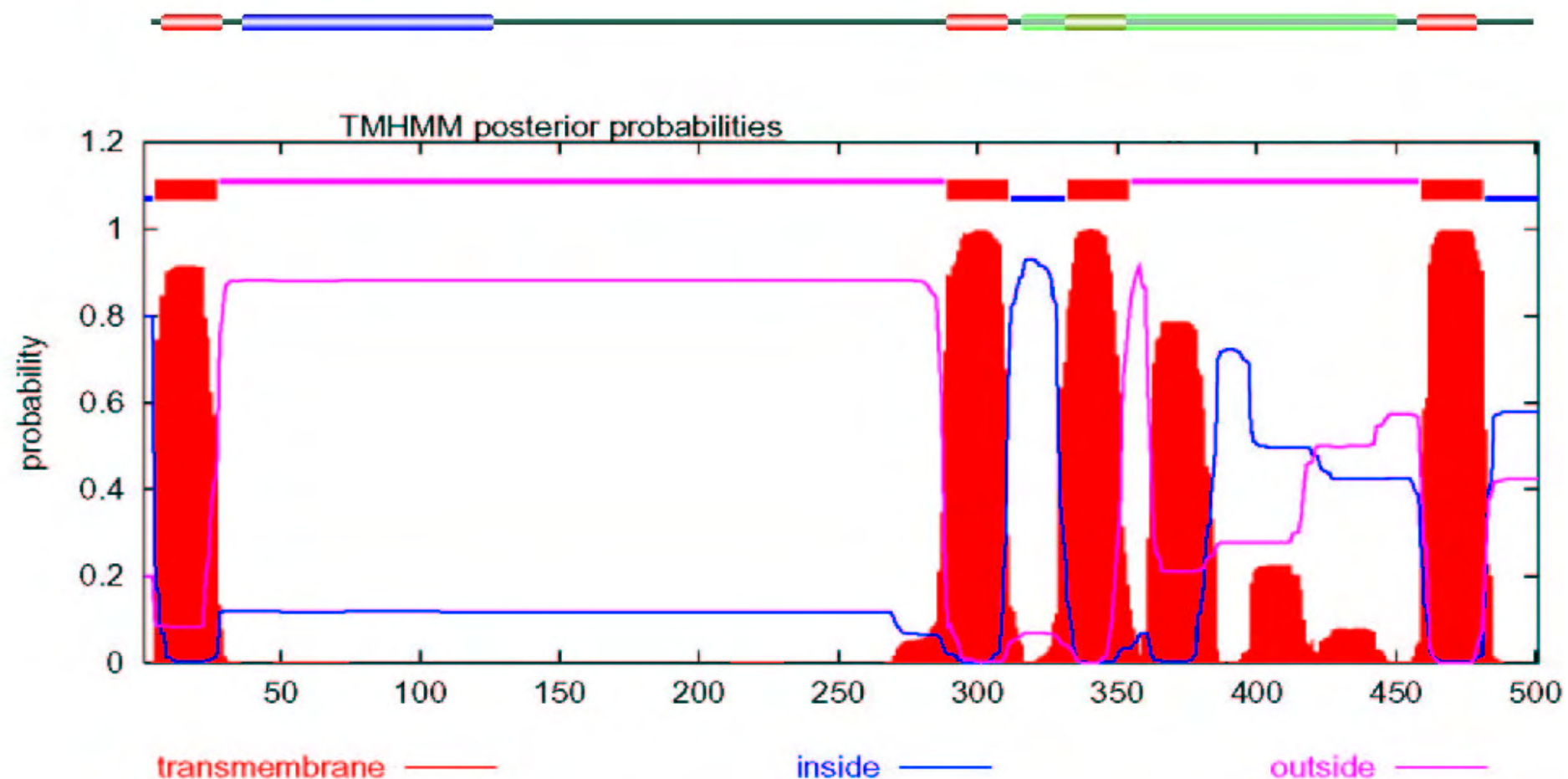

07

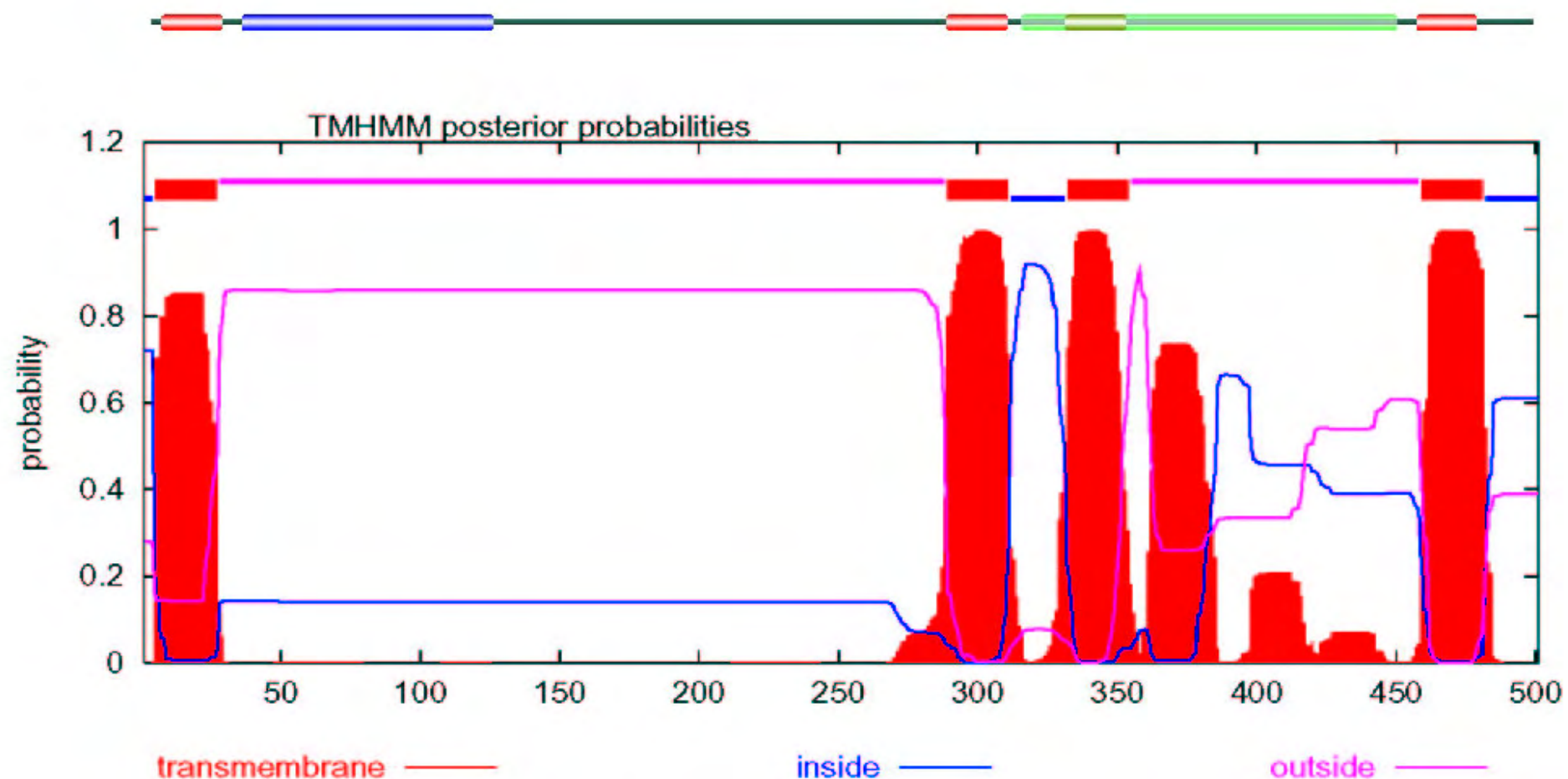

08

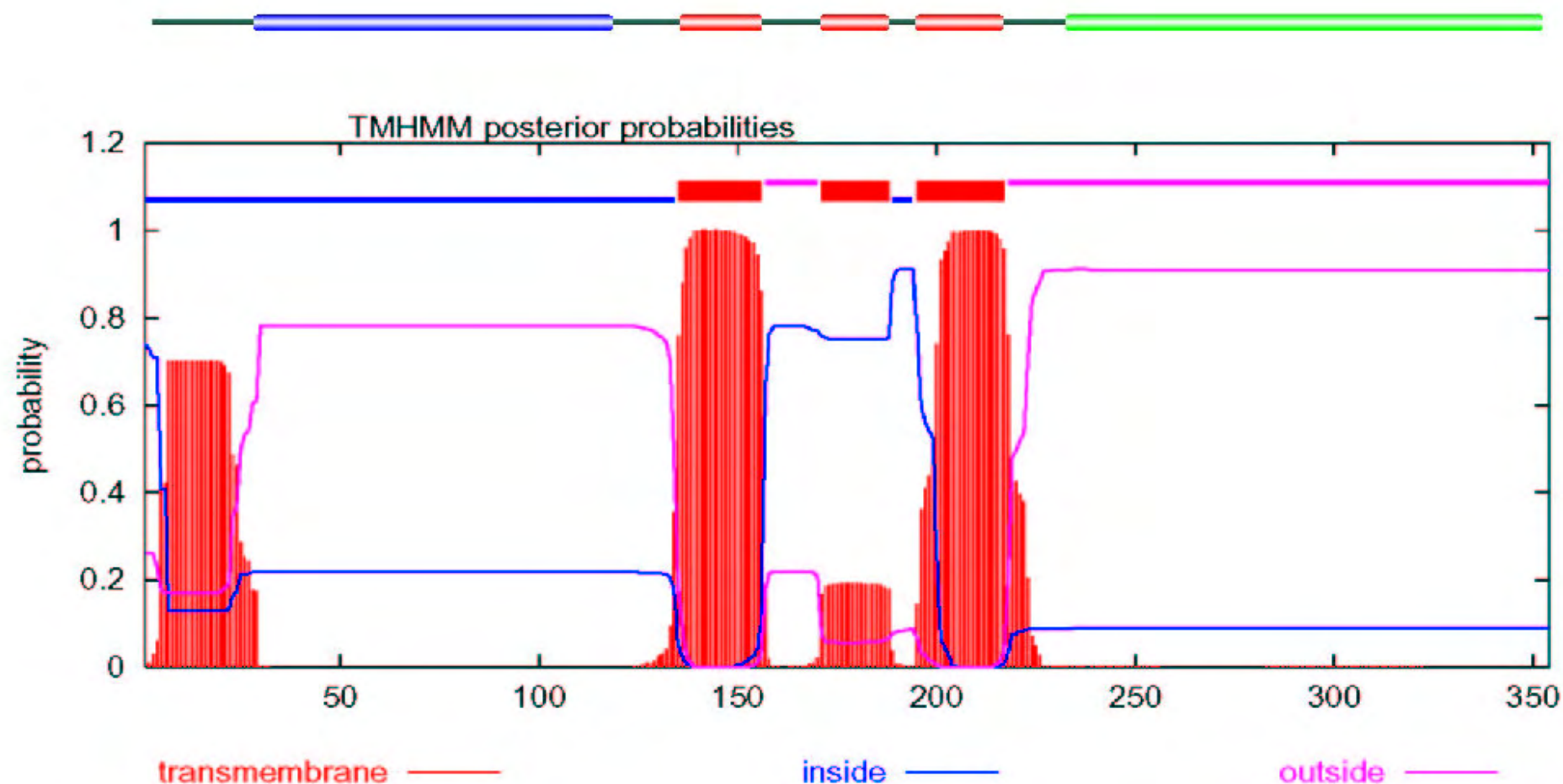

09

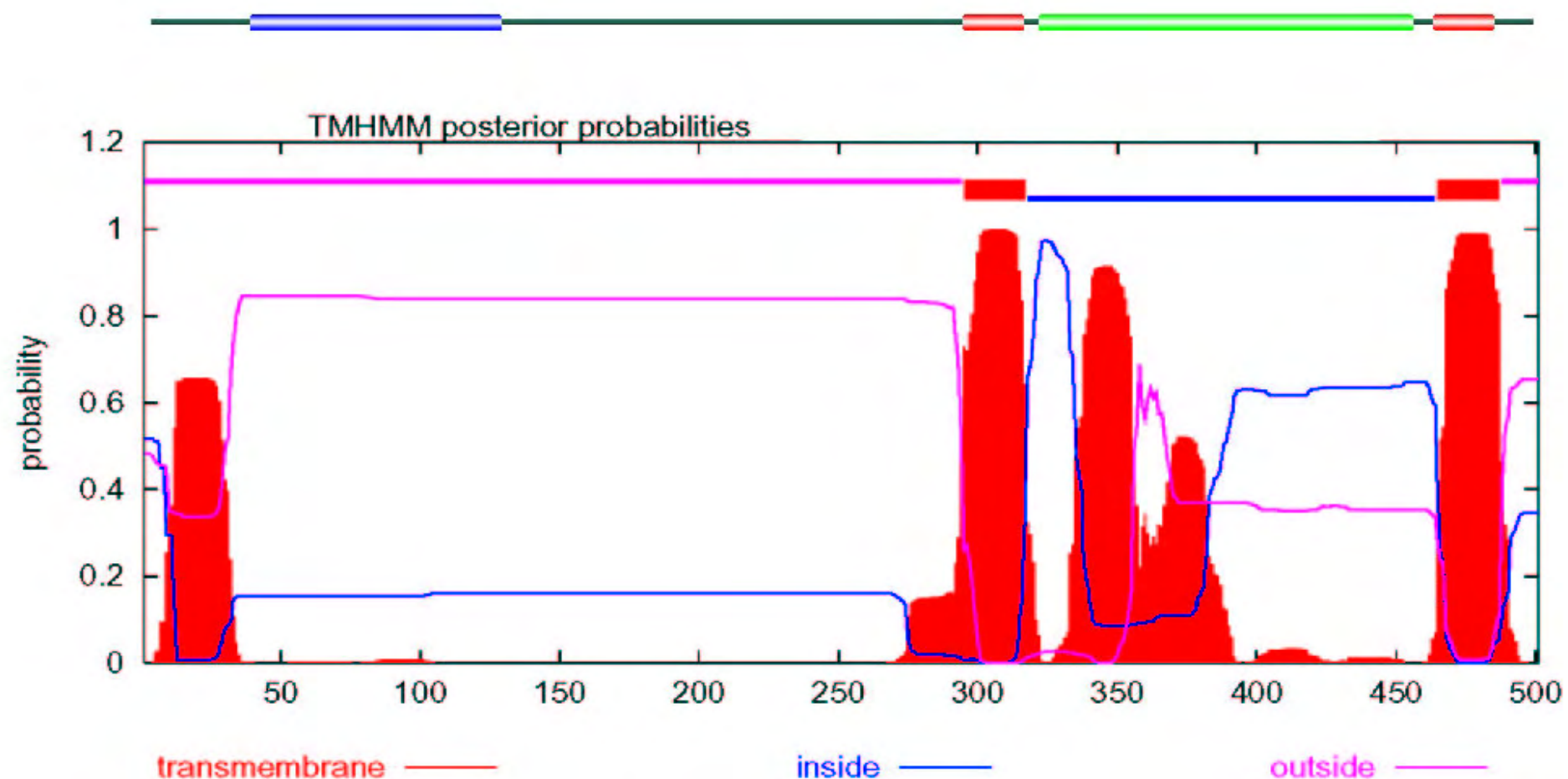

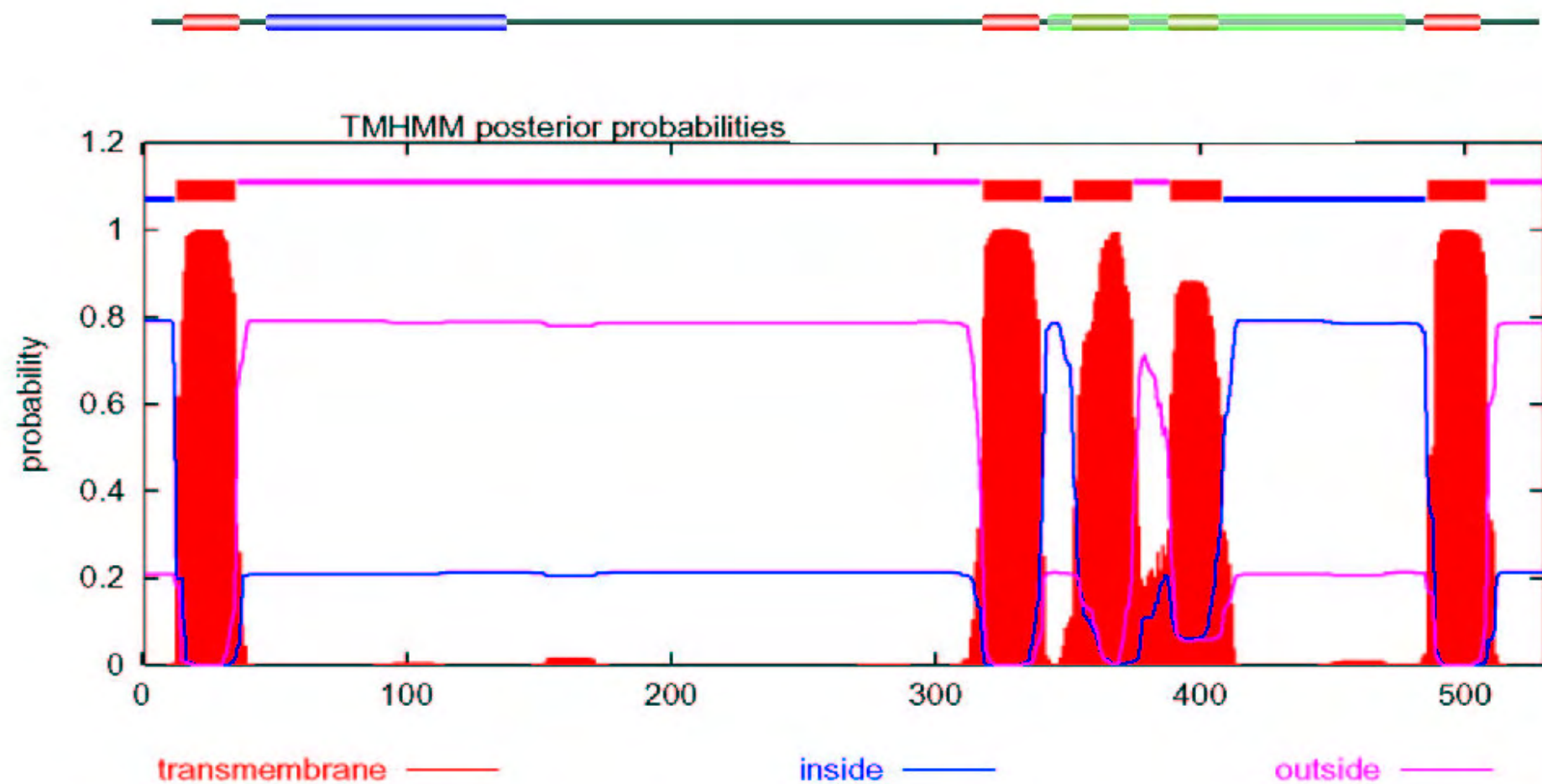

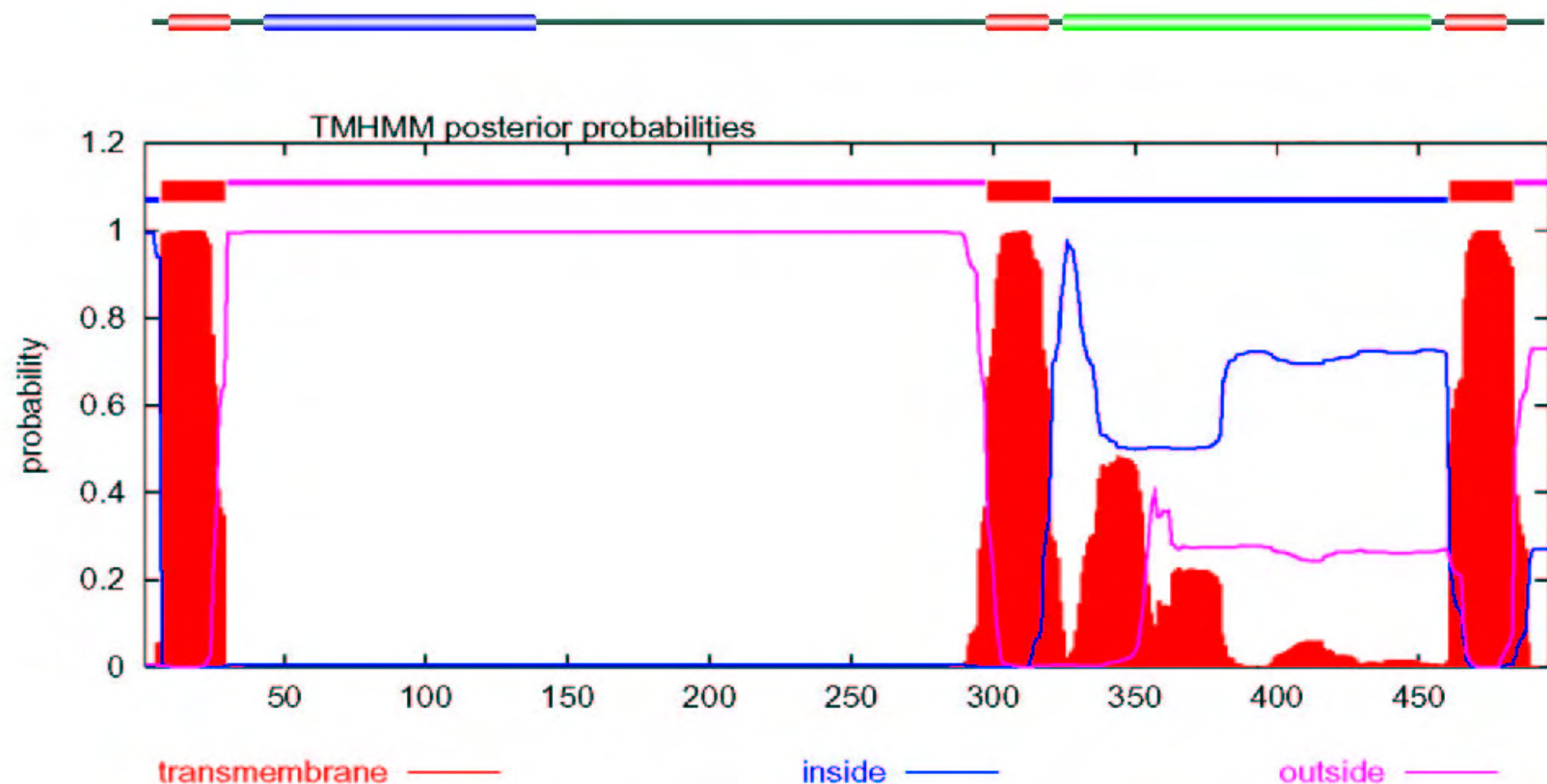

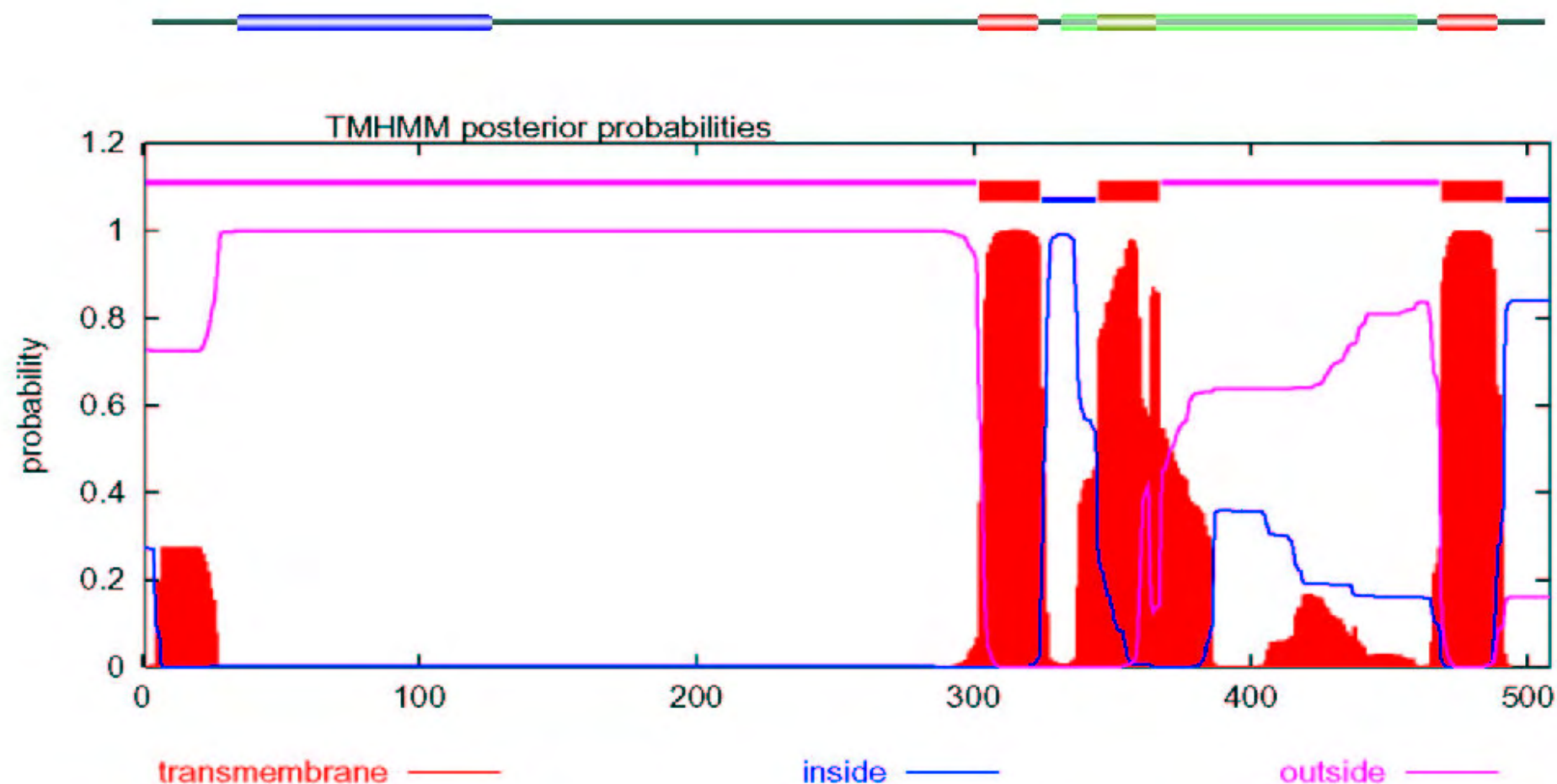

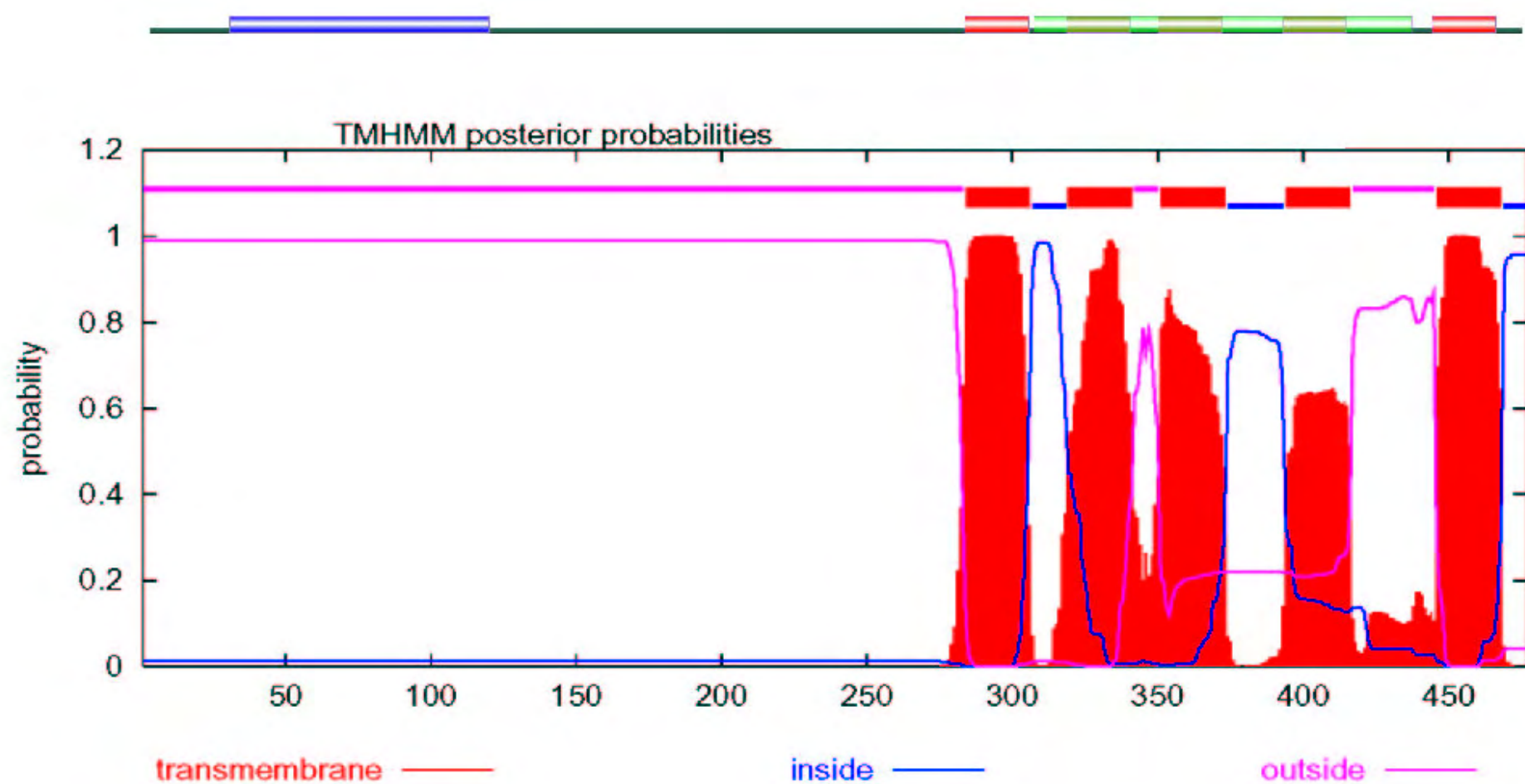

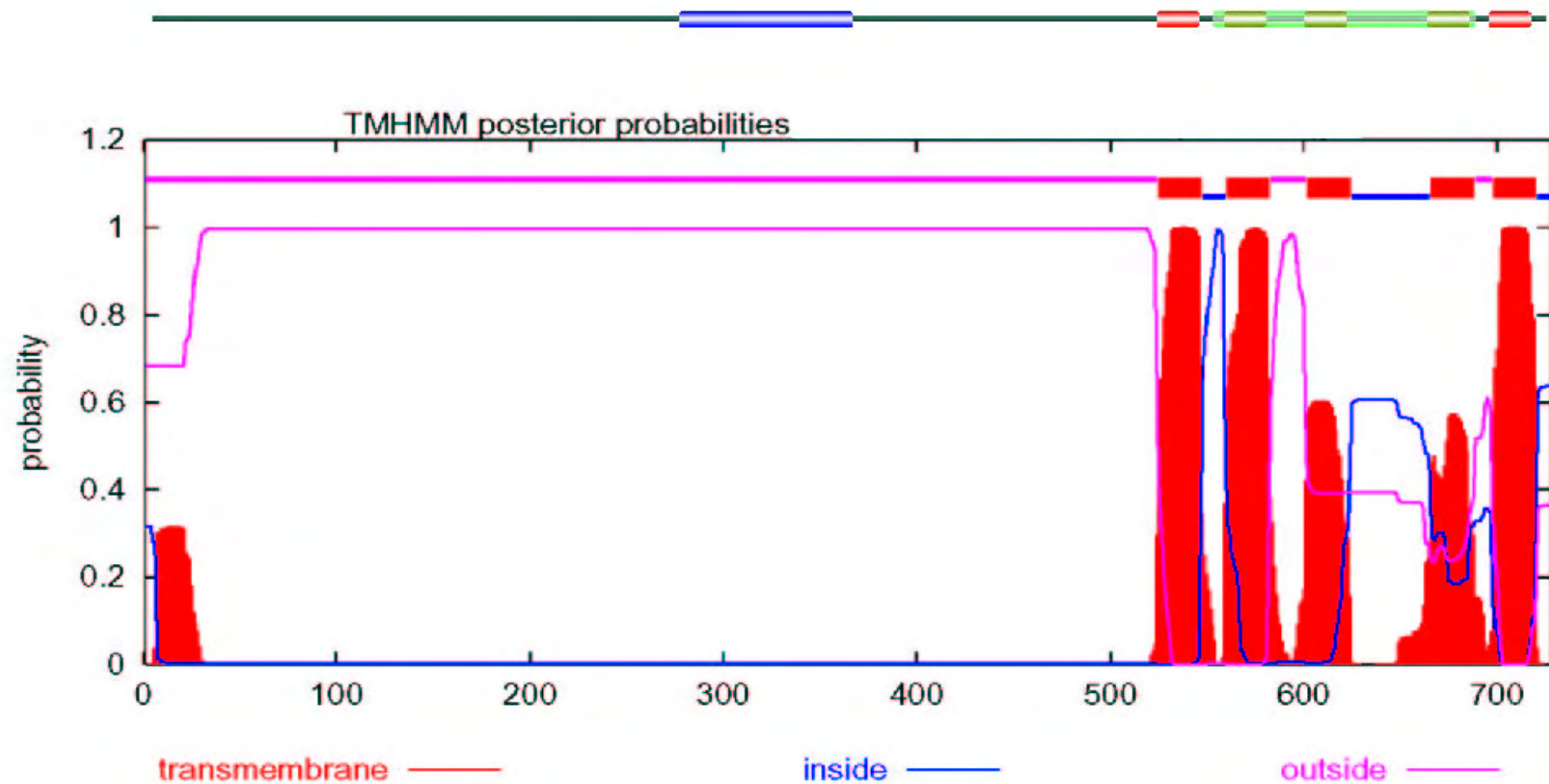

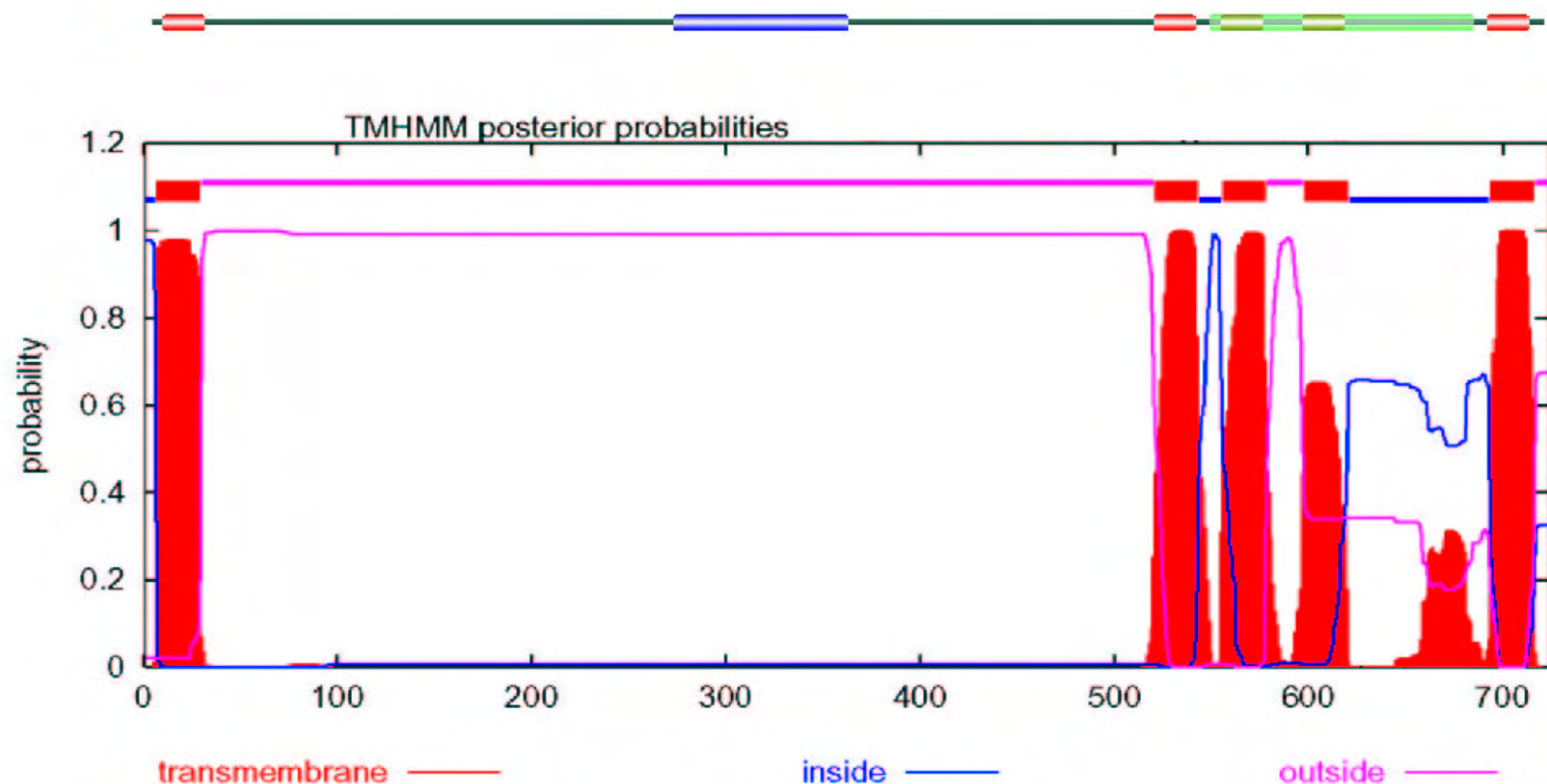

16

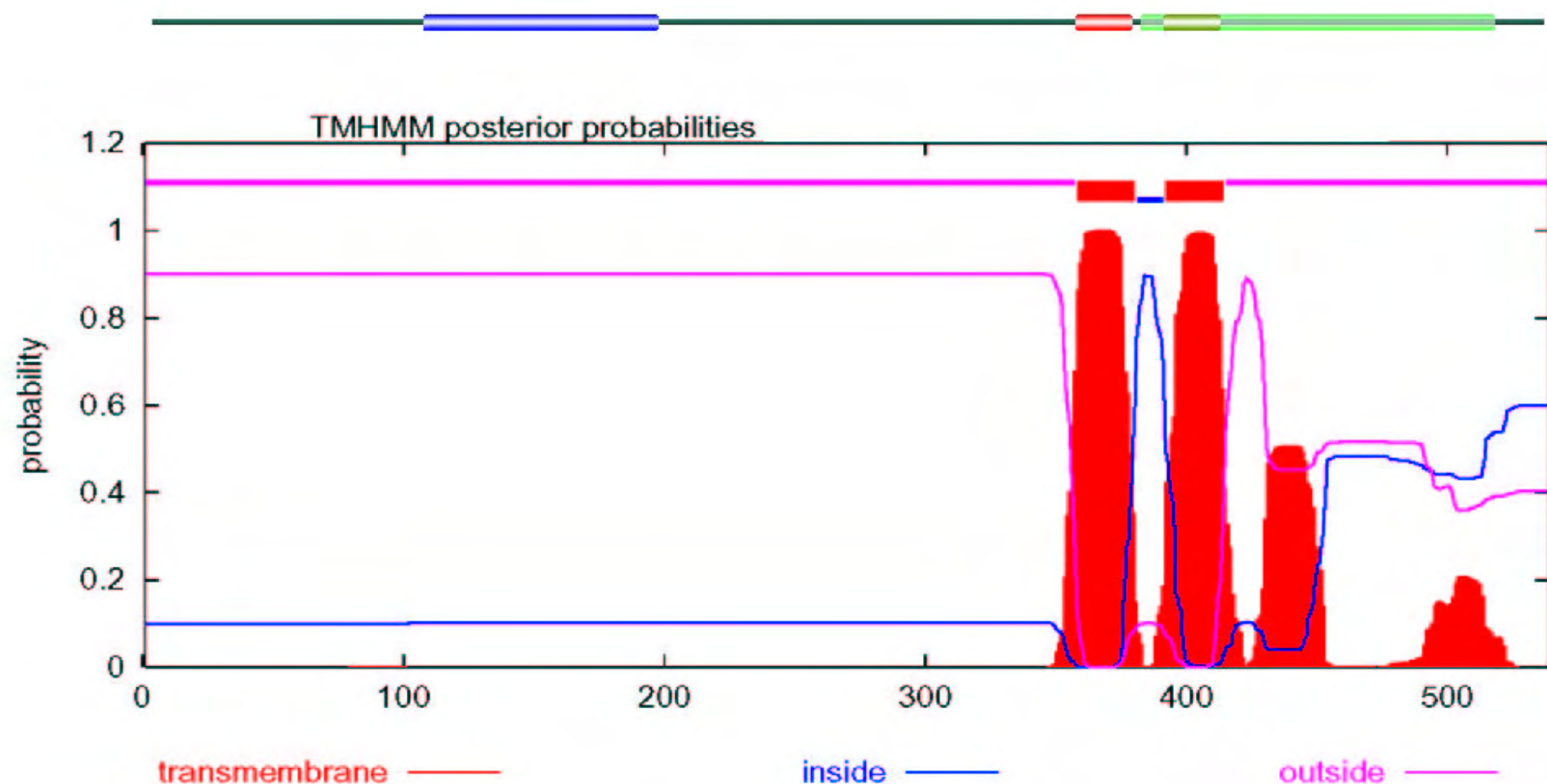

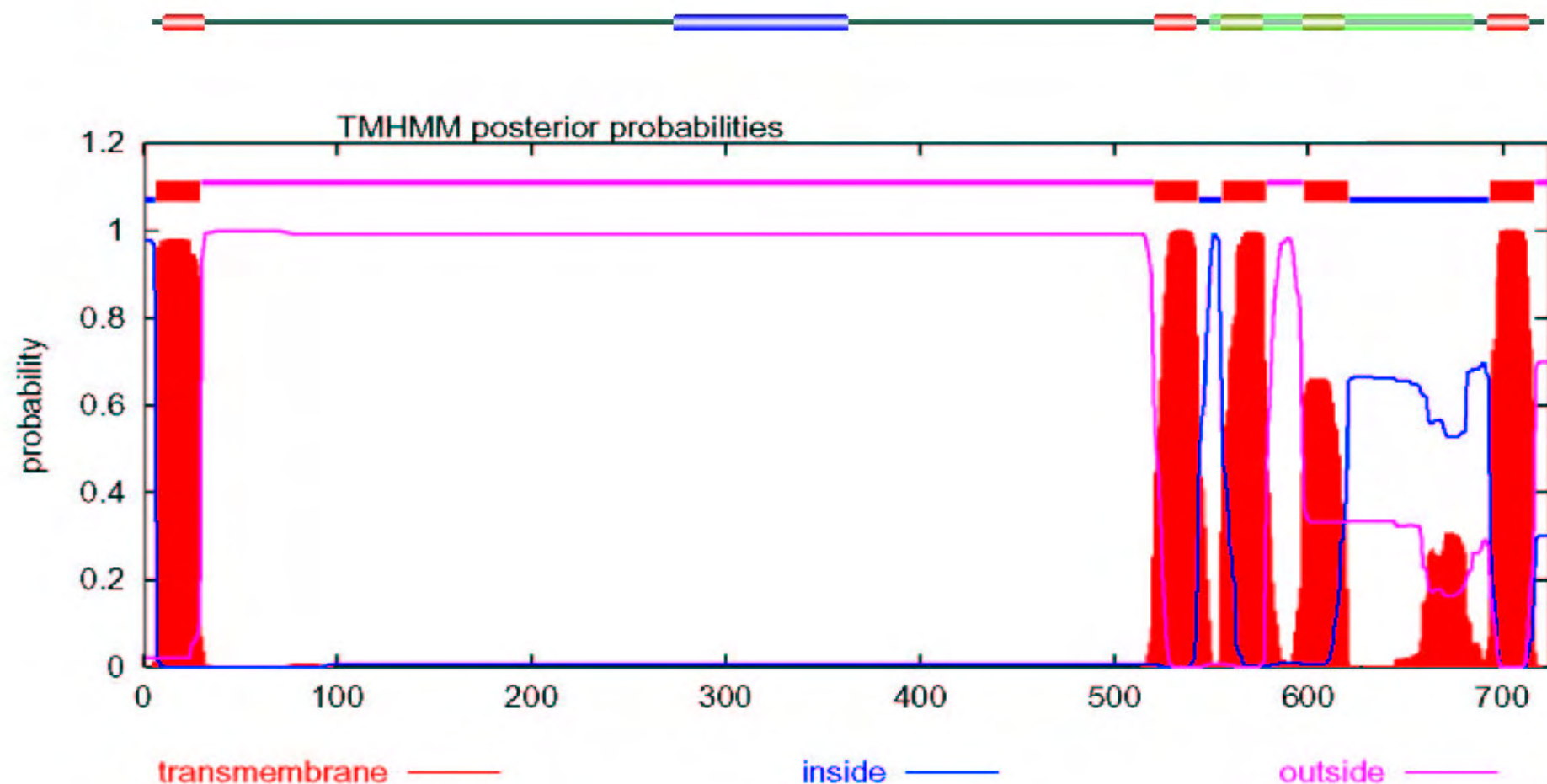

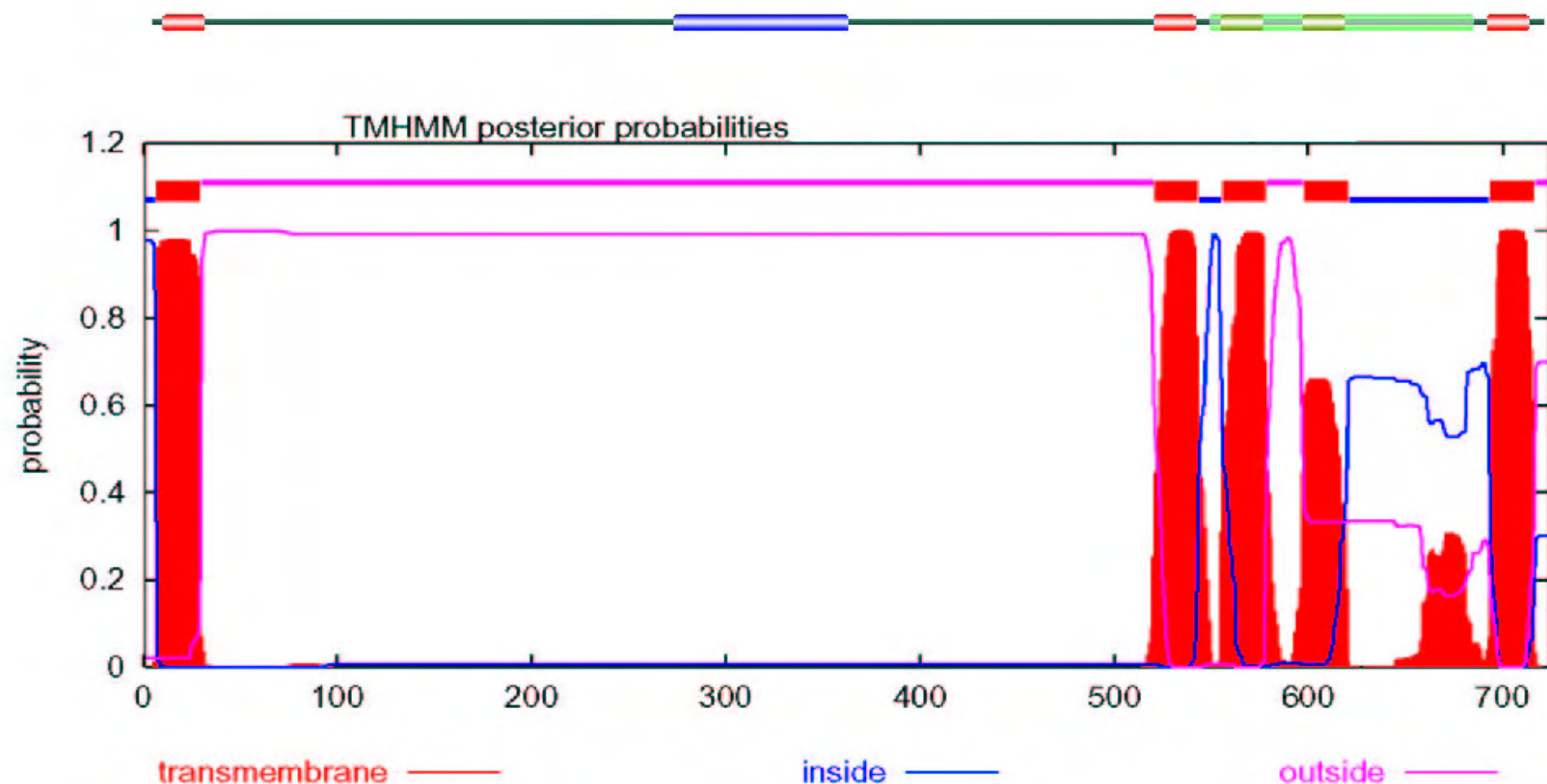

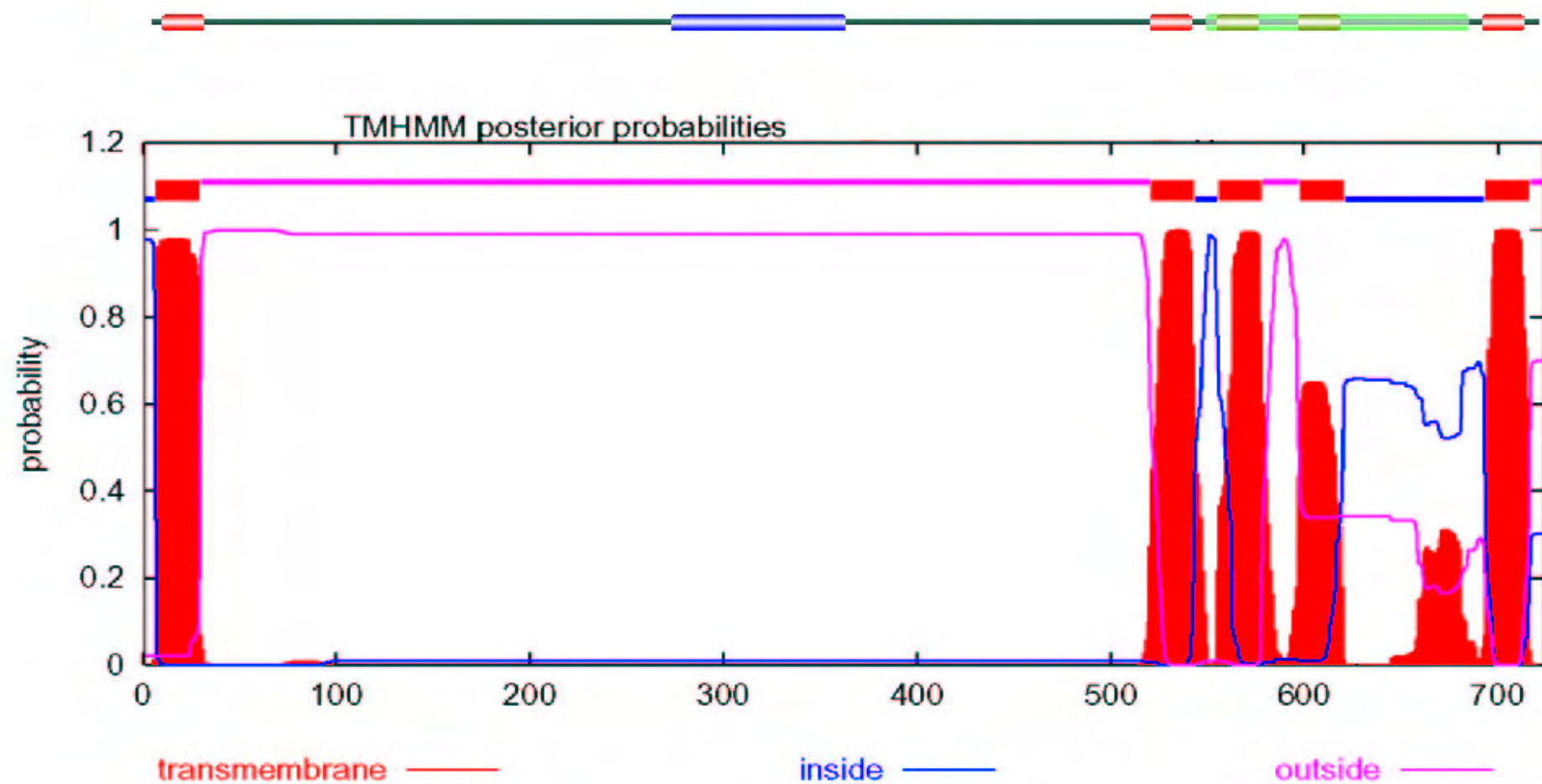

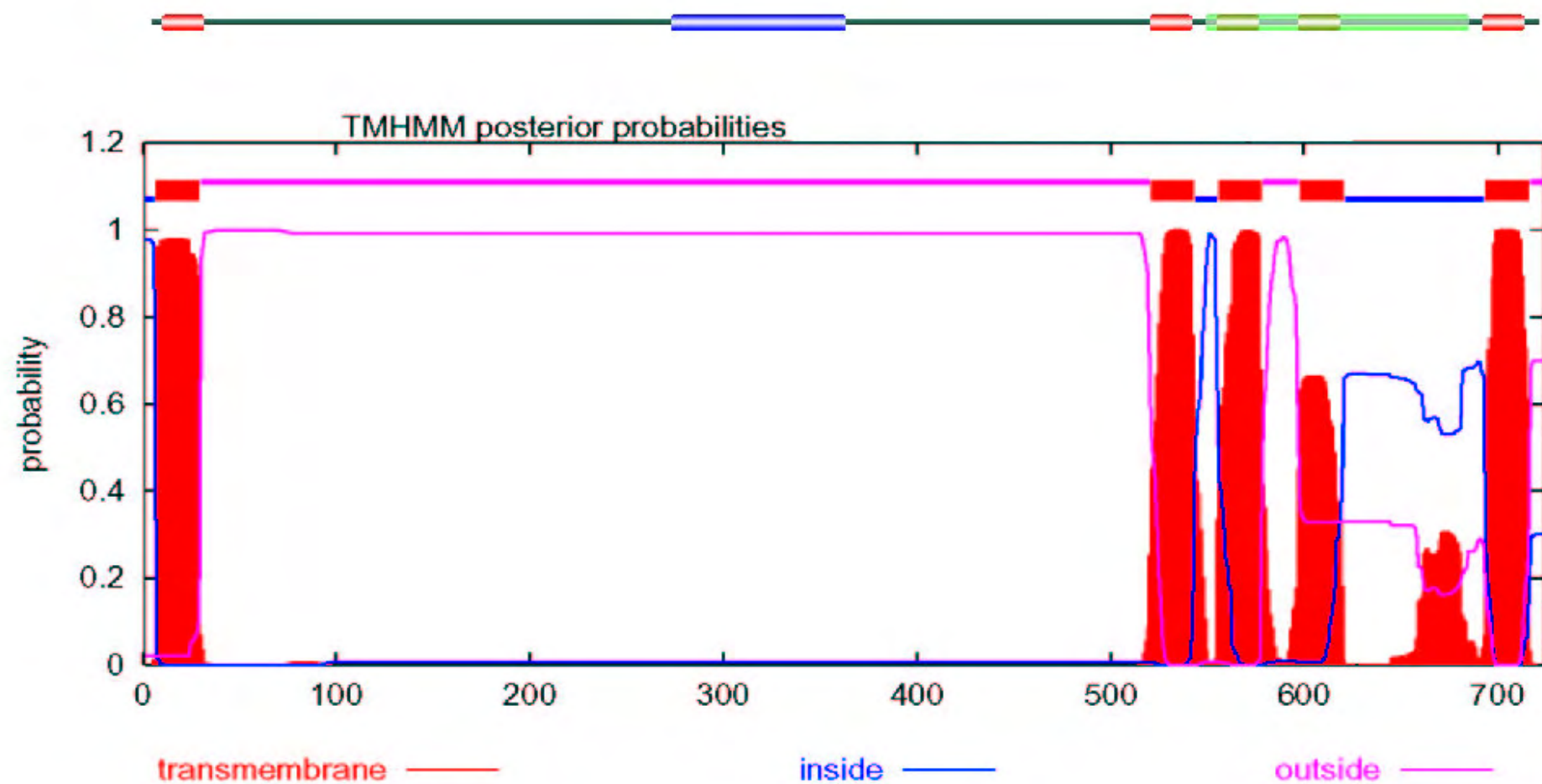

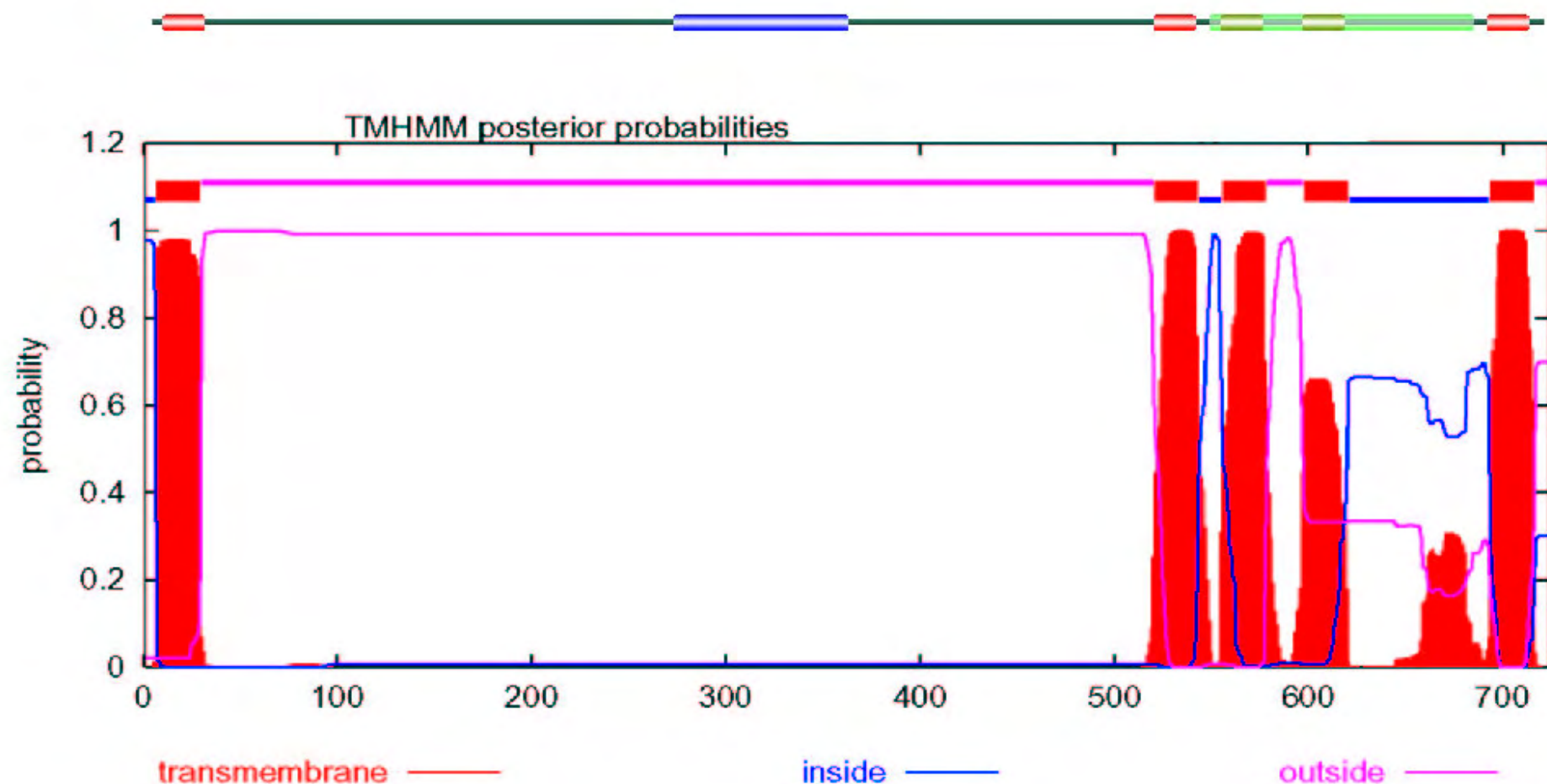

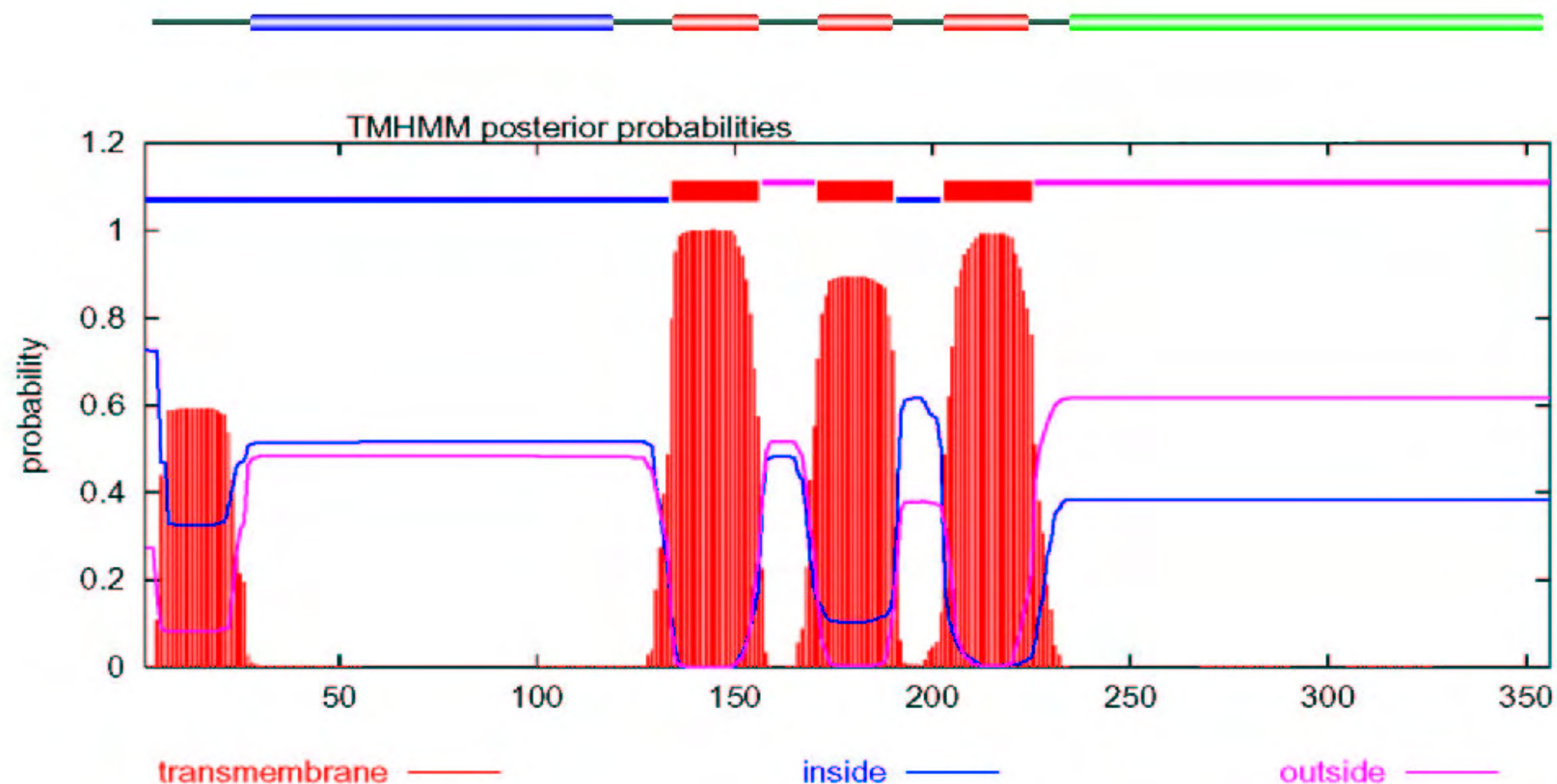

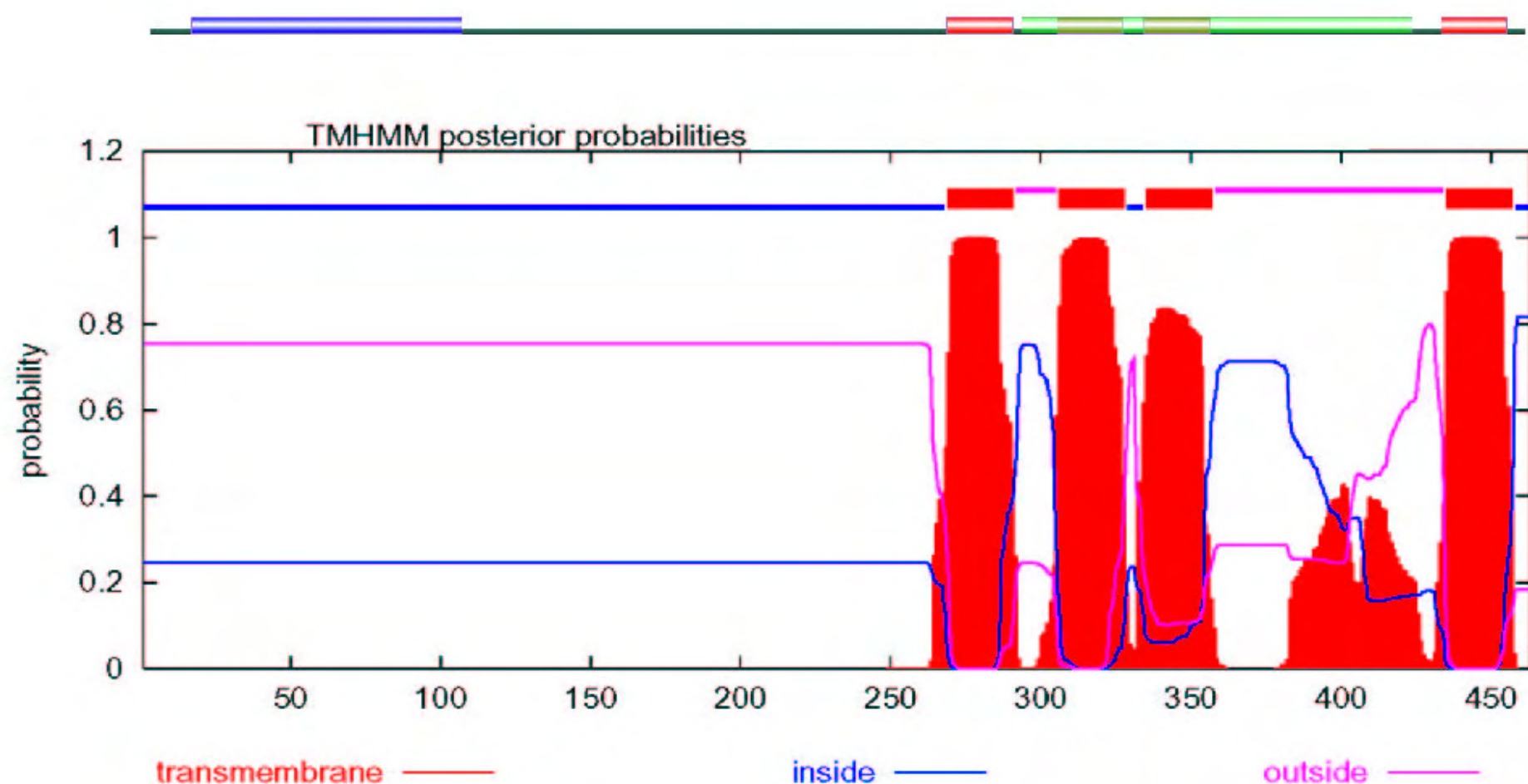

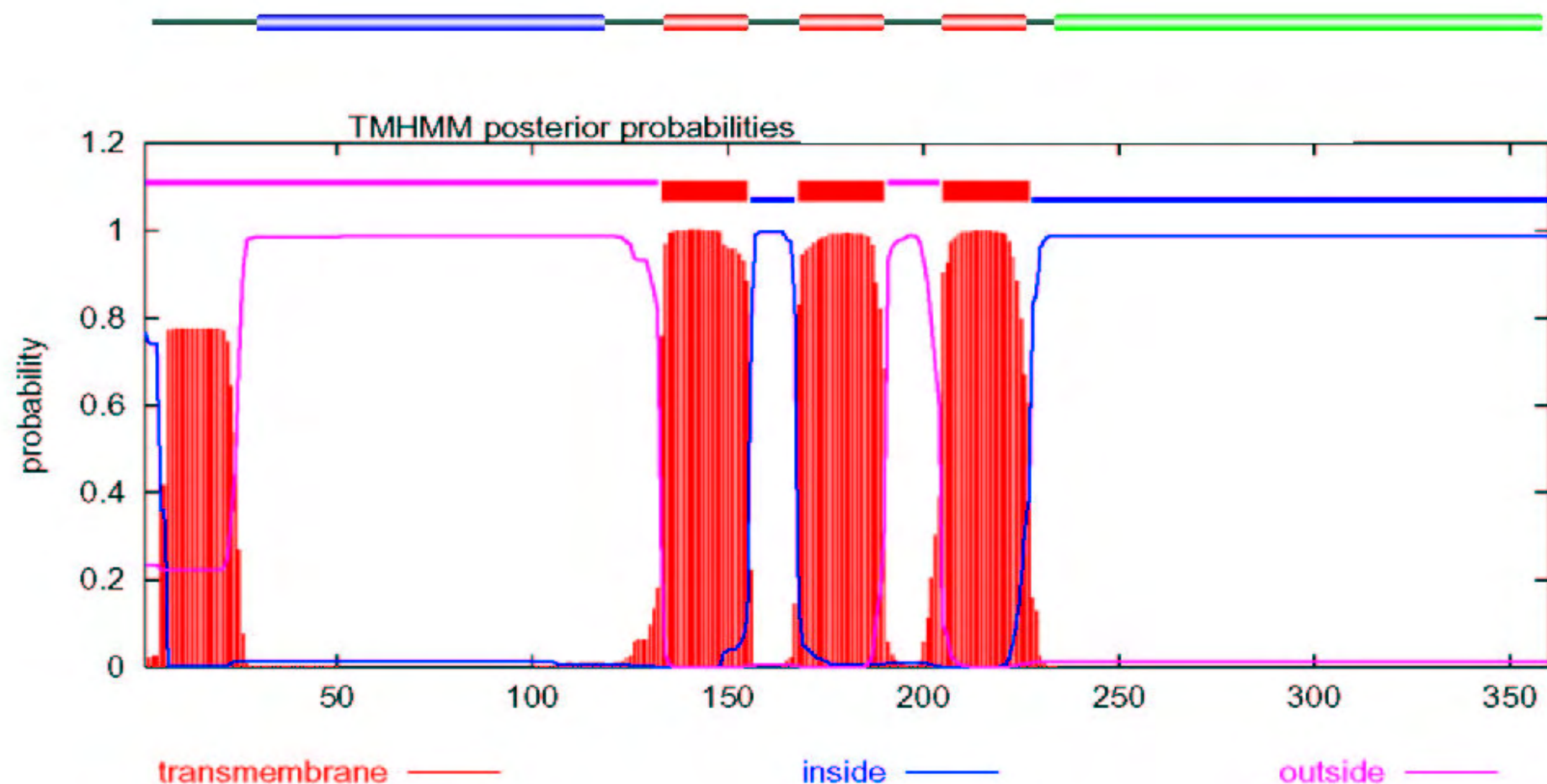

25

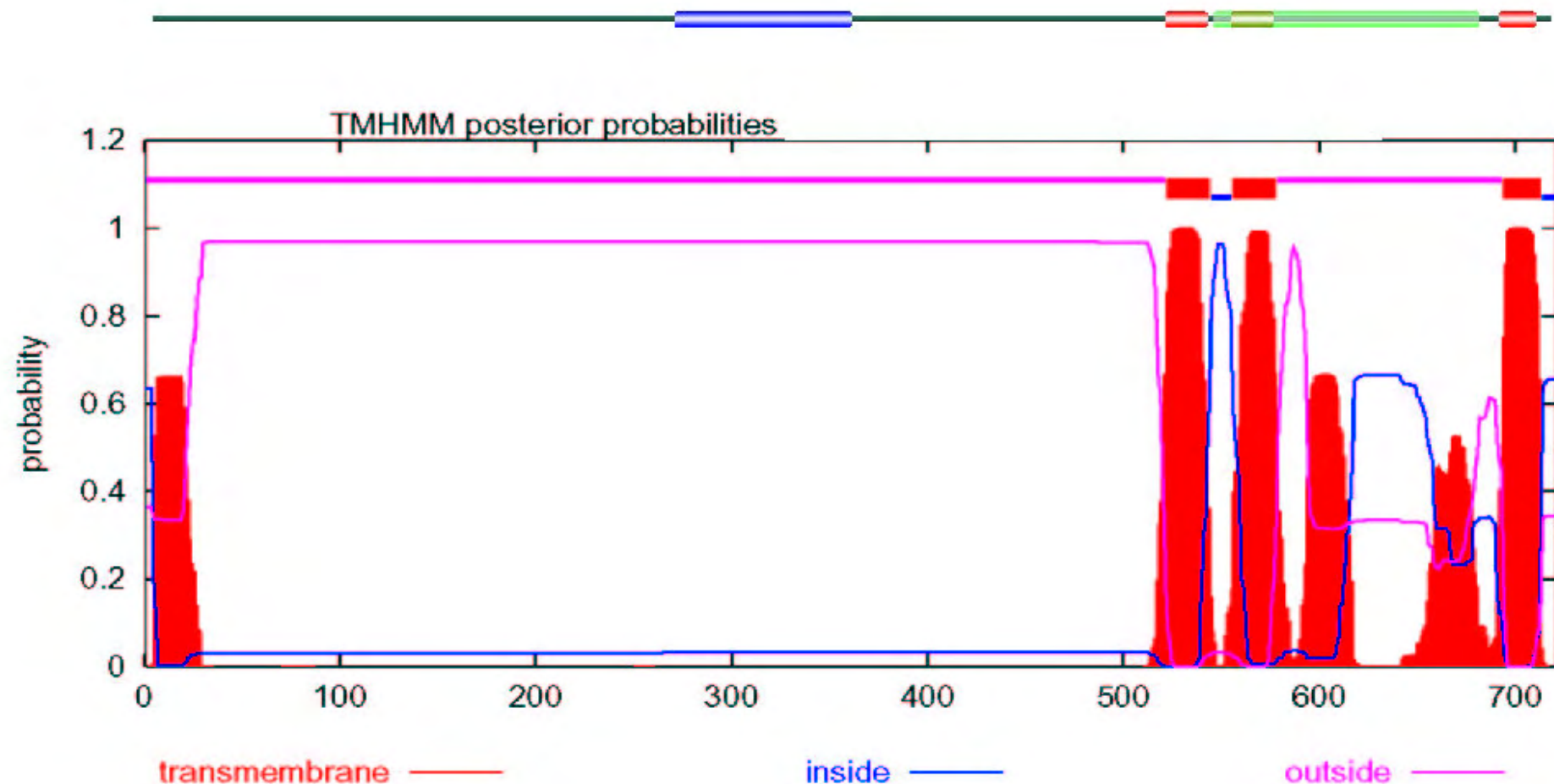

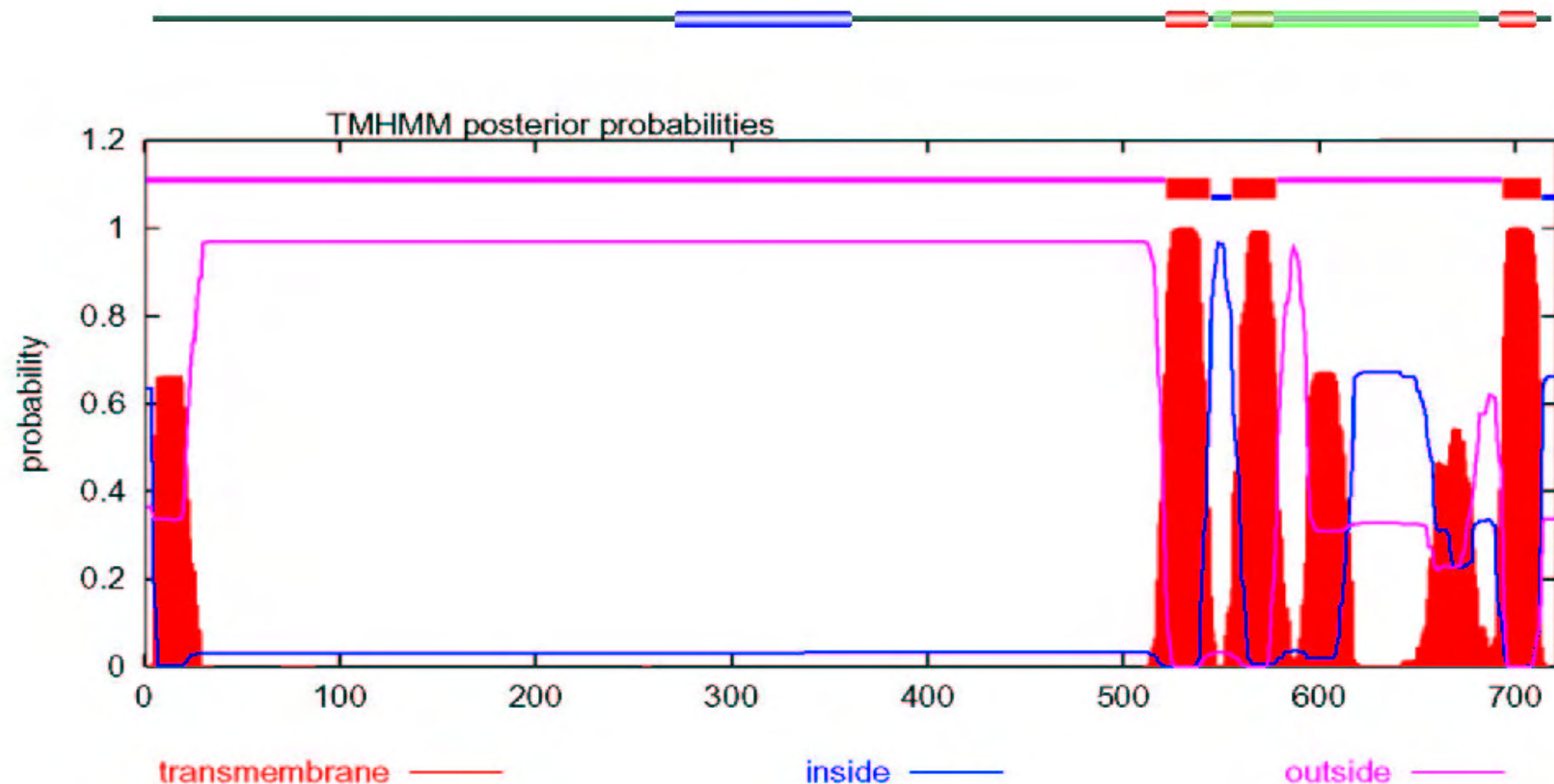

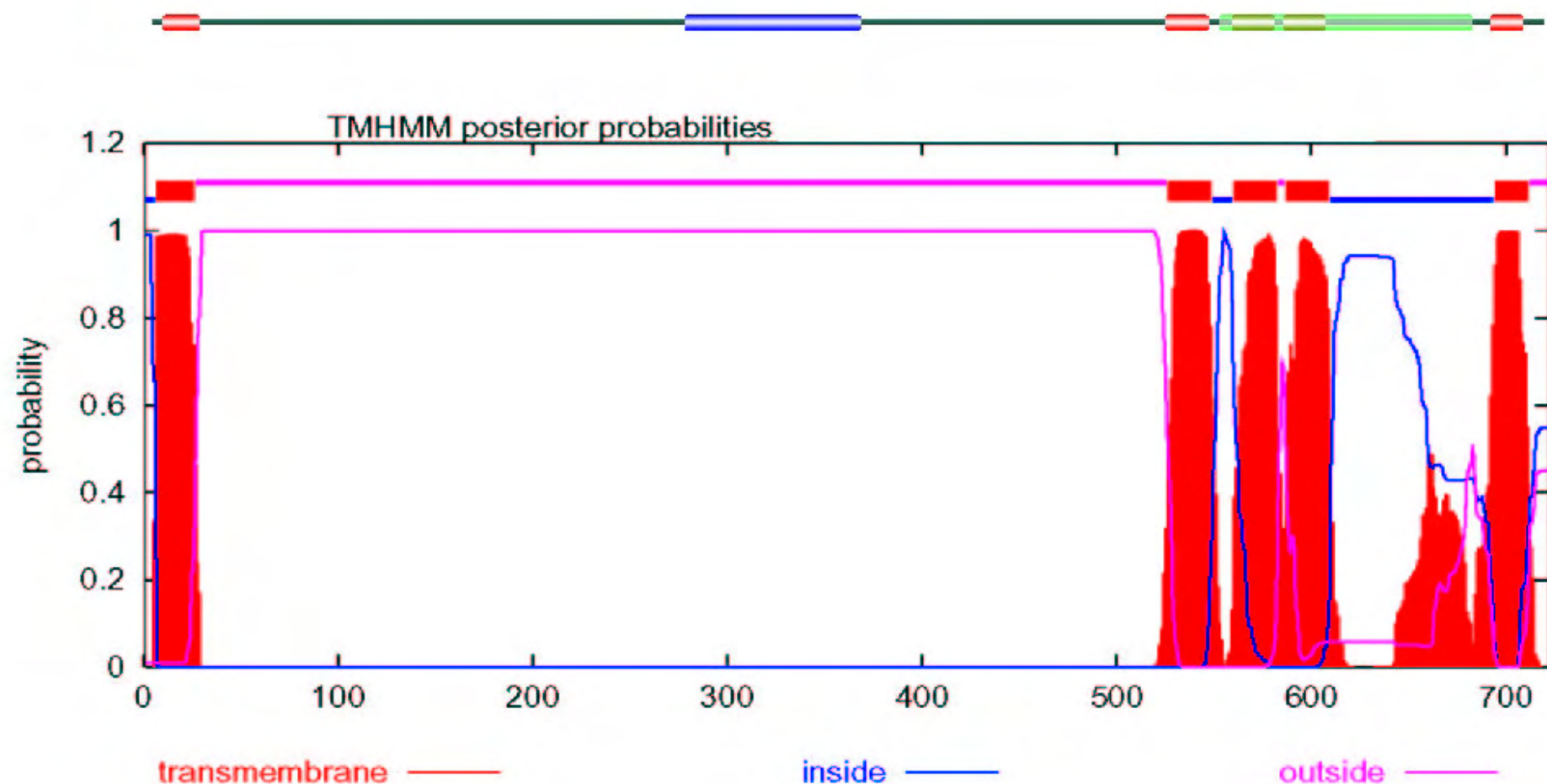

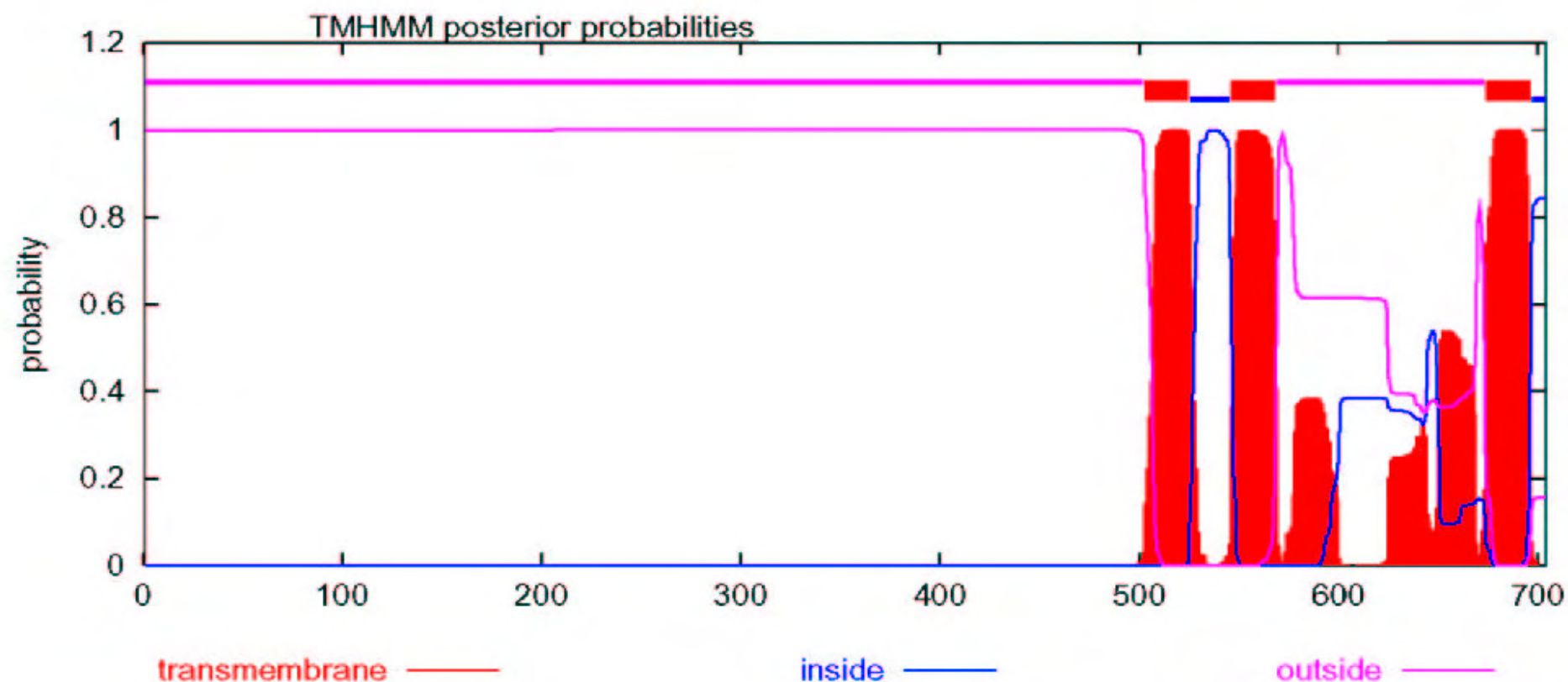

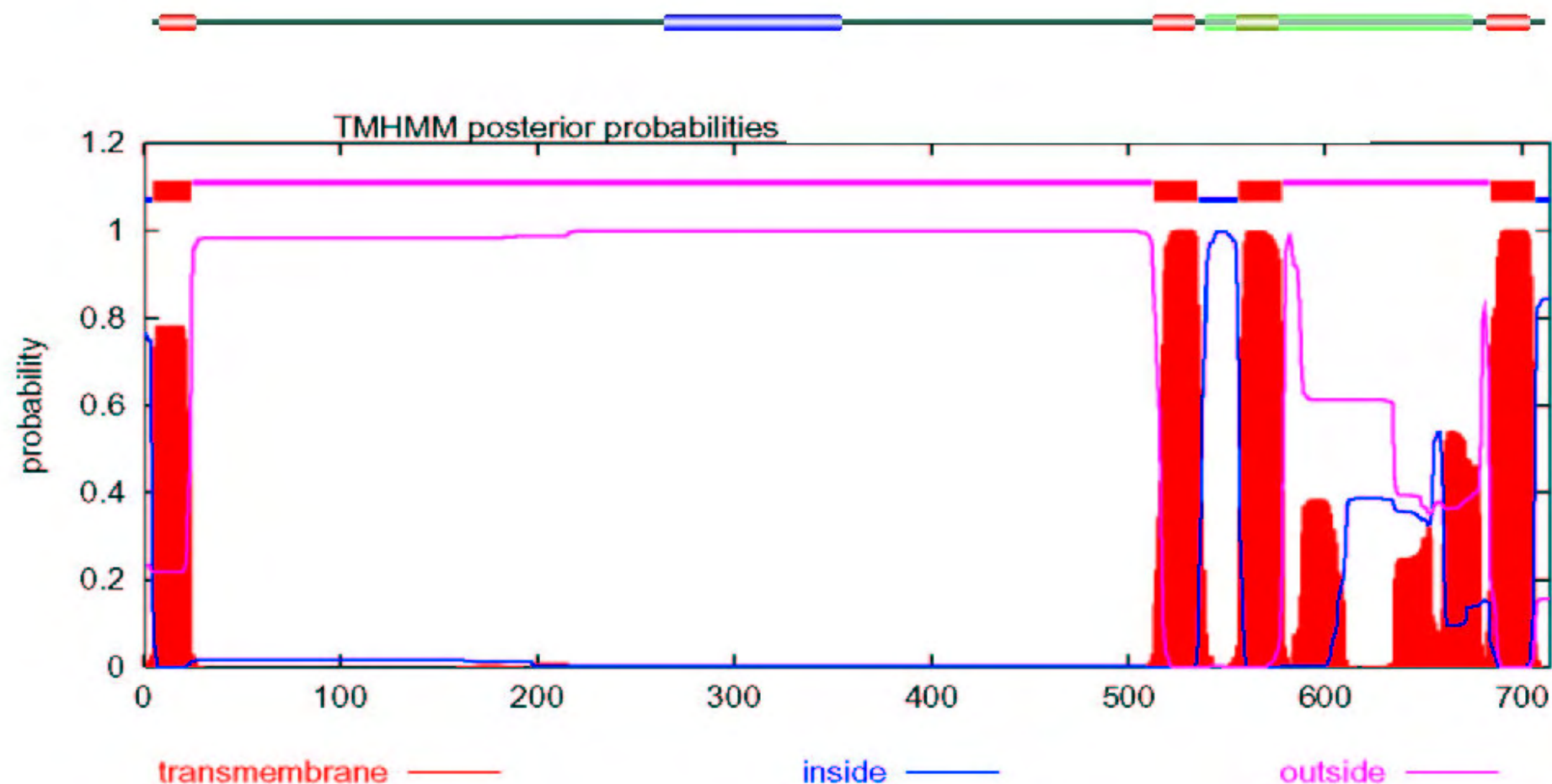

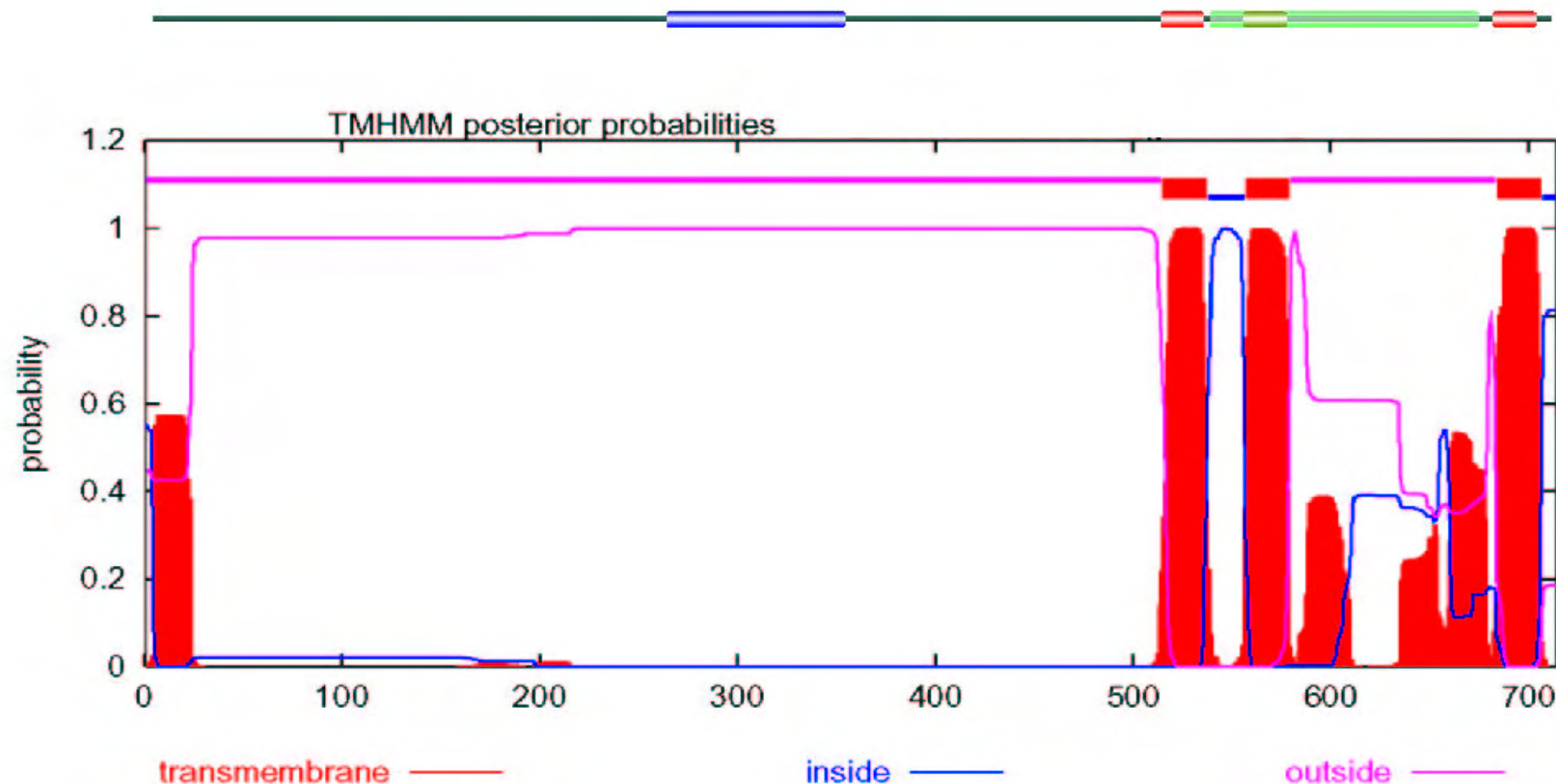

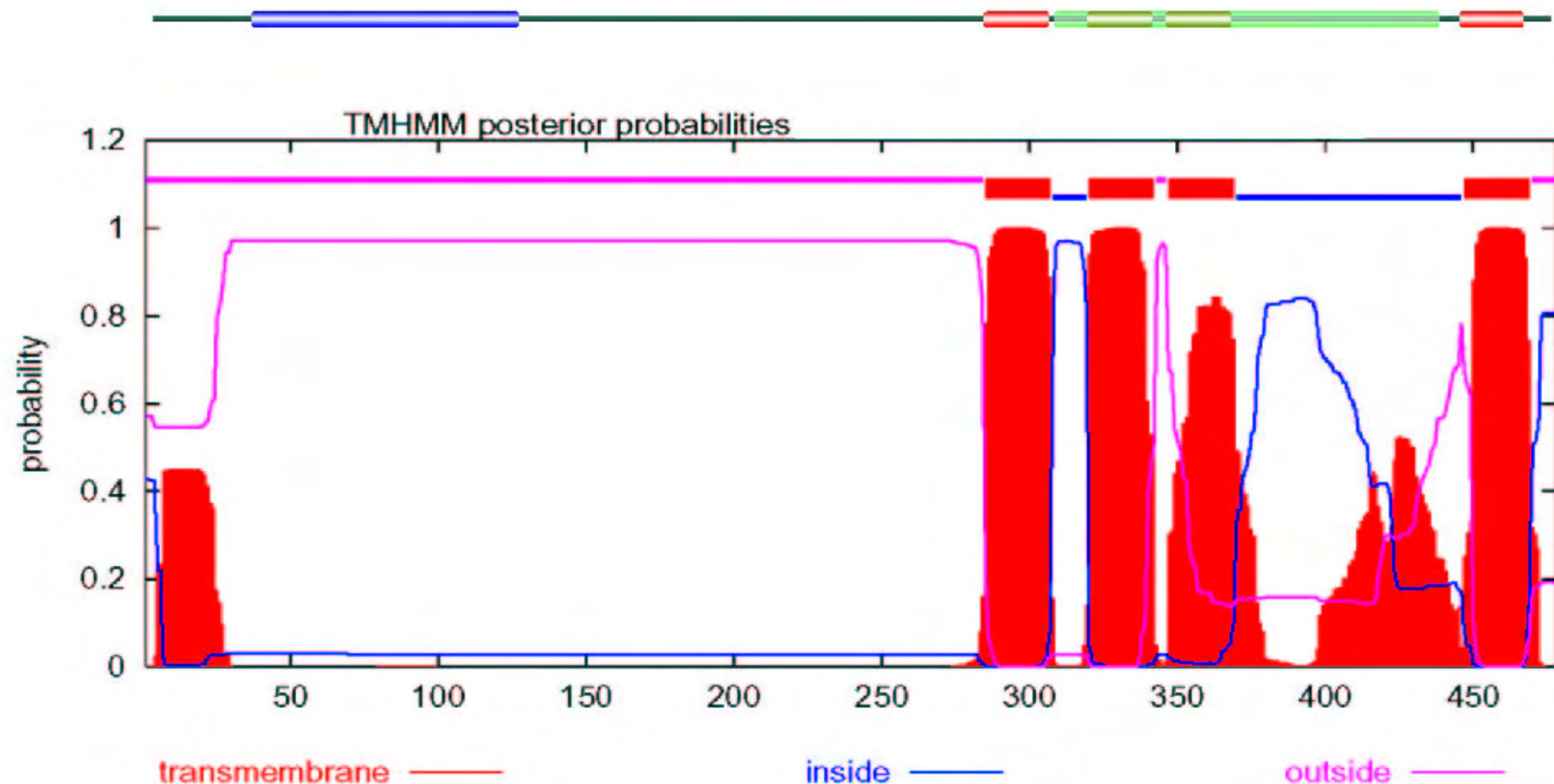

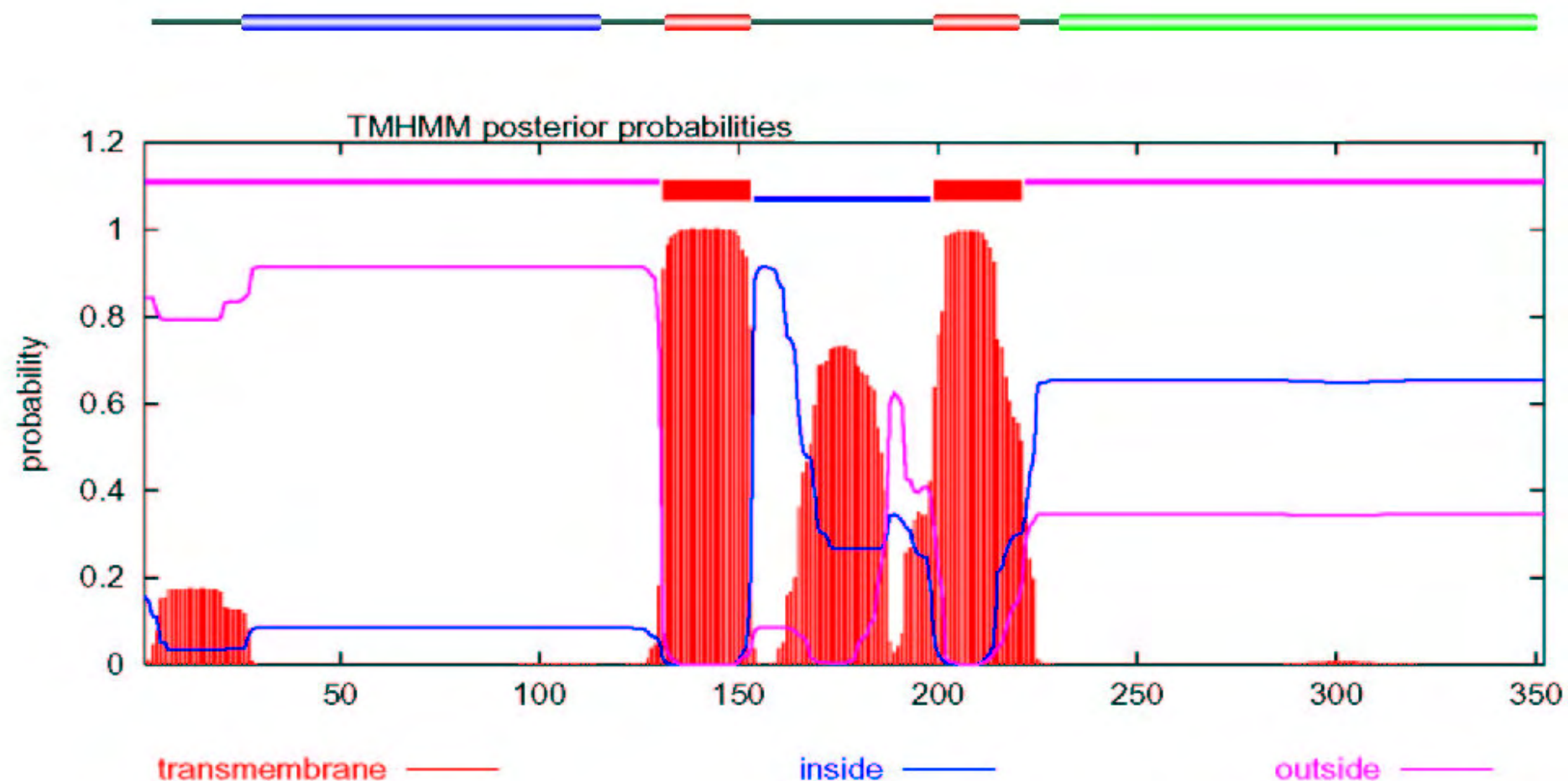

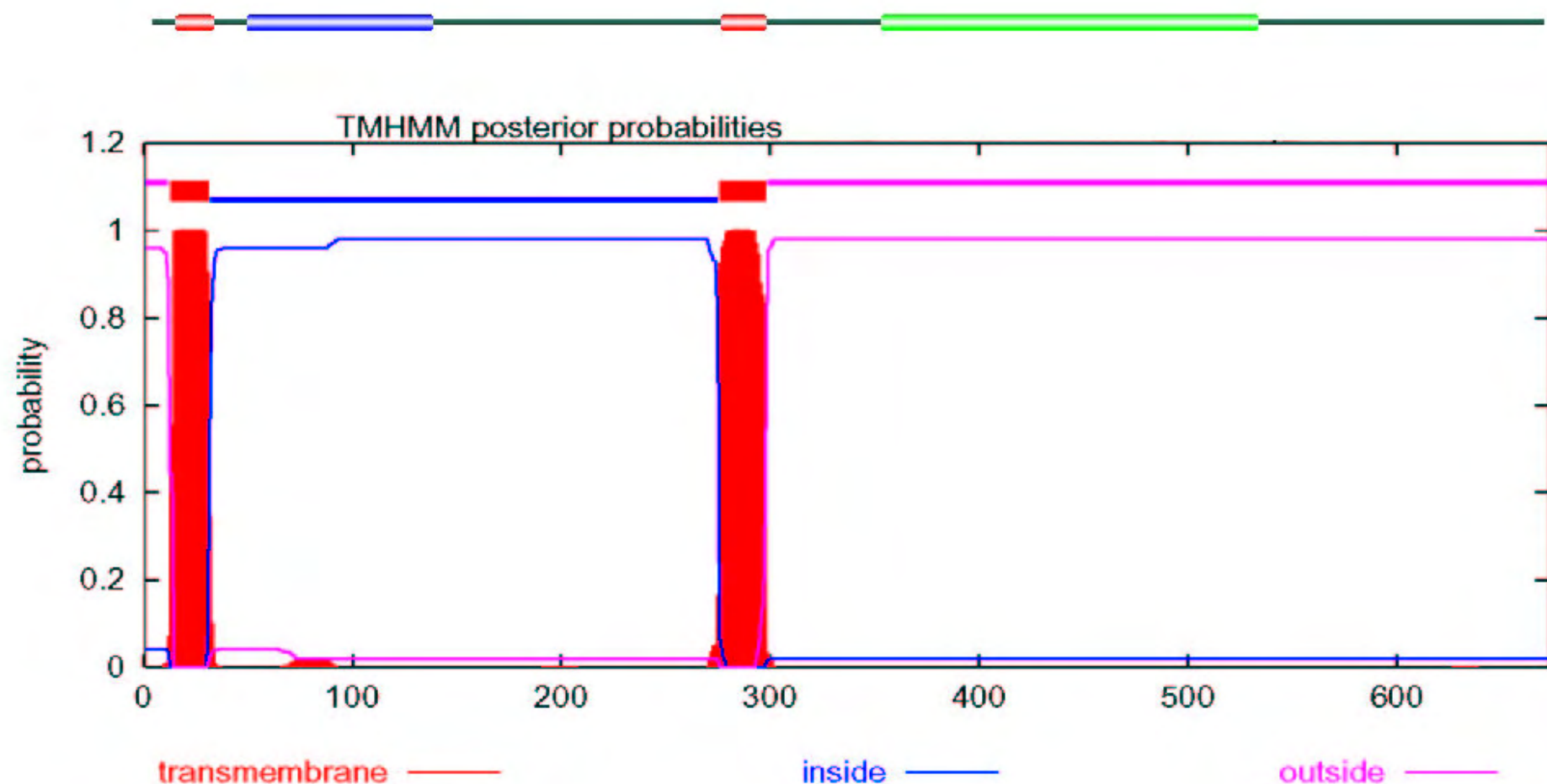

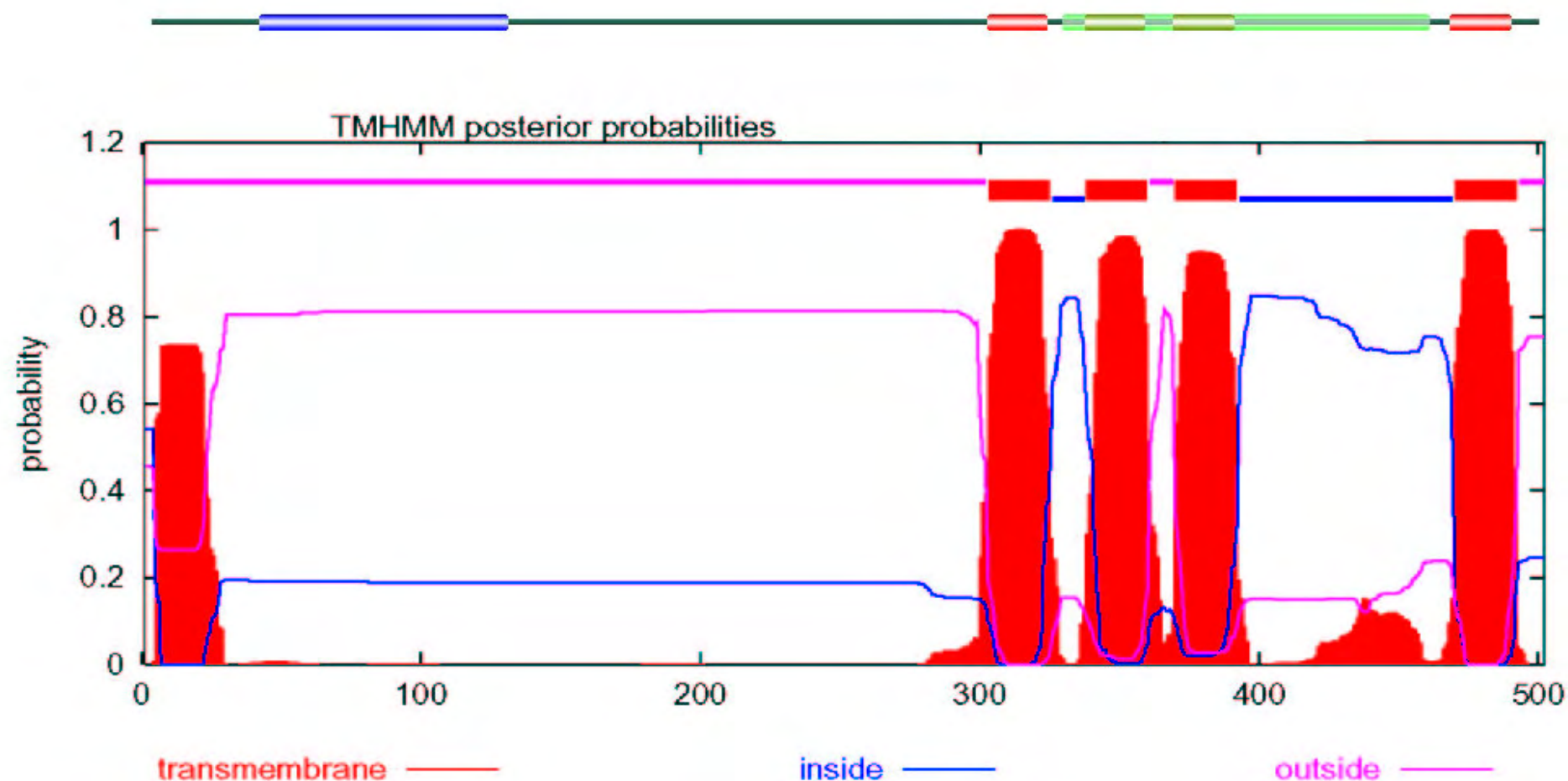

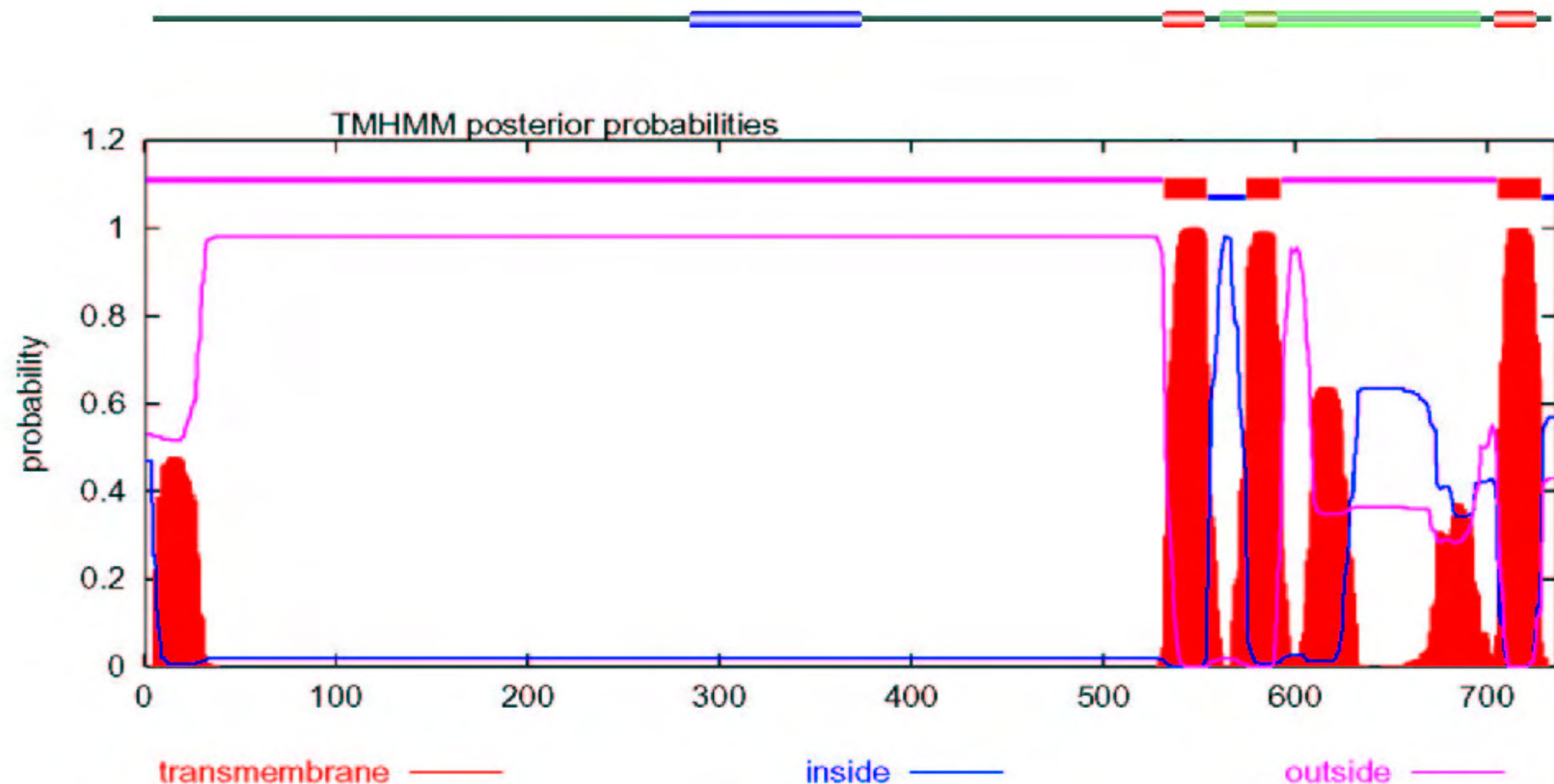

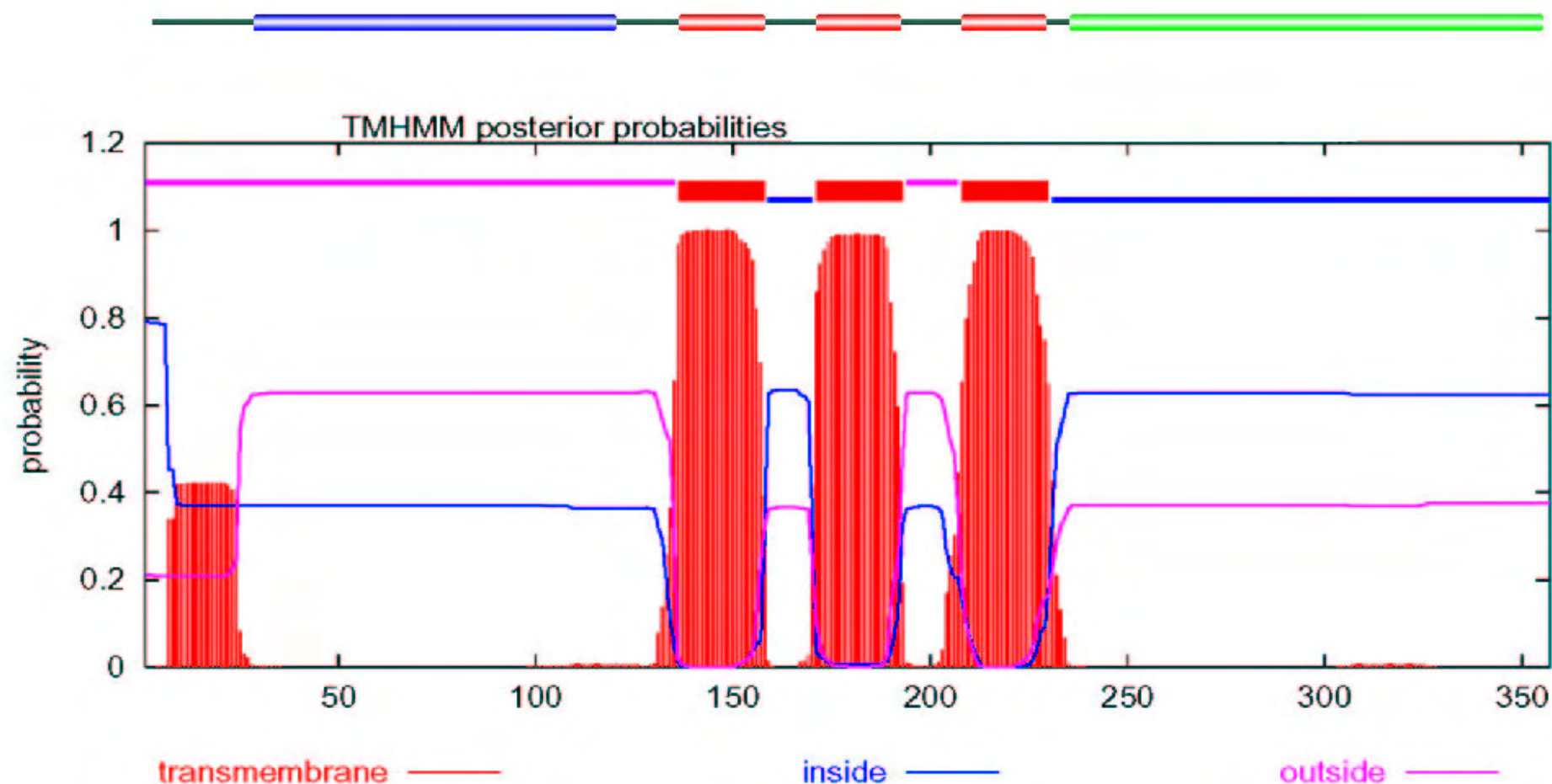

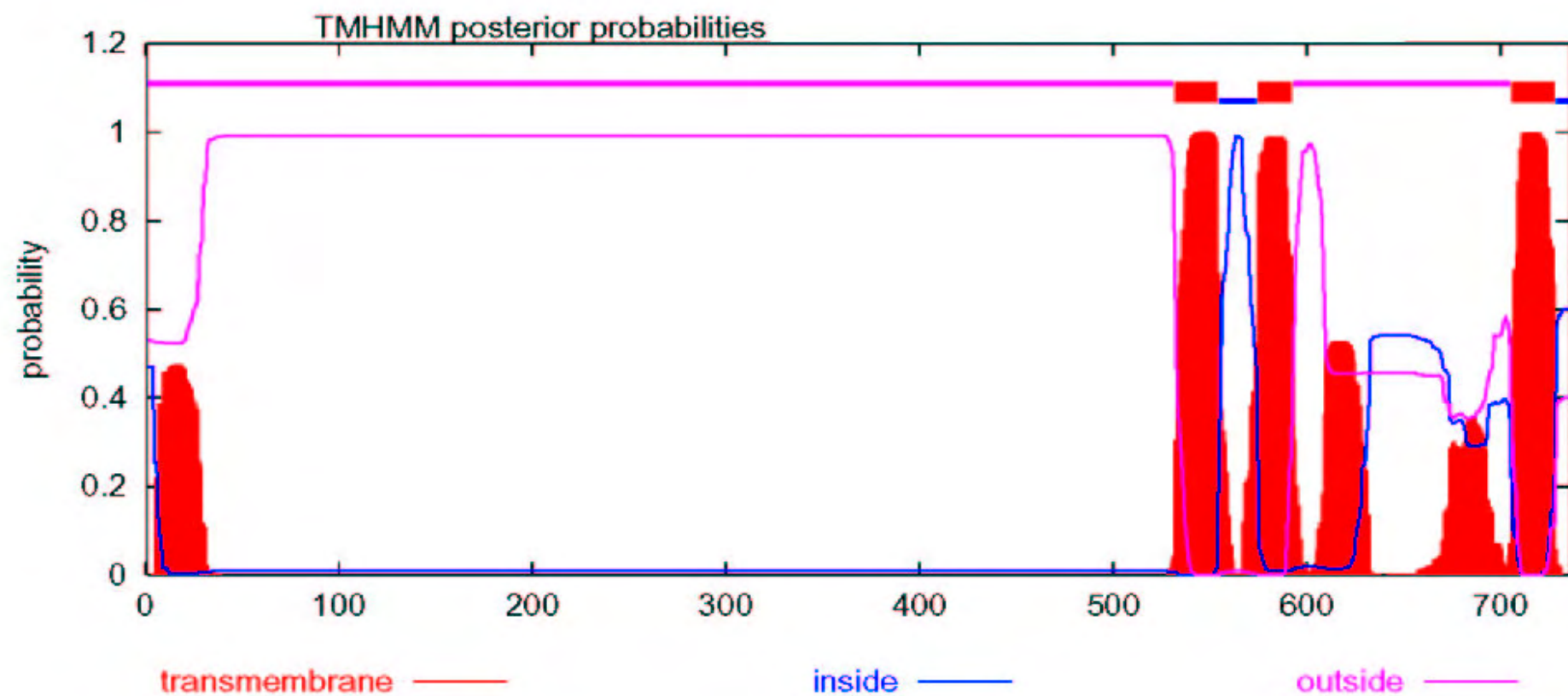

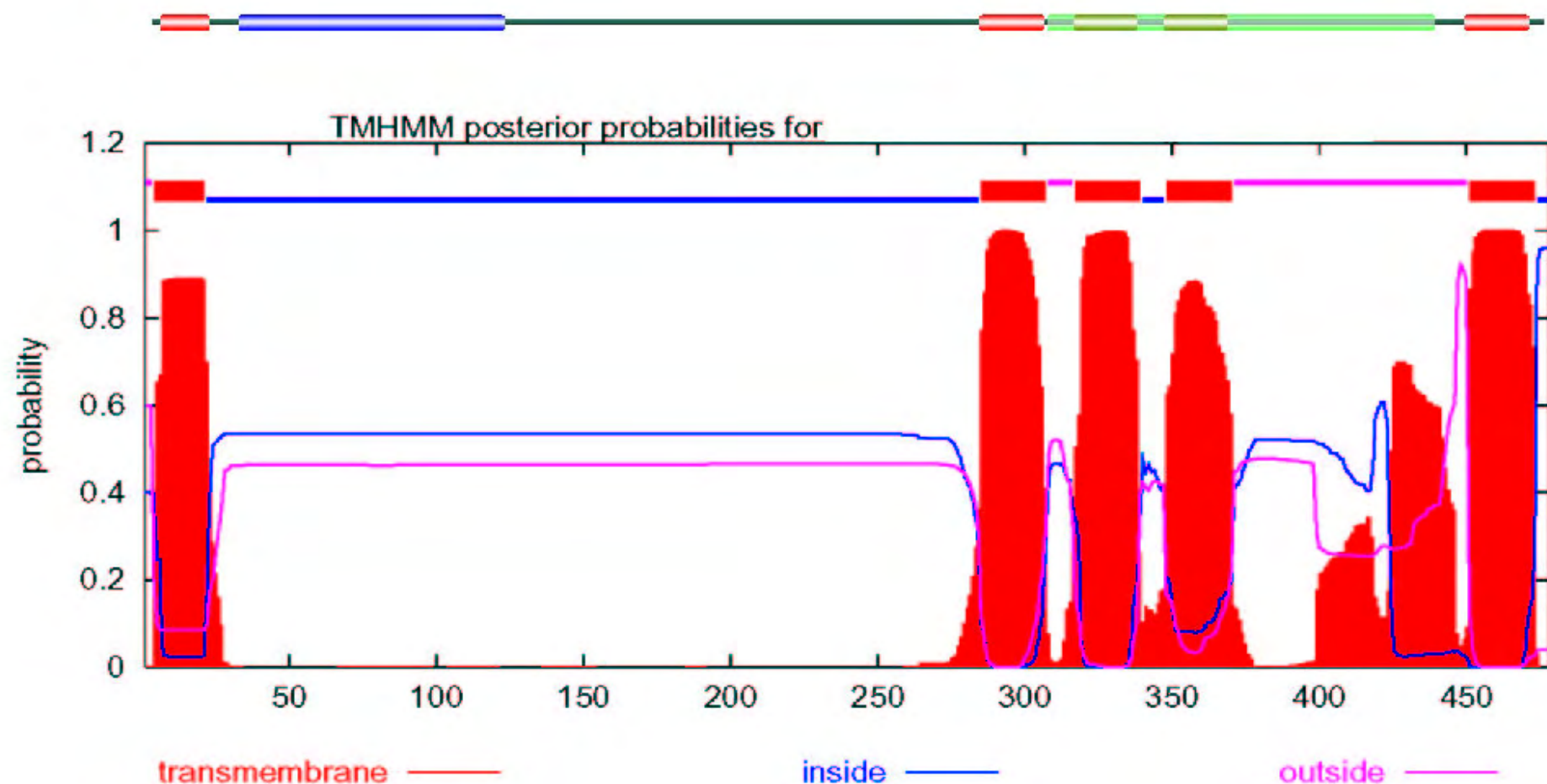

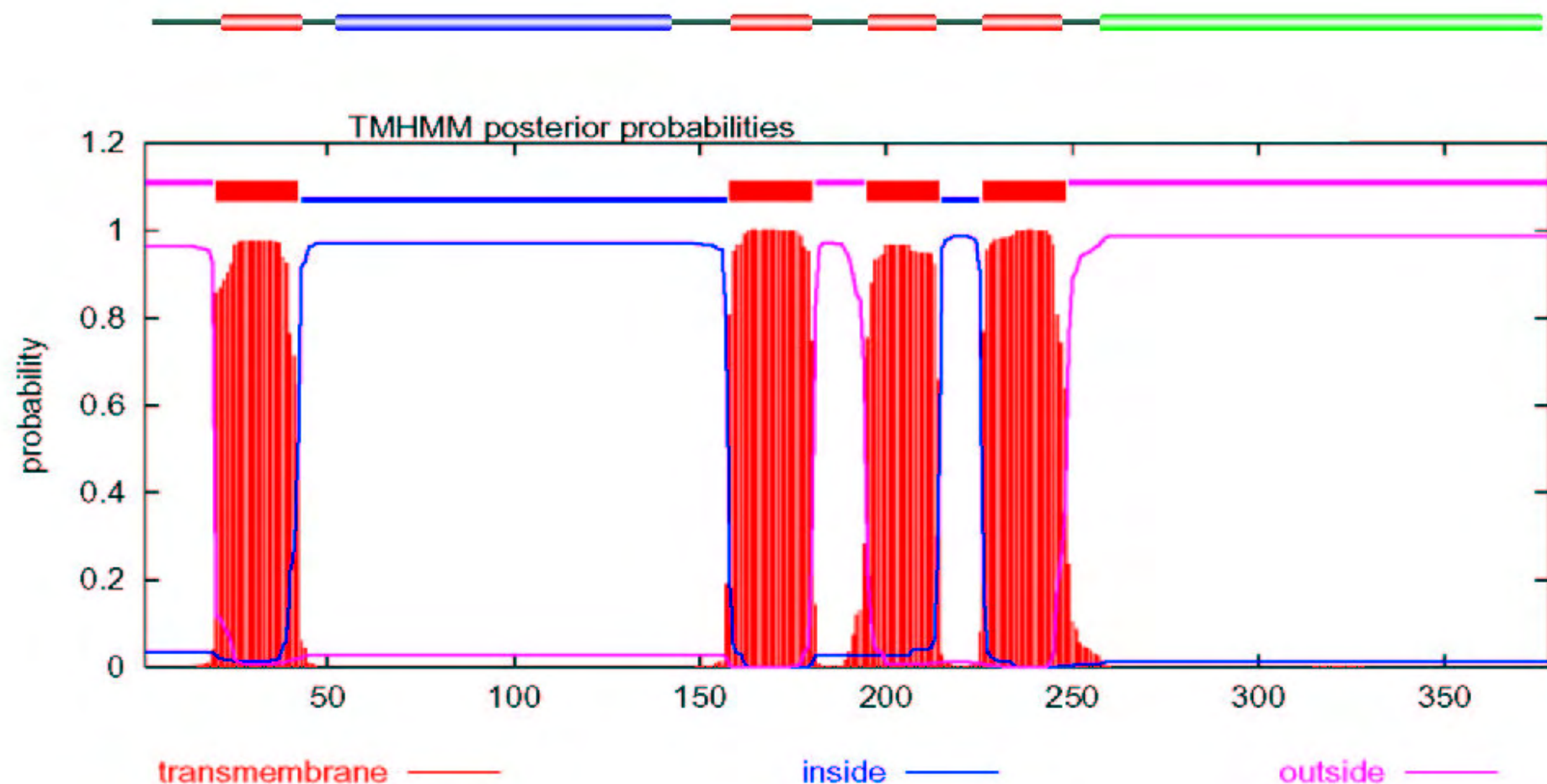

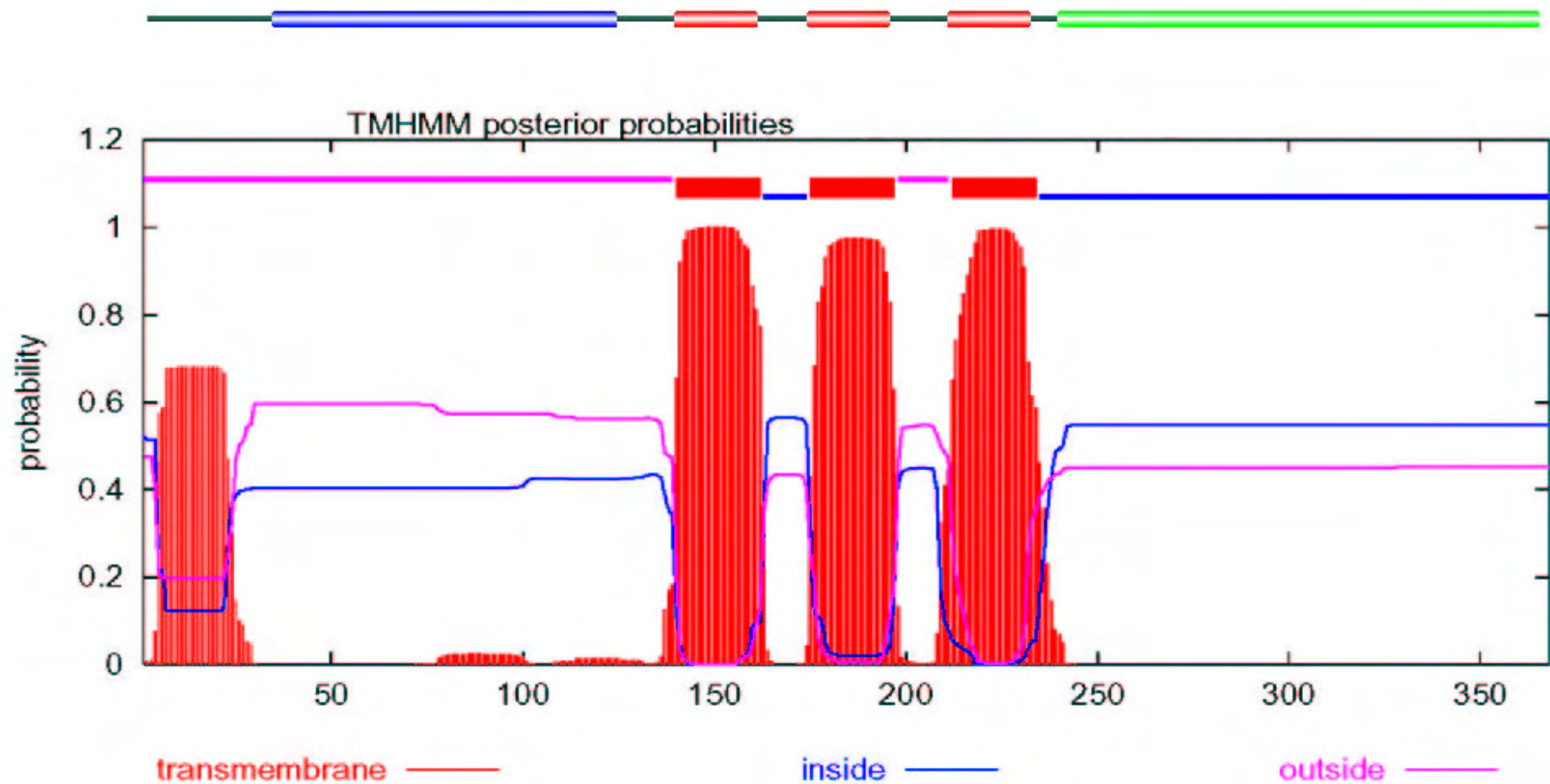

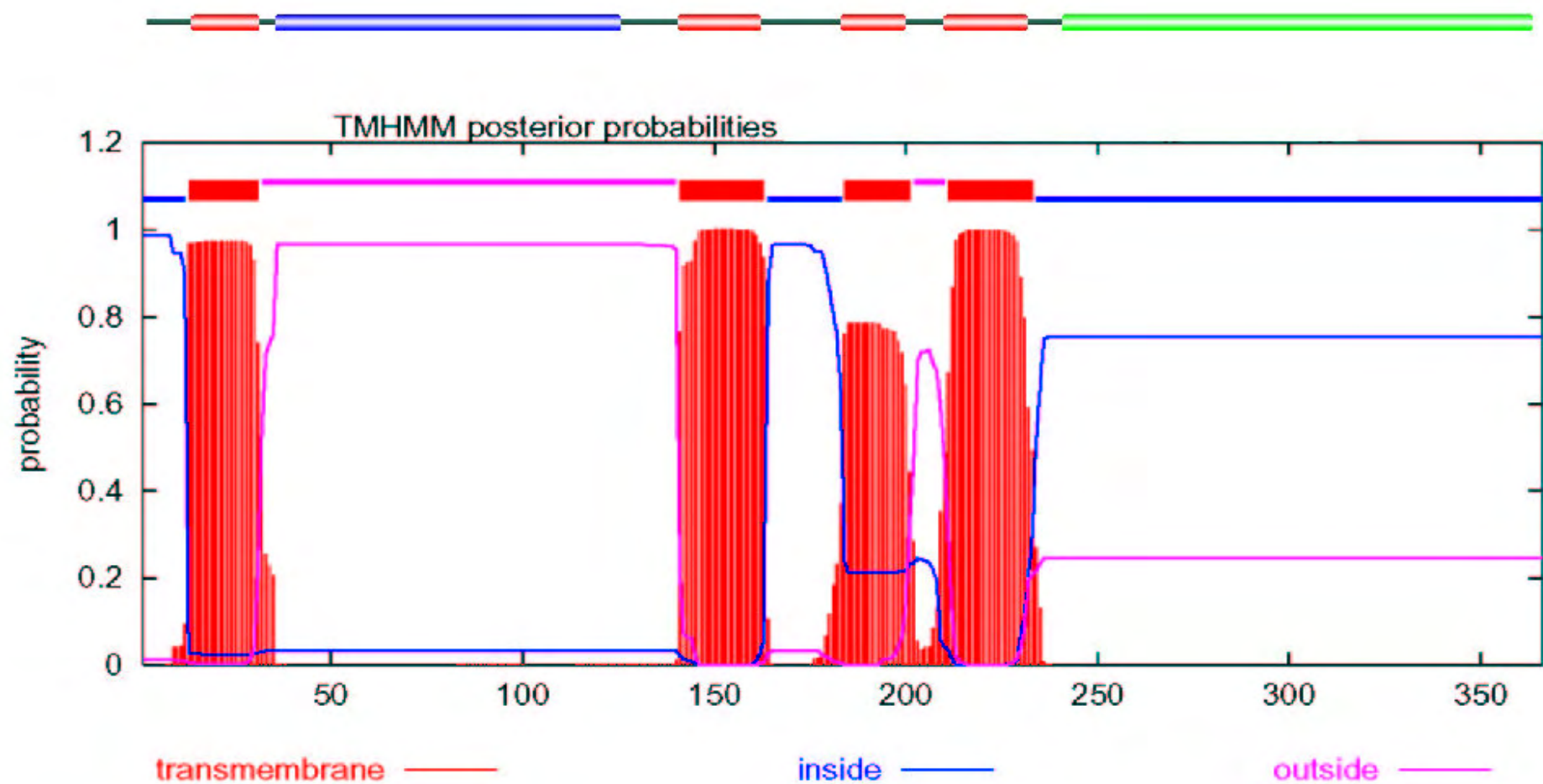

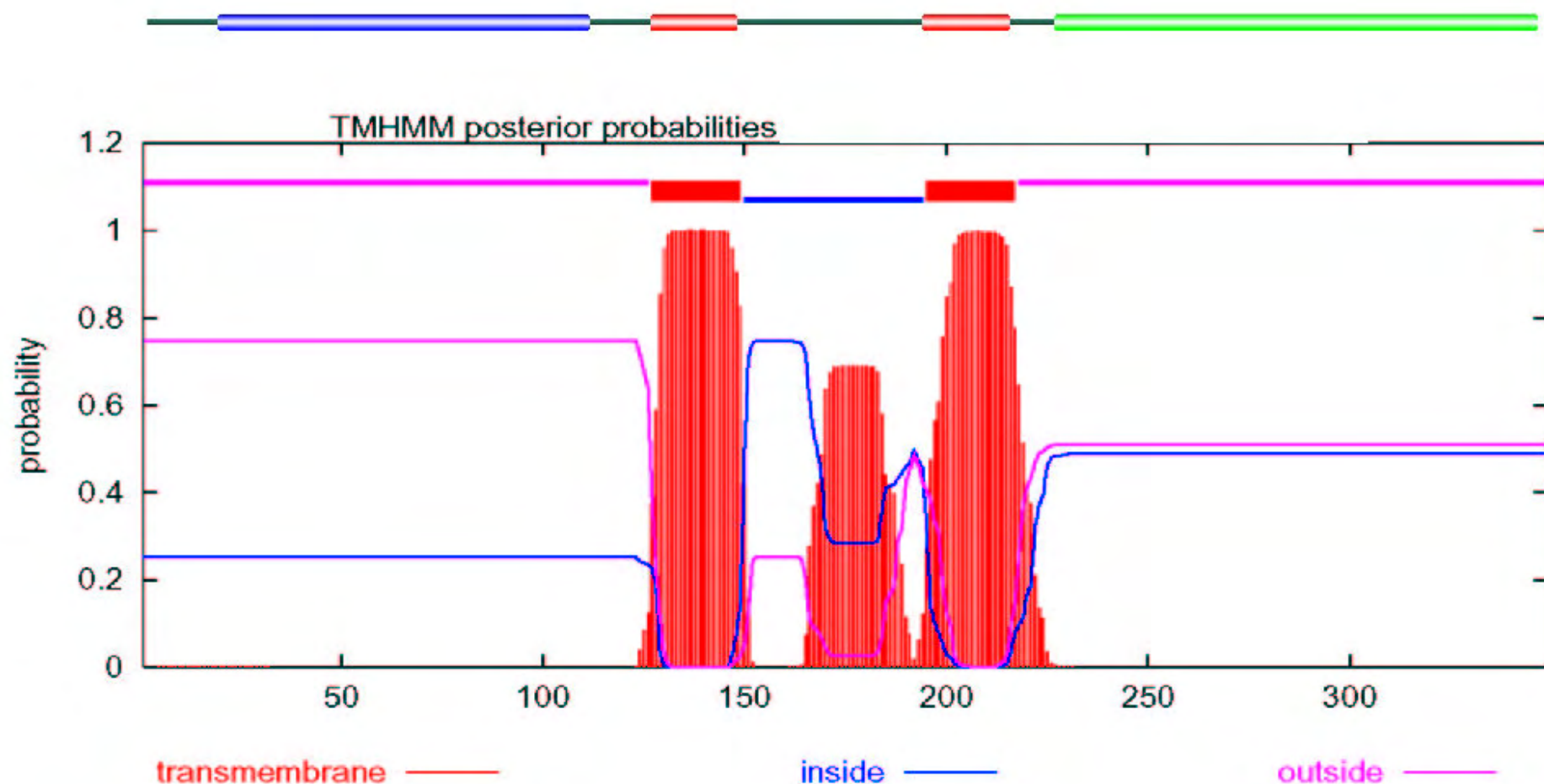

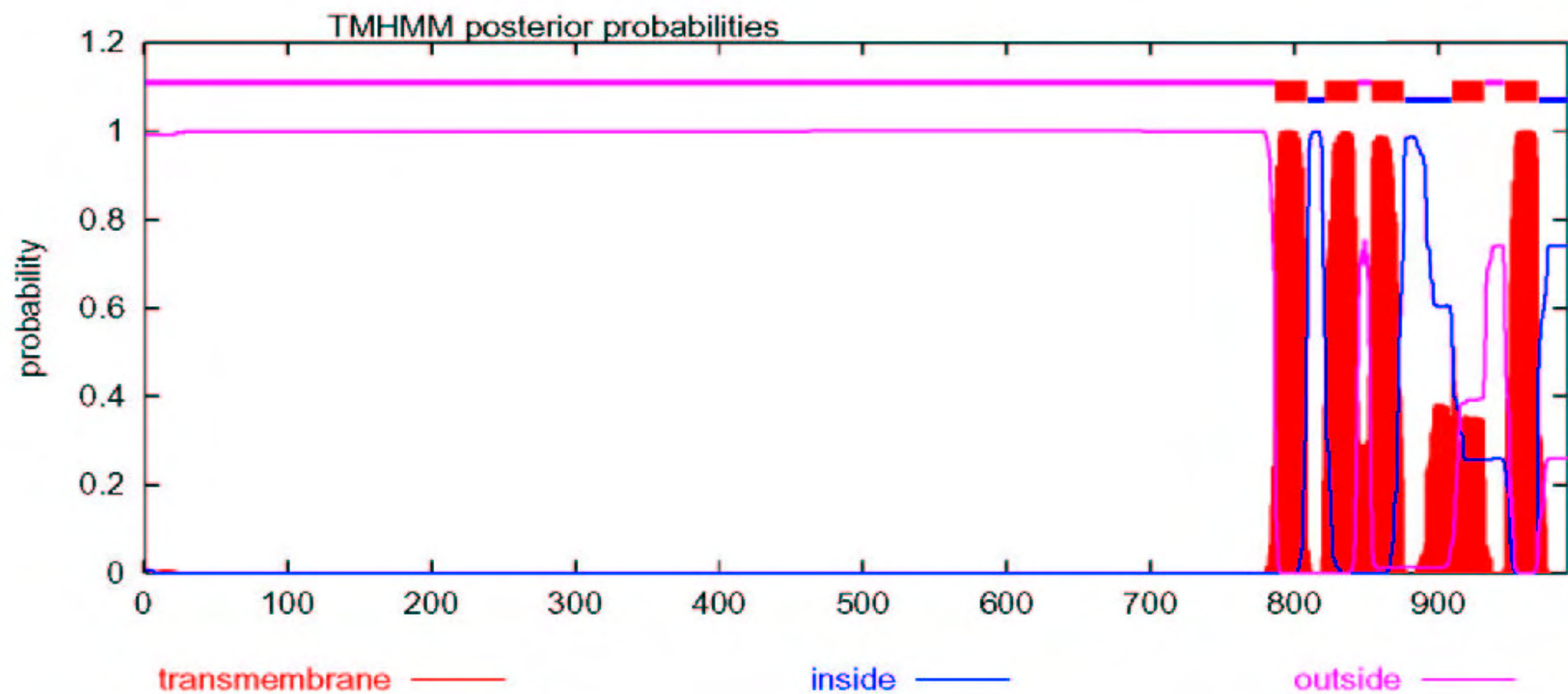

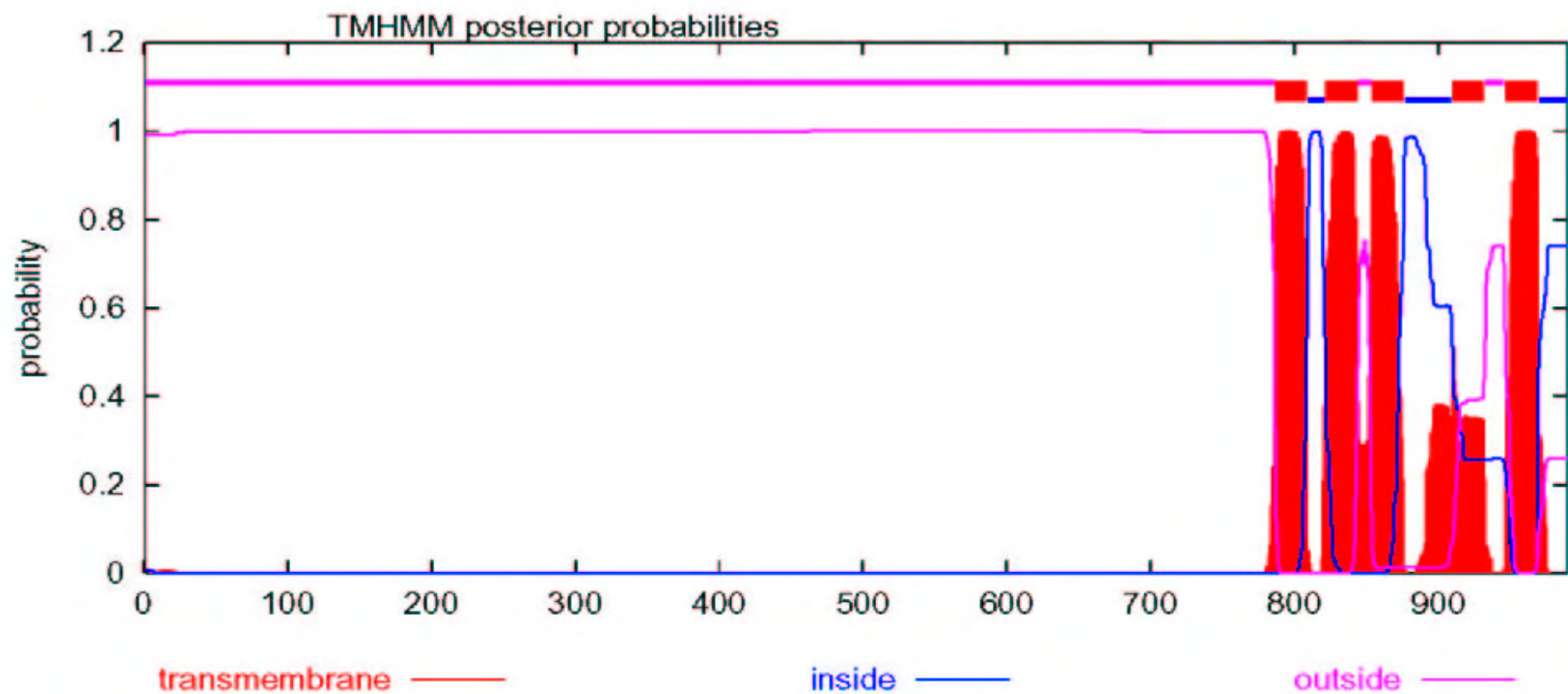

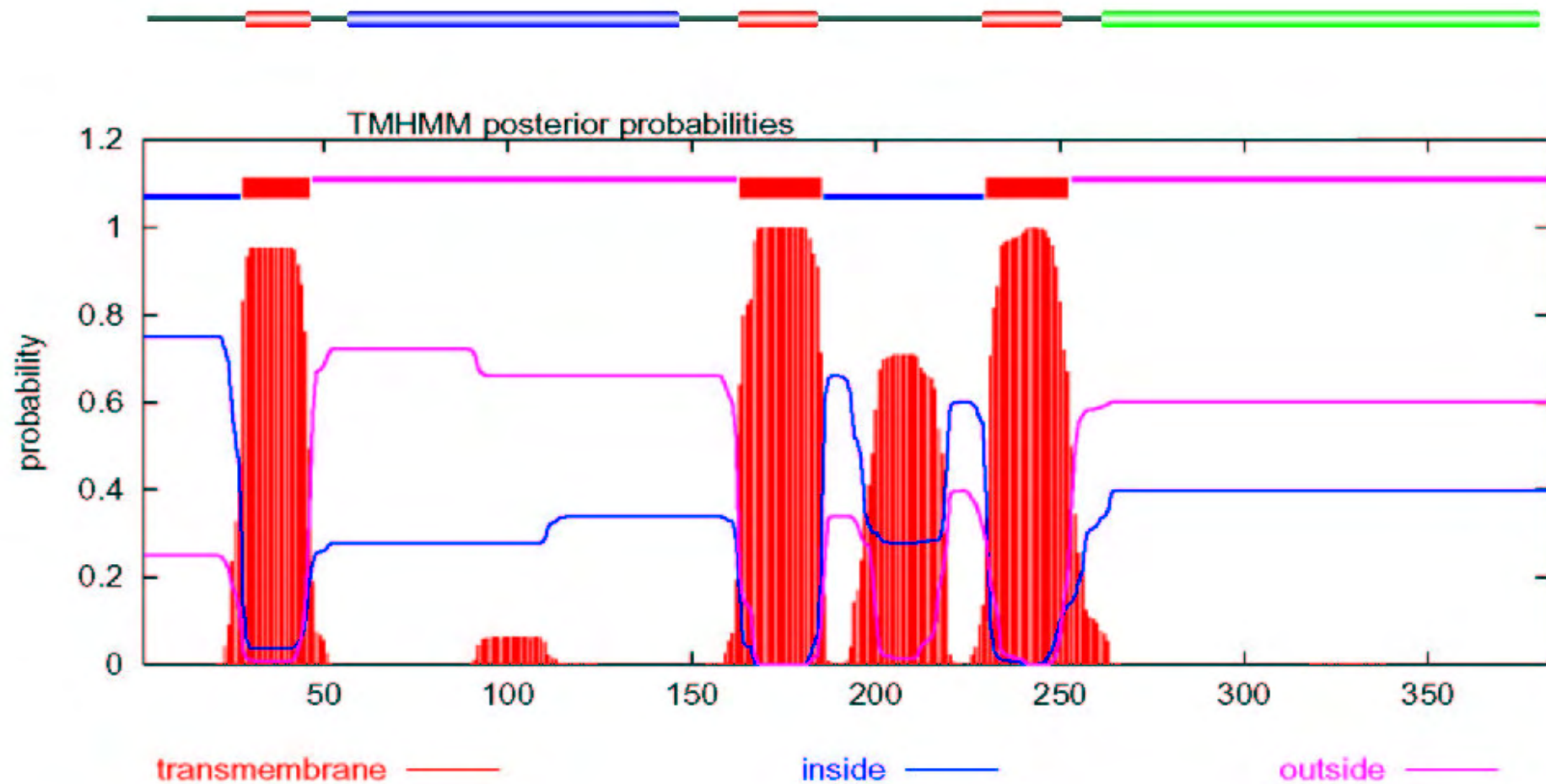

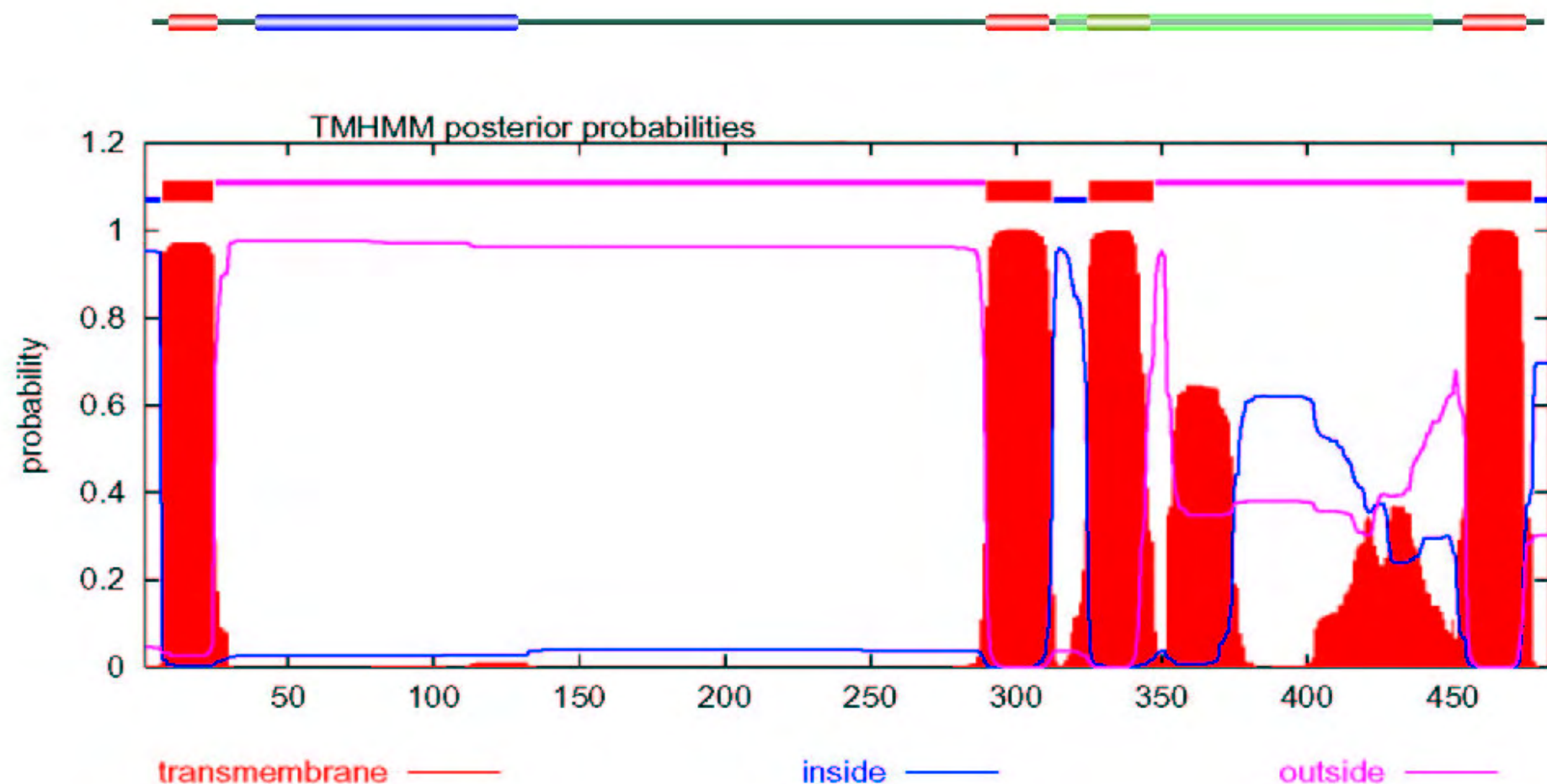

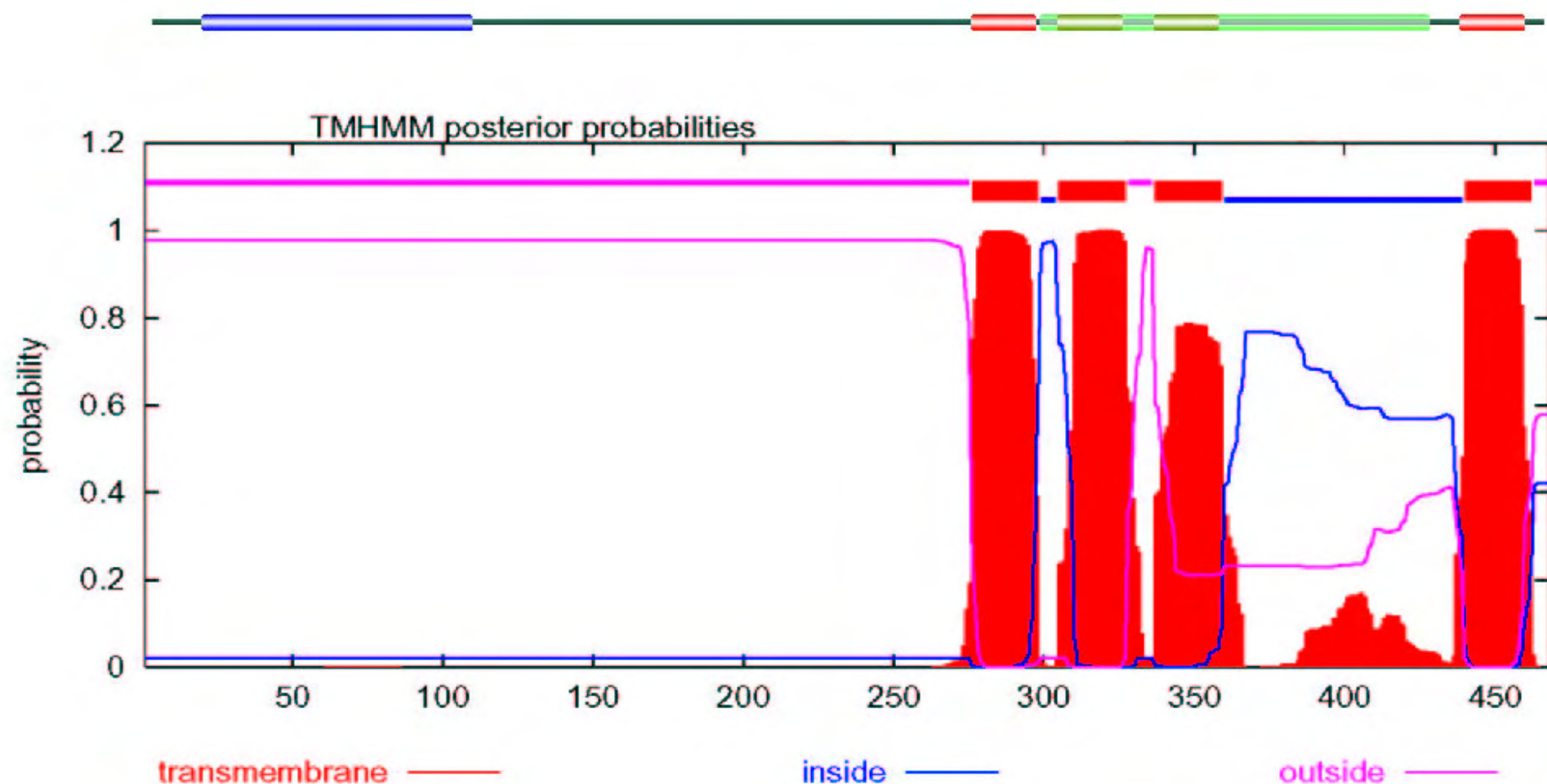

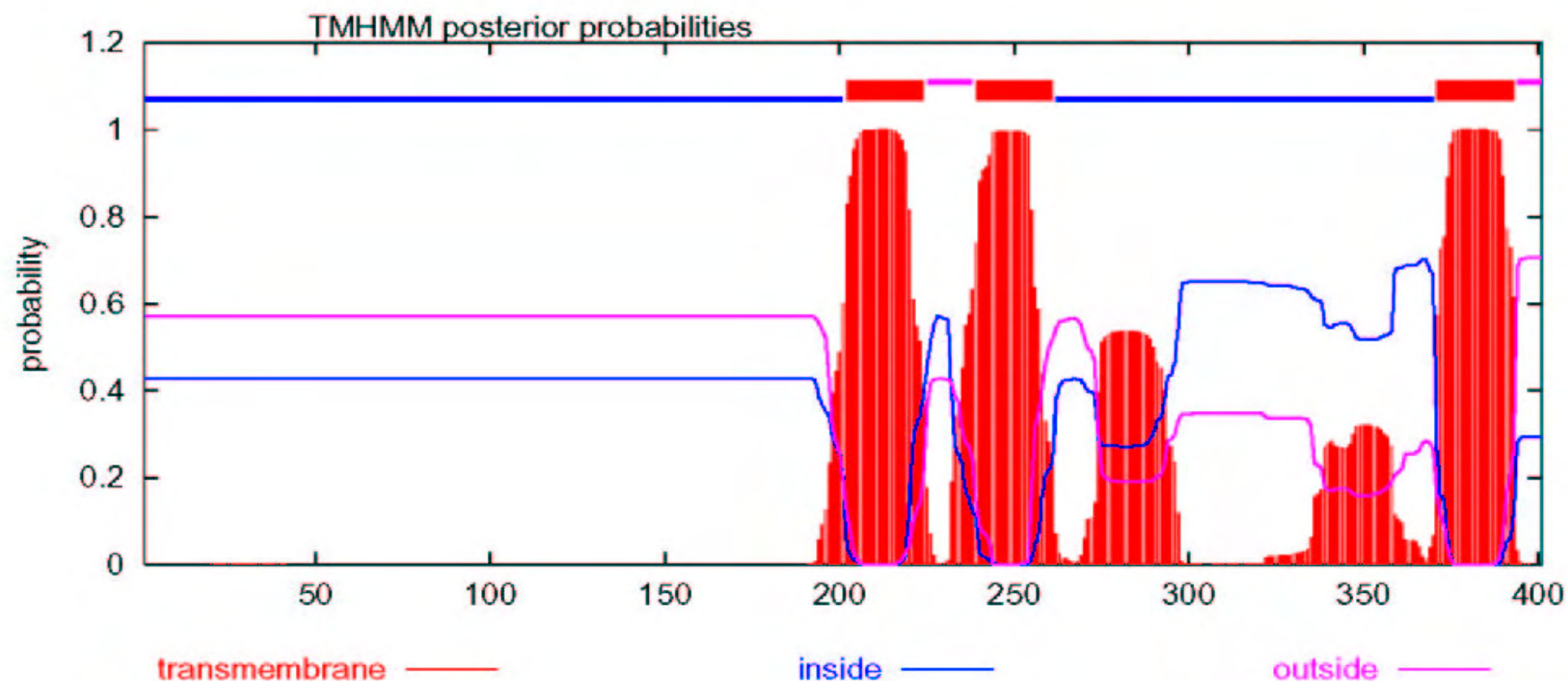

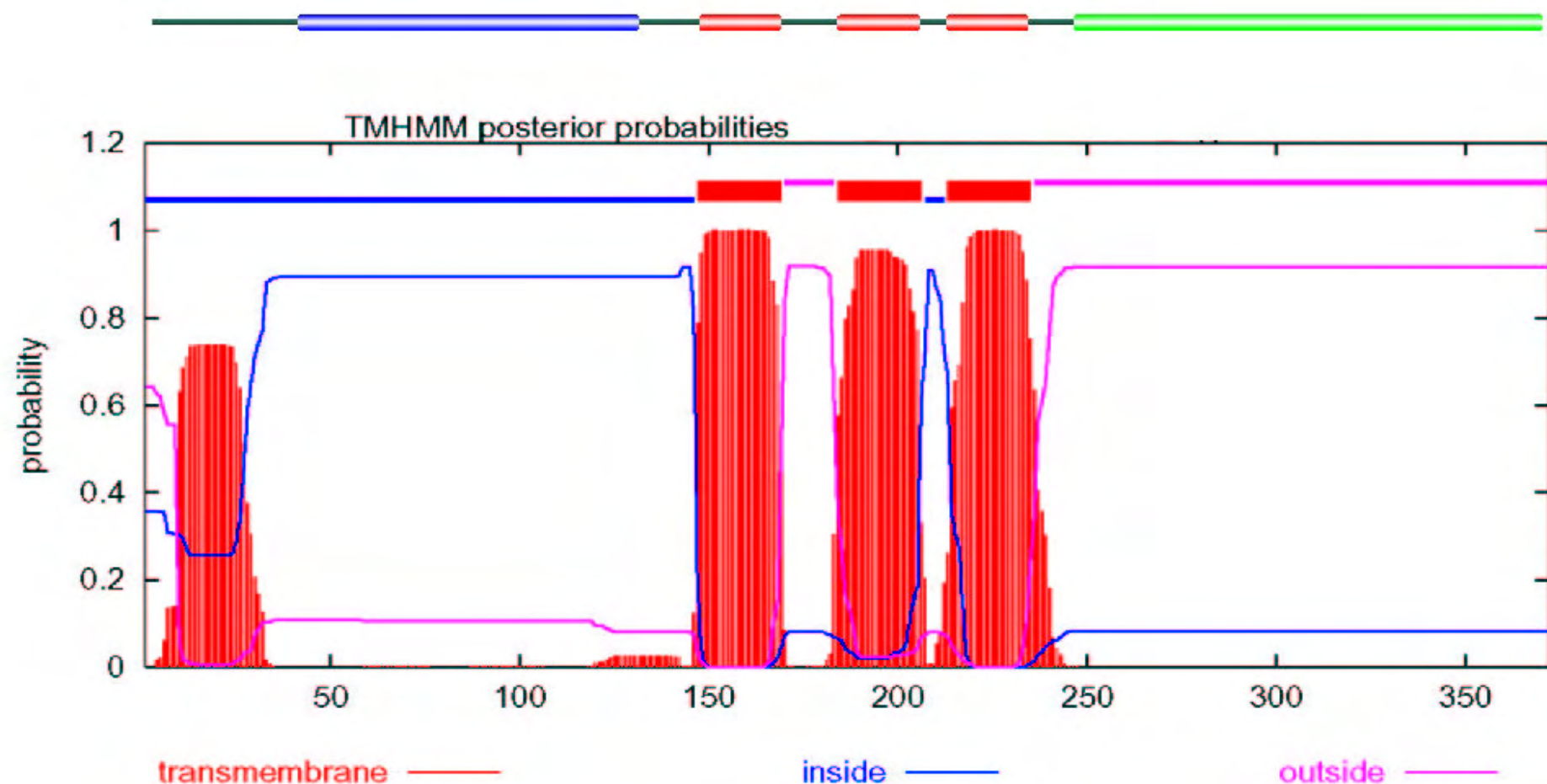

50

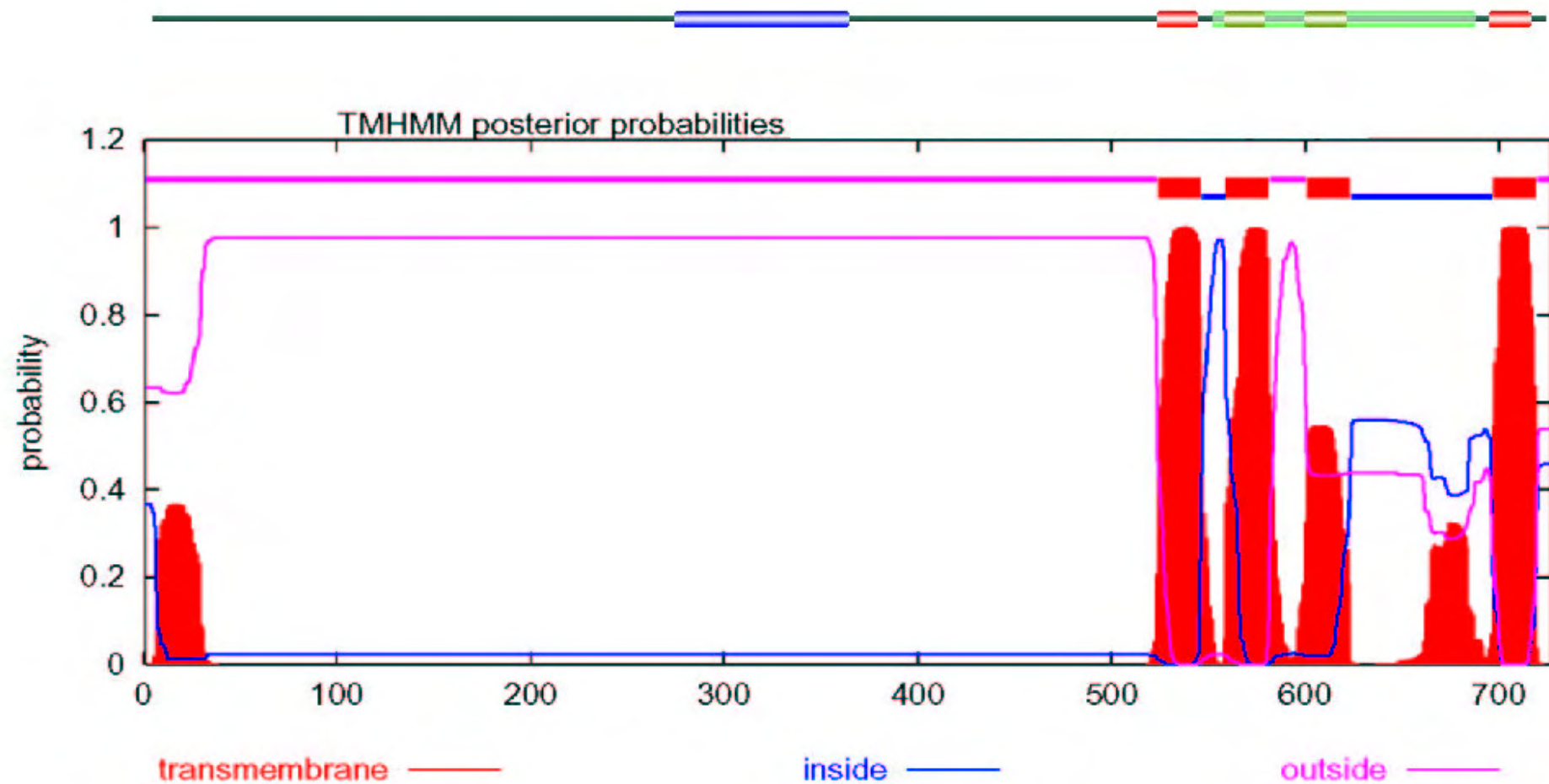

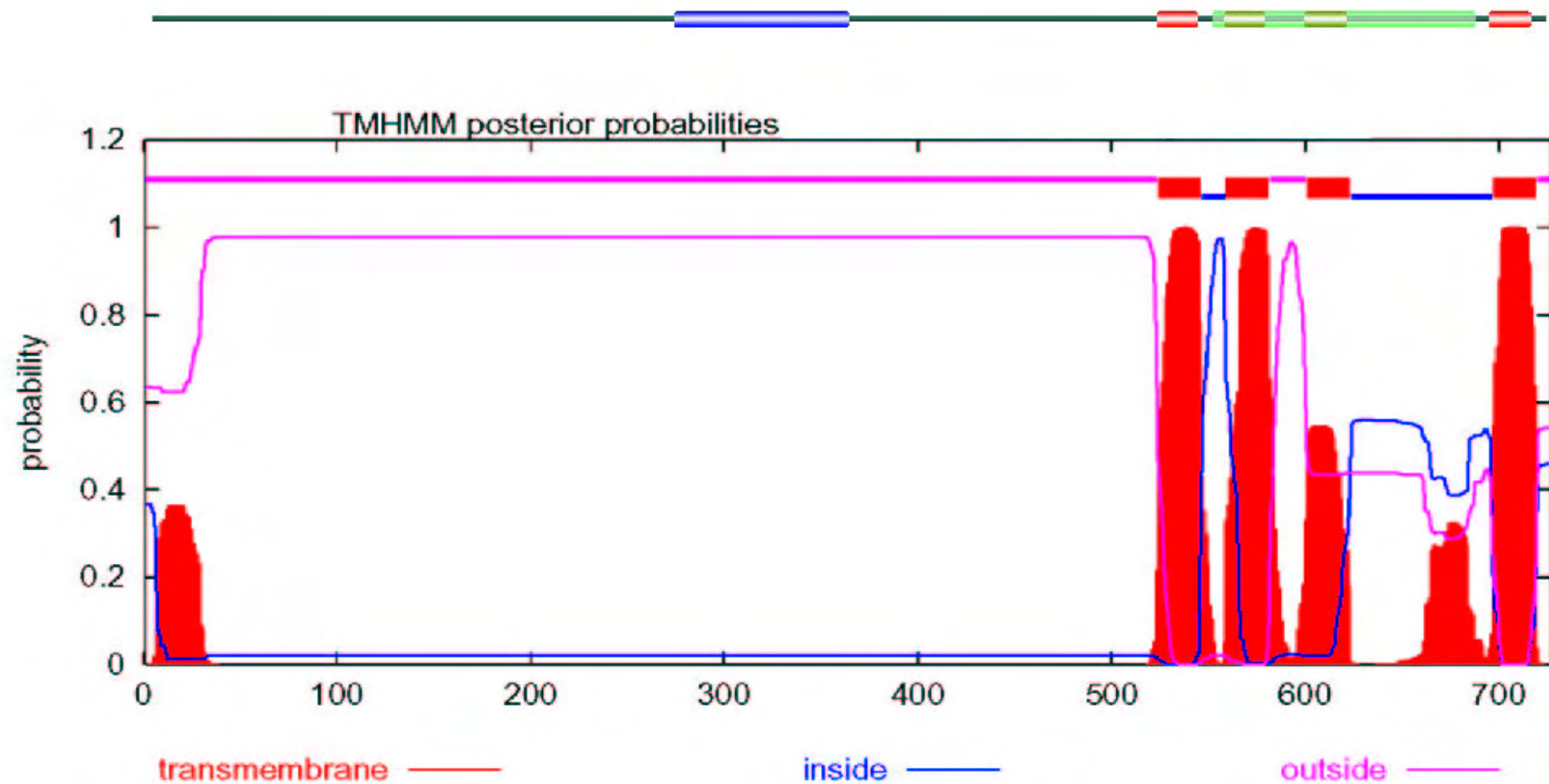

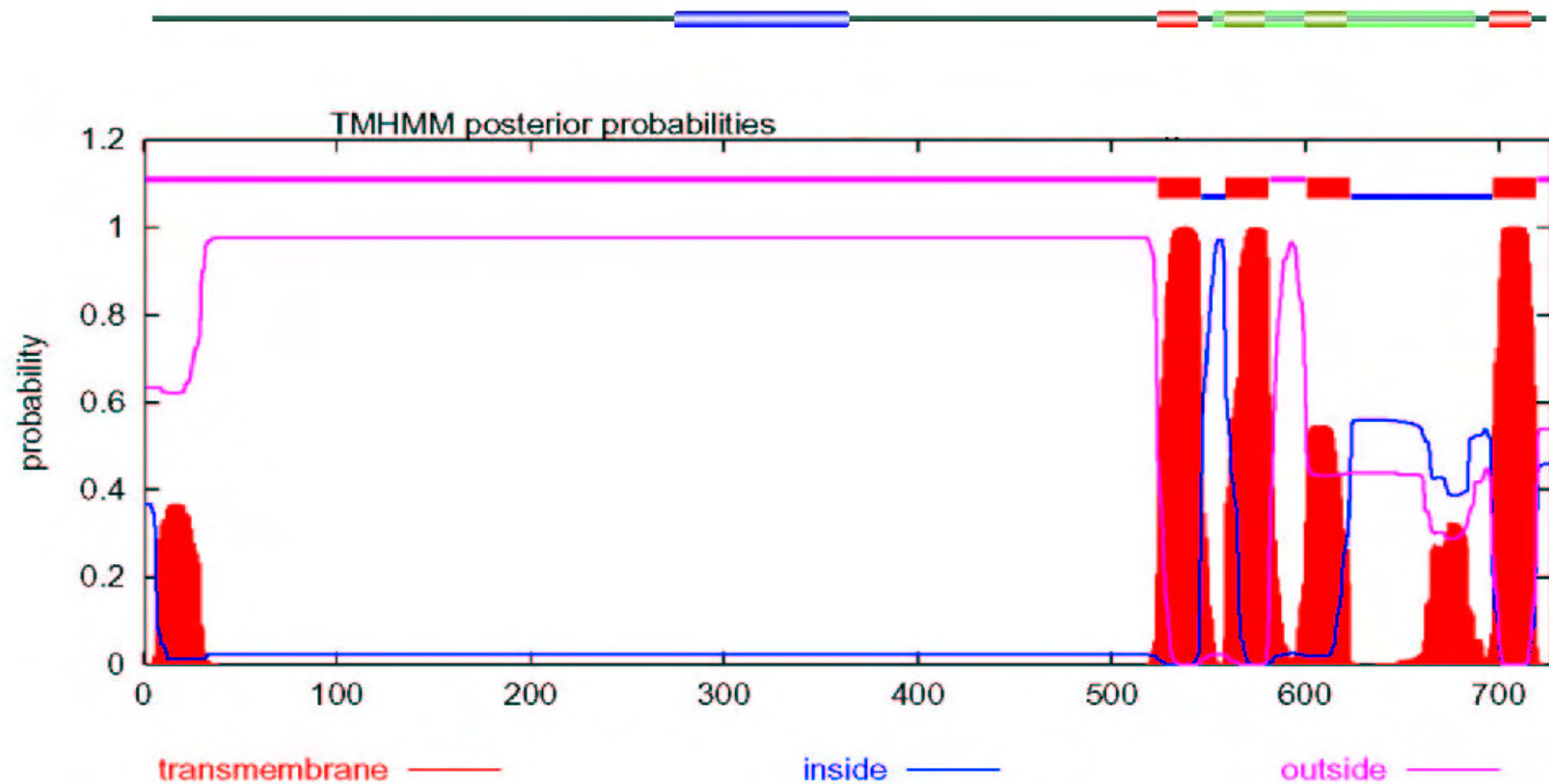

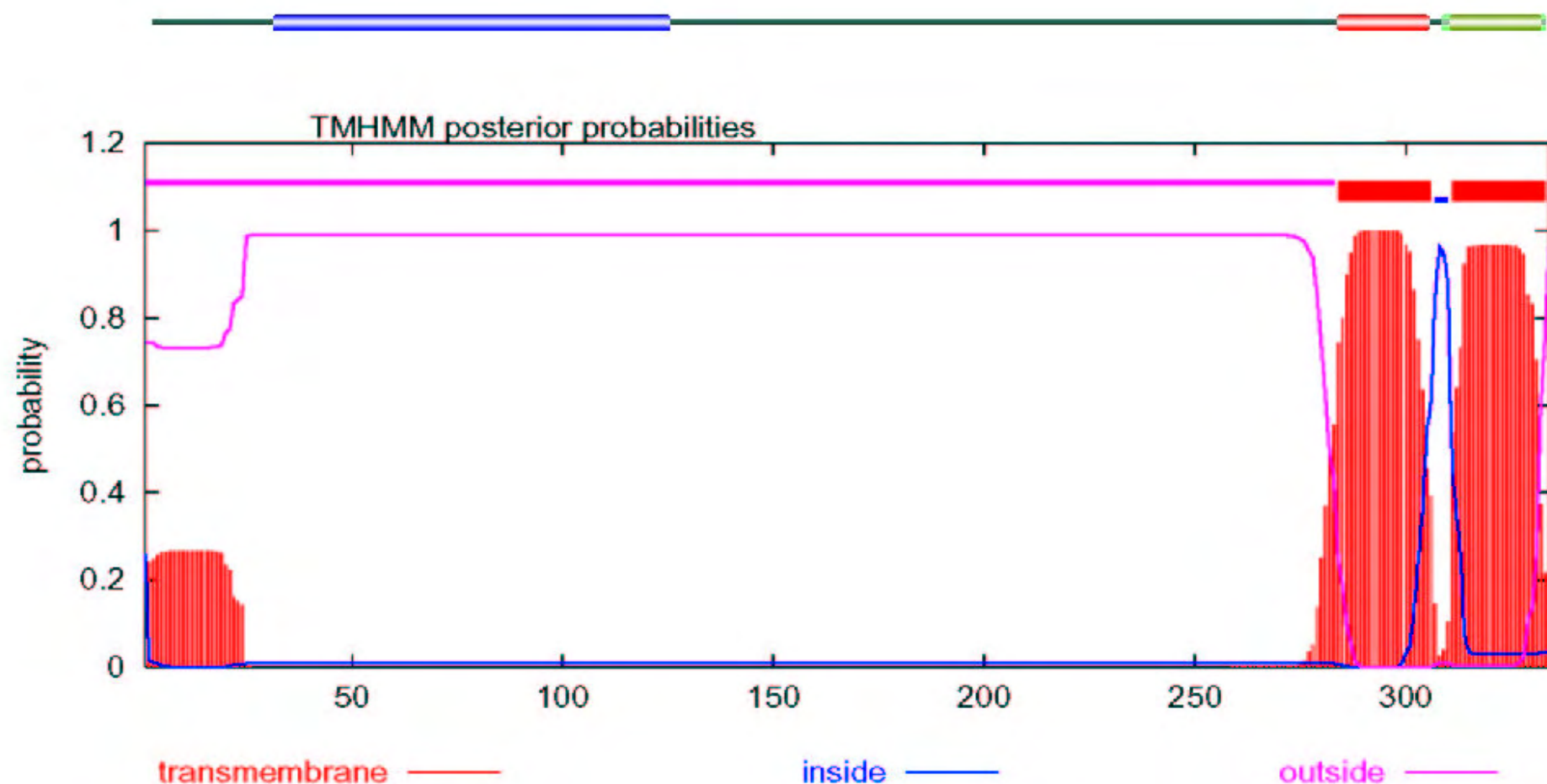

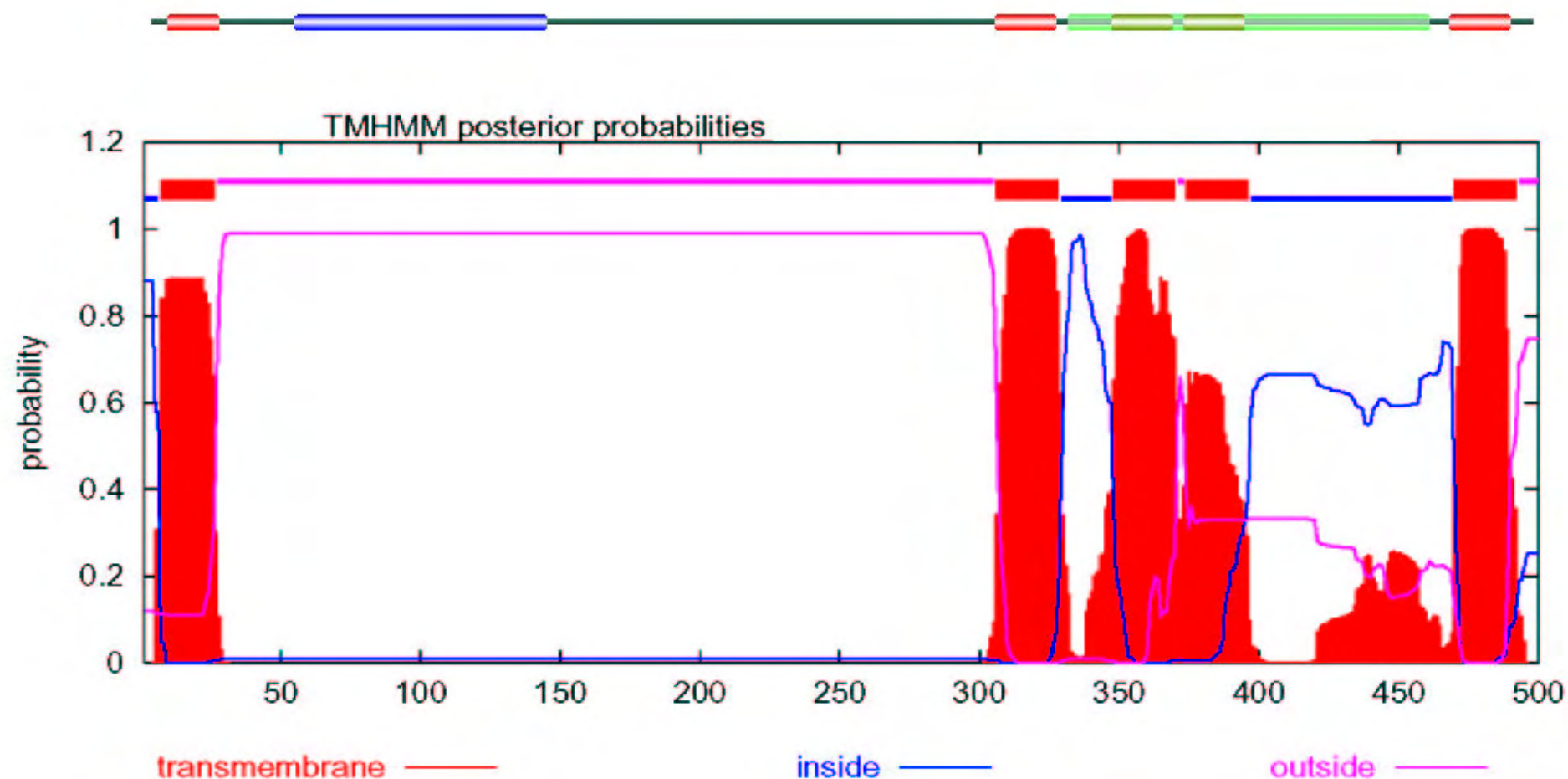

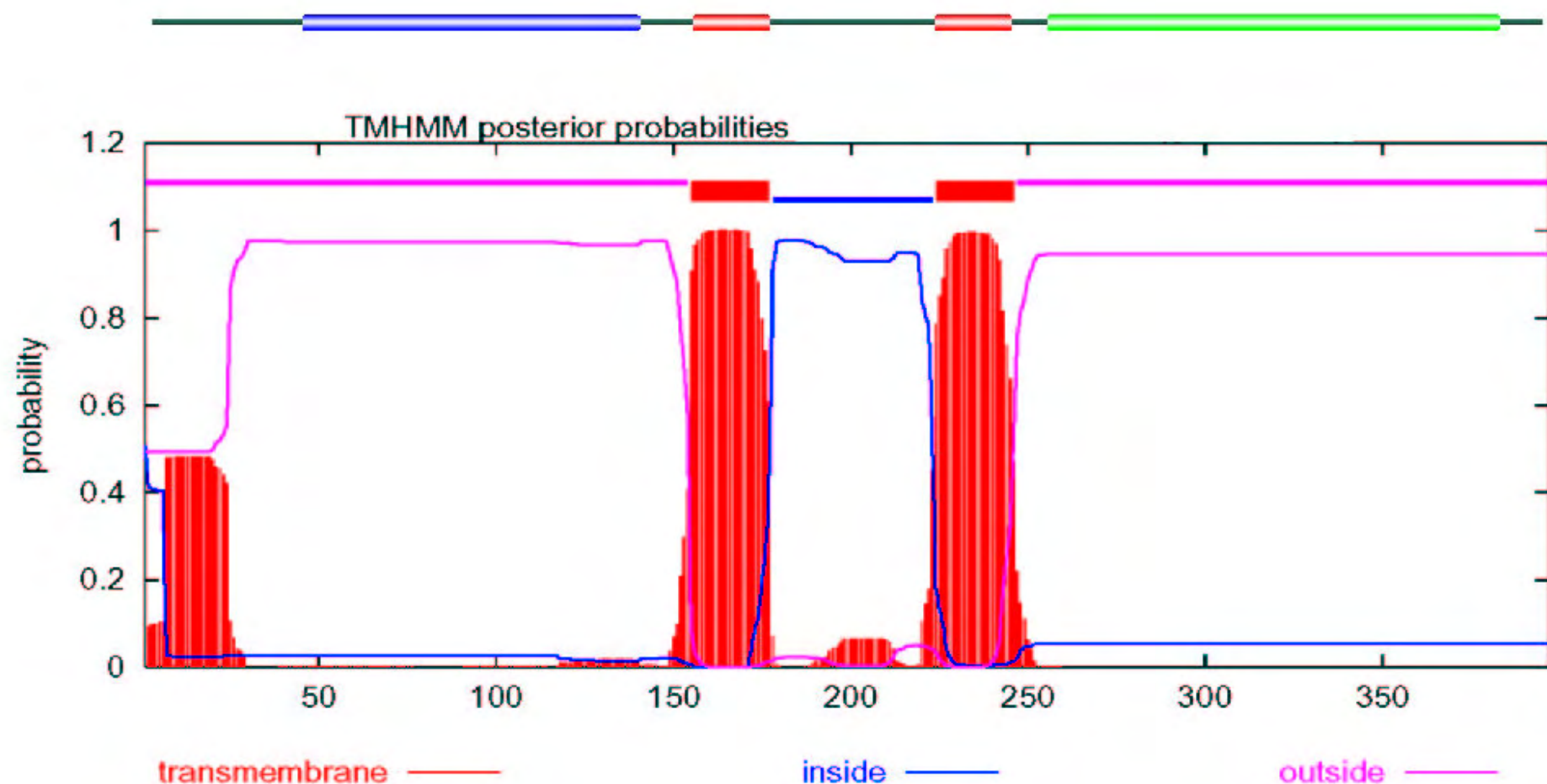

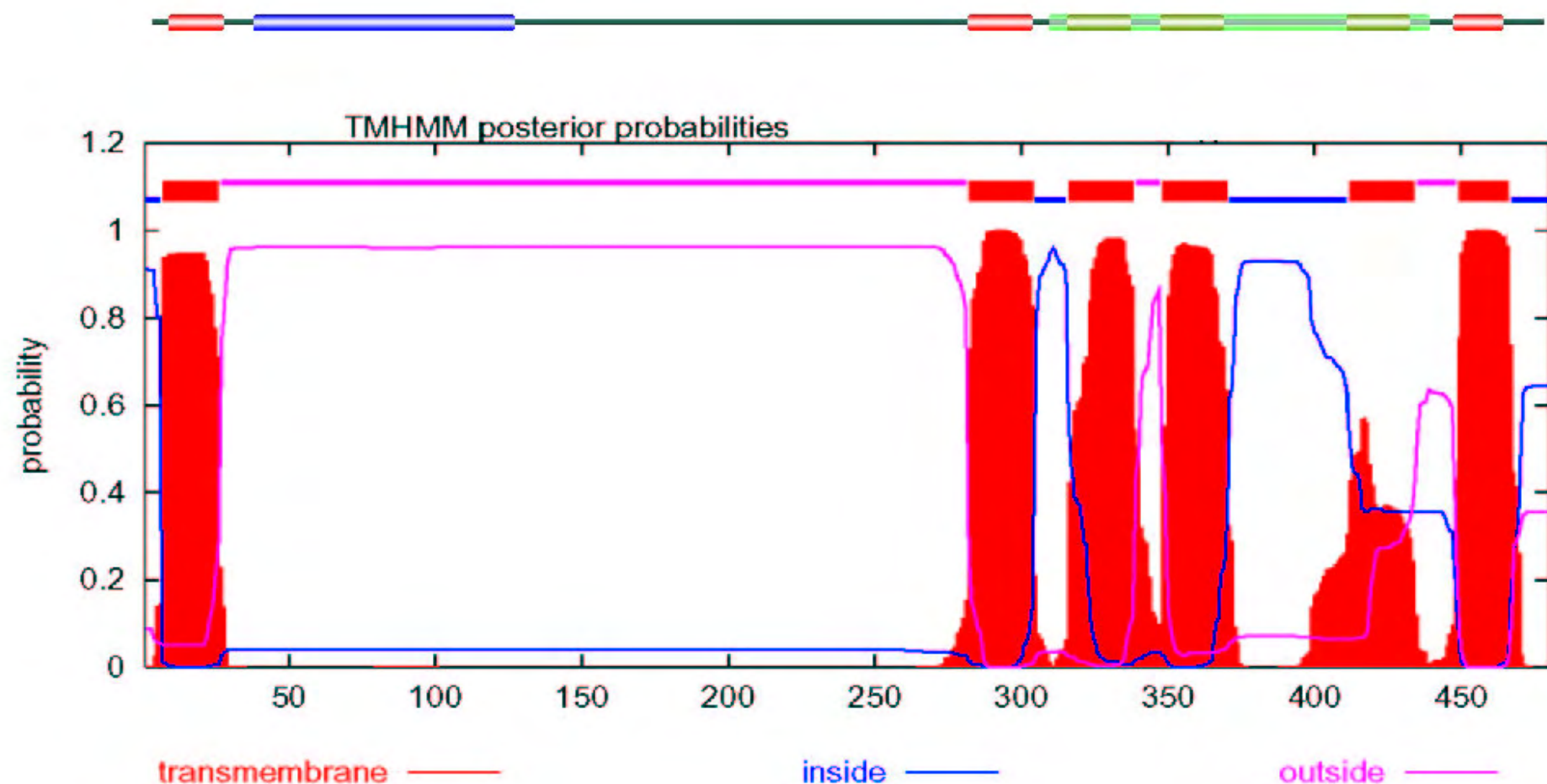

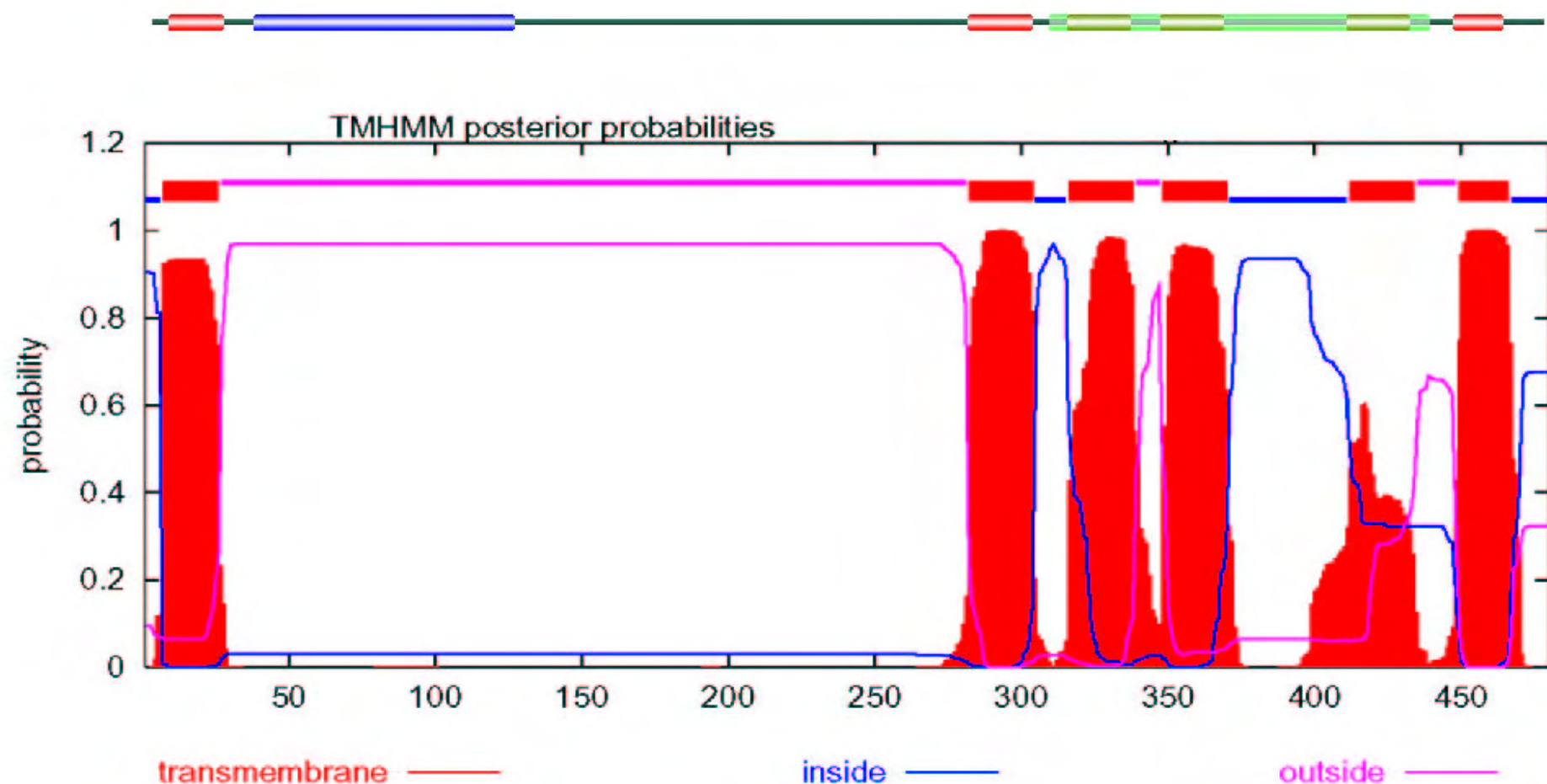

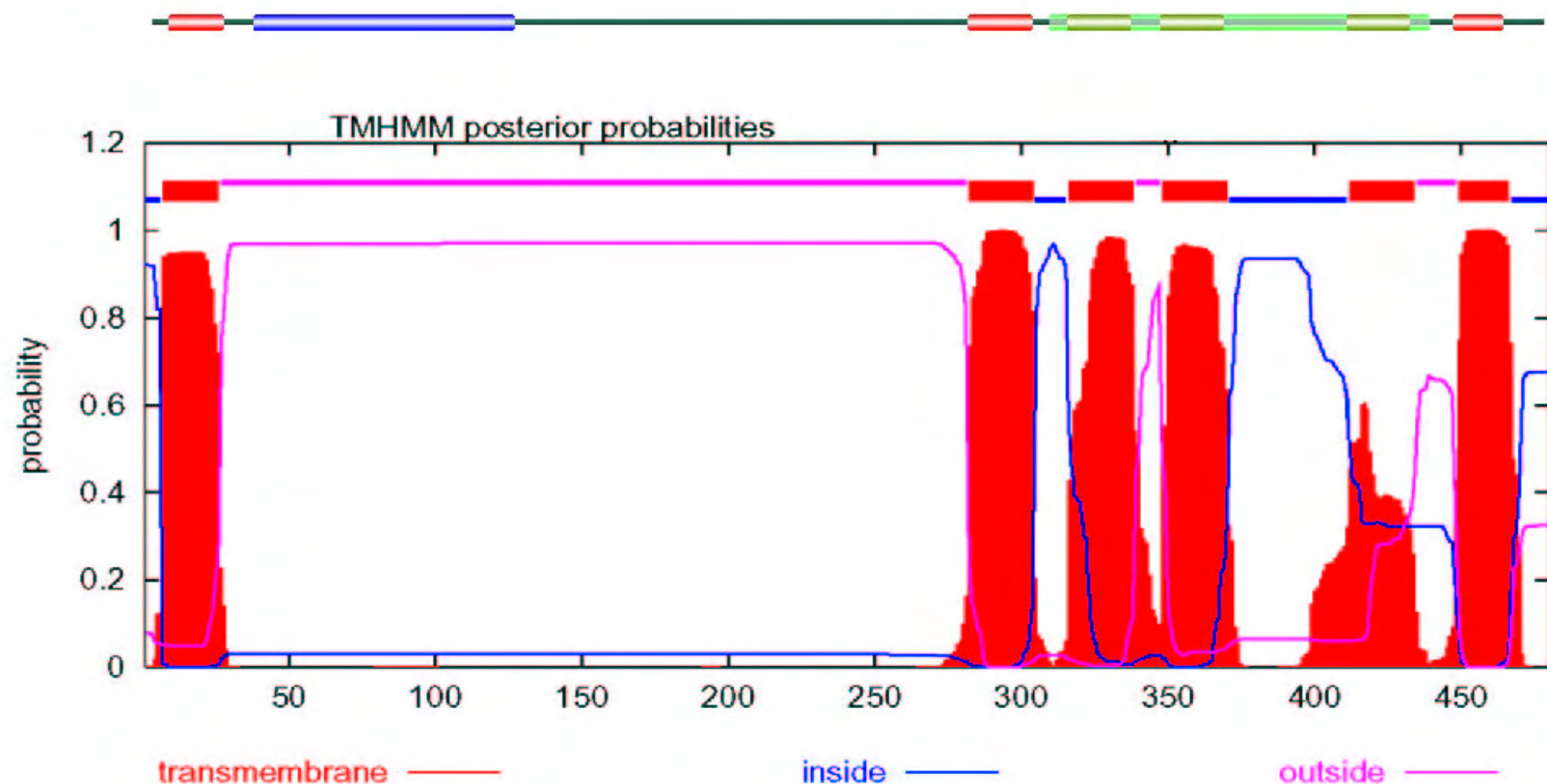

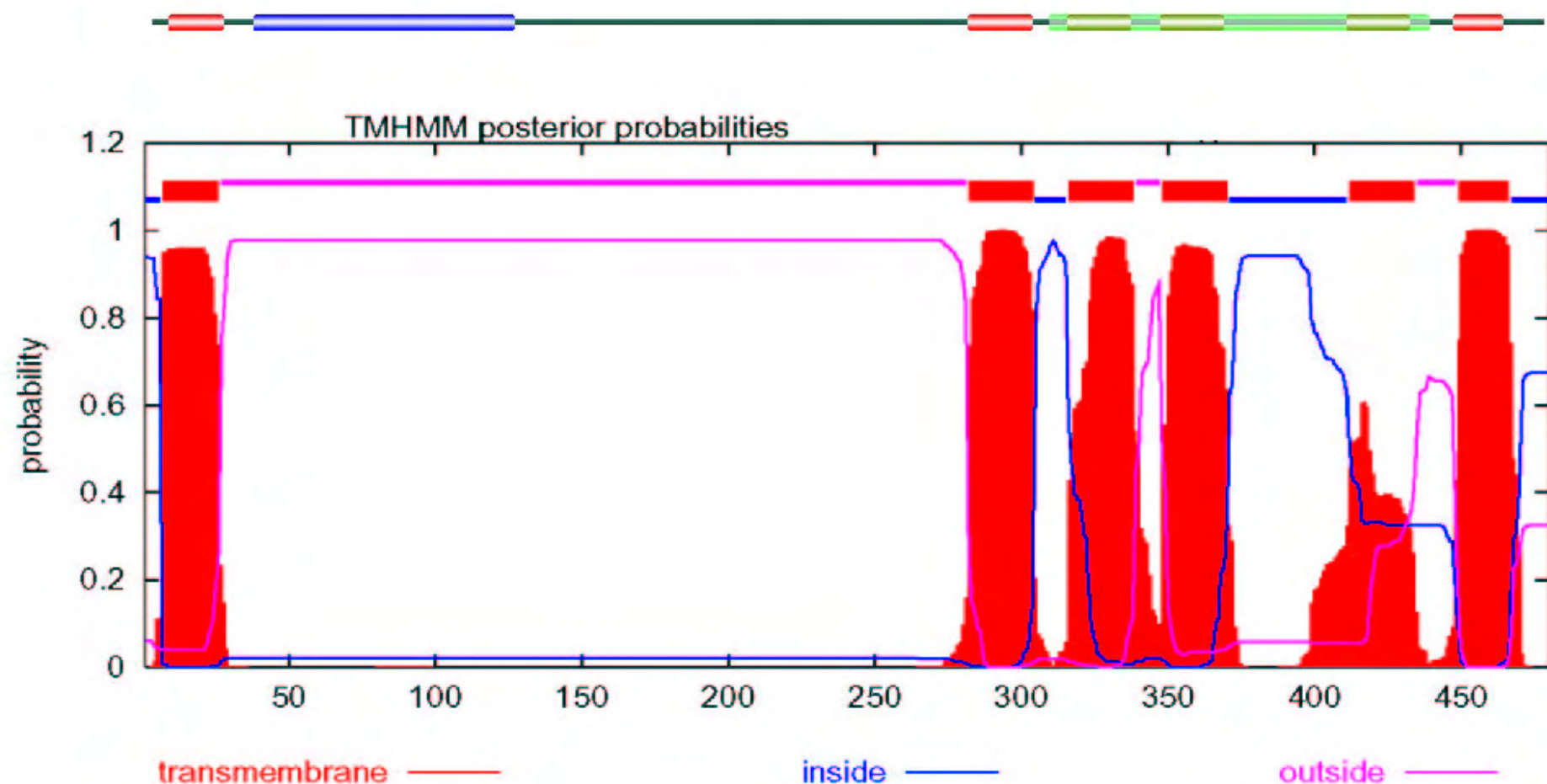

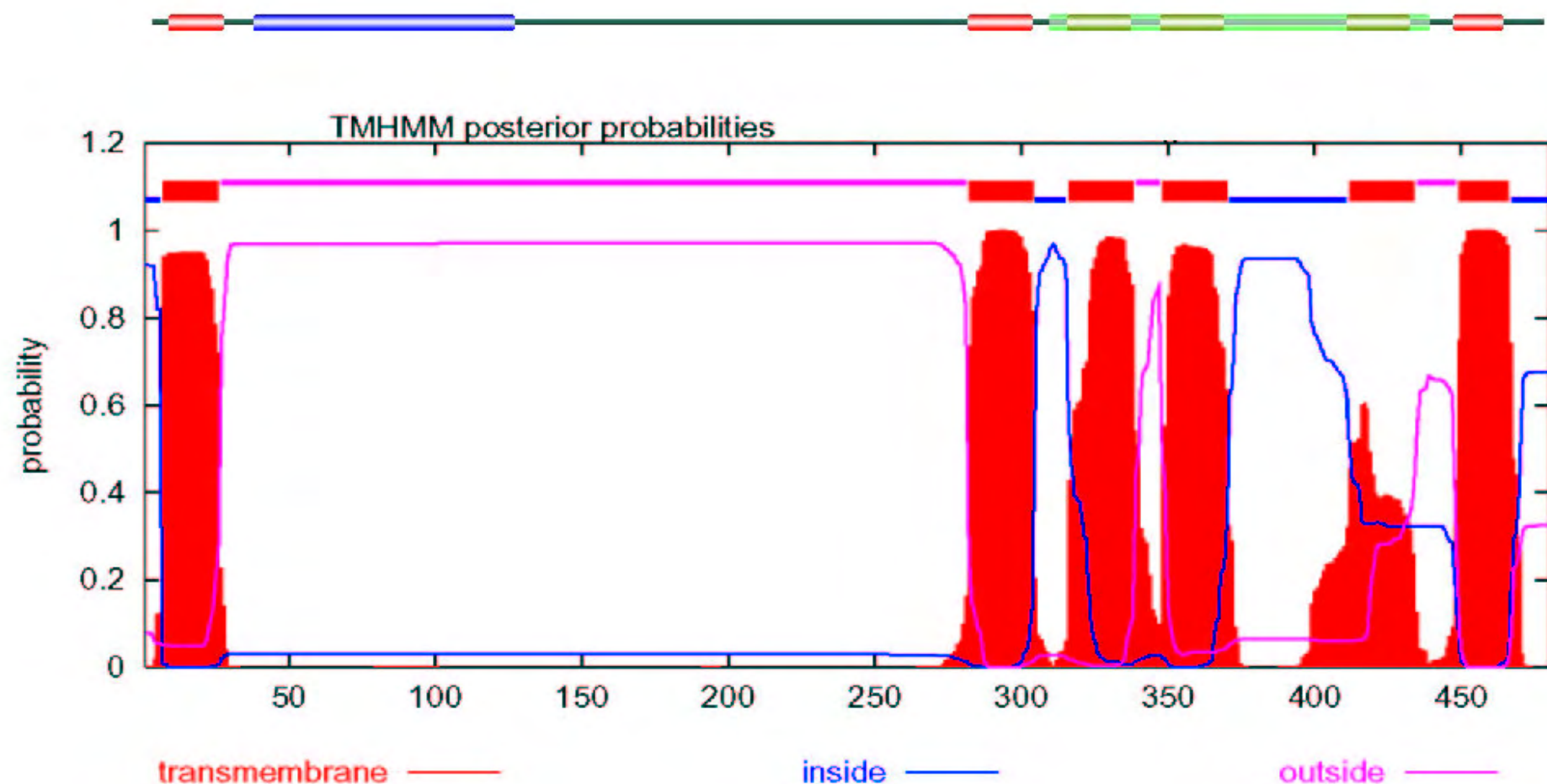

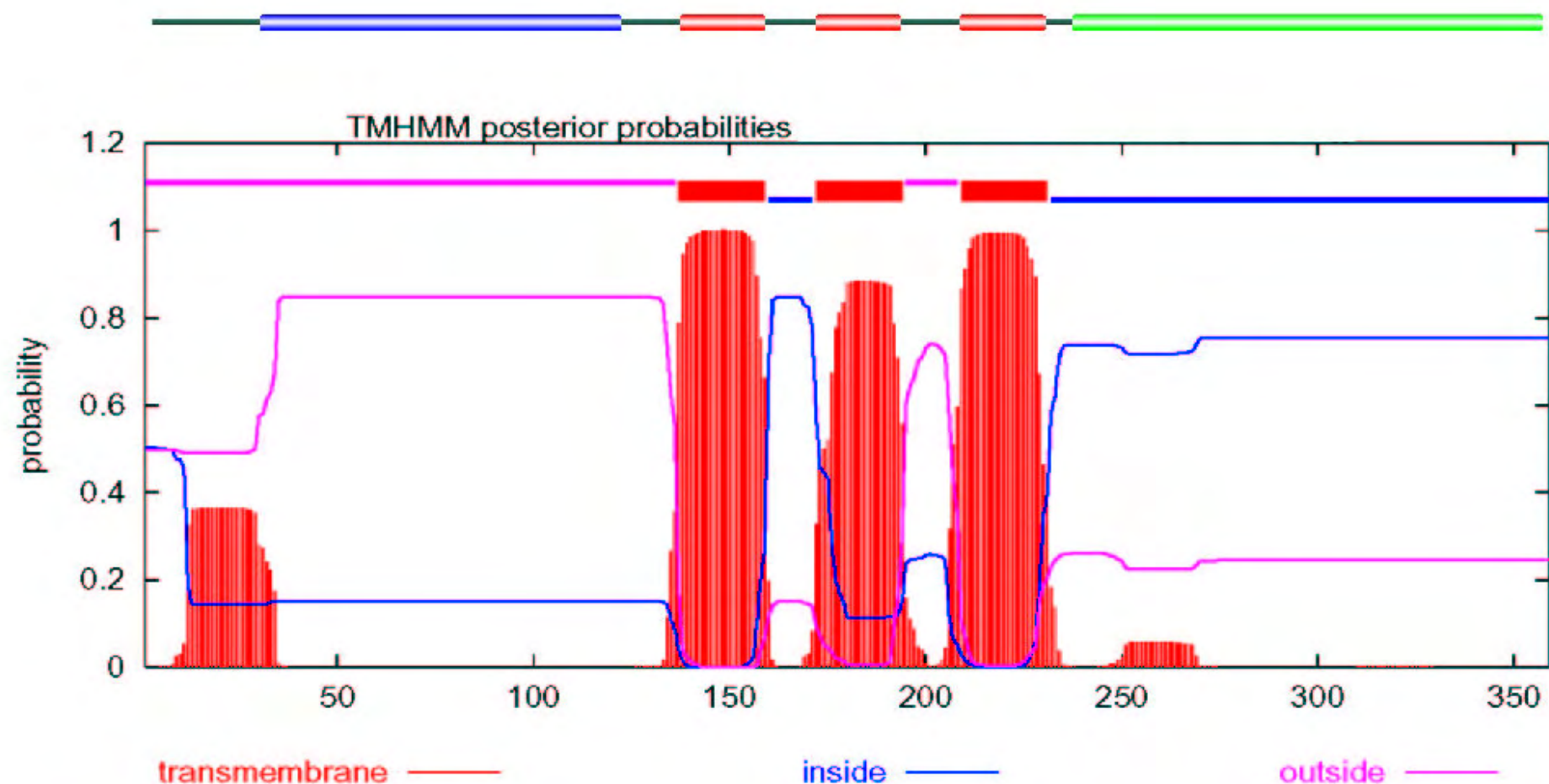

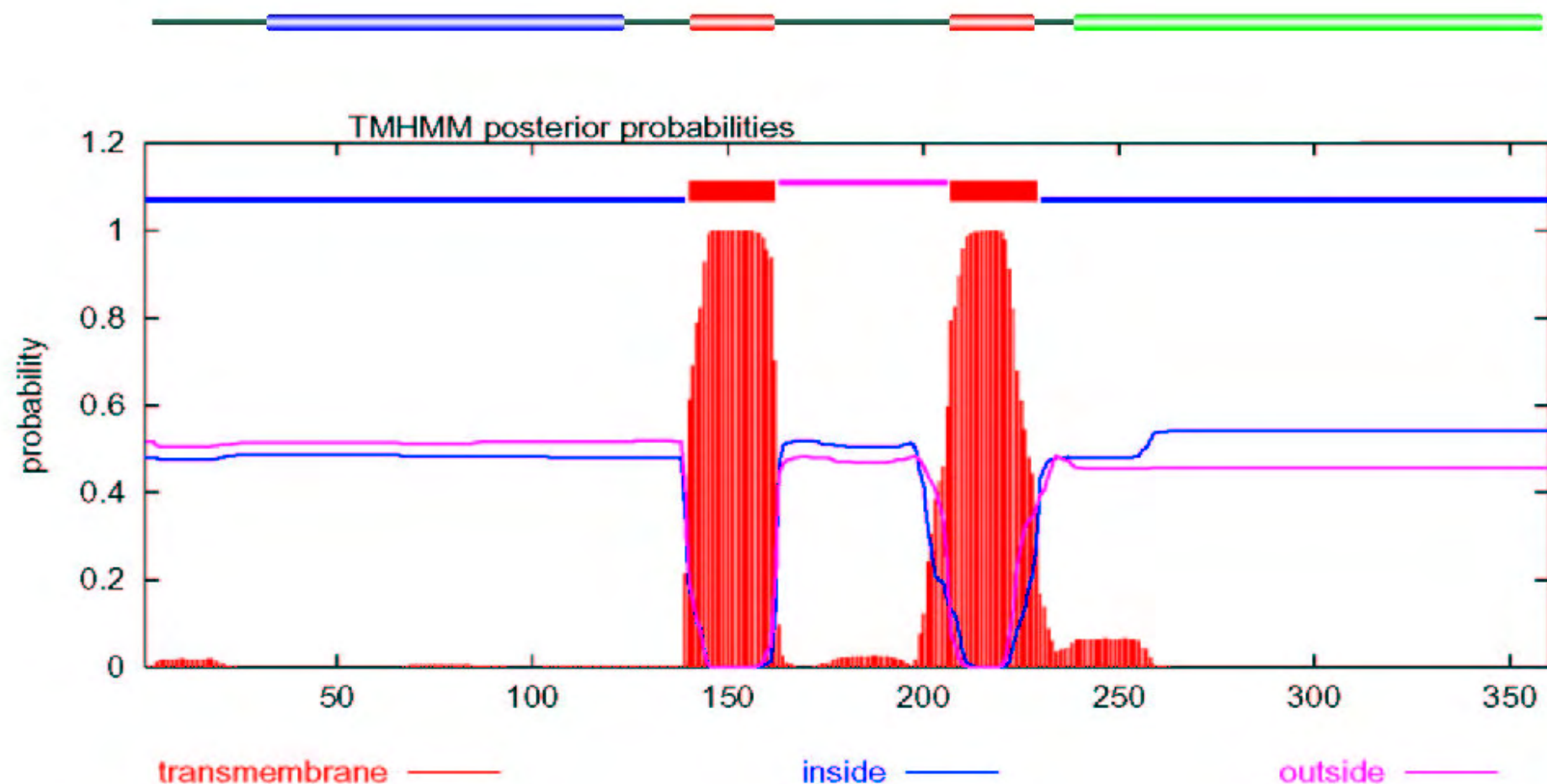

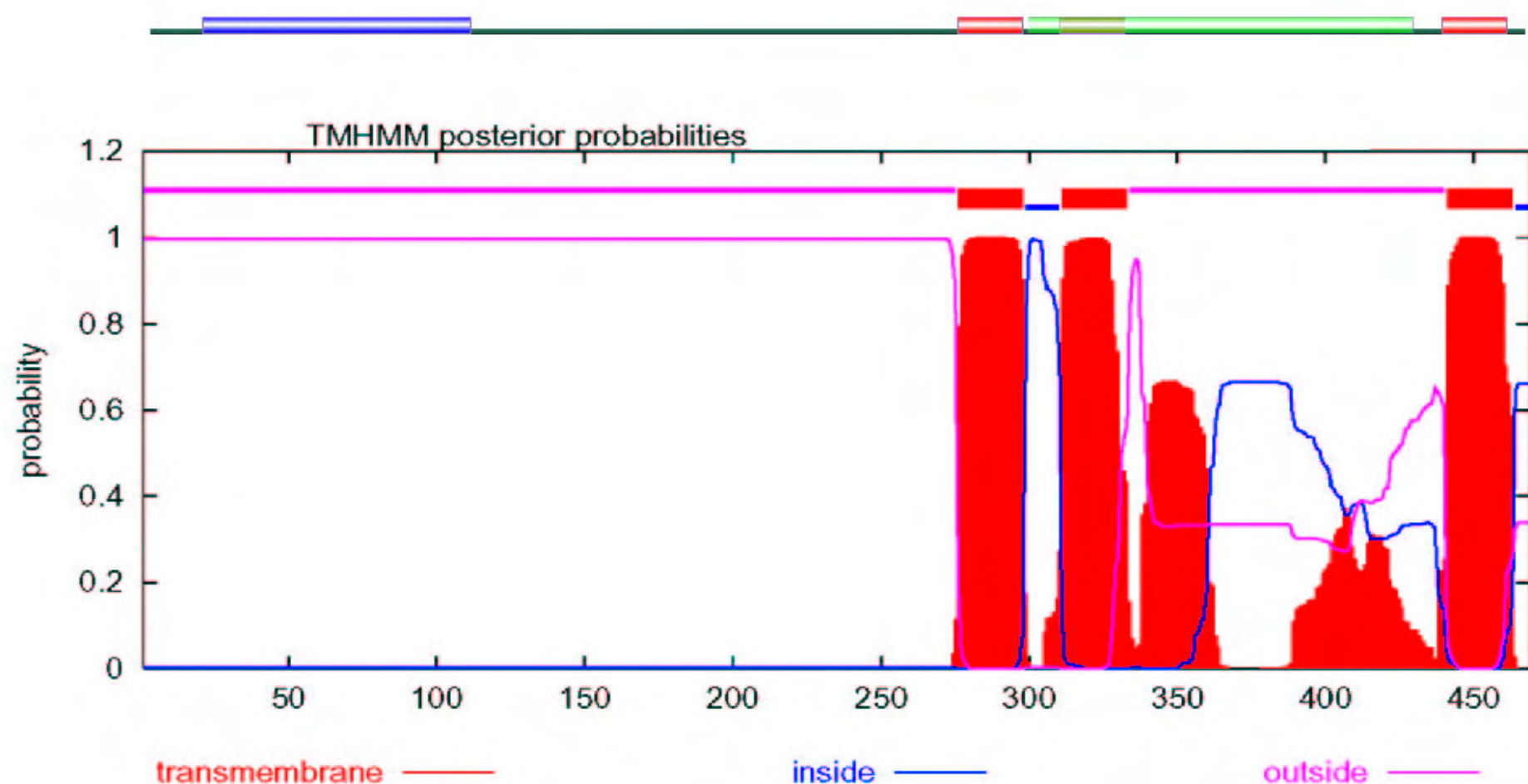

64

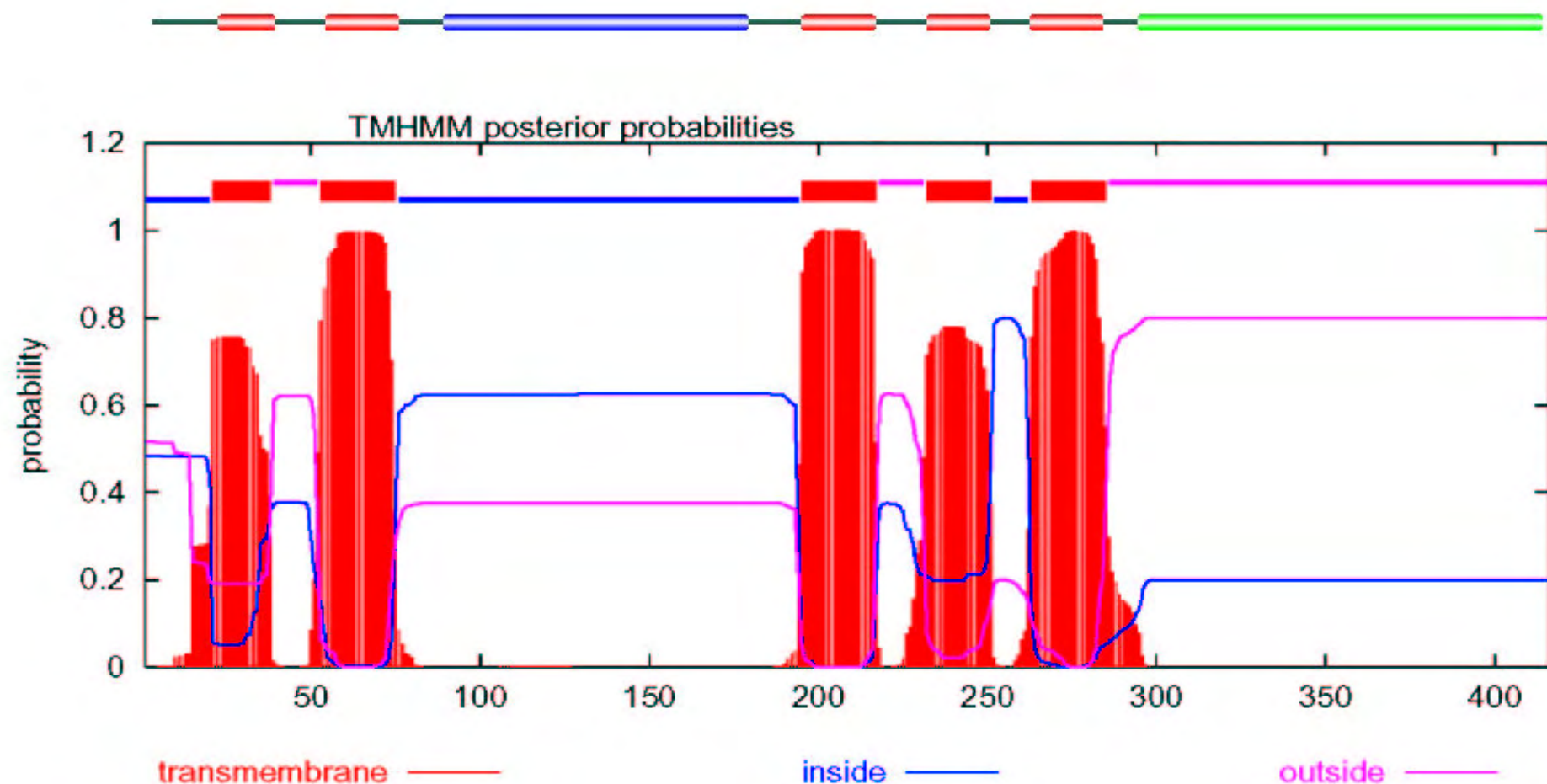

65

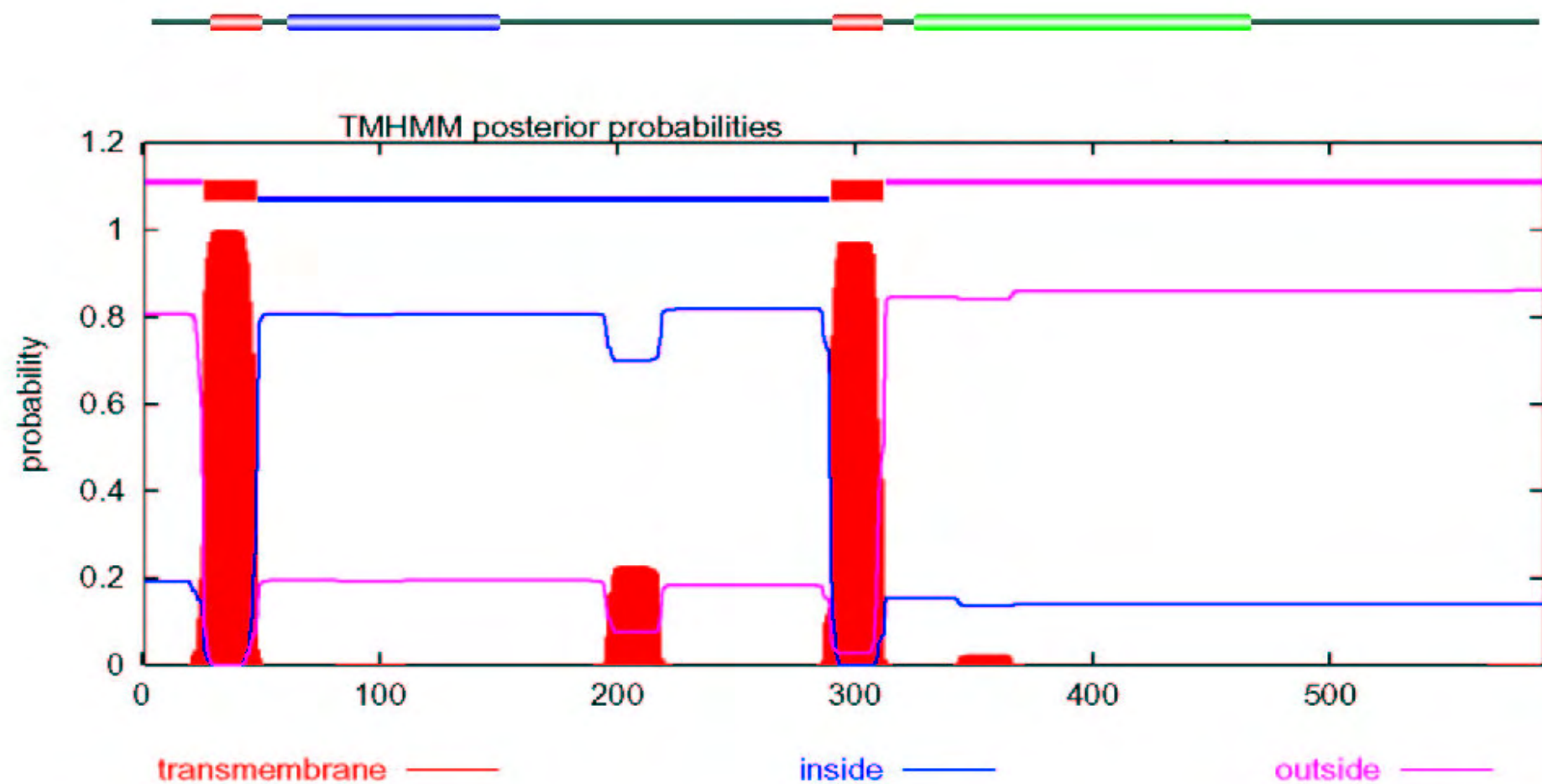

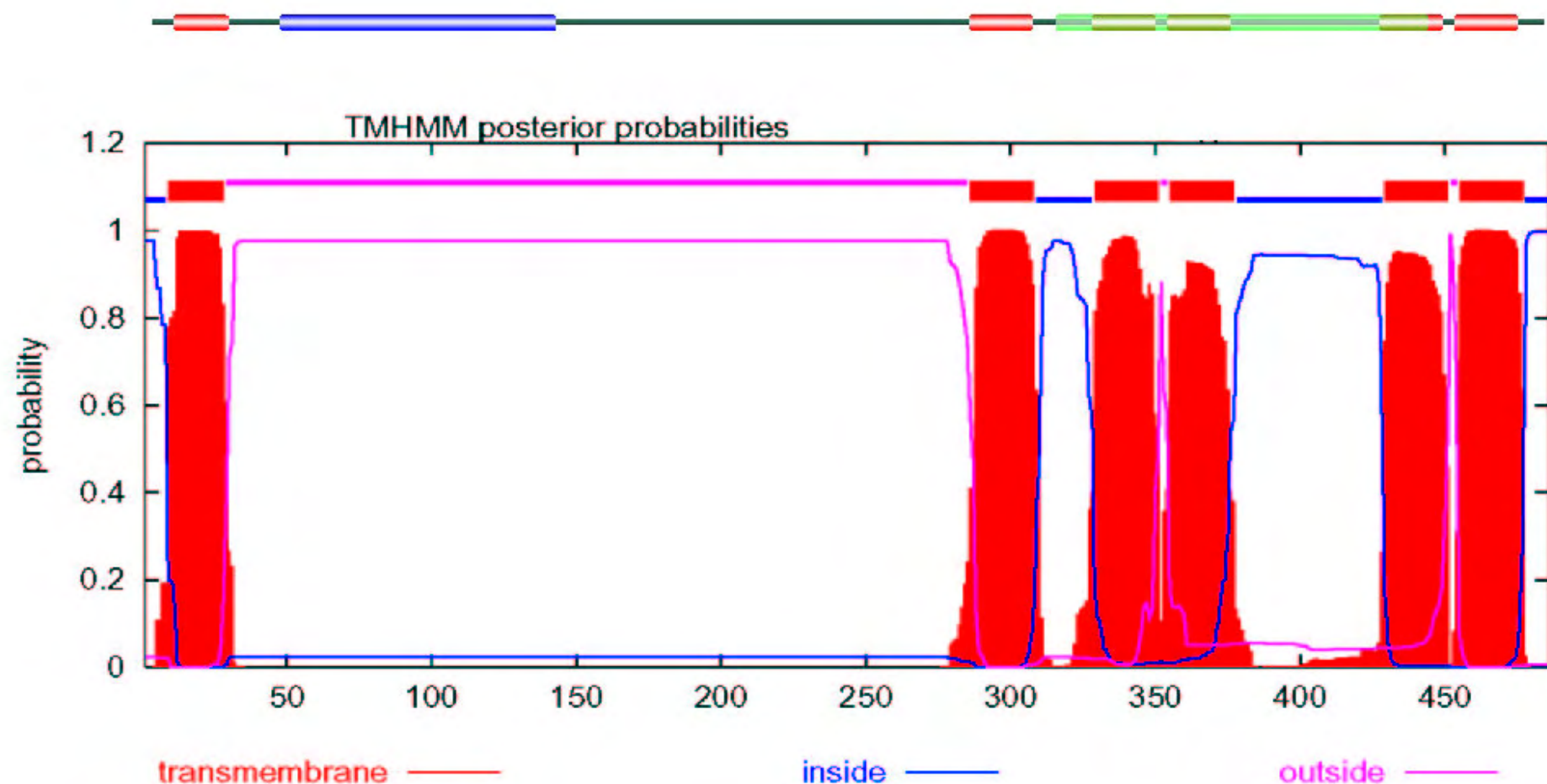

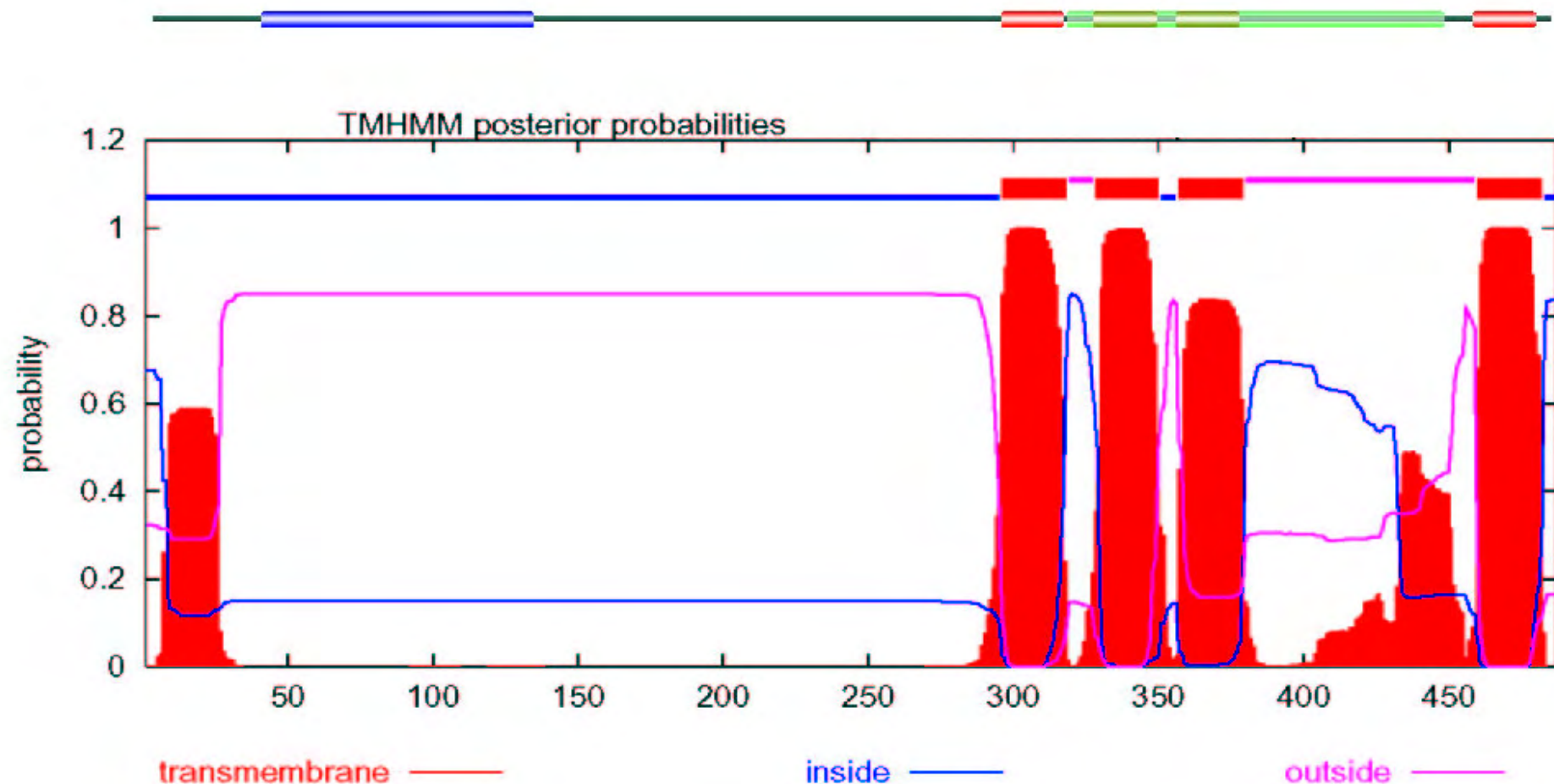

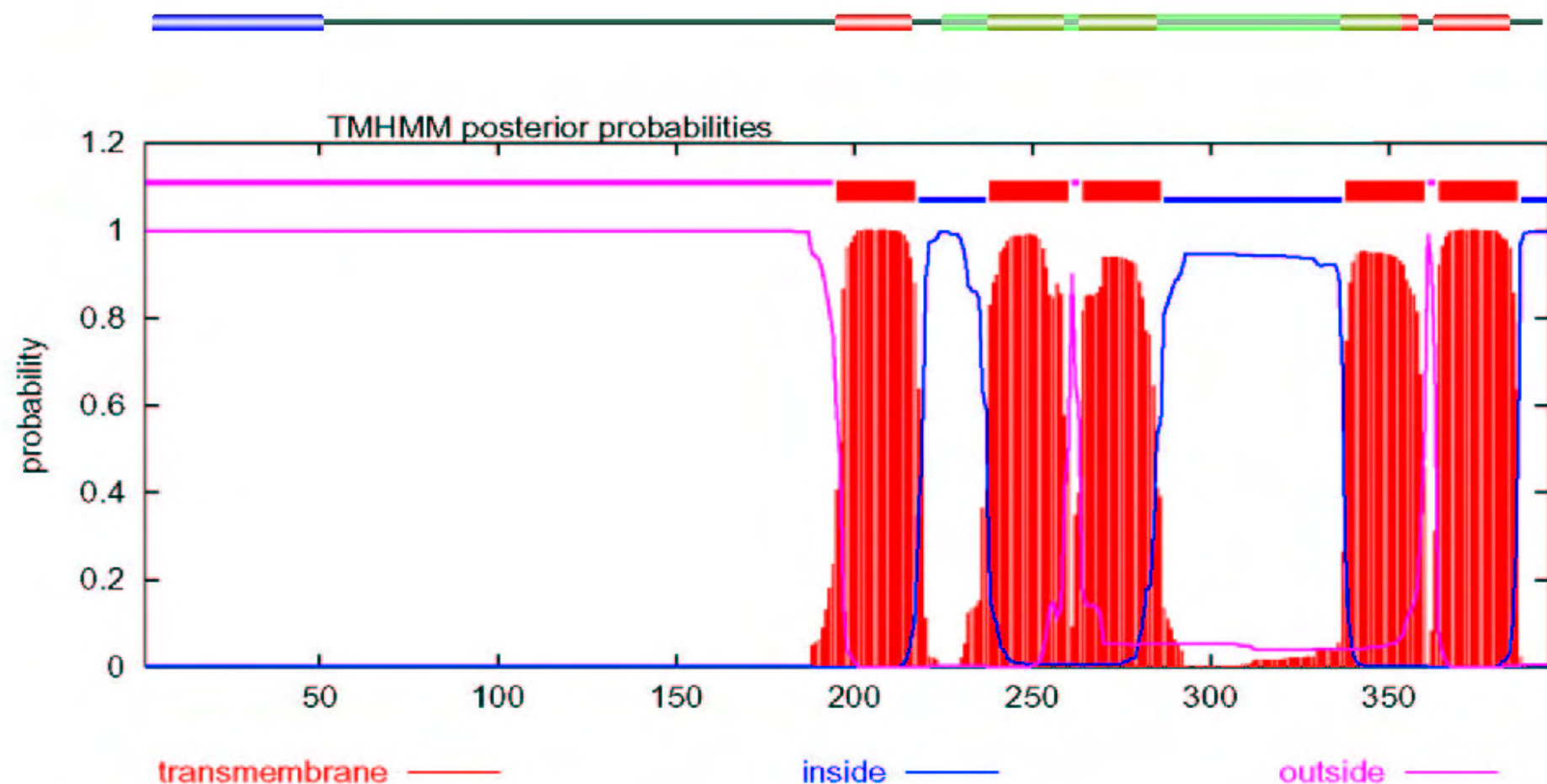

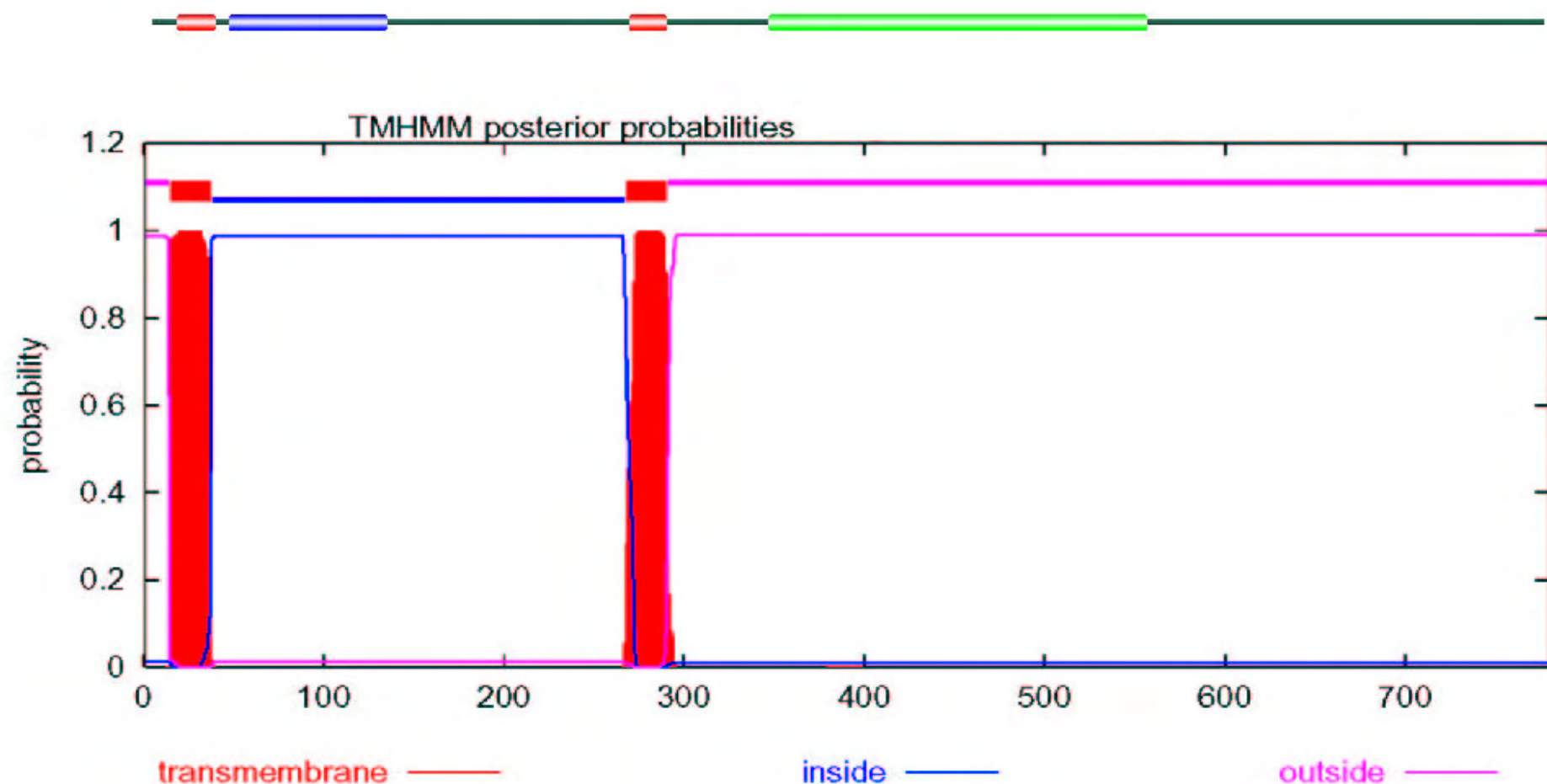

70

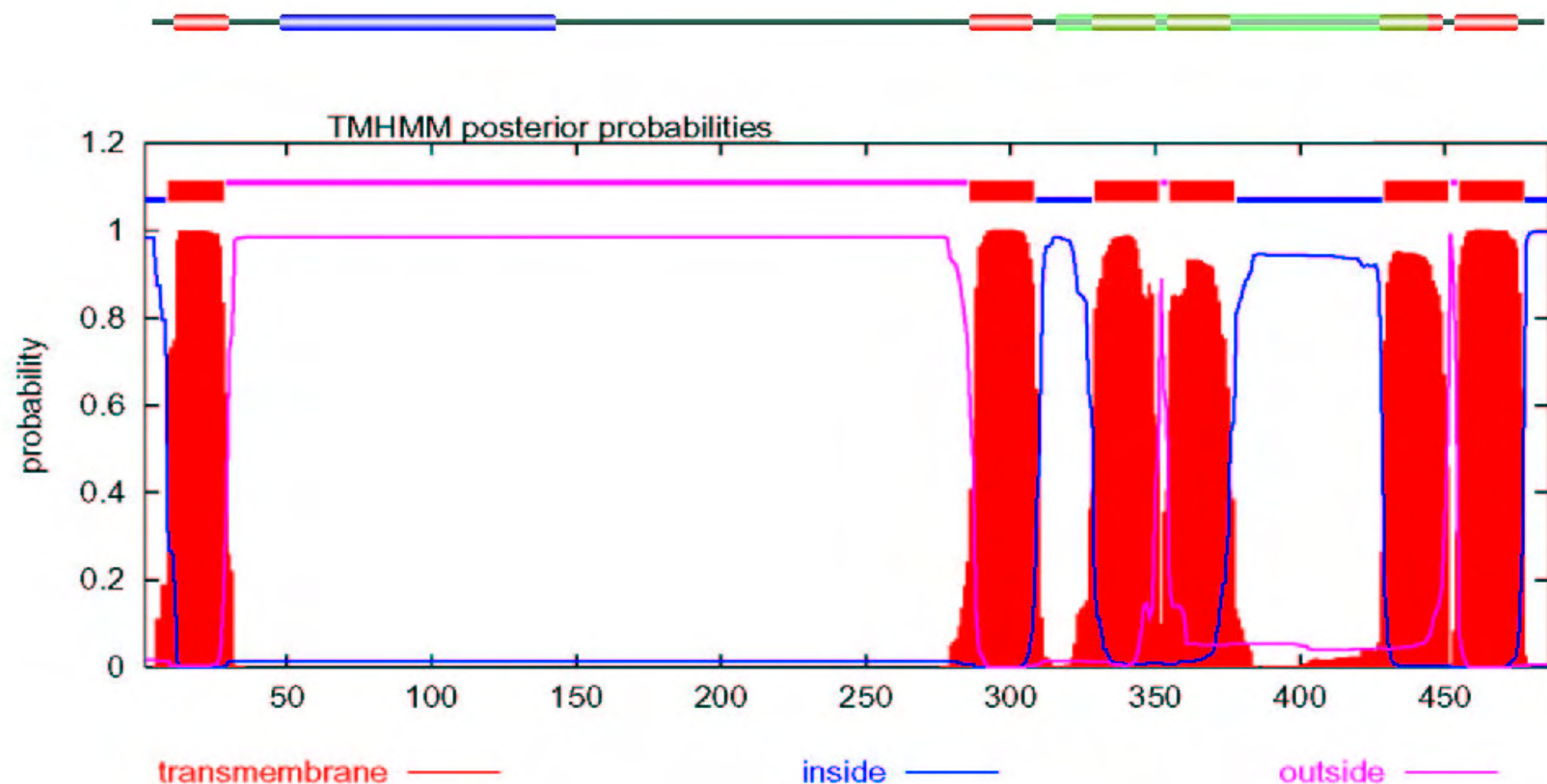

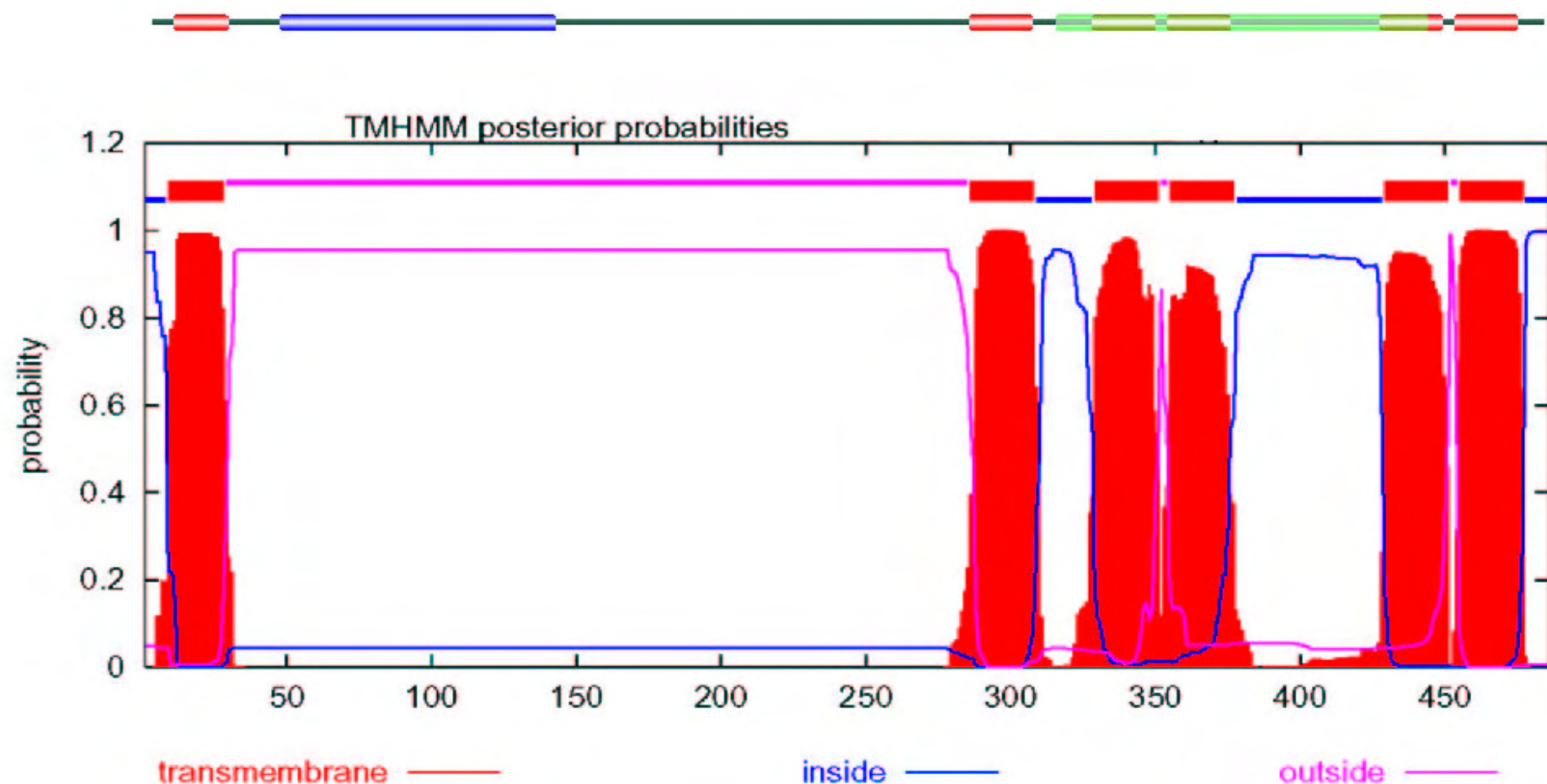

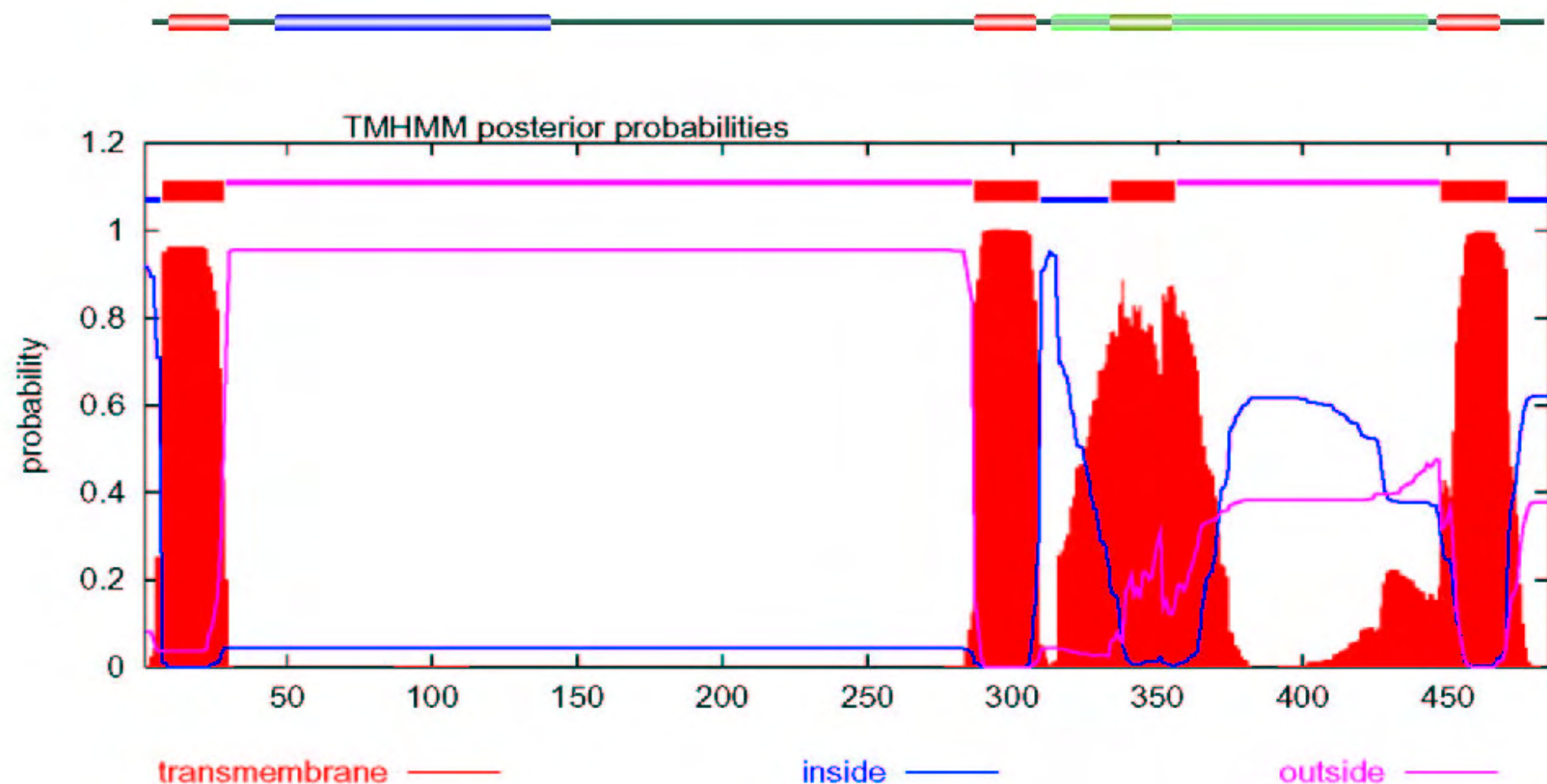

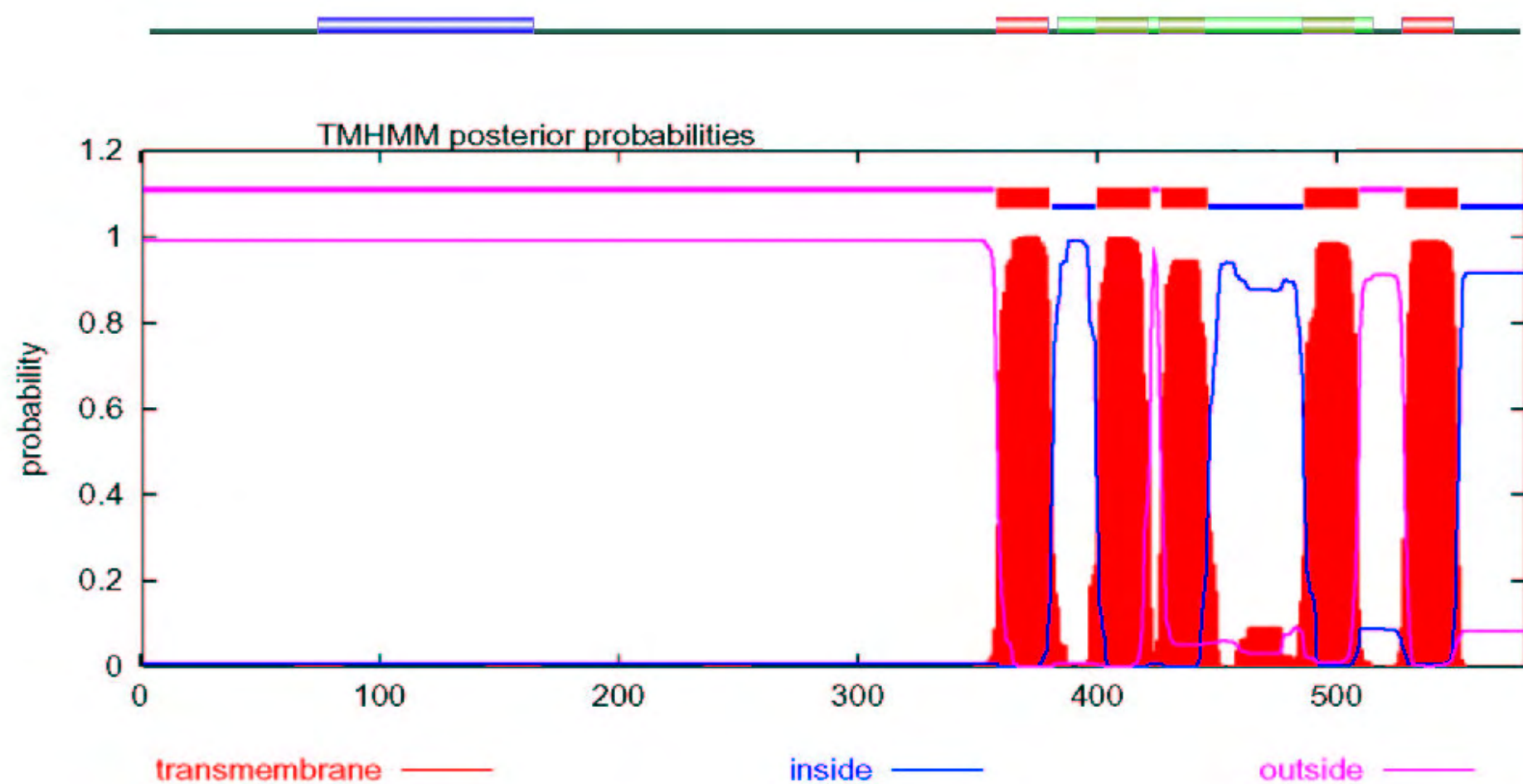

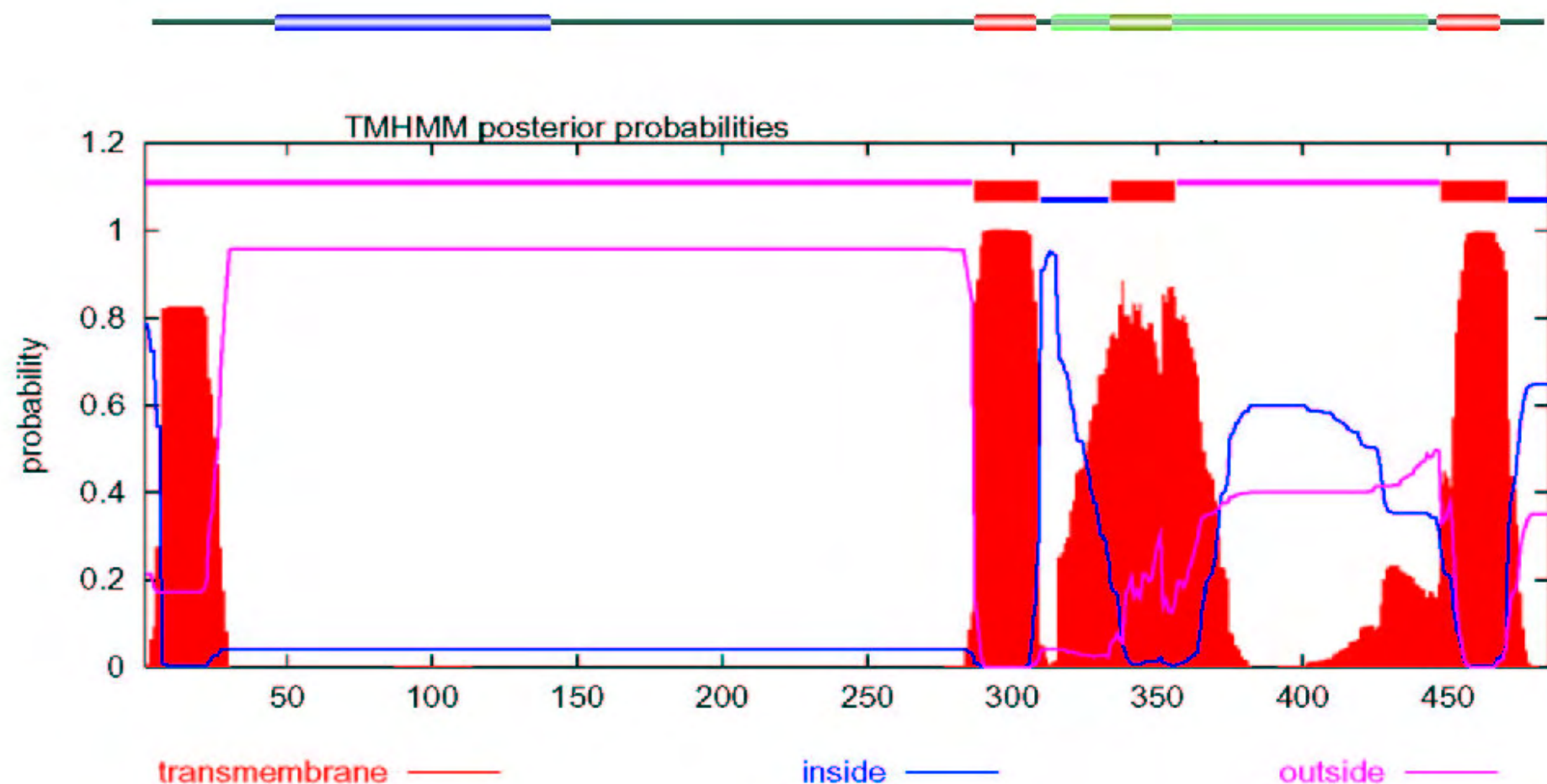

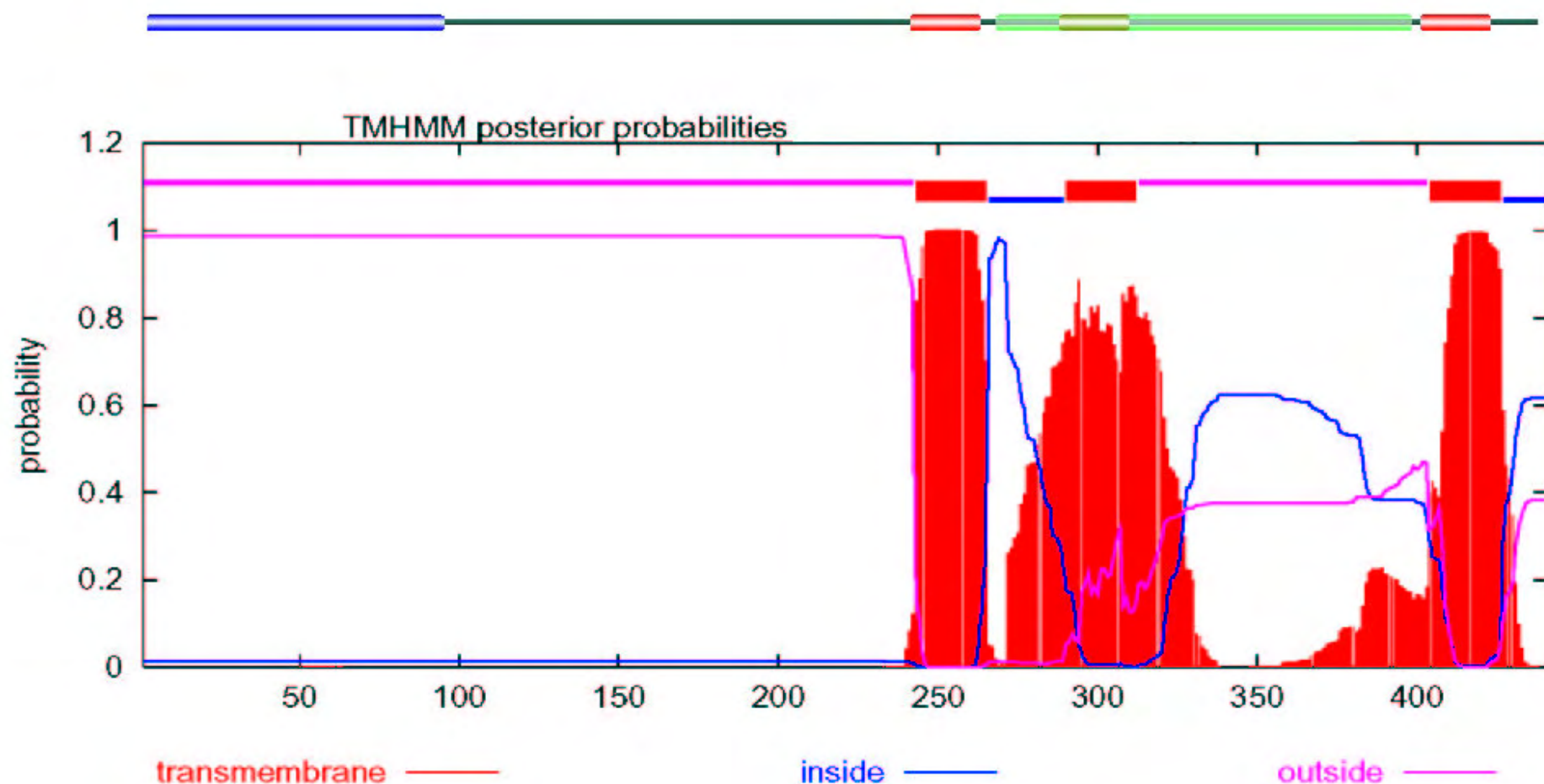

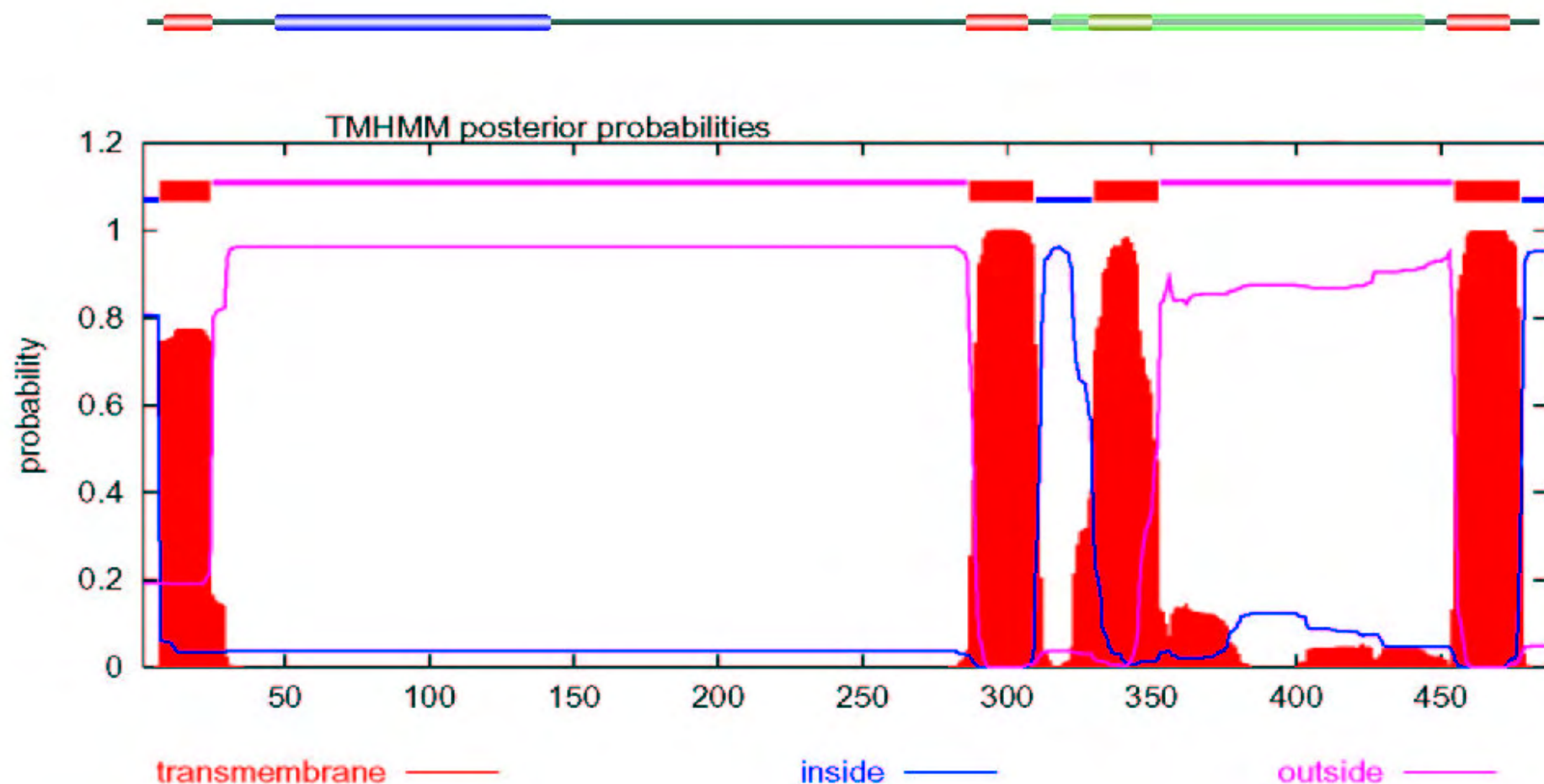

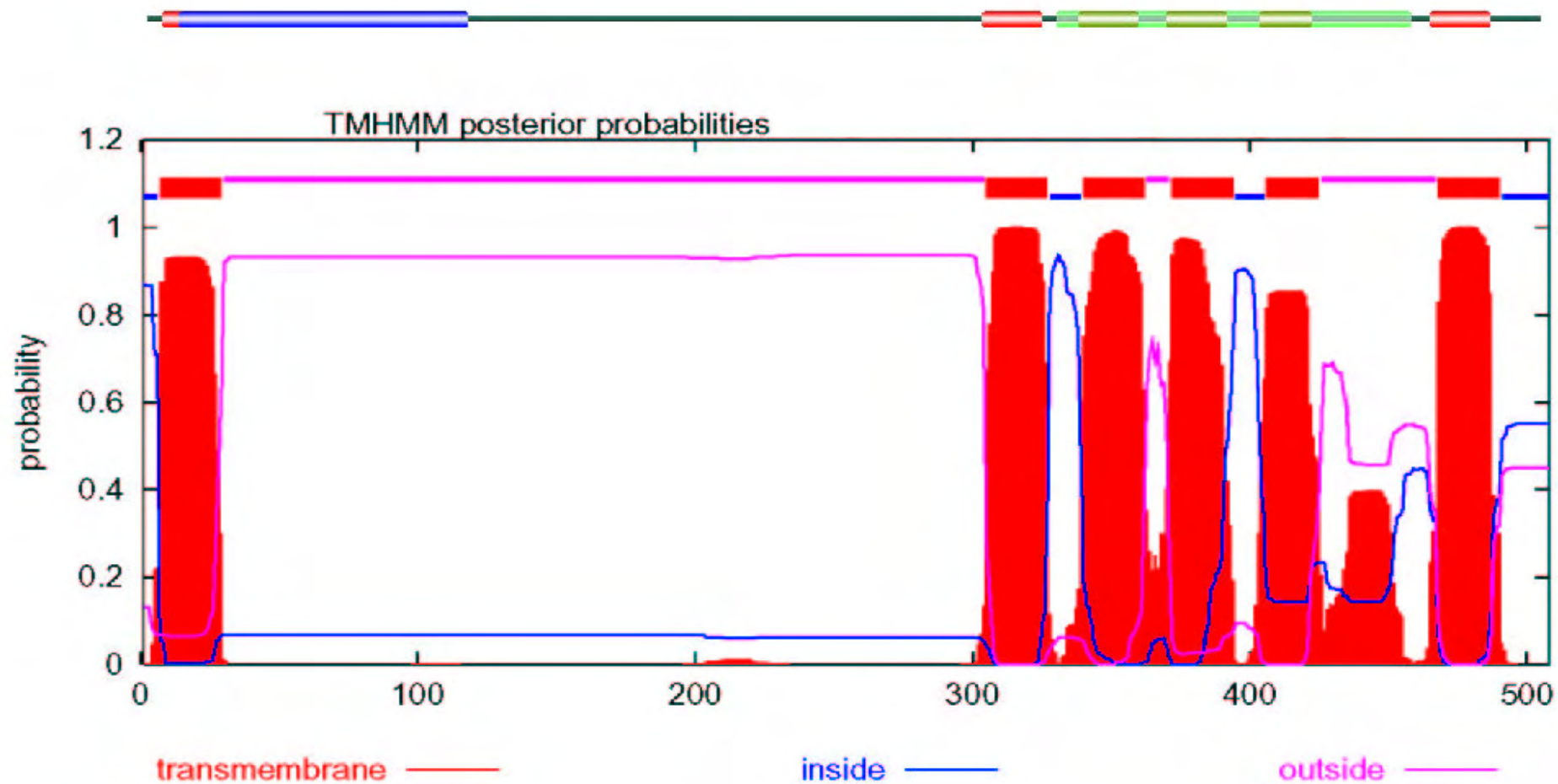

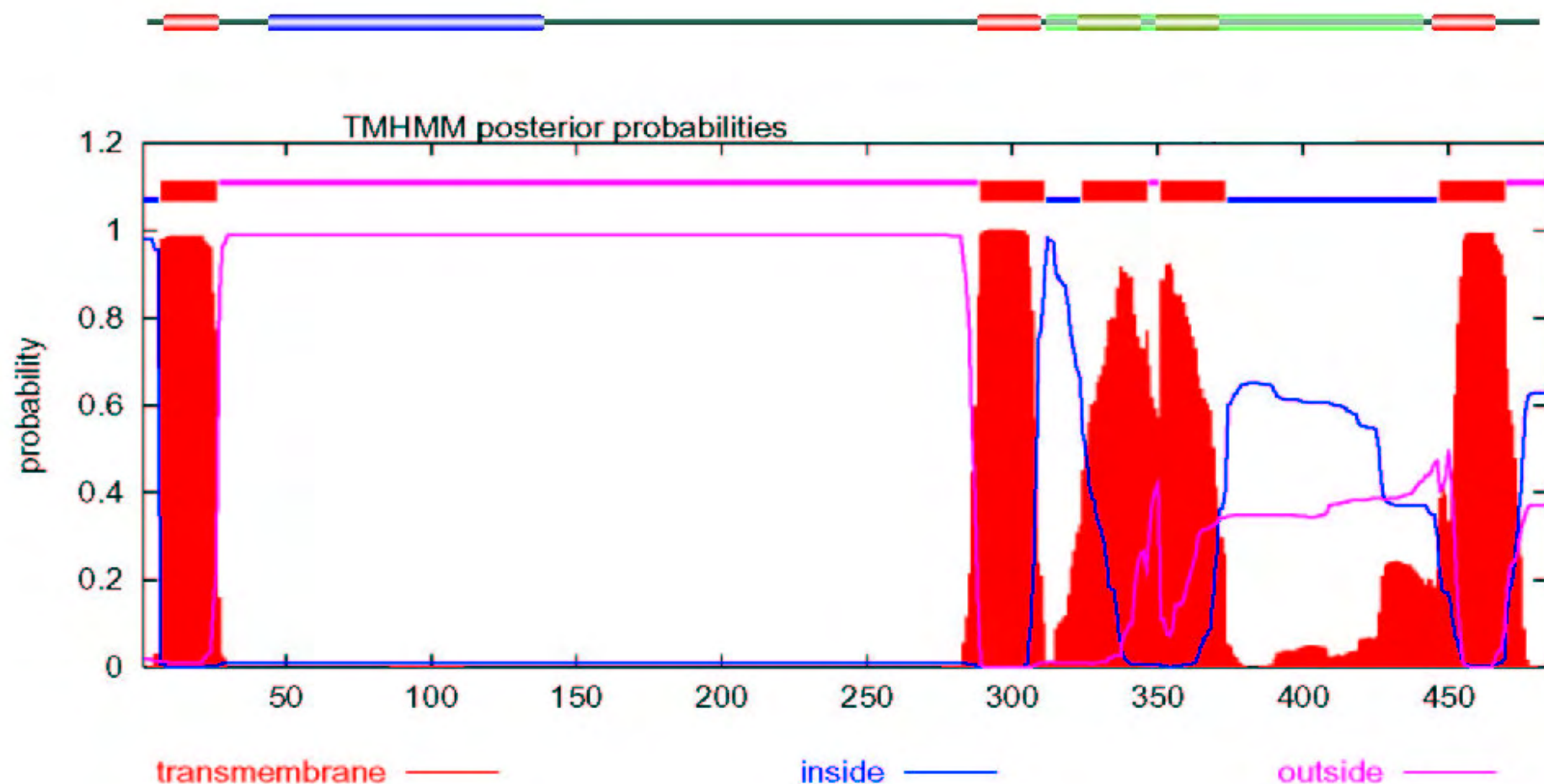

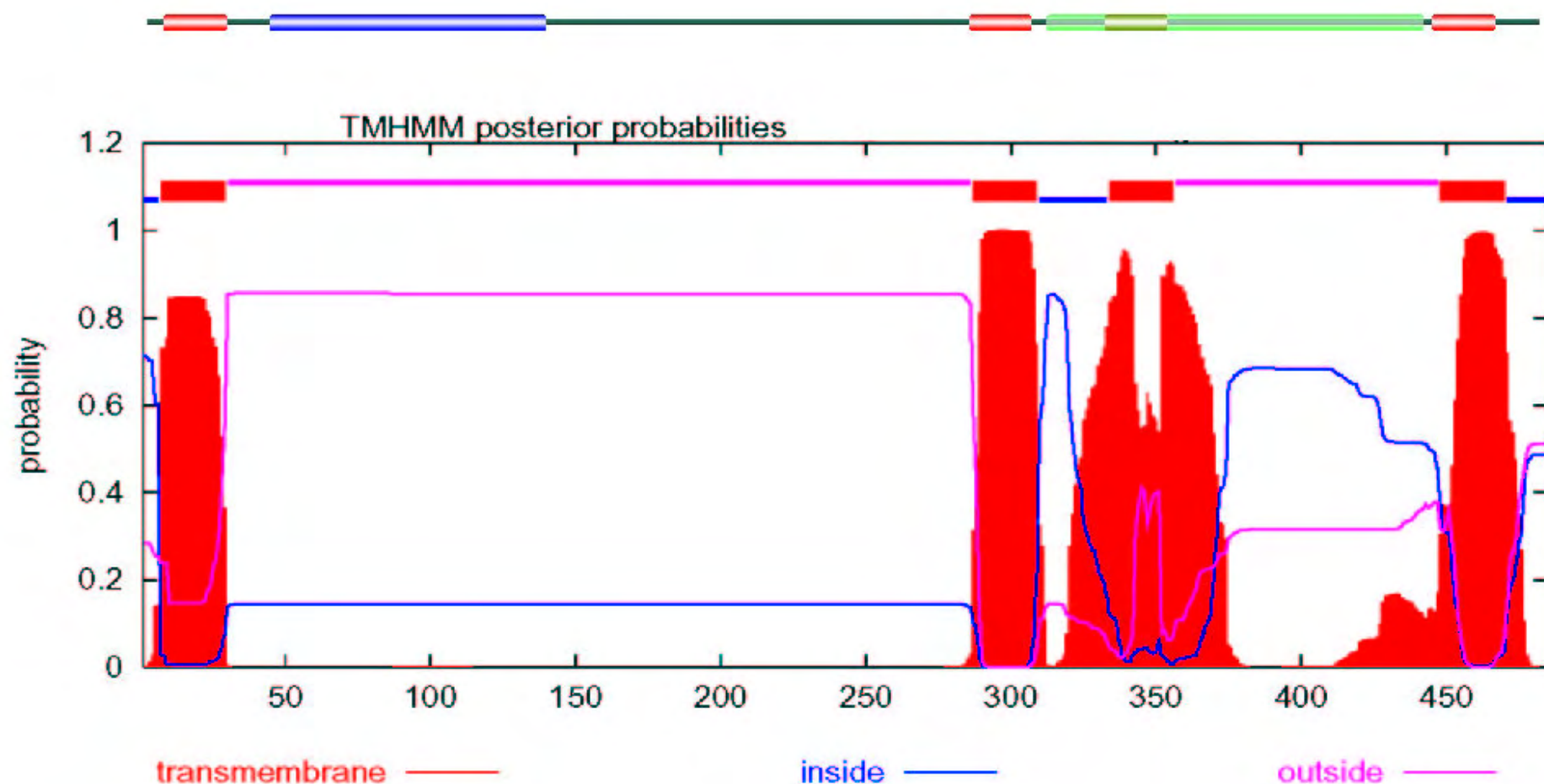

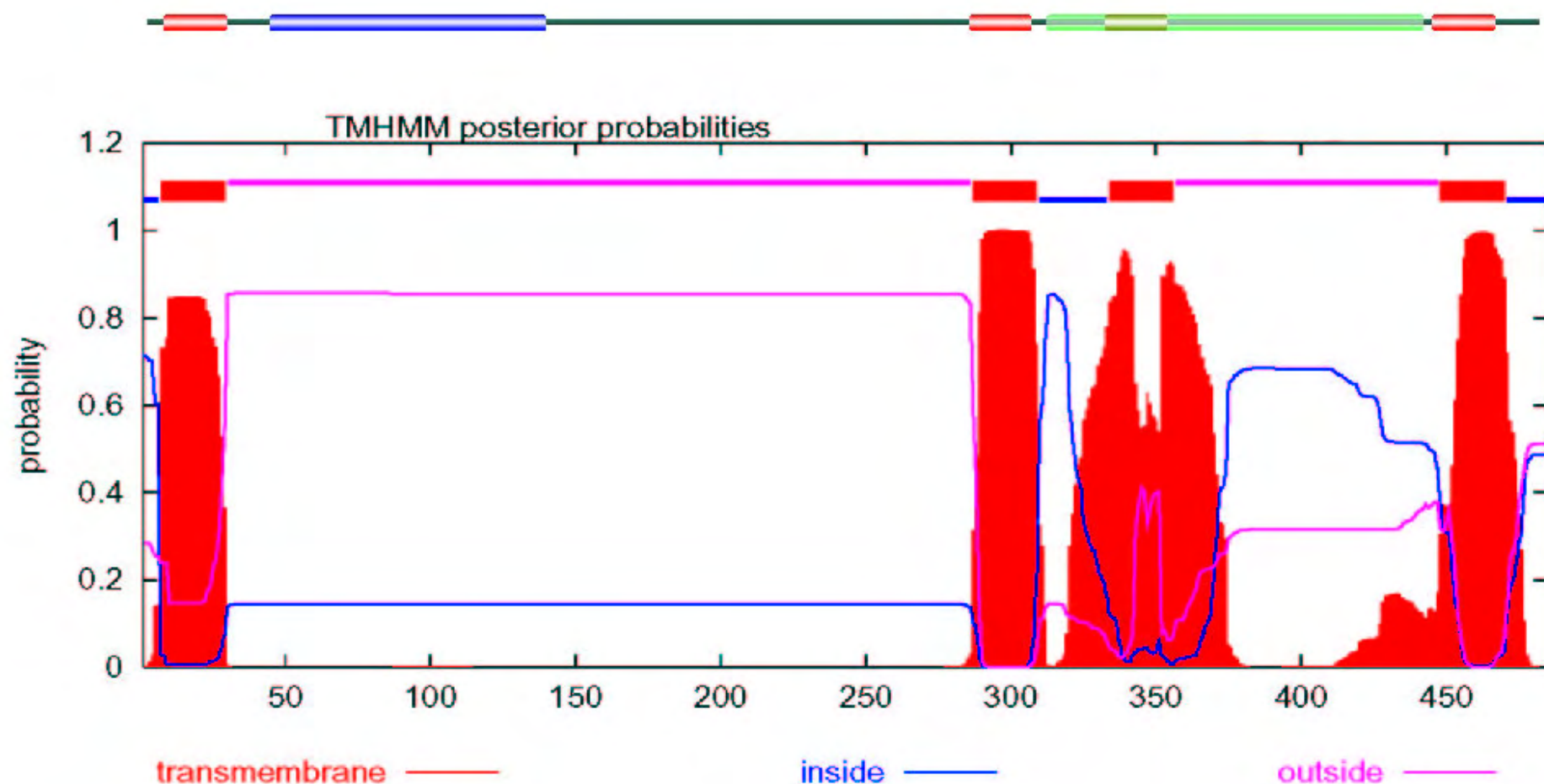

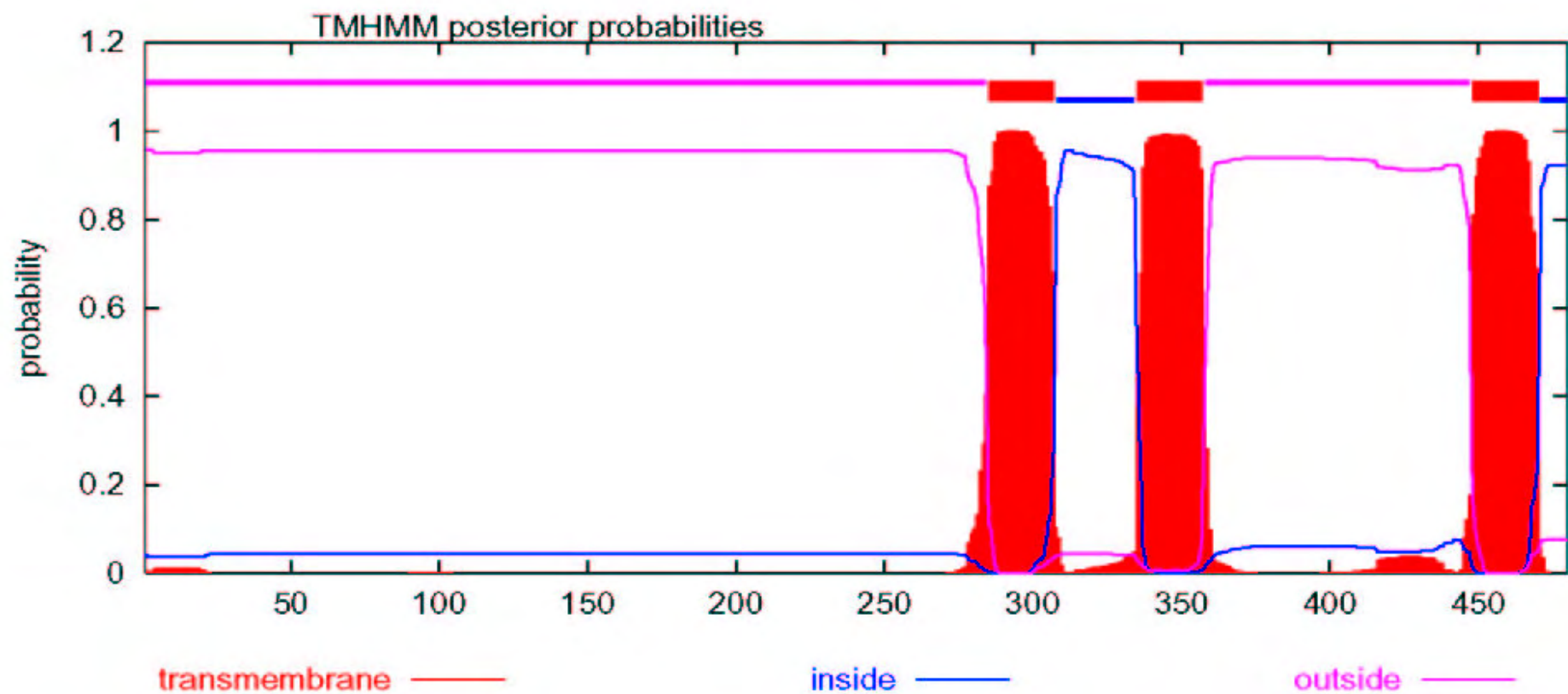

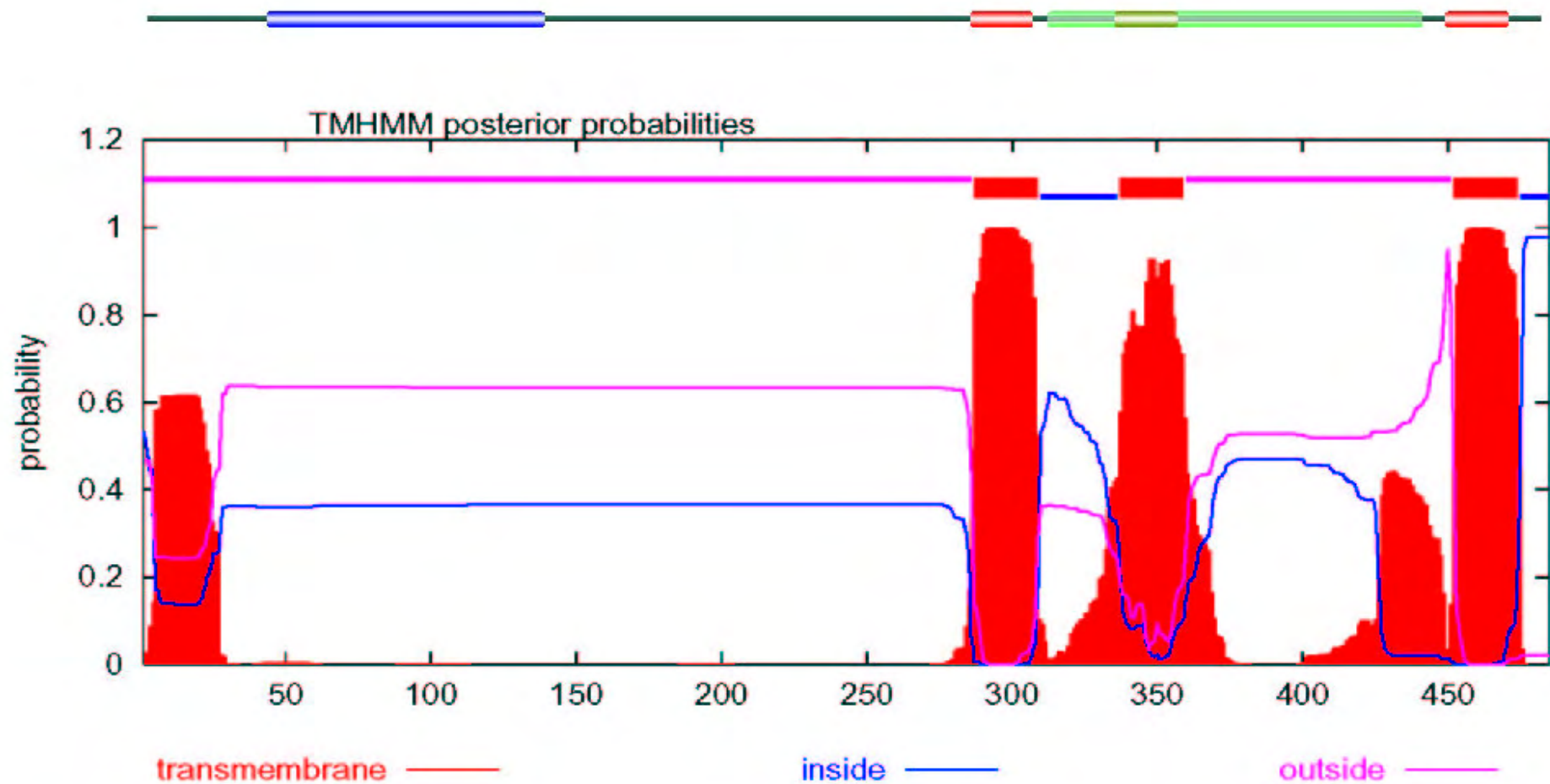

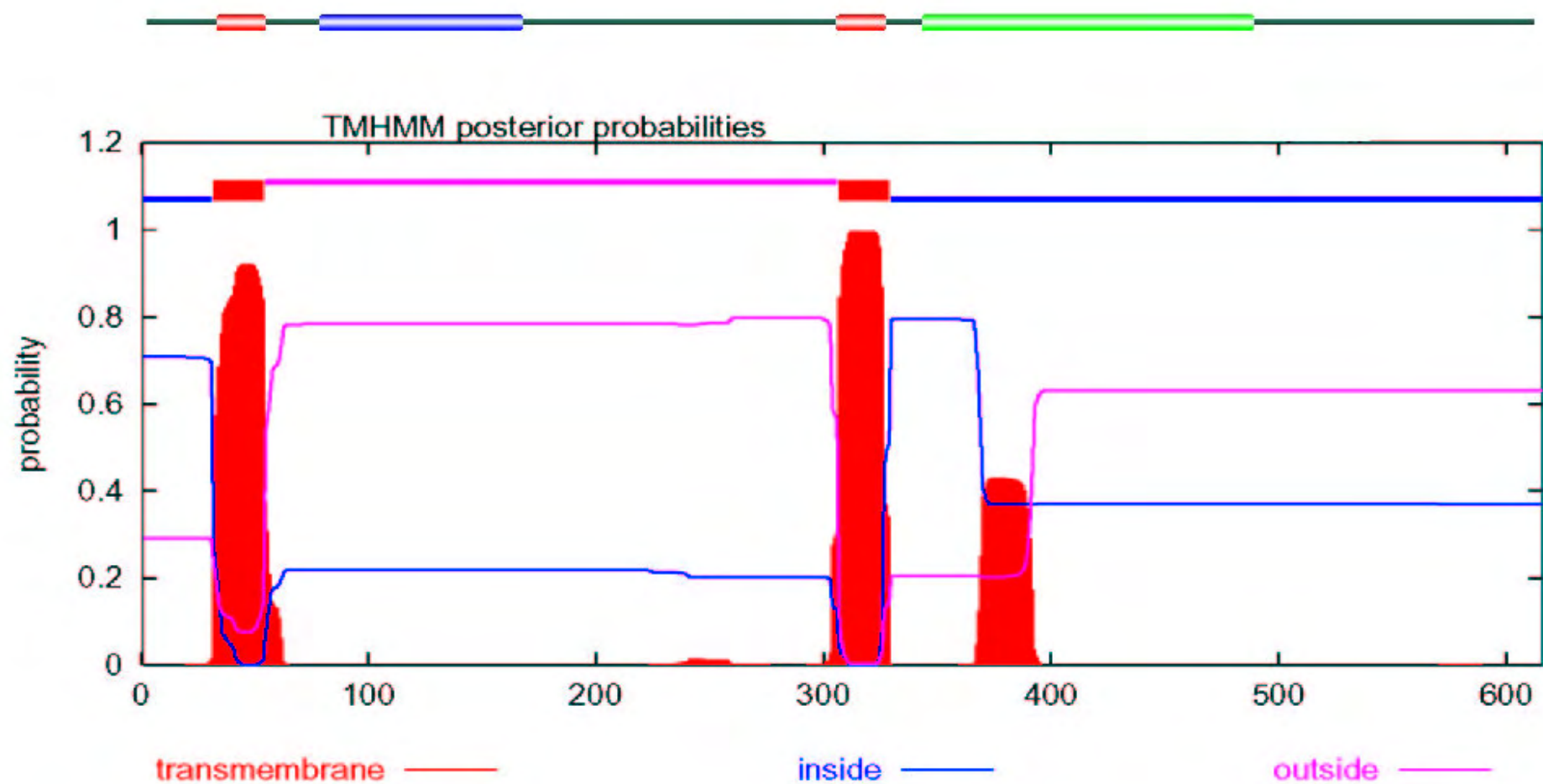

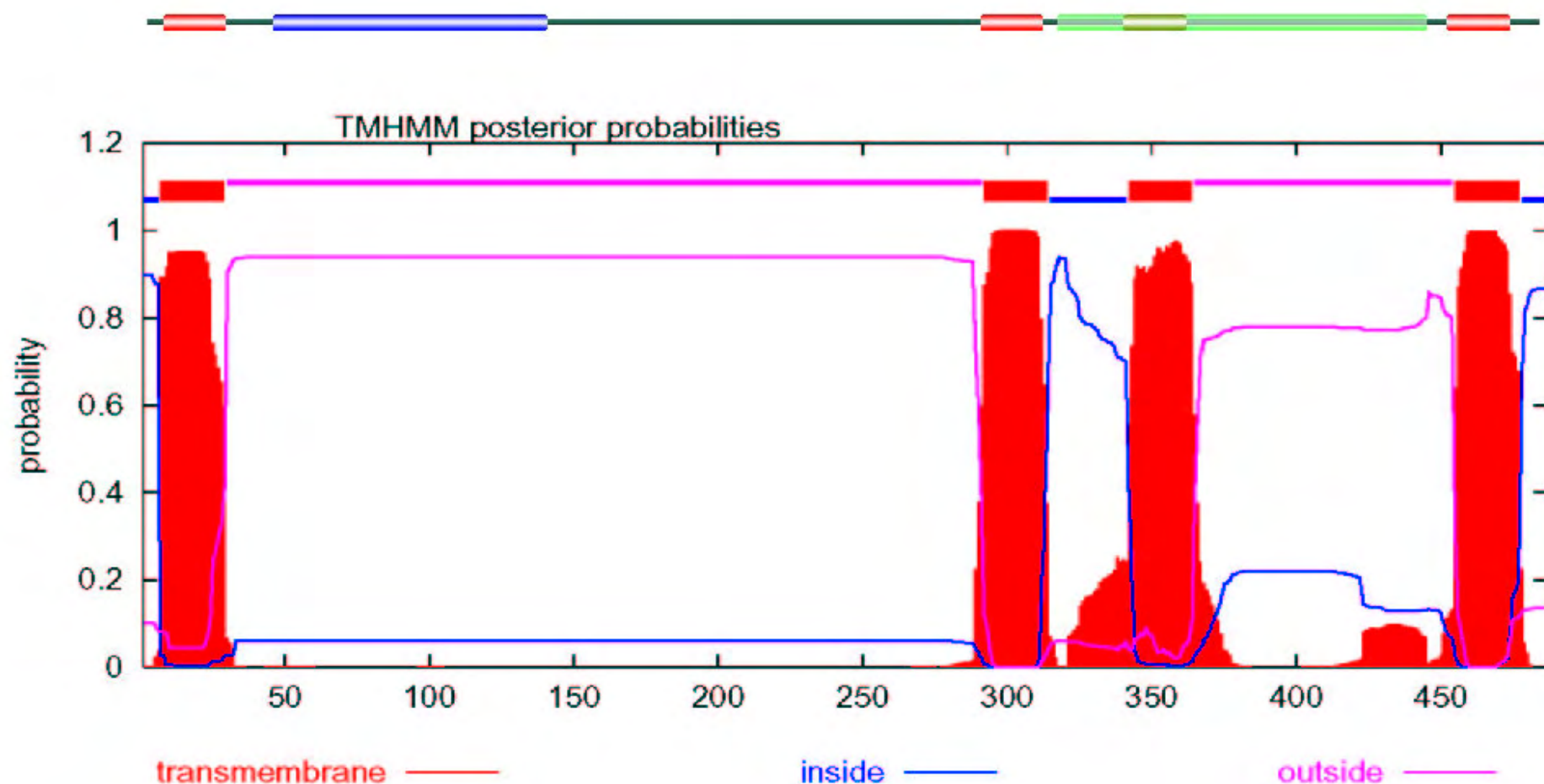

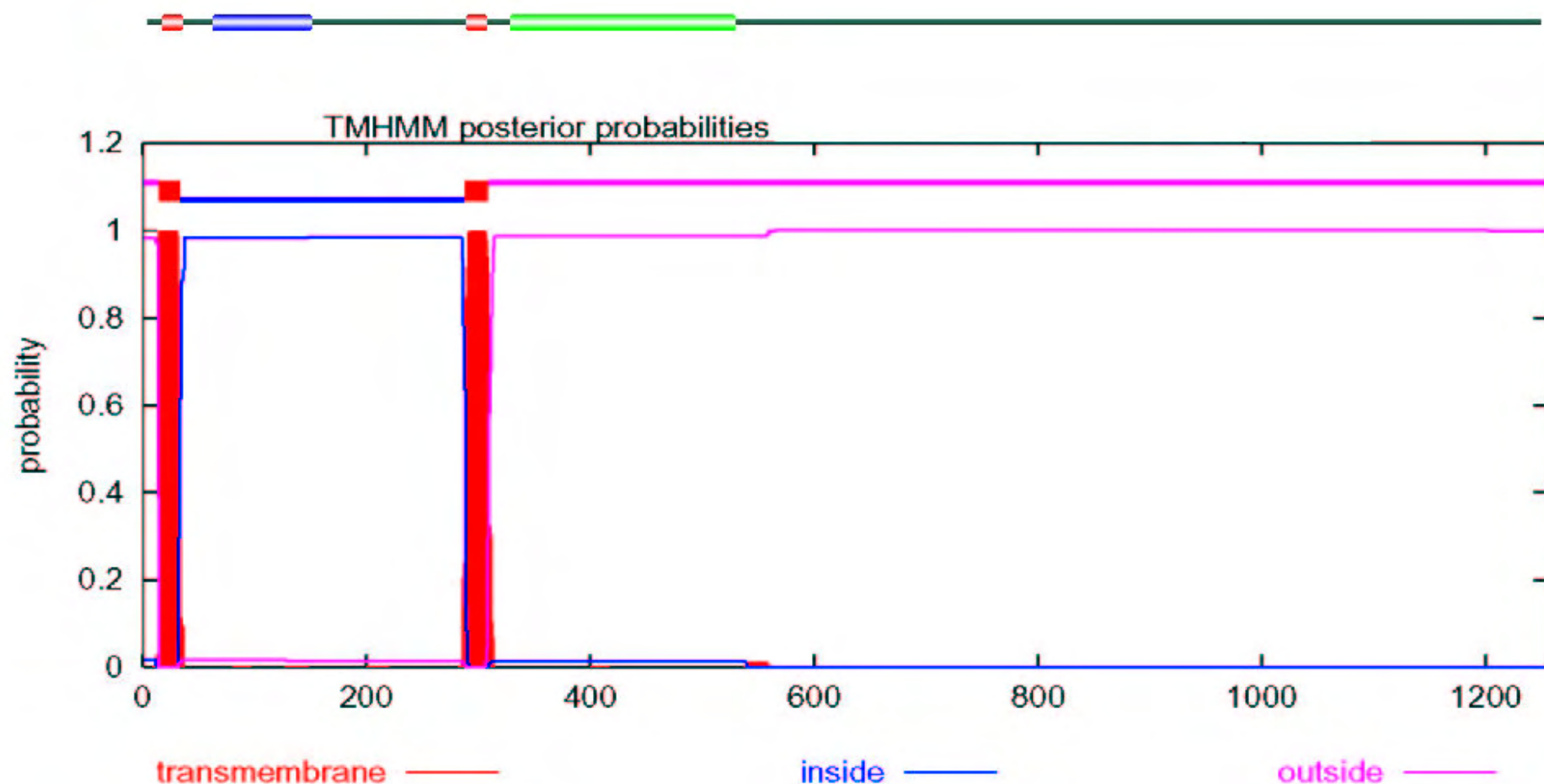

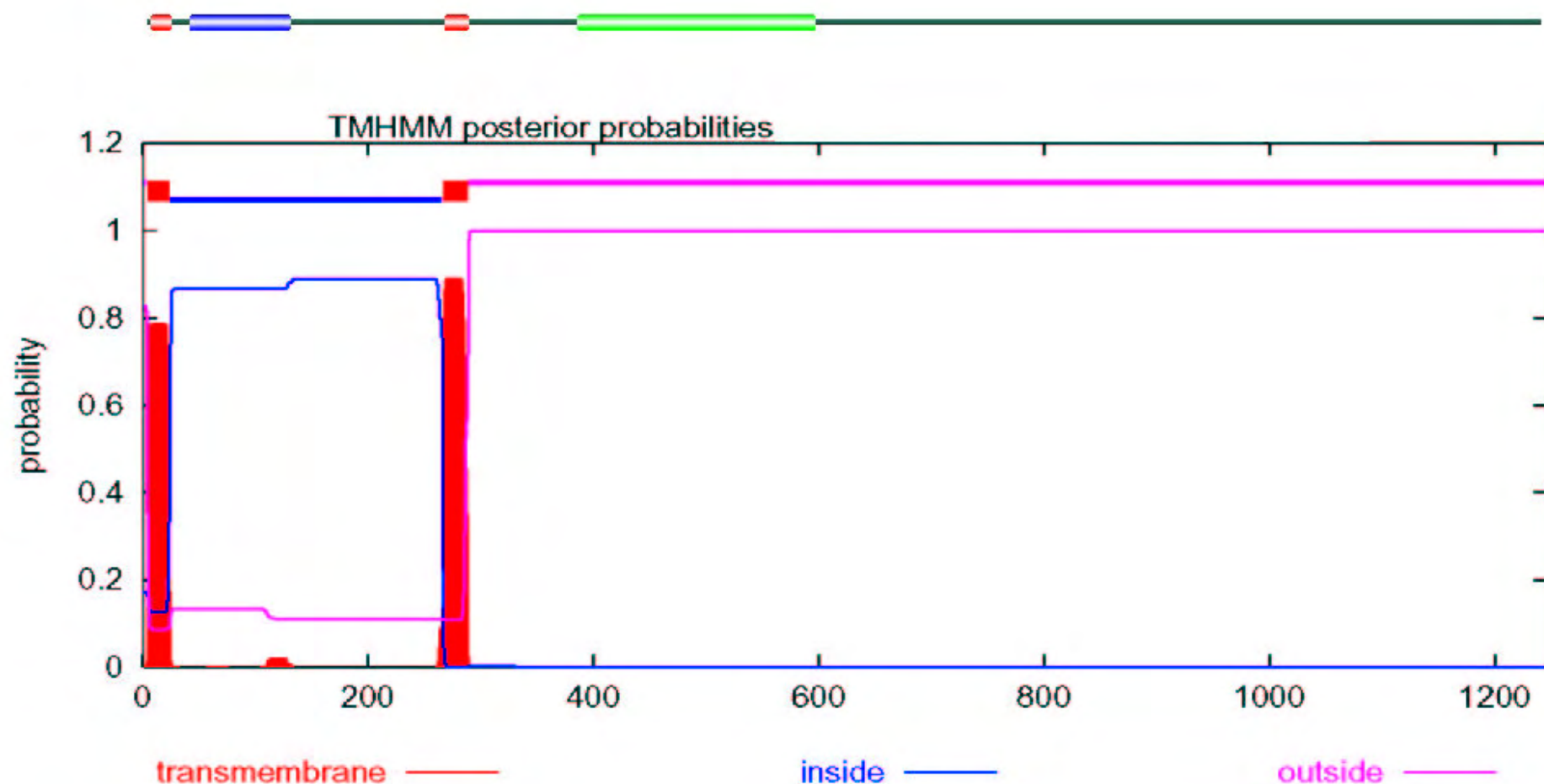

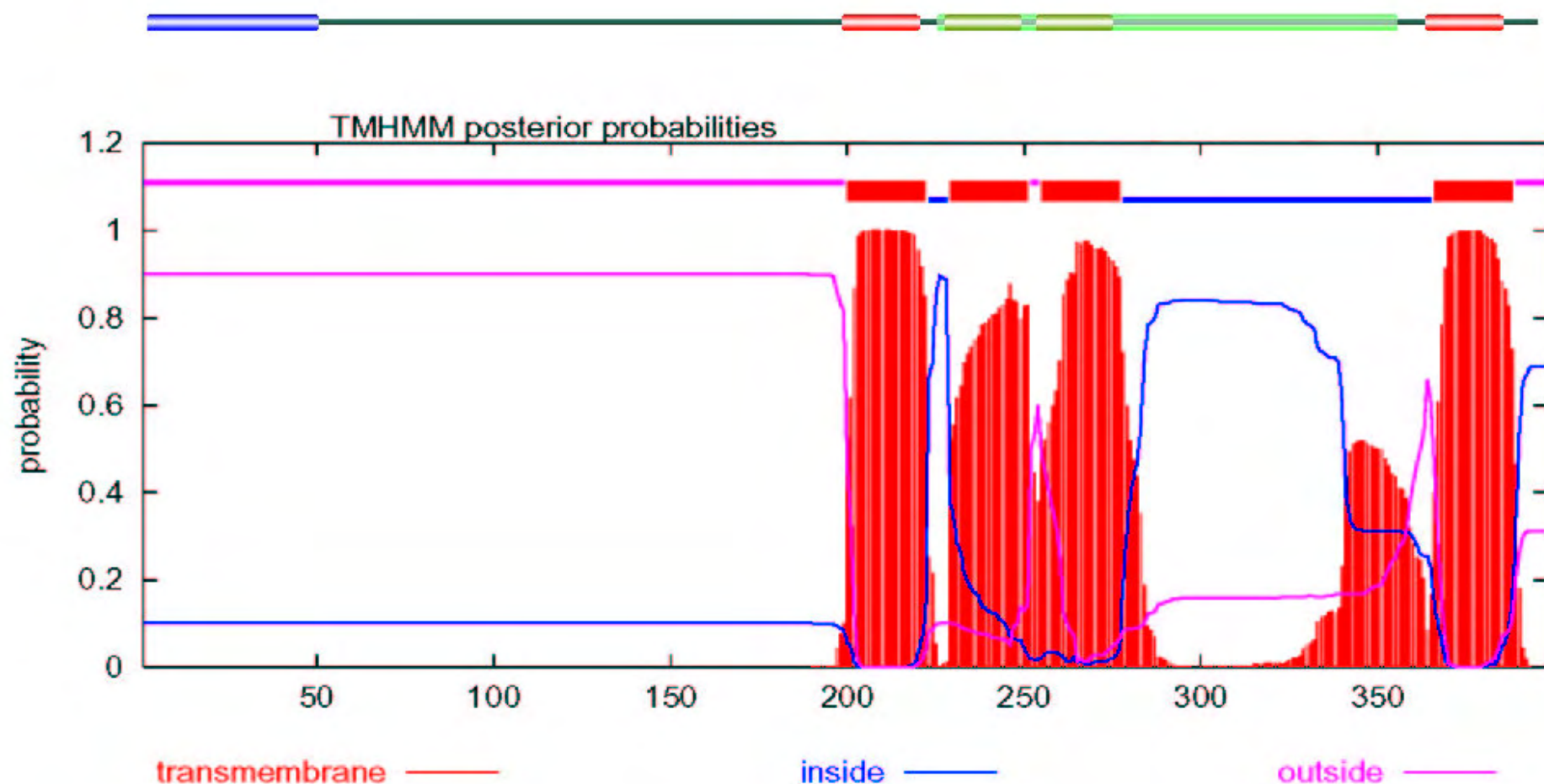

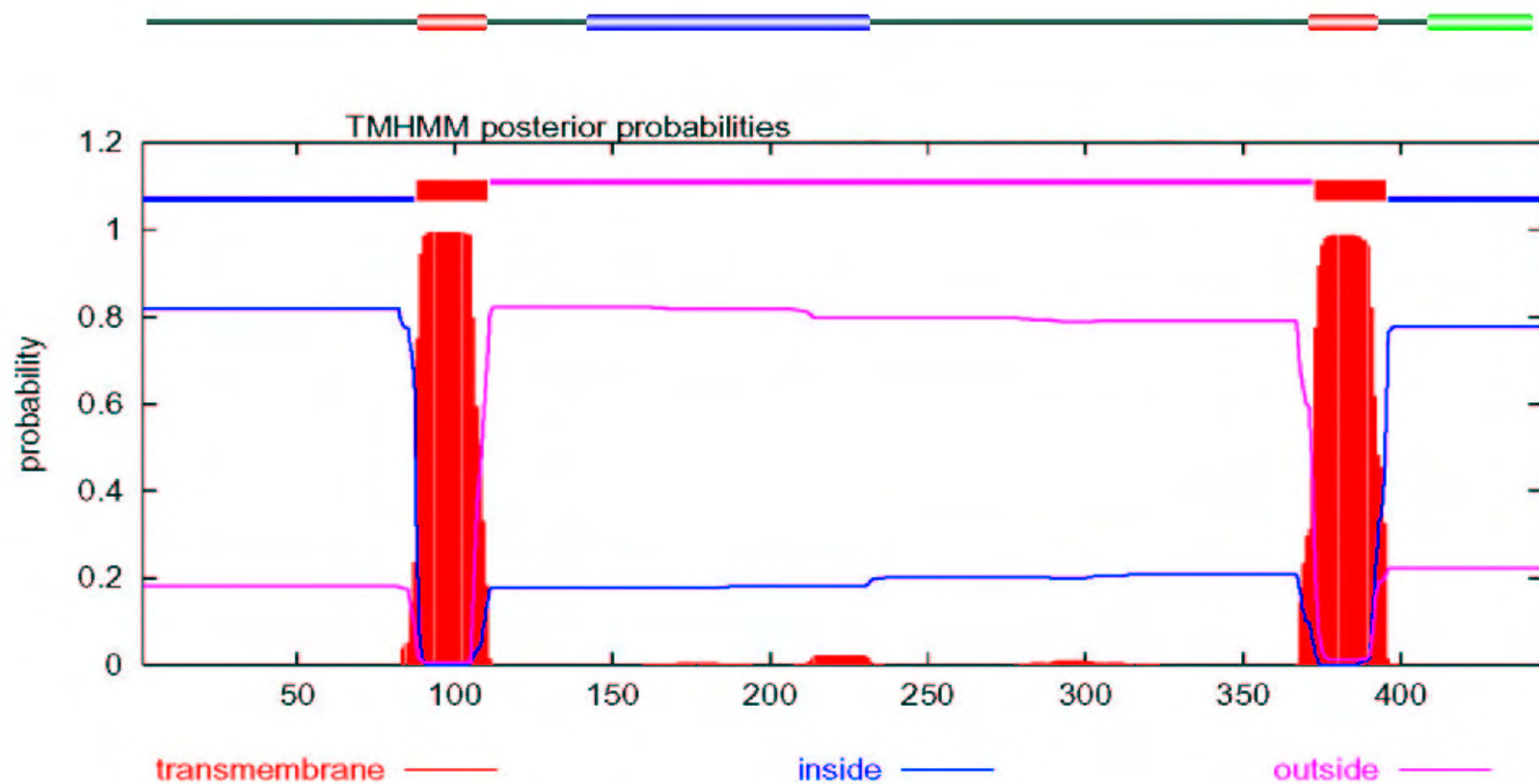

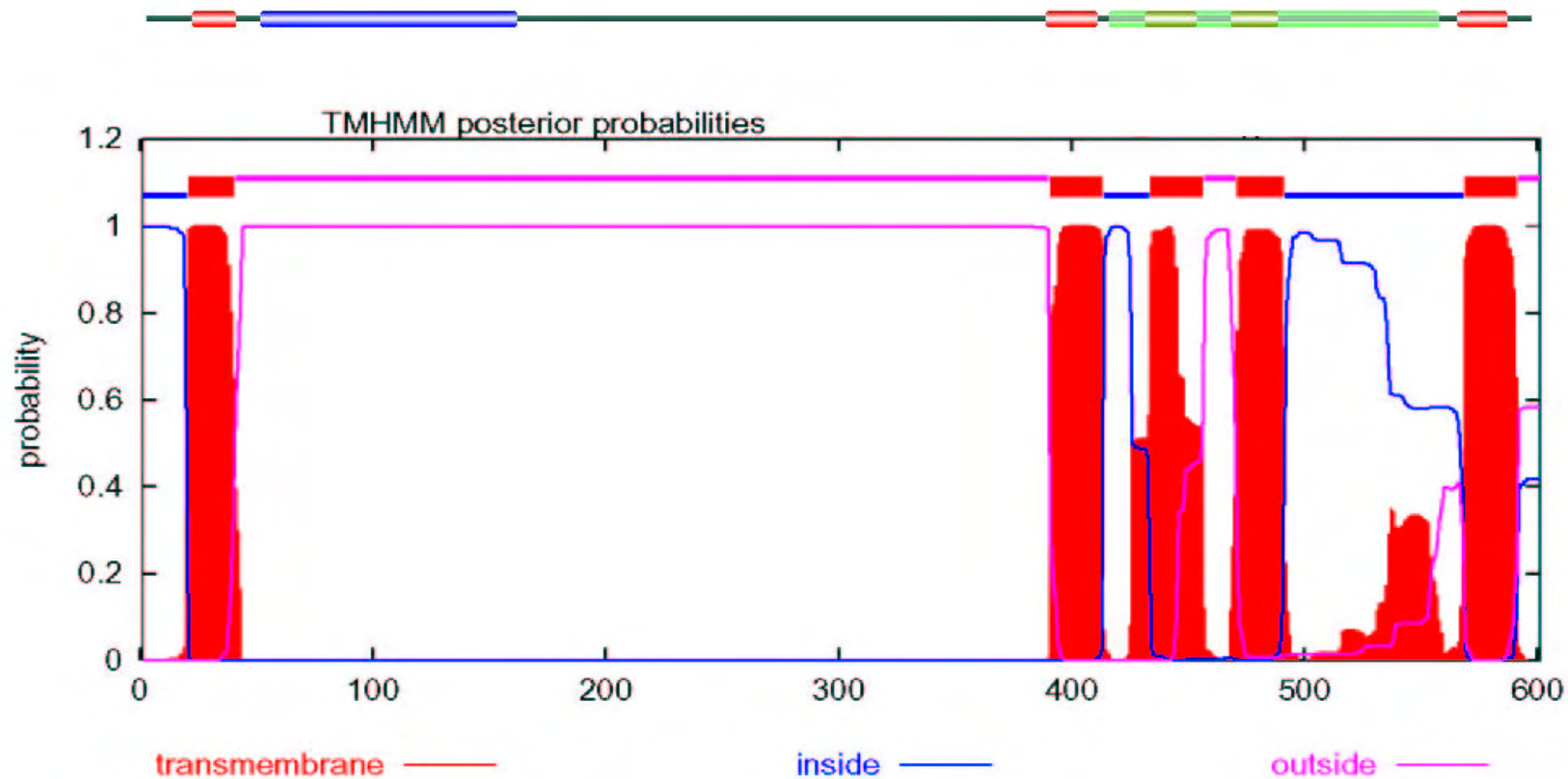

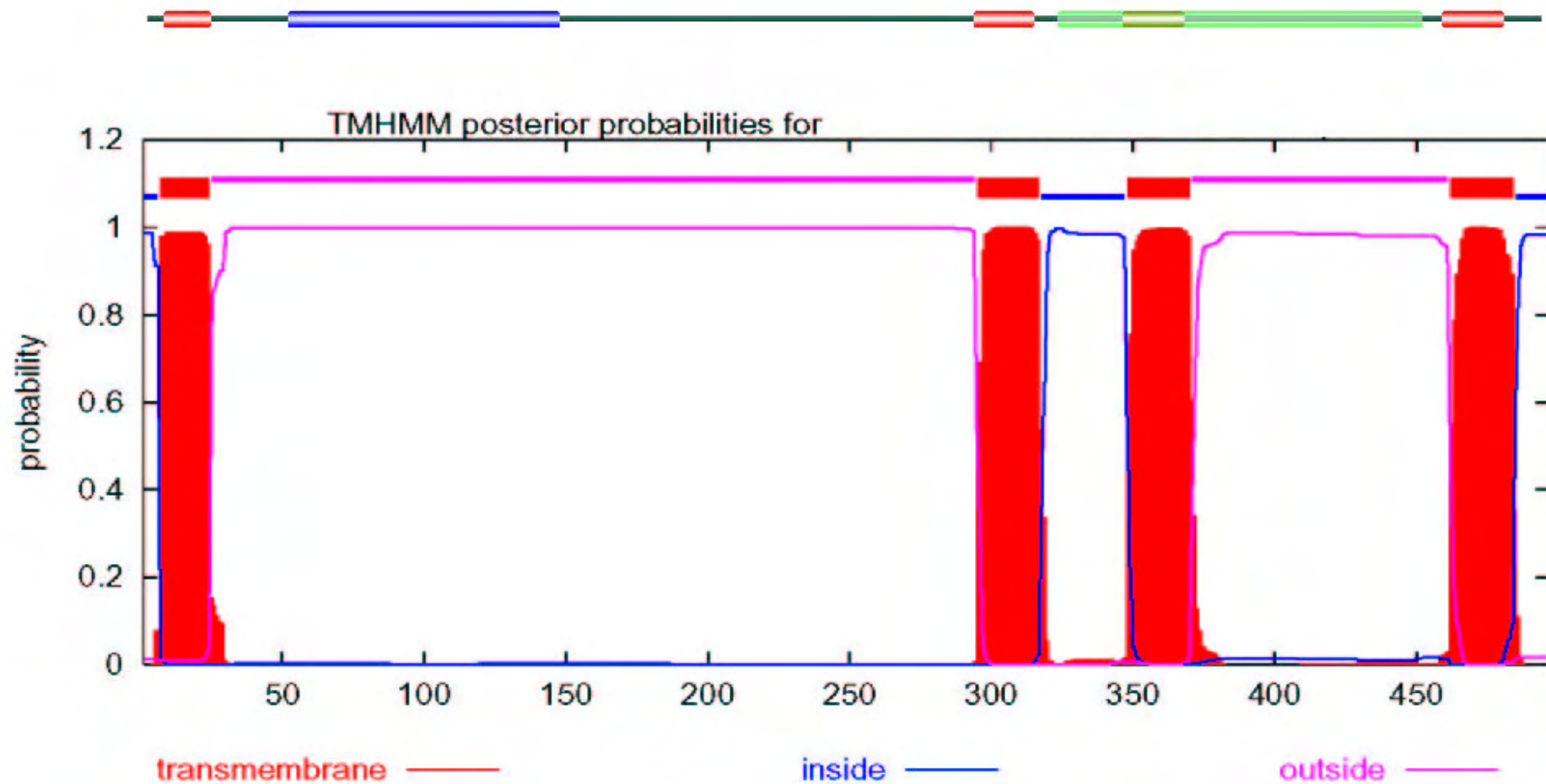

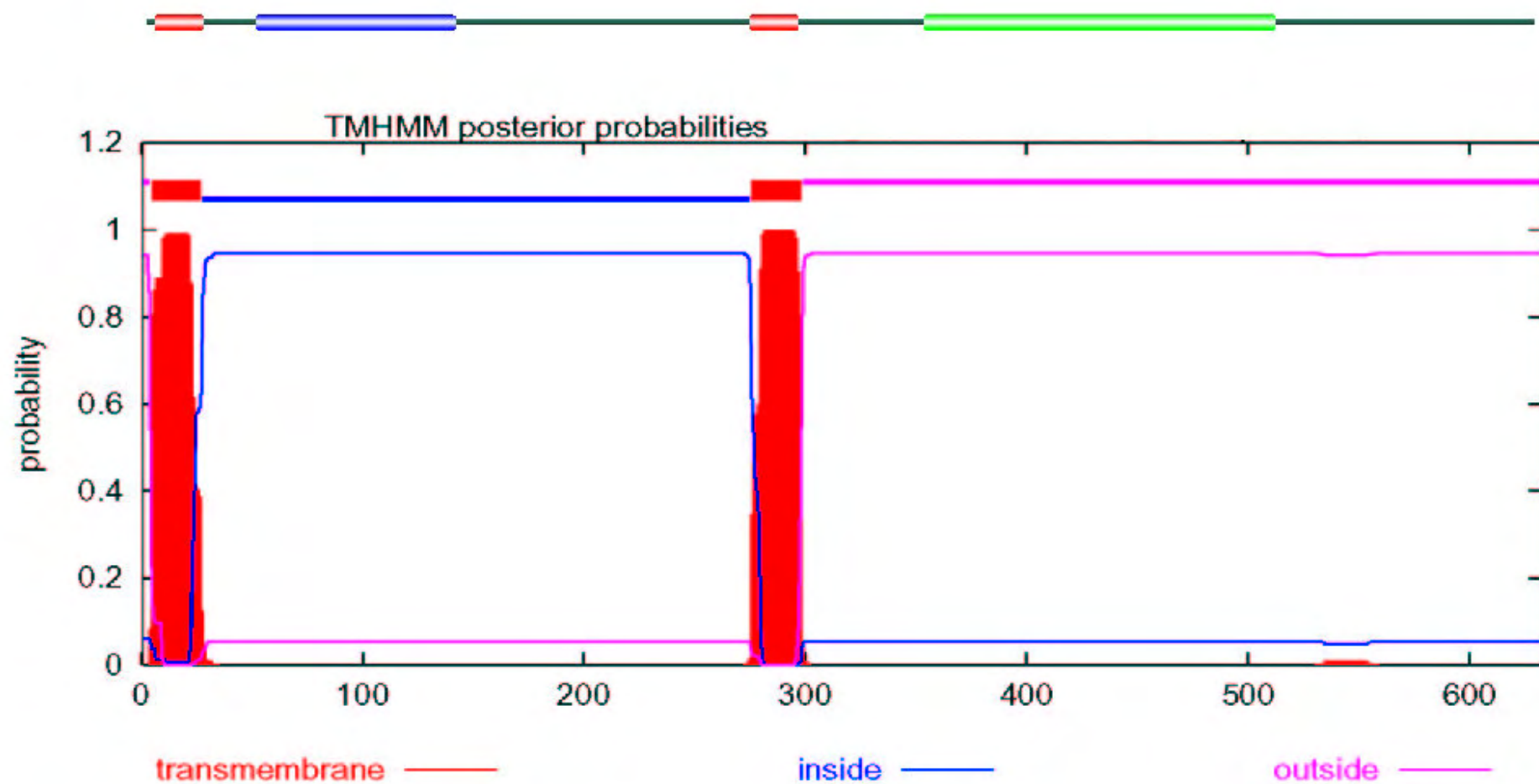

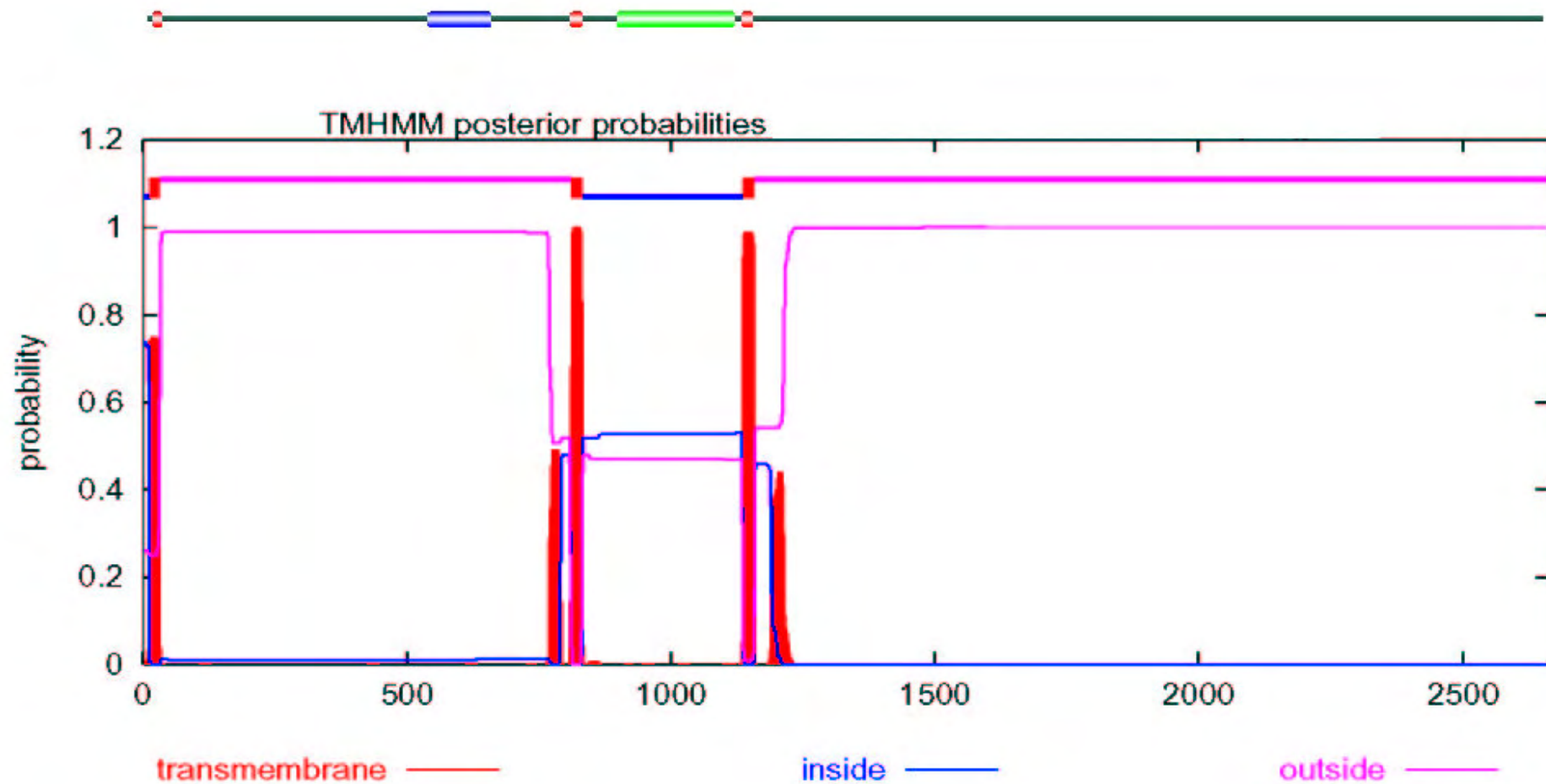

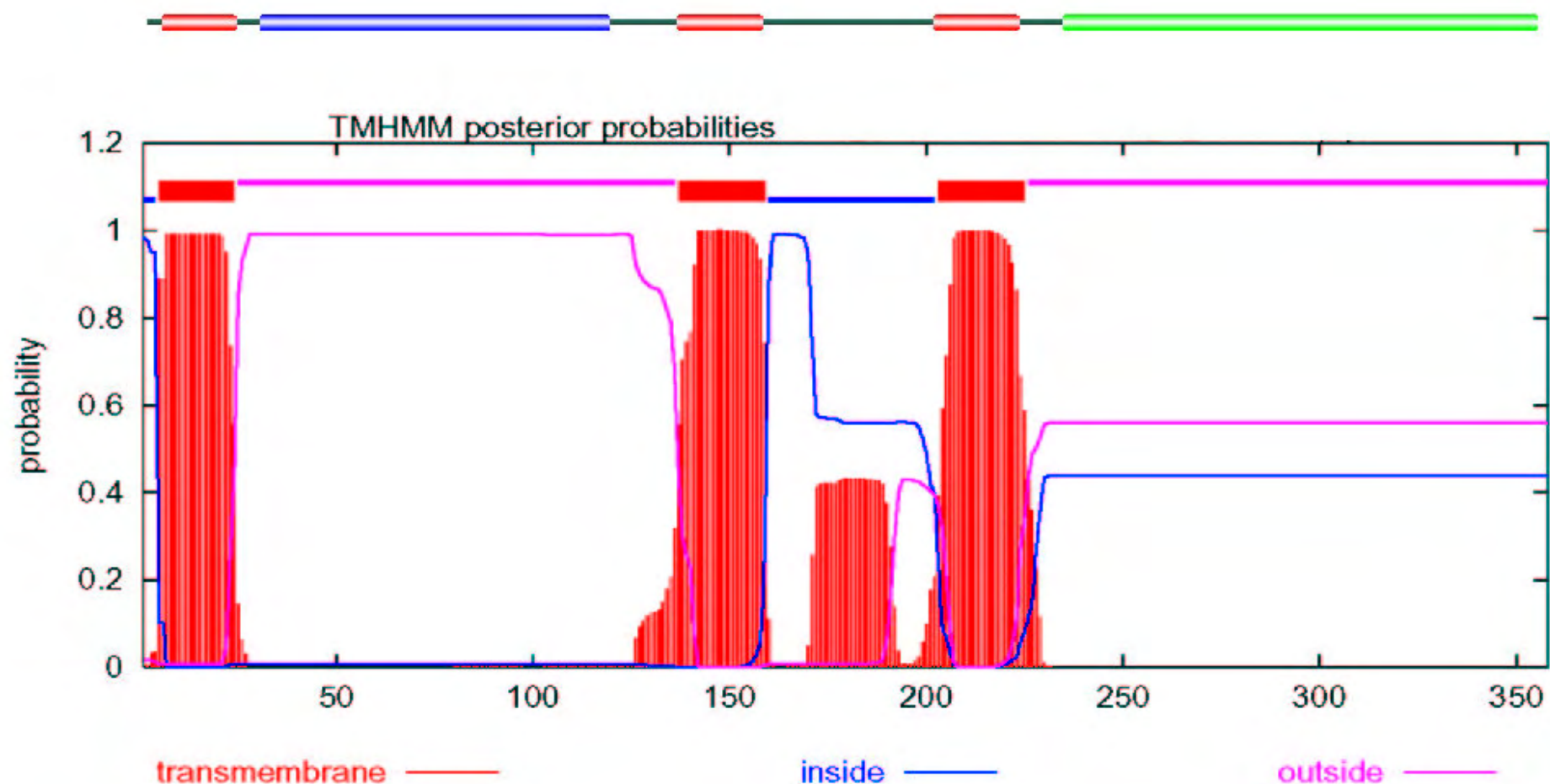

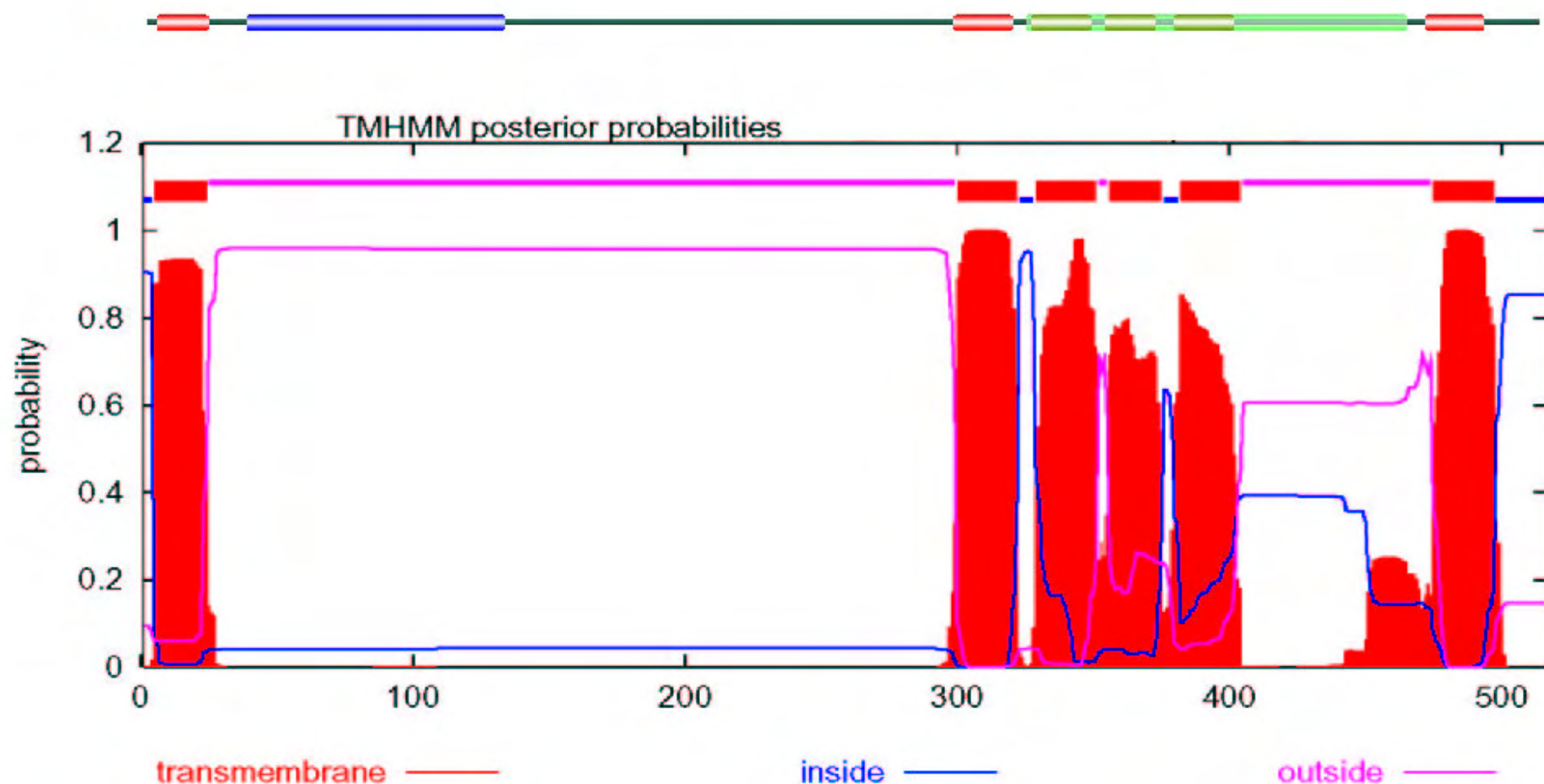

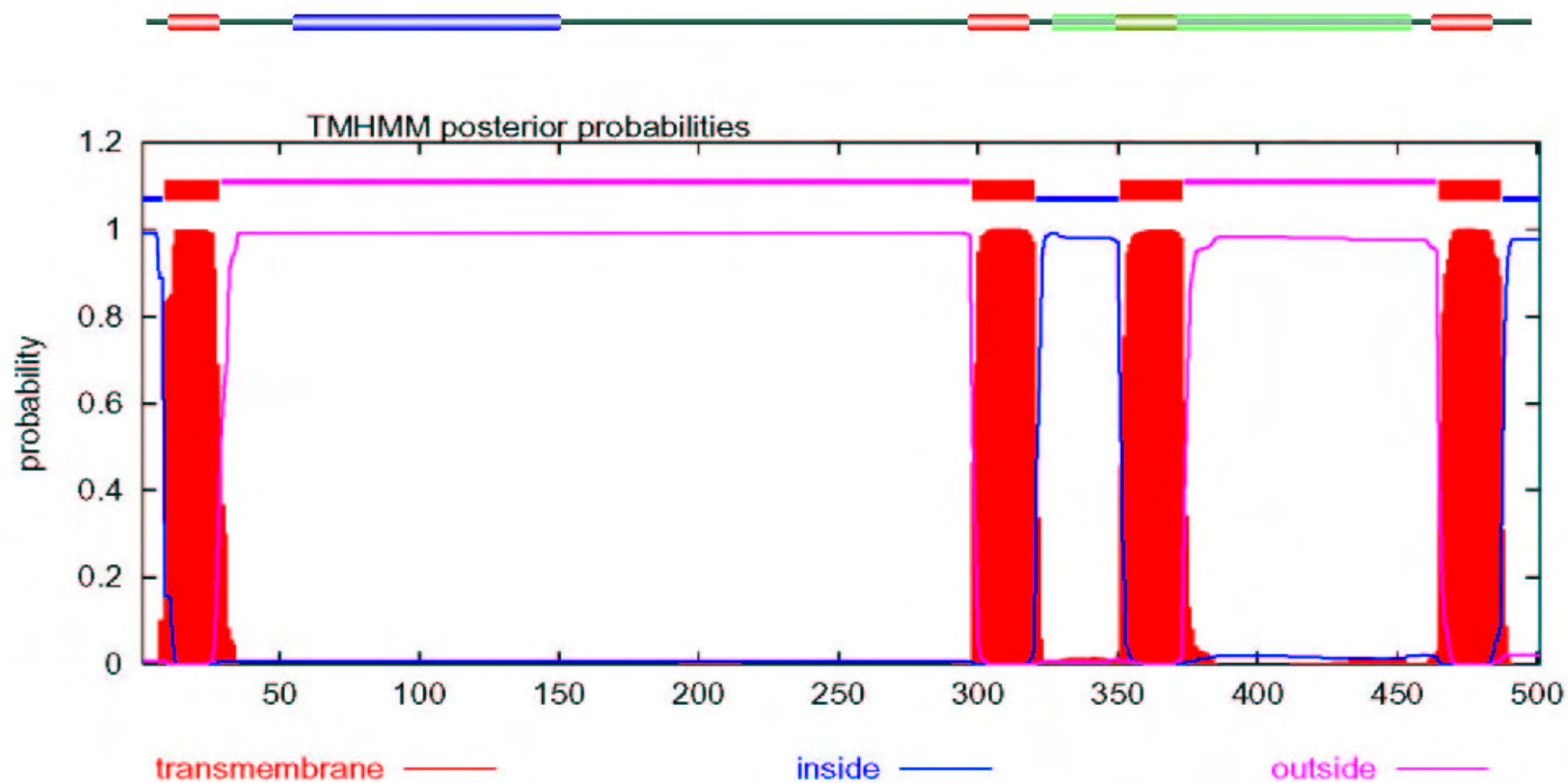

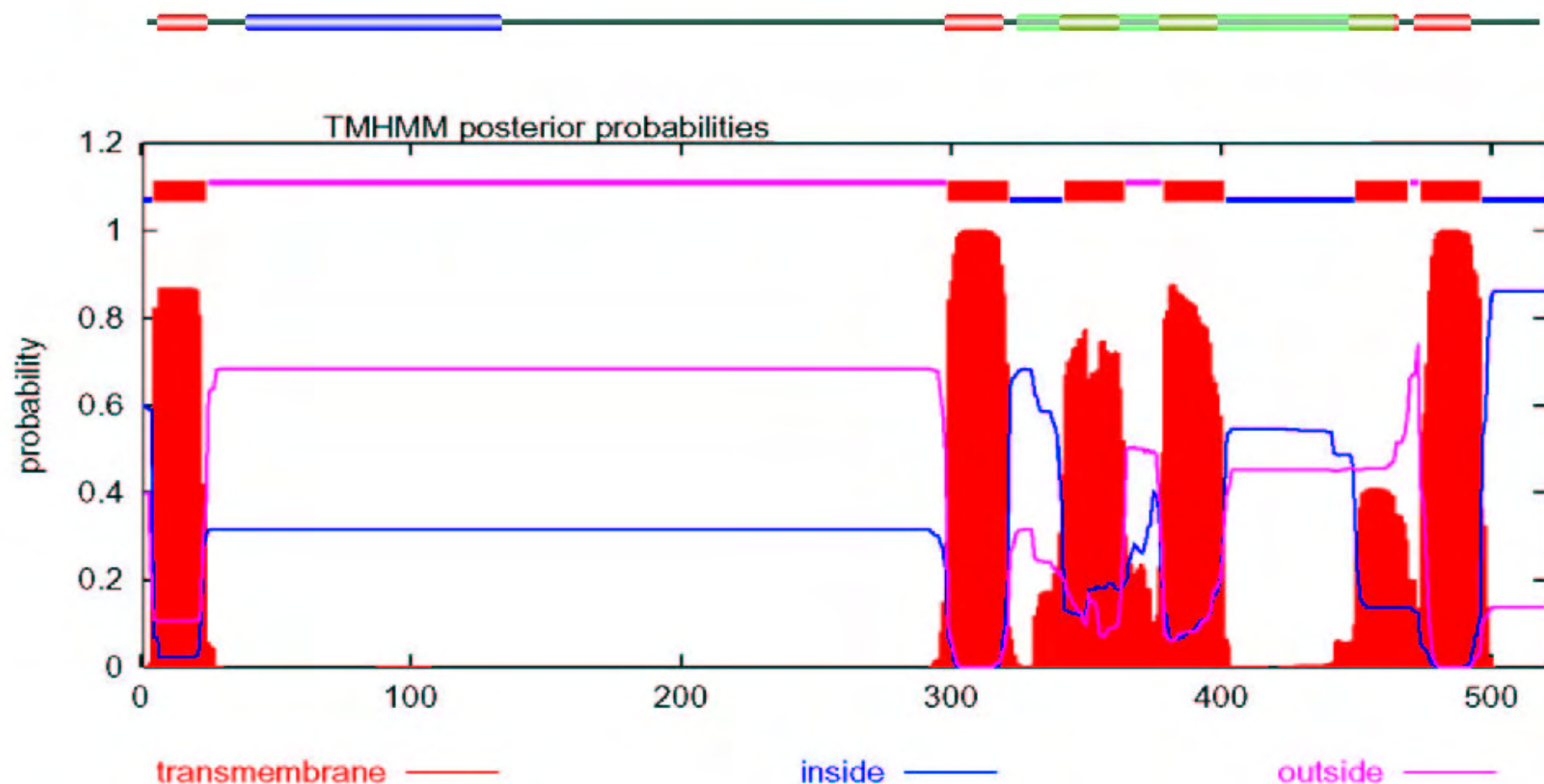

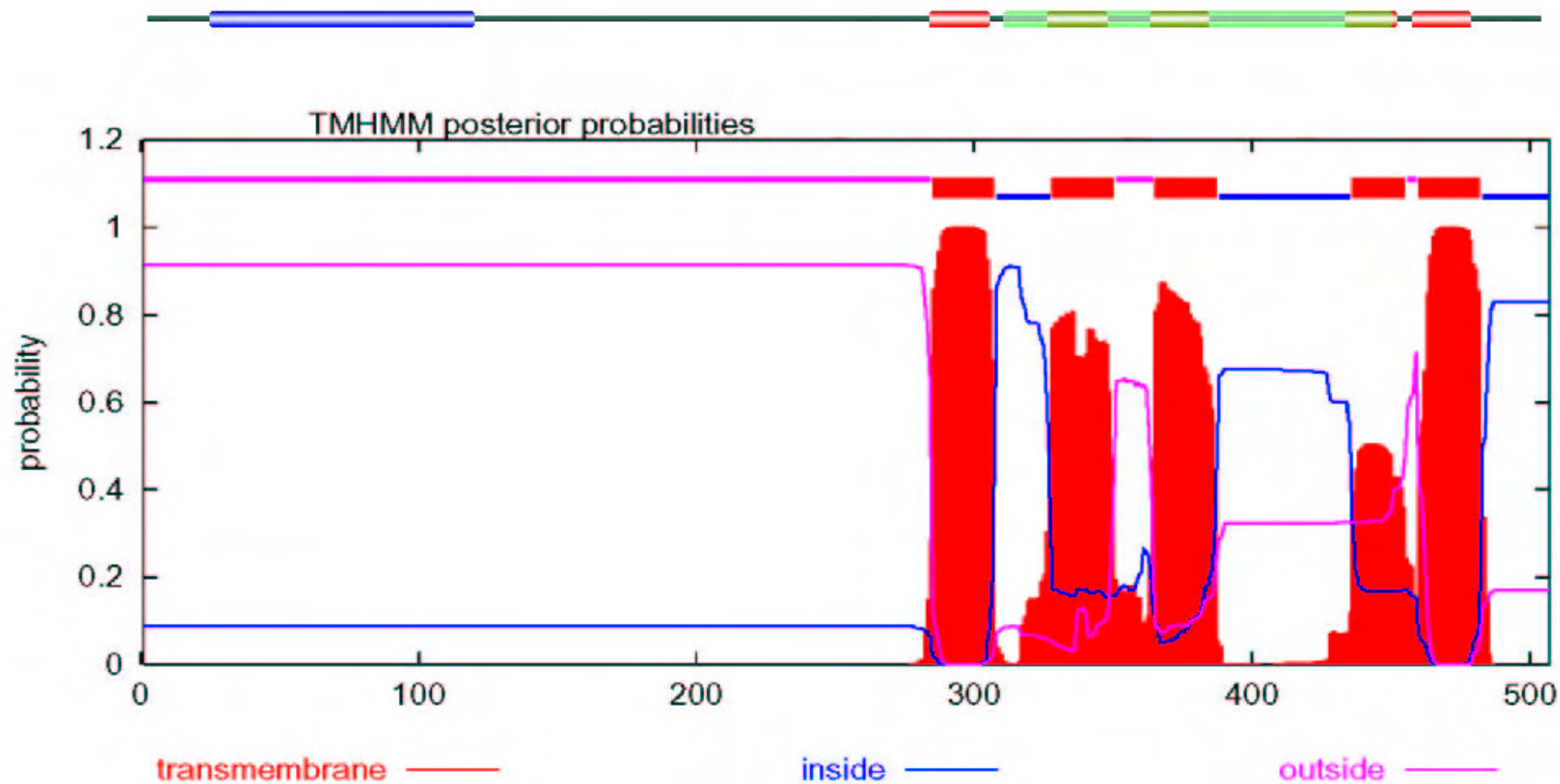

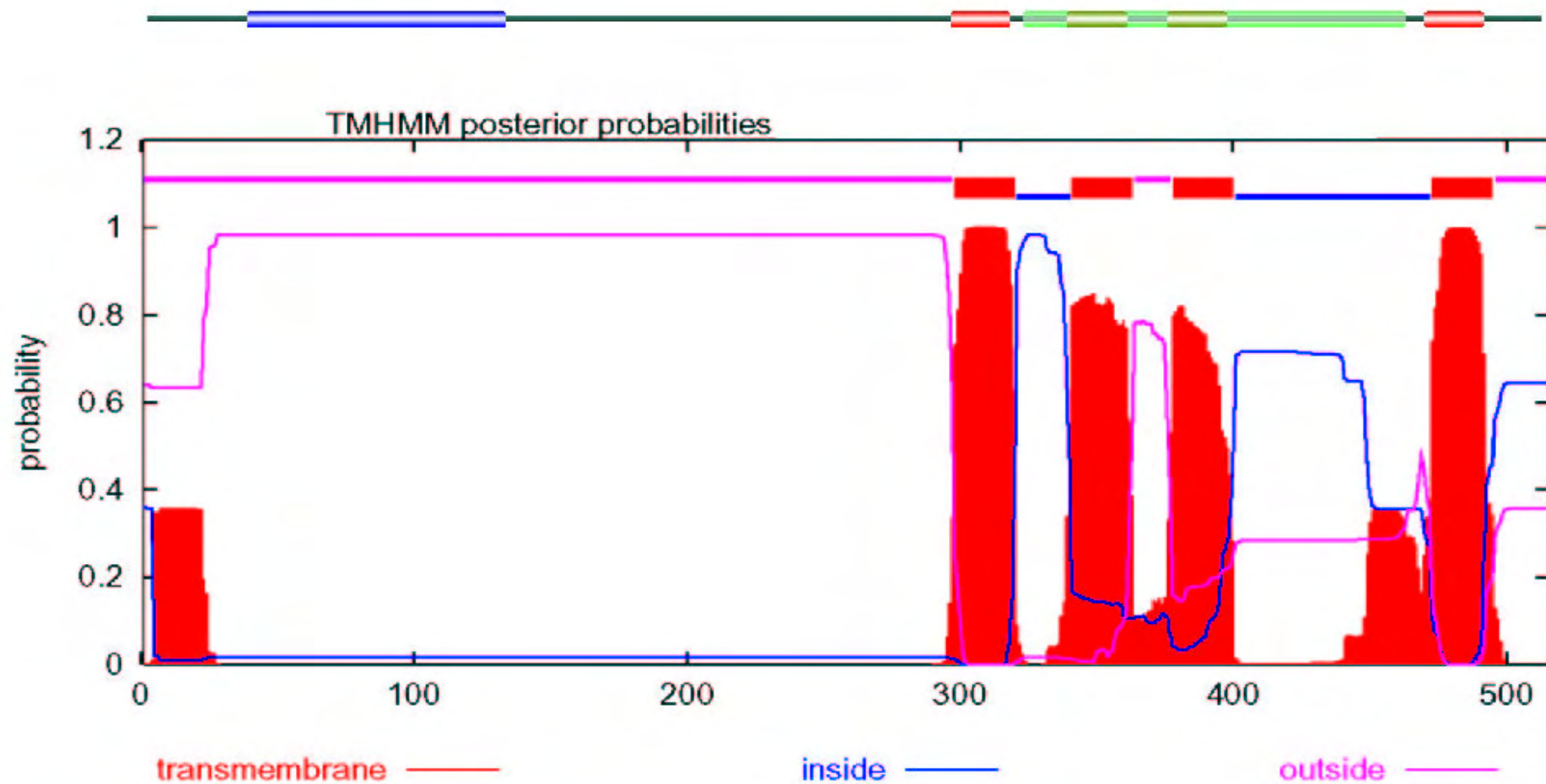

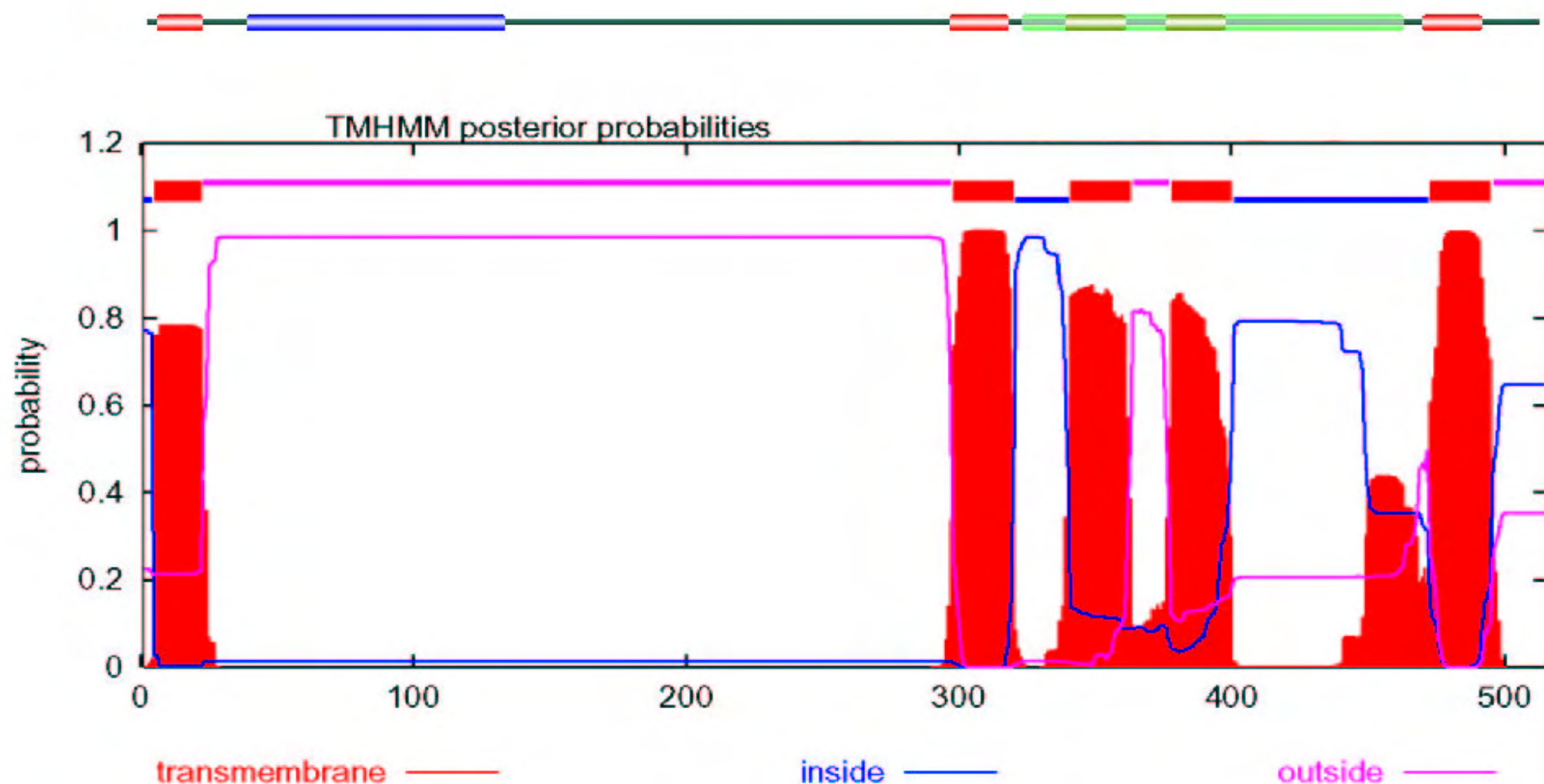

100

Supplement: Data S3 — Topology patterns for 100 sequences included in this analysis. (9.04 MB PDF) [file pone.0012827.s003.pdf]

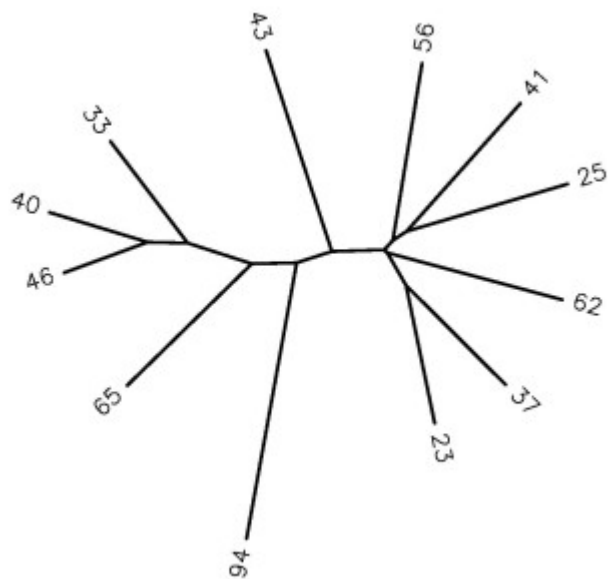

Group 1 : S1

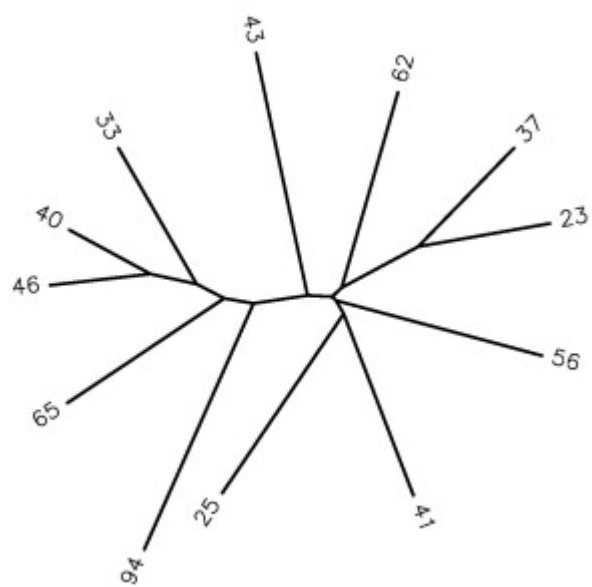

Group 1 : S2

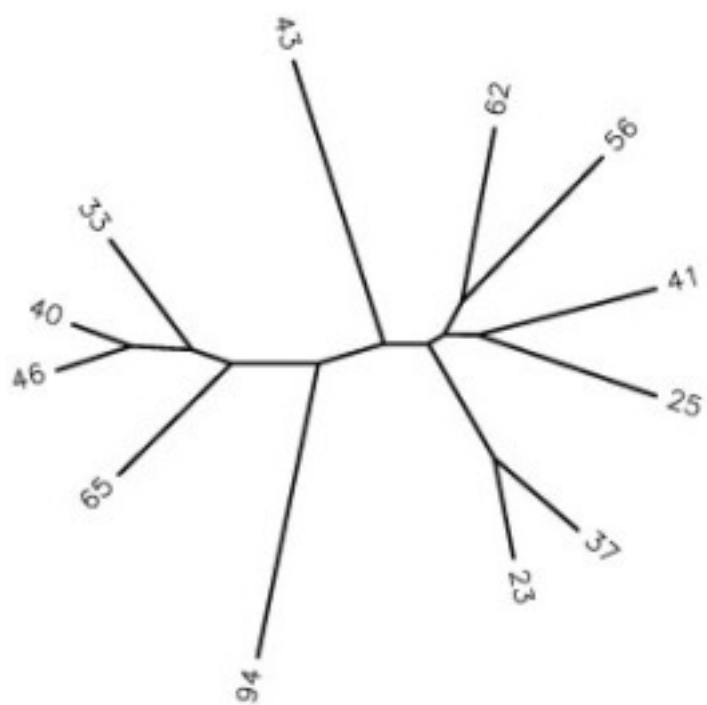

Group 1 : channel

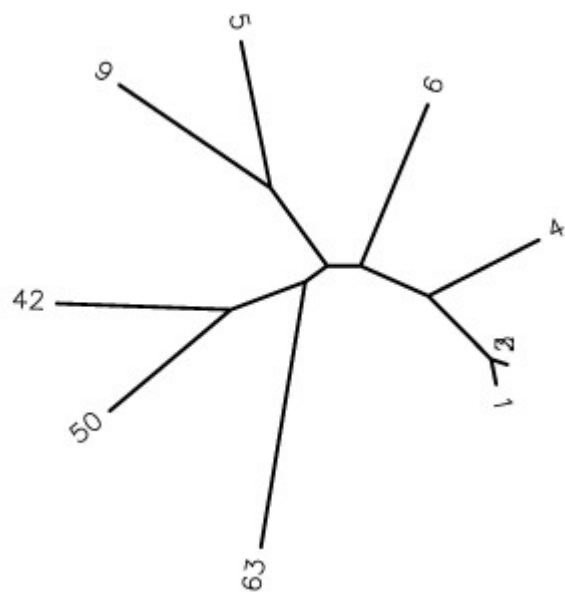

Group 2 : S1

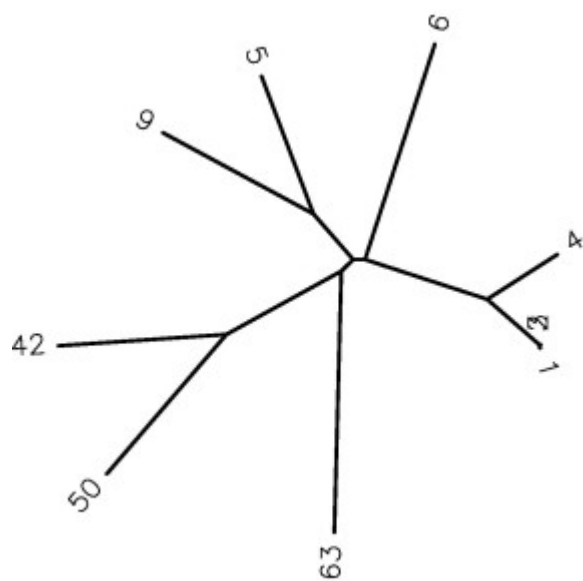

Group 2 : S2

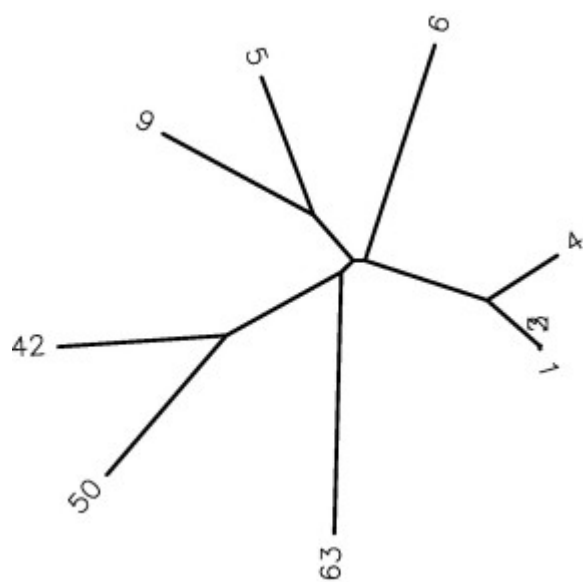

Group 2 : channel

Supplement: Data S5 — Phylogenetic trees of S1, S2 and channel regions in Group 1 and Group 2. (0.10 MB PDF) [file pone.0012827.s005.pdf]
