# Supplementary material for: Domain-Based Identification and Analysis of Glutamate Receptor Ion Channels and Their Relatives in Prokaryotes
Source: PLoS One. 2010 Oct 6;5(10):e12827. doi: 10.1371/journal.pone.0012827 (PMC2950845; doi:10.1371/journal.pone.0012827)
Supplement: Data S4 — Sequence alignments of S1, S2 and channel regions in Group 1 and Group 2. (2.00 MB PDF) [file pone.0012827.s004.pdf]

group 1 S1 region alignment

```
                  10        20        30        40        50        60
                   .         .         .         .         .         .
  1  LKVGITGSPPFVIE...ENNSFQGISLDVWRQVAEDNELVYDLVPQSSPEAGIAAVDQGQ  37
  1  LKVGVSGSAPFVIQ...EEGGSSGISLQVWRRIAEDNNLSYRLIQQATPQKGILALNDGE  23
  1  LRVGVSGSPPFVDK...KAGVYEGISVEVWRQIAHAEKLEYFLIPYPNIDSNIKAVADGK  62
  1  LKVGVVGYPPFSDY...NEETFRGISVELWQEIAADANLRYSLIAQPGIQVGIDGVVAGE  41
  1  LRVGVAGAAPFV.I...KDNVFKGISVEIWKELALSEGLEYEFVPQNSVANSIDSINKGE  25
  1  LKVGVVGNPPFVFYGEGKNAAFTGISLDVWRAVAESQKWNSEYVRQNSISAGITAVAEGE  56
  1  LTVGVAGDAPFVIHNP.QNNSLEGISLTIWENIADKKNWEYQYKYFTTVDEALTALQQGE  43
  1  LTVATKHSPPFAMQK..ENGEWEGISIDLMRAIAKKQDLGITFIPMS.LPDMIEAVAVRR  46
  1  LKIATKHAPPFAMQD..EHGKWNGLSIDLITAIAEKQGFRATFVPMS.LLDMLEAVNRGE  40
  1  LIVATKHSPPFAMKN..AEGEWEGISIDLMHALAEKGKSKLTFKEMS.LTEMLKAVEQGE  33
  1  LIIGTKHSPPFVMKK..SDGSWEGISVALWDKIALENGFSYEFREME.LSDILKGVADNE  65
  1  LKVGYQIAPPFIVE...ESHGIPCPEIERRKHSAASMKVHYARLEYR.LNNLLKALETGE  94
     Lkvgv-g-pPFvm------g-w-gisidlwr-iA----l-y-fv----l---i-av--ge  consensus


                  70        80        90
                   .         .         .
 58  IDVLVGPISITPRRLAMPGVDFTQPYYLAKSGVLLPL  37
 58  IDLLVGPISVTPDRLNLPGVDFTQPYFIGKEGILLPL  23
 58  IDLAIGPISITPDRVARNGIEFTQPYFYAEEGVLVPS  62
 58  LDVLIGPISITTER..FQKVAFTQPYFNAQIGLLVSA  41
 57  LDIIIGPISITSER..LEKVAFTQPYFYAKIGLLASP  25
 61  LDILIGPISVTPERAAIEGITFTQPYFSSGIGLLIPG  56
 60  VDLLAGPISITSER..VEKFLFSQPYYQSSLTIASRD  43
 58  VDIAAAALTMTVER..EEQLDFSHPYFQSGLAIAVRE  46
 58  VDLAAAALTMTVER..EKQLDFSHPYFQSGLAIAVRE  40
 58  VDAAAAAITITKER..EYLLDFSAPYFHSGLAIAVKE  33
 58  LDAGVAAITVTAER..EETLDFSQPYYLSRFGIATLE  65
 57  IDIAISPLTVTASR..MKKFSFSQPYYITNLAFAMKI  94
     iDi-igpisiT-eR-----ldFsqPYf-s-lgi-v--  consensus
```

X non conserved

X similar

X conserved

X all match

group 1 S2 region alignment

```
                  10        20        30        40        50        60
                   .         .         .         .         .         .
1     VIKDFTQVEDIRDKQIAVVSGTTGEKWAQIYQSRVLPSPNLEQAIERLKSGQAEGVMFD  41
1    QRVTEFSYPQDIKDARVSVIKGSTGEKWAKQYEARINQTETLVDAINLLKLNEVDGVIFD  25
1    SATPLFRSVGDLKNKEVAVVRDTTAVDWANFYQADVRETNNLTAAITLLQKKQVEAVMFD  56
1     RETIIRSPAQLQQRRVATIEGTSGVELAERGSMRVLKTENLTSGIELVLSEQAEAVIFD  37
1     TNNSITDPAQLSGQRAAVLEGTSGAELARQRNMRIVPAKTLTAAIDHVLMNRAEAVIFD  23
1     QLSGVMKPSDLDGELIGVVTGTTGVSLARSYGARPFPVPTLKEAIHLIKRNKVSGVISD  62
1        GIRQVDDLYGKKIGTVKASTSSRFLDELAIRGQNFPTVNDALHLLEQGKLDAVVYD  46
1        GIQEMDDLYGKKVGTLEASTSSSFLEELAIKGQYFPTVKEAMQALEKGQIDAVVYD  40
1        EIKEFNDLYGKRVGTVKASSSSSYLEESTMKSKHFLTIQDALHALENDQIDAVVFD  33
1        SVTNPRDLHAVKTGTVEGSTSMQYLNEEYIAFQHFATIQDALQALQERRVEAVLYD  65
1        EINGLDDLRKVKVGCIPSTGTADFLNRFKIKFVDFSTVEDGLVAVQKDEIAAFVYD  94
1       STITQVEQFPGKKIAASAGSPAIGFIEEHQGQKVLVNNLSQAMERLKDKSVDGVVFD  43
     -----i----dl-gkkvg-v-gstg--f-----ir-----tl-dai--l----vdavvfD  consensus

                  70        80        90       100       110       120
                   .         .         .         .         .         .
60   VPALRYYLYQNPT.APLKIADLAVSFEDYGFILPLDNKLIRELDIEIVKLKQSGRLEEII  41
61   VPALQYYLRNDRE.QRLKLSPVNFADEPYGFIVSLDNSFIKNLDIKLLEMRENGKIREIE  25
61   RPALIYYTRQNPN.LNLEVTEIRVSLEPYGFVLKENSPLQKTINVEMLNLLYSRVIAEFT  56
60   RPAMRHHIKQNPH.LAVQLAPFTLAEETYGFVFKTGNPLRNPMNVSILRLQRLGEIKSIA  37
60   RPAIRFHLKNNPE.LAVQLAPFTLAEQTYGFAFRTGDPLRTPLNISILKLQRSGAVEAIS  23
60   TPILSYYMKNSPD.KSLTLAPFRLSLQTYGFVVPSDSPLERLINIELLRLERSHQVKAIS  62
57   EPILRYLITTGEI.HGIKVVNRSFRHEYYALALPPRSTERETLNRNLLEIINSKDWKEIR  46
57   EPILRYMITSGEI.HGVRVVERSFKPEYYAFALPPGSPETETLNRNLLGIINSLAWKEIK  40
57   EPIMRYVLTLGDI.HGVTIINQVLSPEYYAIALPQGSQLRETFNRNLLDITVSERWKKIN  33
57   EPIMRYMIASGKF.QGIKVIETSFRYEYYAVALPPDSPRTEDINRSLLHYLESENWRSVQ  65
57   HATLSYYVNEGSFDEVIEVIPSSYYREYFSFATS.NYAMLKPVNESLIKIIESDNWVVKL  94
58   RPQLLFYKNKHKA.ENIHIPSAEYYKQGYGFAFRADSKLVNDVNLVLLRLAEDQKIEQIM  43
     -p-lry-i--------i-v-------e-ygfal---s-l---lni-ll-l--s--ik-i-  consensus

             130
              .
119  EREIKGDFENQ   41
120  SKWLSSSKDN    25
120  ERWLGPGIEENQ  56
119  NKLLN         37
119  KRLLN         23
119  DRILK         62
116  FKYLHNE       46
116  YKYLREE       40
116  NKYLNTKQ      33
116  FRYLDVVP      65
116  KKYHVENYK     94
117  AEYLGDK       43
     -kyl--------  consensus
```

X	non conserved
X	similar
X	conserved
X	all match

group 1 channel region alignment

```
                   10        20        30        40        50        60
                    .         .         .         .         .         .
1    DSPTIFSRVKVFFGWAVISSILVLISVLLVVGSLIWLAERRENSEQFPKRWLPGIASGM  37
1    KPSTLLNRLQVFLGWAVLSSVLVLITVLLVVGSLIWLAERRSNSEQFPAQPLPGIASGM  23
1    QPPGLWARIKPLFGVAALSSITFLLFTLFCVGNLIWLAERKRNPEHFPPQYIKGLGNGI  62
1    KPVSLWERFSPFFGIAALSSAGVLTLLLFLVGNLIWLAEHRKNPEQFSPHYPEGVQNGM  56
1    EKVSVWSRIRPIFRVAVISSVGGLFLILFVVGNLMWLAESRRNPEQFPRQYIRGVGSGM  41
1    ESPTVWSIIRPFFGLAFISSVCLLTVLLFIMGNLLWLAERRRNKEQFPPNYFHGVGNAM  25
1    ENASIWGKIKPFFSMQLLIAVAIFLSLLAIVGLLFWLAERKHSPEQFPADPKRGIANGM  43
1    KDDGWEYALRRLFSPAFLKVLGGLSLLLLLSGFLVWVFERRNNPDNFGGGPVEGIWSGF  46
1    KEDGWKYALGRLFSPAFLRILGGLSLLLLLSGFLVWLFERRQNPGDFGGGPAEGIWSGF  40
1    KETRWFHMIGRLFSPAFLQVLAALSLLLLISGFLVWLFERKKNSDHFGGTPAQGIWSGF  33
1    EGVDWKNILKVFFSYGFFRIVVLLSLVLLLSGFLVWLFERRKNPEHFGGSPTHGIGSGF  65
1   EKSKDLISFFLNFFTLNFFKAIFSLFLVILIFGLAVWLFEKKRNSAQFREG.LNGIGDGI  94
    -----lw--ik-ff--a-l--v--l-llllivG-lvWlaErrrnpeqF------Gigsgm  consensus

                   70        80        90        100       110
                    .         .         .         .         .
60  WFAVVTLTTVGYGDKAPISRTGRGITATWMVISLIAVSSLTASLASAFTLFFSGA  37
60  WFALVTLTTVGYGDKAPITRIGRGLTAIWMVTSLIAVSSLTASLASAFTLFLSGD  23
60  WFALVTLTTVGYGDRAPLTKAGRSIAGVWMVISLASVSTITAGLASAFTVSLSQT  62
60  WFALVTLTTVGYGDRSPRTKLGQLVAGVWMLVALLSFSSITAGLASAFSTALSEA  56
60  WFALVTLTTVGYGDKAPITKTGKIITSLWMLTTLVAASSLTAGIATVMTLLLSAE  41
60  WFALVTLTTVGYGDMTPVTKSGRIITGVWMVLTTVTISSLTAGIATALTVSLTN   25
60  WLAIVTMSTTGYGDMAPVSLKGRVVAGCWMIISLIFATSMIAGIASTLTVSSFSS  43
60  WWAAVTMTTVGYGDKAPKTPPGRIVALIWMFTSLVVISGFTAAMTTSLTVGSLGT  46
60  WWAAVTMTTVGYGDKAPKTRPGRMVALVWMFTSLIVISGFTAAITTSLTVGSLGS  40
60  WWAAVTMTTVGYGDKTPITLLGKLVALVWMFTSLMVISGFTAAMTTVLTVESLES  33
60  WWAAVTMTTVGYGDKSPKTPAGRVVALVWMFASLVMISGFTAAITTTLTVGSLGV  65
60  WWSAVTMSTVGYGDKSPITAWGRLLSVVWIFTGVIIISGLTAGISSSLTVHQLKT  94
    WfalVTmtTvGYGDkaPitk-Grlva-vWmv-sli-issltAgias-ltv-----  consensus
```

X non conserved

X similar

X conserved

X all match

group 2 S1 region alignment

```
                   10        20        30        40        50        60
                    .         .         .         .         .         .
 1  LVVATRAIPPFVFN.DNGELSGFSIDLWRSIASQIGAESKFVEYPNVADLLSSVRNGKAN  3
 1  LVVATRAIPPFVFN.DNGELSGFSIDLWRSIASQIGAESKFVEYPNVADLLSSVRNGKAN  2
 1  FVVATRAIPPFVFT.DNGELSGFSIDLWRSIASQIGAESKFVEYPSVPDLLSSVQNGKAN  1
 1  LLVATRVIPPFVLS.NKGELSGFSIDLWRSIATQIGIESKLIEYSSVPELISAIKDNKVN  4
 1  VRVATRVLPPFVMD.ERGKLNGFSIELWERIAERIGVTTEYVVRPNVTELLSTVRDGKAD  6
 1  LVVNTVTRPPFSMP.VGEHETGFTMDLIREVSQRLGWDIQINRVDNFAQMLEEVERGEAD  9
 1  LQVTTVTRPPFSFV.EDGVETGFSMDLLAALADKLGLGYTLERKEKFSDMLAAVMDGEAD  5
 1  IKVGSYHCPPFVIHENDEDYTGLTILLWEQIAKRLNLQYEIKQYP.LTELLDAVAEGKIN  50
 1  LKVGAYHCPPFIFESSEEQYDGLSMILWQRIAEIMQVEYKVSHYG.LQELLSAAEVGEID  42
 1  LVVGVYVSPPFVEEHDDGNYSGMAIDLWETVADRMDLAFRYATYPTFGSLVRATASGEVD  63
    lvVatr-iPPFvf--d-gelsGfsidLwrsia-riglesk-v-yp-v-ellsav--gka-  consensus

                   70        80        90
                    .         .         .
60  AGIAAISITAERQQQFDFSLPMFSGGLQILVRN  3
60  AGIAAISITAERQQQFDFSLPMFSGGLQILVRN  2
60  AGIAAISITAERQQQFDFSLPMFSGGLQILVRN  1
60  LGIAAISITAEREQNFDFSLPIFASGLQIMVRN  4
60  LAISAISITAQREAQFDFSHPMYDSGLAILVQV  6
60  MAAANISITAGREAVMDFSQPIFESGLQIMLHQ  9
60  MAIANISITAARETEMDFSQPIFESGLQILVPA  5
60  IGISCISITPEREERLDFSHSFYETHLAIAVKK  50
60  VGVSCISITPDREEHVDFSHSFYETHLAIAVKN  42
61  AAVTNLTITRERAELIAFTQPWYDAGLRIMVPS  63
    mgiaaisITaeRe-qfdFs-pmfesgLqIlvrn  consensus
```

X non conserved
X similar
X conserved
X all match

group 2 S2 alignment

```
                          10        20        30        40        50        60
  1  DINSVSDLPGRVVATTTGSTAATYLKQQKISVLEVPKIEQAYDALETKKAEAVVFDSPVL  3
  1  DINSVSDLPGRVVATTTGSTAATYLKQQKISVLEVPKIEQAYDALETKKAEAVVFDSPVL  2
  1  DINSVSDLPGRVVATTTGSTAATYLKQQKISVLEVPKIEQAYDALETKKAEAVVFDSPVL  1
  1  DIRSIDDLPGKVVATTAGSTAATYLREHHISVLEVPKIEEAYKALQTKKADAVVFDAPVL  4
  1  GISSPSDLIGKTVASVEGSTAAKYLATLRVDTLVFPTVAEAIASTESGKTSAVVYDSPVL  6
  1  SVNSVNDLYGKRVGTIQGSTAAGFLDRREIDYVAFPGLDKMLEDFEDGDTRIVVFDAPVL  9
  1  SVNSVNDLYGQEVGTIDGSTAAGFLTRRDIDFAAFSDLQEMITAFERGALDAIVFDAPIL  5
  1  GISALSDLPGNTVGVLSGSVAEQYMRDMGIATRSYVNIDASVVALREGRLDAVVGDAPIL  63
  1  DIKNPNDLANIKTGAKKSSTSSQFLTNHSIVHKAYNTVEDMLVALEKGEIEAVIADDPVL  50
  1  DVKGINDLSKVKTGAKQGTTASLLLSNHSIAHDTYKDMDALLLALEKGQLDAVVADDAVL  42
     dinsvsDLpgk-vgt--gstaa-ylkq--i--l-yp-ie-al-aletgk-davvfD-pvL  consensus

                          70        80        90       100       110       120
 61  LFYAANQGQG....KVEVVGSVFREENYGIVLPNNSPYRKPINNALLKLKENGTYQSLYD  3
 61  LFYAANQGQG....KVEVVGSVFREENYGIVLPNNSPYRKPINNALLKLKENGTYQSLYD  2
 61  LFYAANQGQG....KVEVVGSVFREENYGIVLPNNSPYRKPINNALLKLKENGTYQSLYD  1
 61  LFYAANEGKG....KVEIVGSILREESYGIILPNNSPYRKPINQALLNLKENGTYQSLYD  4
 61  RYFASHDGRG....RMKLAGPTFREEKYGIAFKQNSTLRKRVNEELLRLREDGTYGDMQV  6
 61  NHYVKTDGAG....IGHVMGRKFLTEYYGIIFPQGSDMVEPFNRALLKMQEDGAYETLYR  9
 61  SYYASHQGKG....KAVTTGAIFLRENYGIAFPTGSARVEEVNQALLALREDGTYEELYR  5
 61  EHYVLTHQKD....NLAVVGNLFHPDKYGFAFPHESNLTTPTTLELLDLQEDGTLGNLRL  63
 61  KYQIKKAKERGVYTDMIVLPYQFEKQNYGFAIEDNSPYEEELNRALLKIRQSIEWRQALN  50
 61  RYMIKKGKGKDLFLDLTVLPYQLEKQNYGLVLIENSPYEEKINRALLQVRETNEWHQILL  42
     -yya---g-g----kv-vvg-vfreenYGivlpnnSpyrkpinnaLLklre-gty-sly-  consensus

117  KWFGVKNS   3
117  KWFGVKNS   2
117  KWFGVKDS   1
117  KWFDPKNS   4
117  RWFGTEDNS  6
117  KWFGQSF    9
117  KWFGRRN    5
117  EYFGSRN    63
121  KYFTDIP    50
121  EYFAEK     42
     kwFg-kn--  consensus
```

X non conserved

X similar

X conserved

X all match

group 2 channel region alignment

```
                  10        20        30        40        50        60
  1  PKLSNSGTPNILSLFFSTTILQVLGLALFLIVIAAHVIWLSERHQKEGM...ISPSYFPG  3
  1  PKLSNSGTPNILSLFFSTTILQVLGLALFLIVIAAHVIWLSERHQKEGM...ISPSYFPG  2
  1  PKLSNSGTPNILSLFLSTTILQVLGLALLLIVIAAHVIWLSERHQKEGM...ISQSYFPG  1
  1  LESKNSAFPNILQLFFSTSLLQVIGVALVLIVIAAHIIWLSERNHKEGM...ISESYFPG  4
  1   GAQHQSFMDIV.IQWSTQLLPAMGLGFILVMIPAHLIWFIERRGESDLP..VHRSYYPG  6
  1     DDVSEPSLISALMSWDLFAAIAIAFVLLLVGGMLMWVFERRAQPYFDRPLKDAWFP.  9
  1     ETNAGSSLLRALLSRDLFLAIGAAFVILLTGGMLMWYFERRAQPYFDRKLHEAWFP.  5
  1      QGYLASIINILTNKKLLLVLAILLCAAALIGSVFYFLEHKINDKLY..SMQTRPAR  50
  1      RSYLDSFISIITNKTLWKAICLFLFVAISVGTFYYLLERKNNDKLY..SMPTKRGR  42
  1    QGGGGFRGMLNGLQNAGHLRAYIFLLSMILIATVGLSLFDRRFDPDHP....RRWRES  63
     ----nsg-p-ilsll-sttll--iglalvliviaahviwl-err--e-m---i--syfpg  consensus

                  70        80        90       100       110       120
 58  IFKACWWAAATLAT..QADEMPKGVIGRILAIIWMFIGVLFVTYFTAAATTSLTVQQLQA  3
 58  IFKACWWAAATLAT..QADEMPKGVIGRILAIIWMFIGVLFVTYFTAAATTSLTVQQLQA  2
 58  IFKACWWAAATLAT..QADEMPKGVIGRILAIIWMFIGVLFVTYFTAAATTSLTVQQLQA  1
 58  IFKACWWAAATLAT..QADEMPKGVLGRFIAIVWMFIGVLFVAYFTASATTSLTVQQLQG  4
 57  IVLAAYWVATLMGG..QADPMPTRRASRVVAVIGIYVGLVFVAYFTAYATSMLTVQQLKS  6
 57  ...SFWWALNLVVNGGFEERVPRSALGRVFGVLLVLSSLFVVSVFVAKITAAMTVEAING  9
 57  ...SFWWALNLVVNGGFEERVPRTAFGRLFGVLLVISSLFVVSVFTARITSVMTVEAISG  5
 55  FVEGFILGLLFITKGPFNYFEFKTLSGRVLTVIIAIFSTLFIASITAVLASTFTLGLLNS  50
 55  IAEAFILGVLFITKGPFNYFEFKTLSARVVTVLIAMFSTVFIASITAVLASSLTLEQLRF  42
 55  IAESFYQVMLIITSGRTARKNLFGWVGRIWQALWLIVGVAVIAYITSSVTSVMTTVSLTQ  63
     i--afwwaa--latg-qad-mpkgvigRilaviwm-igvlfvayfta-atsslTvqqlqg  consensus
```

X non conserved
X similar
X conserved
X all match
